# Sugar‐Armored Pesticides: Self‐Assembled System for Enhanced Foliar Adhesion and Sustained Delivery of Hydrophobic Antimicrobials Against Bacterial Diseases

**DOI:** 10.1002/advs.202524286

**Published:** 2026-01-28

**Authors:** Jinghan Yang, Juan Liu, Xiaohui Wang, Peiyi Wang

**Affiliations:** ^1^ State Key Laboratory of Green Pesticide Key Laboratory of Green Pesticide and Agricultural Bioengineering Ministry of Education Center For Research and Development of Fine Chemicals of Guizhou University Guiyang China

**Keywords:** bactericidal activity, foliar adhesion, hydrophobic pesticides, intelligent delivery, supramolecular self‐assembly

## Abstract

Naturally hydrophobic characteristics of most pesticides seriously affect their effective deposition and bioavailability on susceptible plants, causing inevitable off‐target movement and environmental pollution. To solve this problem, we employ supramolecular self‐assembly strategies to deliver and remedy such defective pesticides, thereby creating a self‐assembled, multifunctional delivery system—PyE28@HP‐*β*‐CD, which comprises a high‐performance pyridyl ether microbicide (PyE28) complexed with 2‐hydroxypropyl‐*β*‐cyclodextrin (HP‐*β*‐CD). This approach transforms insoluble hexagonal sheet structures (PyE28) into biocompatible sugar‐armored spherical assemblies (PyE28@HP‐*β*‐CD) with sustained release profiles in aqueous environment, ultimately improving the physicochemical/biological properties and targeted delivery efficiency of hydrophobic pesticides. The advancement concretely manifests as: (i) markedly enhanced foliar deposition via improved wetting coverage and reduced interfacial tension (contact angle decreased by 18°), (ii) effective biofilm disruption (achieving an 81.40% biofilm reduction at 23.3 µg mL^−1^) through suppression of extracellular polysaccharide biosynthesis, and (iii) superior bactericidal activity against *Xanthomonas* pathogens mediated by oxidative damage amplification and redox homeostasis interference. In vivo evaluations demonstrated that PyE28@HP‐*β*‐CD at 200 µg mL^−1^ effectively suppressed citrus canker and rice bacterial blight by 79.73% and 45.53%, respectively, superior to conventional bactericides by 30.76% and 20.33%. This study presents a supramolecular self‐assembly approach to optimize hydrophobic agrochemical delivery, combining sustained release with on‐demand precision.

## Graphical abstract

This study develops a sugar‐armored, self‐assembled pesticide delivery system that enhances foliar adhesion, provides sustained release, effectively disrupts biofilms, and demonstrates superior bactericidal activity for improved disease control.

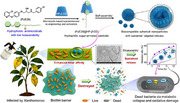

## 1 Introduction

Harmful invasive phytopathogenic bacteria cause devastating diseases in plants that severely reduce crop yield and quality. Among these pathogens, *Xanthomonas* species are particularly notorious, being associated with approximately 400 different crop diseases [1]. For example, *Xanthomonas axonopodis* pv. *citri* (*Xac*) infiltrates plant tissues through stomata or wounds, leading to the formation of pustules and characteristic raised canker lesions [2]. These symptoms ultimately result in fruit drop and diminished citrus quality [3, 4]. Similarly, *Xanthomonas oryzae* pv. *oryzae* (*Xoo*) devastates rice production through leaf discoloration, wilting, and photosynthetic suppression, leading to catastrophic yield losses [5]. For decades, combating these resilient pathogens has posed a persistent challenge, as they consistently evade conventional control measures. Their extraordinary survival capability originates from a sophisticated defense strategy: biofilm formation [6]. Biofilms are structured microbial communities encased in a self‐secreted extracellular polymeric substance (EPS) matrix—a robust scaffold consisting of proteins, polysaccharides, and lipids [7]. This matrix not only establishes a physical barrier but also facilitates irreversible adhesion to plant surfaces. More alarmingly, biofilm maturation triggers an exponential surge in antimicrobial resistance, orchestrated through sophisticated quorum‐sensing networks that precisely regulate virulence expression and collective defense mechanisms [8]. Despite remarkable progress in agricultural science, most commercial agrochemicals fail to penetrate or disrupt established biofilms effectively [9]. The absence of a definitive solution highlights the critical demand for novel strategies to overcome these impervious microbial fortresses and mitigate their crop‐devastating consequences.

On the other hand, the inherently hydrophobic nature of most pesticides severely limits their effective deposition, bioactivity, and bioavailability on target plants, leading to off‐target drift and environmental contamination [10, 11, 12]. To address these challenges, nano‐formulation strategies—such as the application of pesticide adjuvants (e.g., surfactants and wetting agents) or the use of nanocarrier‐based delivery systems (e.g., silica nanoparticles and metal–organic frameworks)—have been widely explored and demonstrated to be effective [13, 14, 15, 16, 17, 18]. However, such approaches exhibit certain limitations: adjuvants frequently rely on excessive organic solvents and surfactants, which aggravate environmental burdens, while nanocarrier systems introduce high production costs and operational complexity, hindering large‐scale adoption [19, 20, 21]. In this context, supramolecular self‐assembly has emerged as a transformative alternative. By leveraging molecular recognition and spontaneous organization, this strategy not only enhances pesticide delivery efficiency but also rectifies the suboptimal physicochemical properties (e.g., solubility, stability) and biological performance of hydrophobic pesticides, all while minimizing synthetic complexity and environmental impact. This approach represents a pivotal advancement toward sustainable agriculture, balancing efficacy with ecological safety.

Supramolecular self‐assembly focuses on constructing programmable multimolecular systems through synergistic non‐covalent interactions, such as hydrogen bonding, host‐guest recognition, hydrophobic effects, *π*–*π* stacking, and van der Waals forces [22]. Beyond enabling the fabrication of diverse topological nanostructures, this approach exhibits intrinsic dynamic reversibility and sustained release kinetics, which collectively provide unparalleled versatility in functional material engineering [23, 24, 25, 26, 27]. Critically, the precise orchestration of these molecular interactions progressively modulates their absorption and metabolic behaviors in biological systems, ultimately enhancing bioavailability and biocompatibility. In supramolecular systems, host‐guest recognition represents a highly efficient and versatile platform enabled by macrocyclic hosts' selective molecular encapsulation [28]. This process generates multifunctional building blocks that integrate self‐assembly behavior with enhanced physicochemical and biological properties. Among common macrocyclic hosts, oligosaccharide‐based cyclodextrins (CDs), composed of 6–8 glucose units linked by *β*‐1,4‐glycosidic bonds, are particularly advantageous due to their unique amphiphilic architecture (hydrophobic interior/hydrophilic exterior), exceptional molecular selectivity, outstanding biocompatibility, superior chemical stability, and inherent ecological safety and biodegradability [29]. These characteristics enable CDs to form host‐guest inclusion complexes with various hydrophobic molecules, which subsequently self‐assemble into structurally tunable aggregates. Notably, 2‐hydroxypropyl‐*β*‐cyclodextrin (HP‐*β*‐CD) has been extensively documented to possess remarkable advantages, including enhanced water solubility, superior inclusion capacity, and broad‐spectrum molecular encapsulation capability [30, 31]. These properties make HP‐*β*‐CD particularly effective for optimizing compound performance, establishing it as a readily accessible macrocyclic host for targeted molecular delivery systems.

**SCHEME 1:**
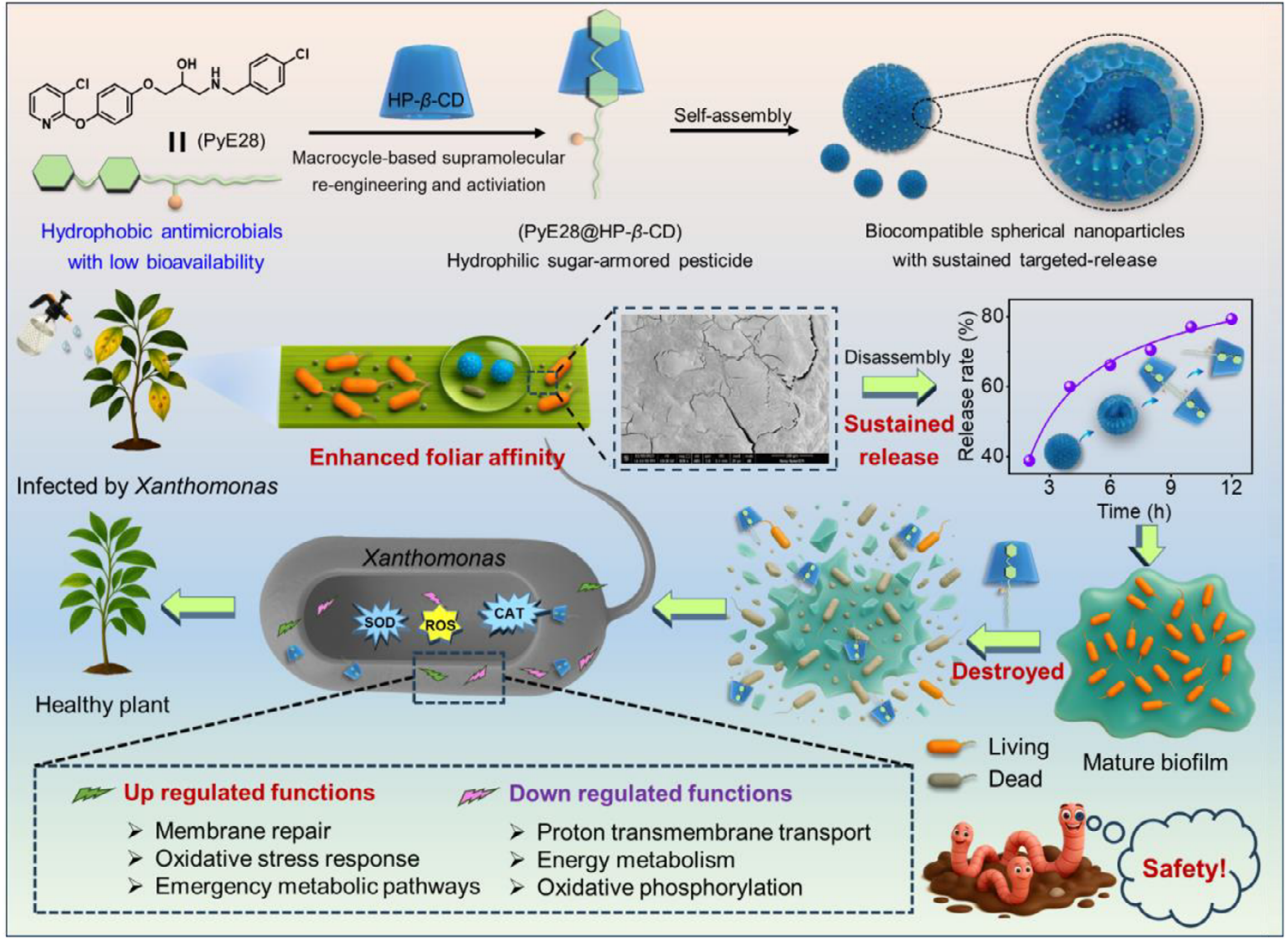
Schematic diagram of engineering sugar‐armored supramolecular self‐assembly systems for robust foliar adhesion and sustained delivery of hydrophobic antimicrobials against bacterial plant diseases.

To develop highly efficient, eco‐friendly bactericidal formulations while improving pesticide utilization, we propose constructing a biocompatible, sugar‐armored supramolecular self‐assembly system with sustained release properties. This system is designed to simultaneously enhance the physicochemical/biological properties and targeted delivery efficiency of hydrophobic pesticides, ultimately providing a comprehensive control against bacterial plant diseases (**Scheme** **1**). Building upon this design framework, we first developed a series of structurally innovative pyridyl ether bactericides, identifying PyE28—containing water‐repellent 4‐chlorobenzyl and 4‐((3‐chloropyridin‐2‐yl)oxy)phenoxy groups—as the most active compound. This hydrophobic molecule naturally forms insoluble hexagonal microstructures in aqueous environments, inevitably compromising its foliar adhesion efficiency and bioavailability to target pathogens. Subsequently, through supramolecular engineering, we constructed a macrocycle‐based delivery system (PyE28@HP‐*β*‐CD) via HP‐*β*‐CD‐involved host‐guest complexation, transforming insoluble crystalline structures into nanoscale spherical assemblies with enhanced aqueous compatibility, sustained release properties, and biocompatible carbohydrate surfaces. Functional evaluations demonstrated that PyE28@HP‐*β*‐CD exhibited reduced interfacial tension and superior wetting/adhesion capabilities, ensuring efficient delivery to susceptible plant surfaces. The system also effectively suppresses biofilm formation by inhibiting extracellular polysaccharide biosynthesis, while showing enhanced bactericidal activity against *Xanthomonas* pathogens through oxidative stress induction and redox homeostasis disruption. Furthermore, transcriptomic analysis was used to elucidate the supramolecular bactericidal mechanism. Safety evaluations revealed negligible impacts on crop growth and non‐target soil organisms (e.g., earthworms), supporting its environmental compatibility. Pot experiments validated PyE28@HP‐*β*‐CD's superior efficacy in controlling intractable bacterial diseases, underscoring both the design concept and its potential applicability for sustainable crop protection.

## 2 Results and Discussion

### 2.1 Identifying the Pyridyl Ether Derivative—PyE28 as the Supreme Bactericide

**SCHEME 2:**
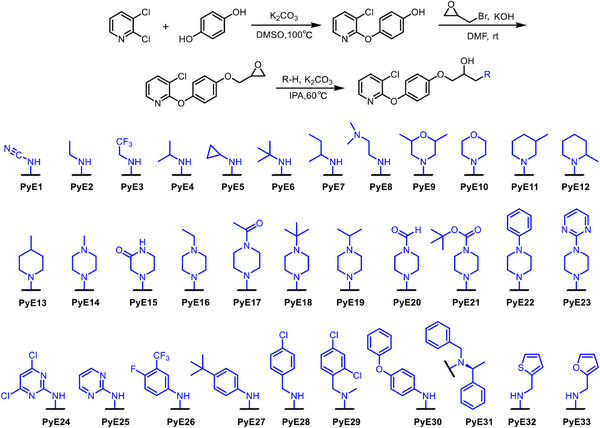
The synthetic route for target molecules PyE1‐PyE33.

The scarcity of agrochemicals capable of targeting both bacteria and biofilms highlights the demand for dual‐functional agents with combined bactericidal and anti‐biofilm effects. To address this critical gap, we designed a series of pyridyl ether derivatives by strategically combining the versatile isopropanolamine pharmacophore with a phenoxypyridine ether scaffold—both structural motifs well‐established in antimicrobial and anti‐biofilm applications. A straightforward and efficient three‐step synthetic route was developed to access these bactericidal compounds, as outlined in Scheme 2. First, 2,3‐dichloropyridine was employed as the starting material and underwent nucleophilic aromatic substitution with hydroquinone in the presence of potassium carbonate in dimethyl sulfoxide (DMSO) at 100°C, affording the key intermediate 4‐((3‐chloropyridin‐2‐yl)oxy)phenol. Subsequently, this phenolic intermediate was reacted with epibromohydrin under basic conditions (KOH/DMF) at room temperature to construct the epoxide‐containing framework, yielding 3‐chloro‐2‐(4‐(oxiran‐2‐ylmethoxy)phenoxy)pyridine. Finally, structural diversification was achieved through epoxide ring‐opening reactions with various amine nucleophiles, generating a series of phenoxypyridine ether derivatives featuring the crucial isopropanolamine pharmacophore. All designed compounds (PyE1‐PyE33) were unequivocally characterized by ^1^H/^13^C/^19^F NMR spectroscopy and HRMS (Figures S16–S116). Preliminary/further assessment of bactericidal activity was performed using the standard turbidimetric assay, with structure‐activity relationships as detailed in Table 1. In the anti‐*Xac* activity assay, fifteen compounds demonstrated potent inhibitory effects, with EC_50_ values ranging from 2.91 to 27.3 µg mL^−1^, significantly outperforming the commercial bactericide thiodiazole‐copper (TC, 84.2 µg mL^−1^). This outcome supports the soundness of the initial molecular design strategy. A brief structure‐activity relationship (SAR) against *Xac* is summarized below: (i) Attachment of a pyrimidine ring to the piperazine moiety boosts anti‐*Xac* performance, such as PyE23 (a pyrimidine ring, EC_50_ = 6.00 µg mL^−1^) vs. PyE22 (a benzene ring, EC_50_> 100 µg mL^−1^); (ii) The antibacterial potency followed the trend: meta‐methyl (PyE11, EC_50_ = 8.83 µg mL^−1^) > ortho‐methyl (PyE12, EC_50_ = 10.5 µg mL^−1^) ≫ para‐methyl (PyE13, EC_50_>100 µg mL^−1^), highlighting the critical role of substitution position; (iii) The compound bearing a 4‐chlorobenzylamino group (PyE28, EC_50_ = 2.91 µg mL^−1^) exhibited superior anti‐*Xac* activity to those with heterocyclic substitutions, including thiophen‐2‐ylmethylamino (PyE32, EC_50_ = 6.72 µg mL^−1^) and furan‐2‐ylmethylamino (PyE33, EC_50_ = 5.95 µg mL^−1^).

**TABLE 1:** EC_50_ values of compounds PyE1‐PyE33 against *Xac* in vitro.

Compounds	Regression equation	EC_50_(µg mL^−1^)	Compounds	Regression equation	EC_50_(µg mL^−1^)
PyE1	y = 1.640x + 3.627	6.87 ± 0.06	PyE18	y = 0.939x + 4.018	11.1 ± 0.2
PyE2	/	> 50	PyE19	y = 1.416x + 2.967	27.3 ± 0.7
PyE3	/	> 50	PyE20	/	> 100
PyE4	y = 1.643x + 3.489	8.31 ± 0.25	PyE21	y = 0.878x + 4.283	6.56 ± 0.27
PyE5	y = 2.447x + 2.738	8.40 ± 0.12	PyE22	/	> 100
PyE6	y = 1.013x + 4.101	7.73 ± 0.27	PyE23	y = 0.705x + 4.451	6.00 ± 0.62
PyE7	y = 1.907x + 2.548	19.3 ± 0.2	PyE24	/	> 100
PyE8	y = 2.474x + 1.9067	17.8 ± 0.1	PyE25	/	> 100
PyE9	/	> 100	PyE26	/	> 50
PyE10	/	> 50	PyE27	/	> 100
PyE11	y = 1.076x + 3.982	8.83 ± 0.05	PyE28	y = 2.541x + 3.818	2.91 ± 0.01
PyE12	y = 1.939x +3.021	10.5 ± 0.5	PyE29	/	> 100
PyE13	/	> 100	PyE30	/	> 100
PyE14	/	> 50	PyE31	/	> 100
PyE15	/	> 50	PyE32	y = 2.076x + 3.282	6.72 ± 0.16
PyE16	/	> 50	PyE33	y = 2.604x + 2.983	5.95 ± 0.7
PyE17	/	> 100	TC	y = 1.687x + 1.753	84.2 ± 1.9

*Note*:EC_50_values of bactericidal effects are indicated as means ± SD (standard deviation); Commercialized bactericide thiodiazole‐copper (abbreviation:TC) as the positive control.

Based on the above analysis, compound PyE28 was identified as the most potent bactericide (anti‐*Xac*, EC_50_ = 2.91 µg mL^−1^). Its structure features hydrophobic 4‐chlorobenzyl and 4‐((3‐chloropyridin‐2‐yl)oxy)phenoxy groups, rendering it inherently water‐repellent. In aqueous environments, PyE28 tends to form insoluble hexagonal microstructures, significantly reducing its foliar adhesion and bioavailability to target pathogens. To overcome this limitation, we explored a facile macrocycle‐based supramolecular self‐assembly strategy to enable efficient delivery and functional remediation of this hydrophobic pesticide. This approach is expected to yield a self‐assembled, biocompatible, and multifunctional delivery system with clearly enhanced performance.

### 2.2 Preparation of a Macrocycle‐Based Self‐Assembled Delivery System (PyE28@HP‐*β*‐CD) for Sustained Pesticide Release

To address the delivery challenges of hydrophobic pesticides, we developed a multifunctional system via the facile supramolecular self‐assembly of PyE28 within HP‐*β*‐CD. This biocompatible sugar‐armored complex exhibits a sustained release profile, enabling effective pesticide delivery. The preparation process was simple and reproducible. Briefly, PyE28 in dimethyl sulfoxide (12 µL, 0.12 m) was added dropwise to an aqueous HP‐*β*‐CD solution (3.00  mL, 0.48 mm) with stirring. After thorough mixing, the supramolecular aggregate assembled from PyE28@HP‐*β*‐CD was obtained, having a final PyE28 concentration of 200 µg mL^−1^ (molar ratio, PyE28:HP‐*β*‐CD = 1:1). Subsequently, the self‐assembly process, the final morphology, and the sustained release properties were systematically investigated using multiple analytical techniques.

**FIGURE 1:**
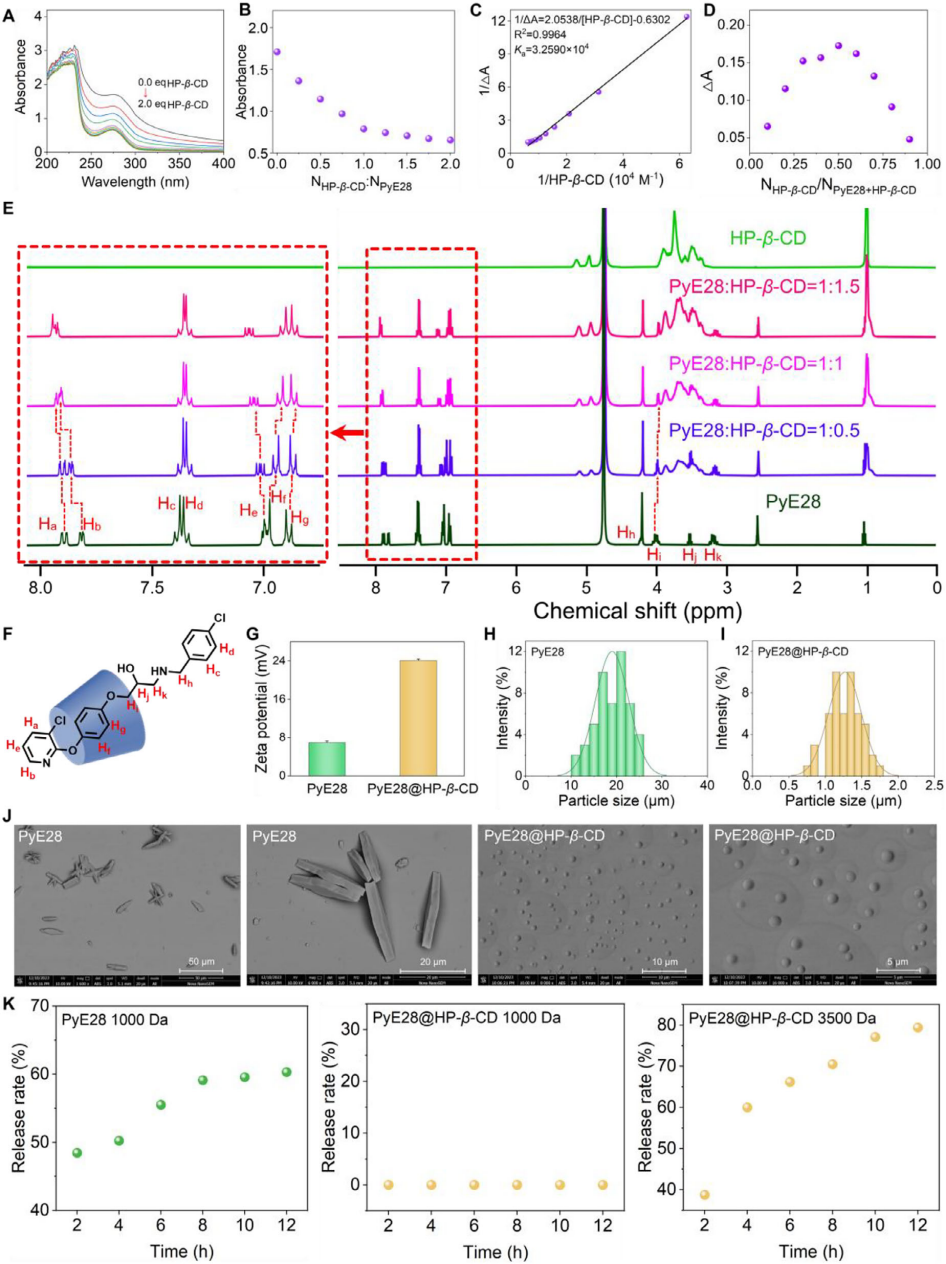
UV–vis absorption spectra of PyE28 (0.16 mm) were recorded with gradually increasing concentrations of HP‐*β*‐CD (0.01–0.32 mm) in aqueous solution (A). The absorbance change of PyE28 at 276 nm as a function of the HP‐*β*‐CD/PyE28 molar ratio is shown in (B). The binding constant was determined using the Benesi–Hildebrand relationship derived from reciprocal absorbance variations (C), while the stoichiometry of the complex was confirmed by Job's plot at 276 nm with a total concentration of 0.12 mm (D). ^1^H NMR titration spectra of PyE28, HP‐*β*‐CD, and their mixtures at molar ratios of 1:0.5, 1:1, and 1:1.5 in D_2_O (C_PyE28_ = 5.0 mm) are presented in (E). The proposed host–guest binding model between PyE28 and HP‐*β*‐CD is illustrated in (F). Zeta potentials (G) and particle size distributions obtained by DLS (H–I) of PyE28 and PyE28@HP‐*β*‐CD were measured at 200 µg mL^−1^, and the corresponding surface morphologies are shown by SEM images in (J). The release behaviors of PyE28 and PyE28@HP‐*β*‐CD through dialysis membranes with molecular weight cut‐offs of 1 and 3.5 kDa are presented in (K).

First, the possible interactions, binding forces, optimal binding ratio, and encapsulation sites between PyE28 and HP‑*β*‑CD were thoroughly explored. As shown in Figure 1A,B, the gradual addition of HP‐*β*‐CD resulted in a continuous decrease in the absorbance of PyE28 at 276 nm, which plateaued at a 1:1 molar ratio. This observation suggests a 1:1 binding stoichiometry between PyE28 and HP‐*β*‐CD. This hypochromic effect is attributed to the progressive encapsulation of specific aromatic groups of PyE28 within the hydrophobic cavity of HP‐*β*‐CD. Meanwhile, the binding constant (*K*_a_) of the PyE28/HP‐*β*‐CD complex was determined using the Benesi–Hildebrand method, yielding a value of 3.259 × 10^4^ M^−1^, based on the linear relationship expressed as 1/△A = 2.0538/[HP‐*β*‐CD]‐0.6302 (Figure 1C). This value suggests a favorable binding affinity that is consistent with the inclusion complexes formed between CDs and aromatic guests [32]. Furthermore, Job's plot analysis was performed to confirm the binding stoichiometry. The absorbance difference (ΔA) reached a maximum when the mole fraction of PyE28 was 0.5, agreeing with a 1:1 inclusion complex between PyE28 and HP‐*β*‐CD (Figure 1D). This inference was further corroborated by high‑resolution mass spectrometry, which detected a prominent peak at 1959.7443 (m/z) corresponding to the [PyE28 + HP‑*β*‑CD + H^+^] complex (Figure S1). This result provides compelling evidence for the assembly of the host and guest molecules in a 1:1 stoichiometric ratio.

Additionally, potential recognition sites between PyE28 and HP‐*β*‐CD were analyzed using ^1^H NMR. As illustrated in Figure 1E,F and Table S1, the initial chemical shifts of the H_f_, H_g_, and H_i_ protons of PyE28 were 7.063, 6.971, and 4.048 ppm, respectively. Upon the addition of 1.0 equivalent of HP‑*β*‑CD, these protons exhibited a significant upfield shift to 7.021, 6.966, and 4.036 ppm, with corresponding changes of −0.042, −0.005, and −0.012 ppm. This purposeful movement indicates that these protons are embedded in the hydrophobic cavity of HP‑*β*‑CD and experience a strong shielding effect, which enhances the local magnetic environment. When the HP‑*β*‑CD concentration was further increased to 1.5 equivalents, the chemical shifts of these protons remained nearly unchanged, suggesting that the host–guest complex reached an approximate 1:1 stoichiometry at equilibrium. Meanwhile, the initial chemical shifts of H_a_, H_b_, and H_e_ protons of PyE28 were 7.911, 7.837, and 7.074 ppm, respectively. Upon the addition of 1.0 equivalent of HP‑*β*‑CD, these protons shifted downfield to 7.958, 7.939, and 7.133 ppm, with corresponding changes of + 0.047, + 0.102, and + 0.059 ppm. This consistent downfield shift suggests that these protons reside outside the cyclodextrin cavity and experience a deshielding environment, likely due to reduced local electron density or conformational restructuring upon complex formation. When the HP‑*β*‑CD concentration was further increased to 1.5 equivalents, the chemical shifts of these protons remained constant, indirectly supporting the formation of a 1:1 host–guest inclusion complex with a well‐defined encapsulation site.

The ζ‑potentials of free PyE28 and the PyE28@HP‑*β*‑CD complex were measured to be −6.99 mV and −24.12 mV, respectively (Figure 1G). The pronounced increase in the absolute ζ‐potential value indicates that host–guest complexation significantly enhances the colloidal stability of the supramolecular assembly [33]. scanning electron microscopy (SEM) images revealed a distinct microstructural transformation upon the conversion of the small molecule PyE28 into the supramolecular PyE28@HP‐*β*‐CD complex. While free PyE28 self‐assembled into 3D microsheets, the complex adopted a nanospherical morphology (Figure 1J). Statistical analysis indicated that free PyE28 in aqueous solution exhibited a broad particle size distribution ranging from 10.618 to 25.503 µm (Figure 1H). In contrast, after supramolecular complexation with the macrocyclic host HP‐*β*‐CD, the particle size decreased significantly to a narrow range of 0.743 to 1.741 µm (Figure 1I). This remarkable reduction in particle size can be primarily attributed to host–guest complexation, which greatly enhances the hydrophilicity, dispersion, and colloidal stability of PyE28 in aqueous media. The observed suppression of large aggregate formation and the subsequent reorganization into uniform nanospheres can be attributed to the structural features and binding behavior of PyE28. The aromatic rings of the phenoxypyridine moiety in PyE28 adopt a head‐to‐tail arrangement stabilized by *π*–*π* interactions, leading to the initial formation of layered structures. These layers further assemble into microsheets through intermolecular hydrogen bonding, as illustrated in Figure S2. Upon incorporation of HP‐*β*‐CD, the phenoxypyridine segment of PyE28 is encapsulated within the hydrophobic cavity of the cyclodextrin, while the chlorobenzene group remains solvent‐exposed, forming new supramolecular building blocks. Driven by molecular anisotropy, these subunits organize with the encapsulated regions oriented outward and the hydrophobic chlorobenzene moieties directed inward, ultimately resulting in the formation of well‐defined nanospheres, as depicted in Scheme 1. In summary, these comprehensive data confirm the successful construction of a novel supramolecular material, PyE28@HP‐*β*‐CD.

Ultimately, the release profiles of free PyE28 and its supramolecular complex PyE28@HP‐*β*‐CD were evaluated using dialysis membranes with different molecular weight cut‐offs (MWCOs) (Figure 1K; Figure S3). Free PyE28 (Mw = 419.30 Da) showed a cumulative release of 60.3% after 12 h when dialyzed against a 1000  Da membrane. In contrast, no detectable release of PyE28@HP‐*β*‐CD (Mw = 1960.85 Da) was observed under the same conditions, as the complex exceeded the membrane's size exclusion limit. When a membrane with a 3500  Da MWCO was used, the cumulative release of PyE28@HP‐*β*‐CD reached 79.4% at 12 h, significantly higher than that of free PyE28 under comparable conditions. This improvement can be attributed to the host–guest complexation, which enhances the aqueous solubility, dispersibility, and stability of PyE28, thereby facilitating more efficient diffusion during dialysis. To assess the storage stability of the supramolecular system, Tyndall effect tests and ζ‐potential measurements were conducted over 7 days (0, 1, 3, 5, and 7 days). As shown in Figure S4A, a clear Tyndall scattering effect was consistently observed, indicating the formation of a stable colloidal system. Moreover, the ζ‐potential remained nearly constant without significant fluctuation (Figure S4B), which collectively confirm the stability of the current system during storage.

Together, these findings demonstrate that the supramolecular complex PyE28@HP‐*β*‐CD effectively mitigates the inherent hydrophobicity of PyE28, leading to improved colloidal stability, enhanced dispersibility in aqueous media, and superior release performance—all of which contribute to its increased bioavailability. Based on these promising results, further studies will focus on evaluating the potential of this system to improve the interfacial properties of active ingredients on plant surfaces and its efficacy in controlling harmful microorganisms.

### 2.3 PyE28@HP‐*β*‐CD Droplets Exhibit Good Dispersion, Wetting, and Deposition on Citrus Leaves

Pesticides are typically applied in the field through spraying. However, when these droplets collide with plant leaves, the strong impact force and the inherent hydrophobic nature of the leaves cause the droplets to inevitably rebound, splash, and break apart. This not only prevents the pesticides from reaching their target areas and fulfilling their intended purpose but also leads to the pesticide active ingredients flowing beyond the plant's range, posing a serious threat to the ecological environment. Therefore, we utilize leaf spraying tests, contact angle measurements, surface tension analysis, rebound behavior observations, and SEM testing to evaluate the deposition characteristics of biocompatible PyE28@HP‐*β*‐CD droplets on citrus leaves.

**FIGURE 2:**
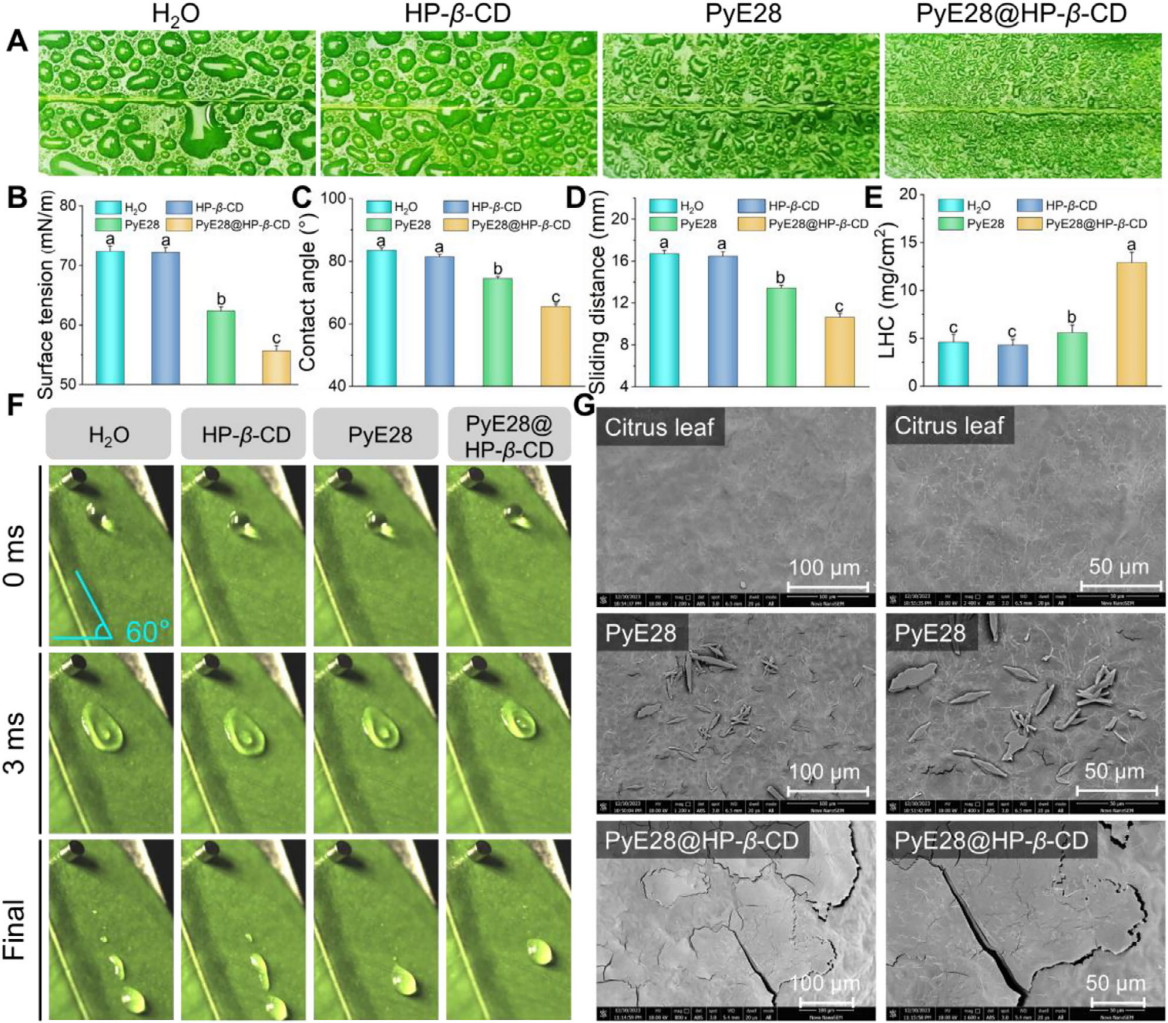
Representative images showing the spraying behavior of H_2_O, HP‐*β*‐CD, PyE28, and PyE28@HP‐*β*‐CD (200 µg mL^−1^) on citrus leaf surfaces (A). Surface tension measurements of the corresponding droplets on citrus leaves are summarized in (B), and their contact angles are presented in (C). The droplet sliding distances on inclined citrus leaves at the same concentration are shown in (D). Liquid holding capacities (LHC) of citrus leaves after immersion in H_2_O, HP‐*β*‐CD, PyE28, and PyE28@HP‐*β*‐CD (200 µg mL^−1^) are compared in (E). In (F), droplets of H_2_O, HP‐*β*‐CD, PyE28, and PyE28@HP‐*β*‐CD were released from a height of 10 cm onto citrus leaves inclined at 60°, and the spreading process was recorded by a high‐speed camera (Video S1; scale bar: 2 mm). SEM micrographs (G) display the surface morphology of untreated leaves and the deposition characteristics of PyE28 and PyE28@HP‐*β*‐CD droplets (200 µg mL^−1^) on the citrus leaf surface. Different lowercase letters denote statistically significant differences (*p* <0.05, *n* ≥3) as analyzed by one‐way ANOVA followed by Waller–Duncan post hoc test.

First, we sprayed its diluted solution, along with control solutions, onto citrus leaves (Figure 2A). Among all the treatment groups, the PyE28@HP‐*β*‐CD solution exhibited the finest and most evenly dispersed droplets compared to the larger water droplets observed in other groups. This indicates that the supramolecular system possesses excellent wetting and dispersion properties, which are beneficial for enhancing the adhesion of active ingredients on the leaf surface, thereby improving the bioavailability and efficacy of the pesticide against the target bacteria. To further explore the superior interfacial properties of the supramolecular system, we measured the surface tension and contact angle—key indicators of a liquid's wetting ability on a target surface, with lower values being more desirable. Consistent with this trend, PyE28@HP‐*β*‐CD demonstrated the lowest surface tension at 55.65 mN/m (Figure 2B), compared to PyE28 (62.37 mN/m), HP‐*β*‐CD (72.18 mN/m), and H_2_O (72.38 mN/m). This improved wettability was further confirmed by the contact angle measurement on citrus leaves (Figure 2C), where the PyE28@HP‐*β*‐CD solution registered 65.5°, markedly lower than PyE28 (74.5°), HP‐*β*‐CD (81.5°), and water (83.5°).

Next, we inclined citrus leaves at a 60° angle to the horizontal plane, released each solution from the same height, and recorded the sliding process using a high‐speed camera (Figure 2F and Video S1). The sliding distances of the droplets were quantified using i‐SPEED software, as shown in Figure 2D. Among the tested solutions, PyE28@HP‐*β*‐CD exhibited the shortest sliding distance of 10.7 mm, compared to PyE28 (13.4 mm), HP‐*β*‐CD (16.7 mm), and water (16.5 mm). Moreover, a leaf surface retention test was performed to evaluate the liquid‐holding capacity of various formulations on citrus leaves (Figure 2E). Impressively, PyE28@HP‐*β*‐CD achieved a retention value of 12.9 mg/cm^2^, which was substantially higher than that of the other formulations (4.3–5.6 mg/cm^2^), demonstrating enhanced adhesion and surface affinity. These results indicate that PyE28@HP‐*β*‐CD adheres more effectively to the leaf surface. Synchronously, SEM images provide vivid microscopic evidence of differential ingredient retention. PyE28 deposits as isolated, flake‐like sheets on the leaf surface. In stark contrast, the PyE28@HP‐*β*‐CD complex coalesces into a robust, continuous film that ensures uniform coating (Figure 2G). This superior performance is attributable to enhanced intermolecular interactions—such as host‐guest encapsulation, hydrogen bonding, and hydrophobic forces—which boost solution viscosity and cohesion to resist droplet sliding. Moreover, HP‐*β*‐CD markedly improves PyE28 dispersibility, facilitating conformal contact with the leaf's micro‐features. Together, these traits minimize runoff, extend residence time, and maximize pathogen‐targeting bioavailability.

To evaluate the practical rainfastness of the formulation, we simulated environmental water exposure using a wash‐off experiment. PyE28 and its supramolecular complex PyE28@HP‐*β*‐CD were first dyed with Rhodamine 6G (0.1%) for visualization. As shown in Figure S5A, the color intensity of PyE28@HP‐*β*‐CD was markedly higher than that of PyE28 both before and after washing, under both natural and UV light. This visual evidence was corroborated by SEM images (Figure S5B), which revealed that the PyE28@HP‐*β*‐CD coating remained largely intact on the leaf surface post‐wash, whereas PyE28 was largely removed. These results collectively demonstrate that the supramolecular formulation possesses significantly stronger rain wash‐off resistance, ensuring longer‐lasting activity under challenging conditions.

In summary, the PyE28@HP‐*β*‐CD system successfully overcomes key application drawbacks like poor wettability and weak adhesion, thereby significantly elevating spray efficiency and reducing waste.

### 2.4 PyE28@HP‐*β*‐CD Enhances the Inhibition and Eradication Ability Toward *Xac*‐Biofilm

**FIGURE 3:**
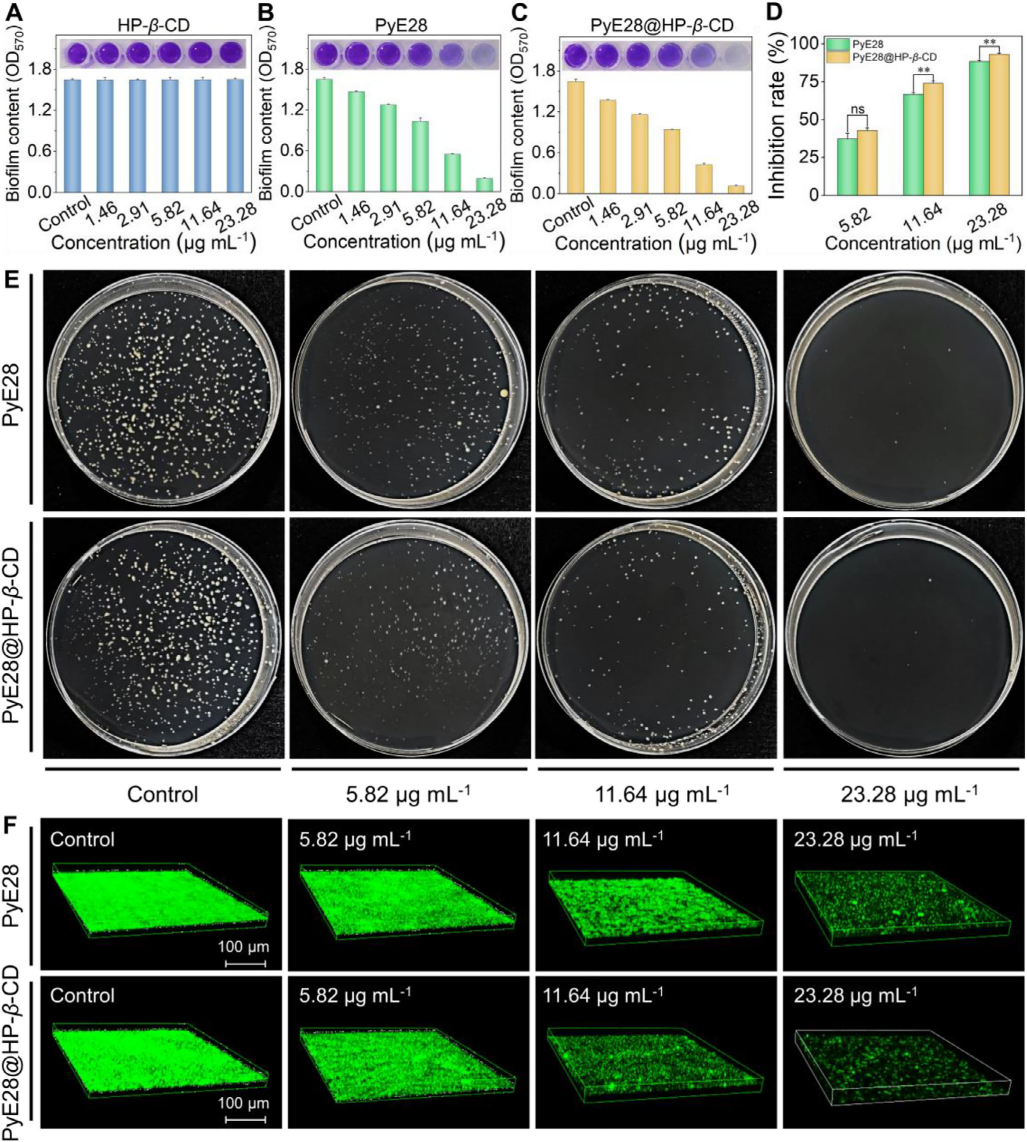
The anti‐biofilm and bactericidal properties of PyE28@HP‐*β*‐CD. (A–D) The photograph of *Xac* biofilms after co‐incubation following crystal violet staining and the corresponding optical density (OD) values at 570 nm for biofilm quantification with different concentrations of HP‐*β*‐CD (A), PyE28 (B) PyE28@HP‐*β*‐CD (C), and the corresponding biofilm formation inhibition rates (D). (E) Representative photographs of *Xac* colonies within the biofilm on agar plates treated with PyE28 and PyE28@HP‐*β*‐CD for 48 h. (F) 3D confocal laser scanning microscopy (CLSM) images of *Xac* strains treated with DMSO, HP‐*β*‐CD, and various concentrations of PyE28 and PyE28@HP‐*β*‐CD (5.82, 11.64, and 23.28 µg mL^−1^) for 48 h, further stained with acridine orange (AO, for live bacteria with green fluorescence), scale bar = 100 µm. Statistical analysis was conducted using an independent‐samples *t*‐test in (D), and significance levels are indicated as *p* < 0.05 (^*^), *p* < 0.01 (^**^), *p* < 0.001 (^***^), and not significant (ns), with *n* ≥ 3 for all experiments.

Bacterial biofilm formation initiates with bacteria adhering to biotic or abiotic surfaces, subsequently entering phases of growth and reproduction. Utilizing the classical crystal violet staining method [34], we assessed the inhibitory activity of PyE28@HP‐*β*‐CD on *Xac* biofilms. Generally, *Xac* cells were incubated for 48 h with varying concentrations of PyE28@HP‐*β*‐CD, alongside other treatment and control groups. Following incubation, the formed biofilms were stained with crystal violet, and their absorbance was measured at 570 nm. Biofilm inhibition rates were calculated relative to the blank control group, providing a quantitative assessment of the treatments' effects. Meanwhile, the growth curves of *Xac* cells (initial OD_595 nm_ = 0.1) under varying concentrations of PyE28 were determined (Figure S6). At concentrations up to 5.82 µg mL^−1^, no significant difference in OD_595 nm_ was observed between the treatment groups and the blank control, indicating that PyE28 does not inhibit *Xac* growth below this concentration. However, both PyE28 and PyE28@HP‐*β*‐CD treatment groups exhibited a marked reduction in OD_570_ compared to the control group, demonstrating distinct inhibition of biofilm formation (Figure 3A–D). Notably, PyE28@HP‐*β*‐CD achieved a biofilm inhibition rate of 42.82% at 5.82 µg mL^−1^, surpassing the 37.24% observed in the PyE28 treatment group. These findings indicate that PyE28 and PyE28@HP‐*β*‐CD can effectively restrict biofilm formation without impairing the normal growth of *Xac*, highlighting their potential as agricultural chemicals with targeted biofilm‐inhibitory properties. As the concentration of the active ingredient increases, the solution's purple color gradually fades to light purple and ultimately becomes transparent, reflecting a progressive reduction in the pathogen's biofilm content. Notably, at concentrations of 11.64 and 23.28 µg mL^−1^, PyE28@HP‐*β*‐CD achieves biofilm inhibition rates of 74.08% and 93.06%, respectively—significantly outperforming PyE28, which exhibits inhibition rates of 66.72% and 88.29% at the same concentrations. These findings highlight the superior biofilm‐inhibitory potency of PyE28@HP‐*β*‐CD.

To further validate the anti‐*Xac* efficacy of PyE28@HP‐*β*‐CD within the biofilm, single‐colony assays were performed using *Xac* cells from different treatment groups. As shown in Figure 3E and Figure S7, PyE28@HP‐*β*‐CD achieved a colony formation inhibition rate of 67.20% at 11.64 µg mL^−1^, compared to 59.22% for PyE28 alone. This trend aligns closely with the results of the crystal violet staining assay. To visualize these findings more effectively, confocal laser scanning microscopy (CLSM) provided compelling evidence (Figure 3F; Figure S8). In the blank control group, a strong green fluorescence signal indicated dense and viable biofilm matrices formed by *Xac*. As the concentration of PyE28@HP‐*β*‐CD increased, the green fluorescence signal diminished sequentially, and this reduction was consistently more pronounced compared to PyE28 at equivalent concentrations.

In addition, to investigate why the constructed supramolecular system exhibits superior efficiency in inhibiting *Xac* biofilm formation, we employed the phenol‐sulfuric acid method to assess its impact on extracellular polysaccharides (EPS), a critical component of biofilms [35]. Initially, *Xac* cells were incubated with varying concentrations of PyE28@HP‐*β*‐CD, along with other treatment and control groups, for 48 h. After incubation, phenol, sulfuric acid, and *Xac* supernatant were sequentially added, and the absorbance at 490 nm was carefully measured. These measurements were subsequently converted to quantify EPS production and calculate the inhibition rates for each treatment. Analysis of Figure S9A–C revealed distinct trends: in treatments with a lower concentration of PyE28 or PyE28@HP‐*β*‐CD (1.46 µg mL^−1^), the solutions appeared dark brown, indicating a substantial presence of EPS. However, as the concentration increased, the intensity of the brown hue diminished, with the solution transitioning to a light yellow. Remarkably, at a concentration of 5.82 µg mL^−1^, PyE28@HP‐*β*‐CD achieved an EPS inhibition rate of 77.97%, significantly outperforming PyE28, which demonstrated an inhibition rate of only 43.59%. These findings suggest that PyE28@HP‐*β*‐CD acts as a “firewall” during EPS formation, effectively restricting its production and accumulation.

**FIGURE 4:**
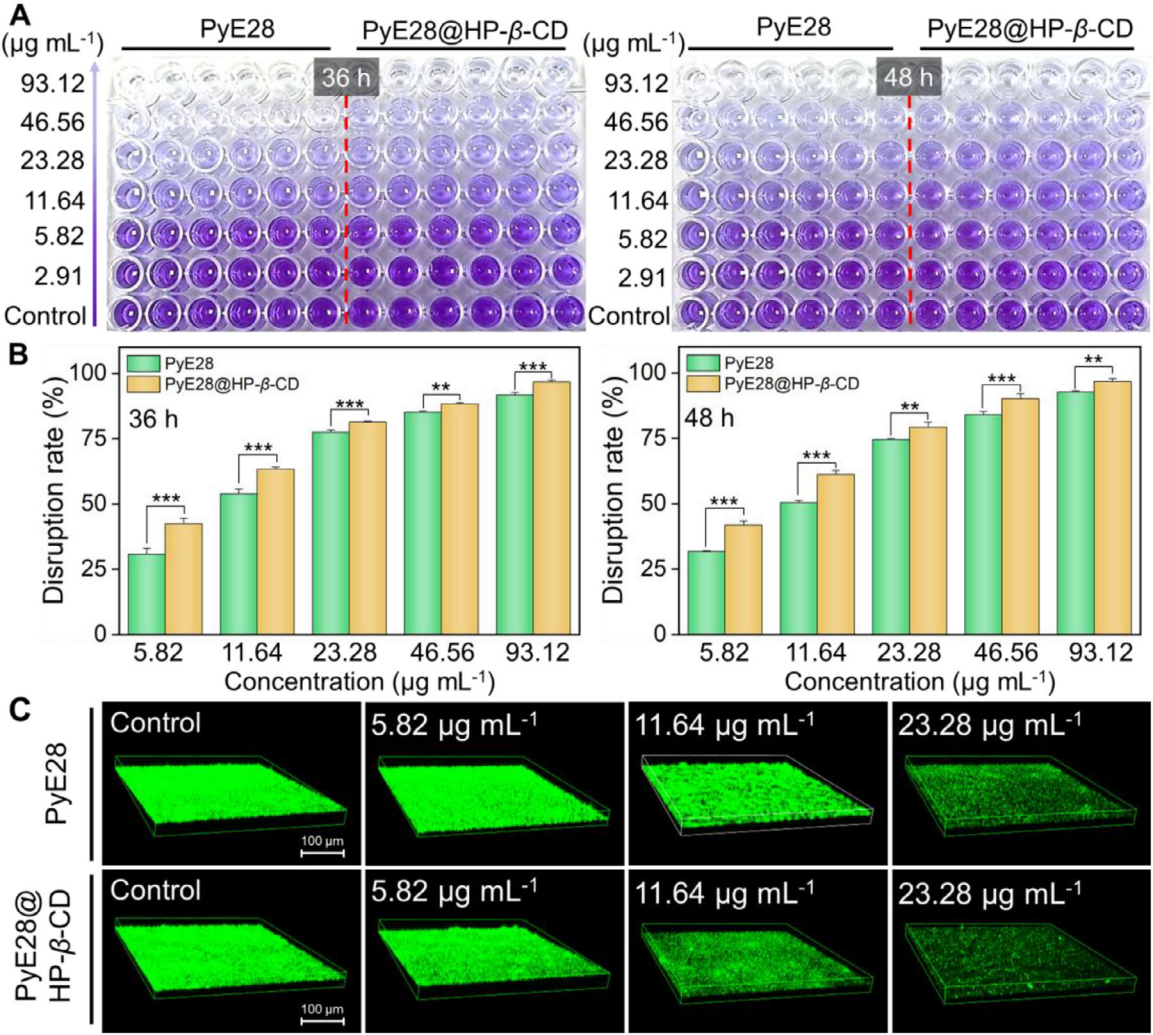
Evaluation of the eradication efficacy of PyE28@HP‐*β*‐CD against pre‐formed *Xac* biofilms. (A) Representative images of crystal violet–stained *Xac* biofilms aged for 36 h (left) and 48 h (right) following treatment with different concentrations (0–100 µg mL^−1^) of active compounds; 0.4% DMSO and HP‐*β*‐CD were used as controls. (B) Quantitative disruption efficiencies of PyE28 and PyE28@HP‐*β*‐CD calculated from OD_570_ nm measurements after 36 and 48 h incubation. (C) CLSM images of pre‐established *Xac* biofilms exposed to 0.05% DMSO, HP‐*β*‐CD, and various concentrations of PyE28 or PyE28@HP‐*β*‐CD (5.82, 11.64, and 23.28 µg mL^−1^) for 36 h, followed by AO staining. Scale bar = 100 µm. Statistical analysis was conducted using an independent‐samples *t*‐test in (B), and significance levels are indicated as *p* < 0.05 (^*^), *p* < 0.01 (^**^), *p* < 0.001 (^***^), and not significant (ns), with *n* ≥ 3 for all experiments.

When bacterial biofilm growth is inhibited, its short‐term harmful effects may be effectively controlled. However, over time, bacteria can re‐emerge, leading to disease recurrence and rendering control efforts futile. Therefore, our objective is not only to inhibit biofilm formation but also to eradicate preformed biofilms, aiming for superior bactericidal efficacy. Although biofilm eradication presents a greater challenge compared to inhibition, we further evaluated the effectiveness of PyE28@HP‐*β*‐CD in disrupting mature *Xac* biofilms. In this study, *Xac* cells were first incubated for 36 or 48 h to generate biofilms at different maturation stages. Subsequently, varying concentrations of these above‐mentioned ingredients were added and co‐incubated for an additional 48 h to disrupt the preformed biofilms. After incubation, crystal violet staining was performed, and the correlative OD_570 nm_ value was measured to assess the biofilm eradication efficiency for each treatment. As shown in Figure 4A, with increasing doses of PyE28@HP‐*β*‐CD, the purple color of the solution gradually faded to near transparency, indicating significant disruption of preformed biofilms. For instance, after 36 h of biofilm incubation, PyE28 and PyE28@HP‐*β*‐CD achieved biofilm eradication rates of 52.90% and 63.50% at 11.64 µg mL^−1^, respectively, demonstrating the superior effectiveness of the supramolecular system in disrupting mature biofilms (Figure 4B). Notably, when the concentration of PyE28@HP‐*β*‐CD was further increased to 23.28 µg mL^−1^, the eradication rate rose significantly to 81.40%. When biofilm incubation was extended to 48 h, the eradication rates of PyE28 and PyE28@HP‐*β*‐CD against biofilms across different concentrations showed trends similar to those observed after 36 h. Meanwhile, CLSM 3D imaging was employed to visualize these results. As shown in Figure 4C and Figure S10, in the control group, *Xac* mature biofilms exhibited intense, dense, and thick green fluorescence, indicating well‐formed biofilm structures. As expected, with increasing concentrations of PyE28@HP‐*β*‐CD, the green fluorescence layer progressively became thinner and sparser. Notably, at a concentration of 23.28 µg mL^−1^, the PyE28@HP‐*β*‐CD treatment group displayed only scattered green fluorescence spots, suggesting that most of the mature biofilm and the bacteria encapsulated within were effectively degraded and eliminated.

Briefly, our supramolecular system targets *Xac* by inhibiting proliferation, preventing biofilm formation, and disrupting preformed biofilms. The enhanced performance is likely due to the synergistic effect of HP‐*β*‐CD, which improves multiple facets including biocompatibility, biofilm penetration, sustained release, and pathogen interaction.

### 2.5 Elucidating the Bactericidal and Anti‐Biofilm Mechanisms of PyE28@HP‐*β*‐CD via Transcriptomic Analysis

**FIGURE 5:**
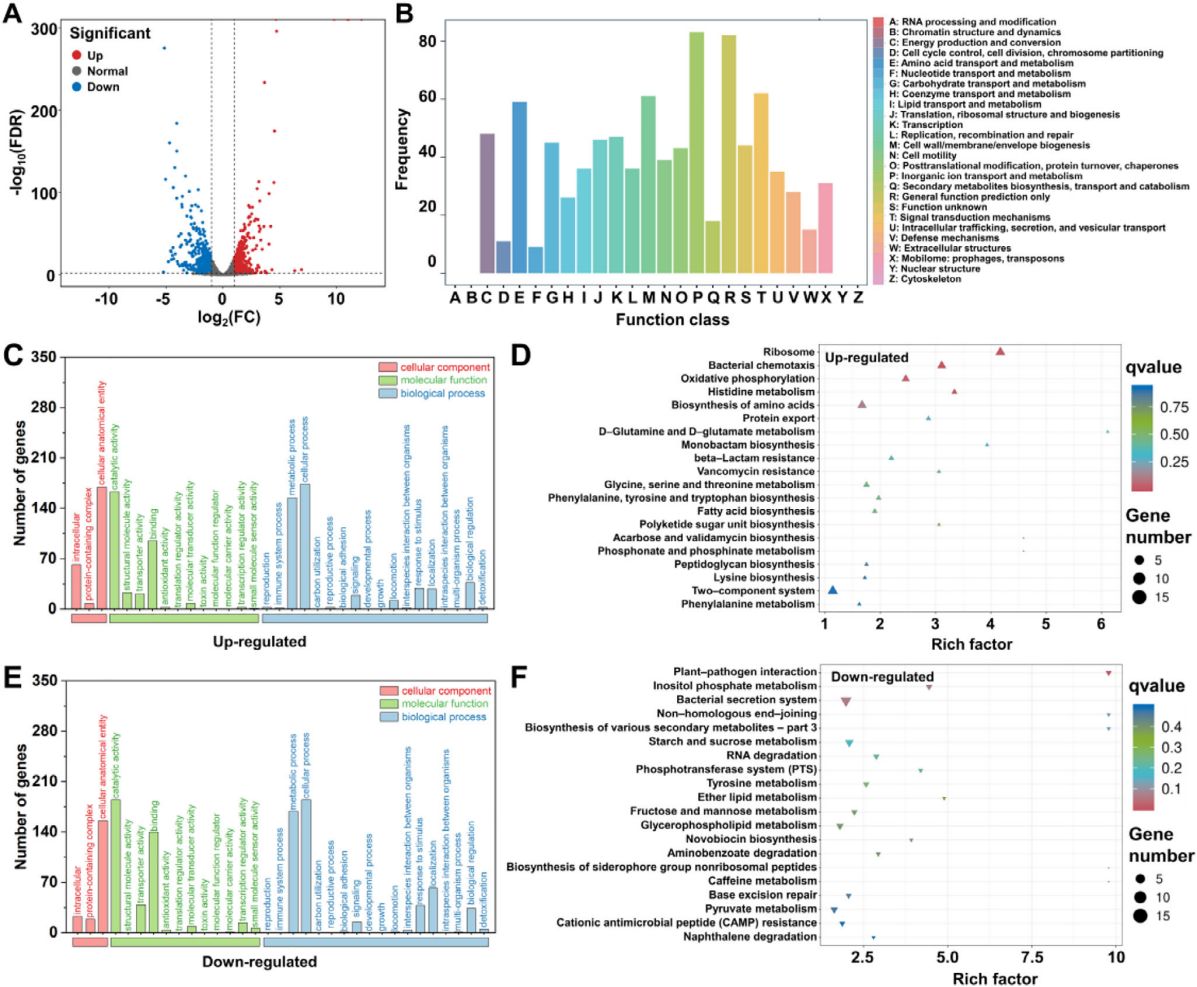
Transcriptomic analysis of bacteria treated with PyE28@HP‐*β*‐CD at 11.64 µg/mL (4 × EC_50_) compared to an untreated control. (A) Volcano plot of differentially expressed genes (DEGs). (B) COG (Clusters of Orthologous Groups) classification of DEGs. (C,D) GO (C) and KEGG (D) enrichment analyses of up‐regulated DEGs. (E,F) GO (E) and KEGG (F) enrichment analyses of down‐regulated DEGs.

To further investigate the antibacterial and anti‐biofilm mechanisms of PyE28@HP‐*β*‐CD, we performed transcriptomic analysis. The sequencing data were of high quality, with read alignment rates of 98.78–99.41% to the reference genome, ensuring reliable functional annotation. Differential expression analysis revealed 502 significantly upregulated and 428 downregulated genes (Figure 5A; Tables S2–S7 and Figure S11), indicating that PyE28@HP‐*β*‐CD elicits a broad transcriptional response.

Functional classification of these differentially expressed genes (DEGs) provided mechanistic insights. COG analysis (Figure 5B) showed that DEGs were primarily enriched in functions related to membrane integrity, metabolic activity, and environmental sensing, including energy production and conversion (C), amino acid transport and metabolism (E), cell wall/membrane/envelope biogenesis (M), transcription (K), replication/recombination/repair (L), and signal transduction mechanisms (T). GO enrichment analysis (Figure 5C,E) indicated that upregulated genes were largely involved in membrane repair, oxidative stress response, and emergency metabolic pathways. In contrast, downregulated genes were predominantly associated with proton transmembrane transport, energy metabolism, oxidative phosphorylation, and biofilm formation. This pattern suggests that PyE28@HP‐*β*‐CD disrupts bacterial homeostasis by suppressing core metabolic processes while simultaneously inducing stress responses. KEGG pathway analysis (Figure 5D,F) further corroborated these findings, with upregulated genes enriched in glutathione metabolism and stress response pathways, and downregulated genes significantly involved in oxidative phosphorylation, ABC transporters, and bacterial secretion systems.

#### 2.5.1 Experimental Validation of the Proposed Mechanisms. Membrane Disruption

To validate the potential bactericidal mechanisms suggested by the transcriptomic data, we first assessed the impact of PyE28@HP‐*β*‐CD on bacterial membrane permeability by measuring electrical conductivity. As shown in Figure S12A,B, the relative conductivity of bacterial solutions increased markedly with higher concentrations of PyE28@HP‐*β*‐CD. After 8 h of incubation at 5.82 and 11.64 µg mL^−1^, conductivity rose from 2.11% in the control group to 38.59% and 62.09%, respectively. In comparison, PyE28 alone resulted in lower conductivity (30.95% and 53.17% at the same concentrations). These results demonstrate that PyE28@HP‐*β*‐CD significantly compromises membrane integrity, leading to substantial leakage of intracellular electrolytes.

#### 2.5.2 Oxidative Stress Induction

Given the transcriptomic evidence of an oxidative stress response, we next evaluated intracellular ROS (Reactive oxygen species) levels. As shown in Figure S12C, ROS accumulation in *Xac* strains increased significantly with higher concentrations of PyE28@HP‐*β*‐CD, exceeding the levels induced by PyE28 alone. Fluorescence intensity analysis at 520 nm provided further evidence that PyE28@HP‐*β*‐CD effectively disrupts redox homeostasis (Figure S12D). For instance, at 11.64 µg mL^−1^, the ROS relative intensity in the PyE28@HP‐*β*‐CD‐treated group reached 6.13 × 10^5^ a. u., significantly higher than the 5.28 × 10^5^ a. u. observed with PyE28.

#### 2.5.3 Impairment of Antioxidant Defense

To uncover the molecular basis for the elevated oxidative stress, we analyzed the activity of key antioxidant enzymes, catalase (CAT) and superoxide dismutase (SOD). The results showed a dose‐dependent suppression of both CAT and SOD activities in the PyE28@HP‐*β*‐CD‐treated group, which was more pronounced than the modest reduction caused by PyE28 alone (Figure S12E,F). This indicates that PyE28@HP‐*β*‐CD effectively disrupts the bacterial antioxidant defense system. We propose that this enhanced inhibition may occur because PyE28@HP‐*β*‐CD can disrupt the 3D structure and function of these antioxidant enzymes, potentially by binding to their active sites. We propose that this enhanced inhibition may stem from the ability of PyE28@HP‐*β*‐CD to either bind directly to the key active sites of these antioxidant enzymes or disrupt their 3D structures, leading to impaired function. Furthermore, its supramolecular structure might interfere with the electron transport chain, thereby exacerbating redox imbalance.

In summary, PyE28@HP‐*β*‐CD exerts its antibacterial and anti‐biofilm effects through a dual mode of action: it efficiently disrupts bacterial membrane integrity, causing electrolyte leakage and interfering with membrane‐associated energy metabolism; simultaneously, it induces severe oxidative stress by elevating intracellular ROS levels and directly suppressing key antioxidant enzymes (SOD and CAT). The transcriptomic profile consistently supports this mechanism, with upregulated genes corresponding to stress and repair responses and downregulated genes linked to core metabolic and biofilm functions. Collectively, these disruptions lead to metabolic collapse, loss of biofilm integrity, and ultimately, irreversible *Xac* cells death.

### 2.6 PyE28@HP‐*β*‐CD Enables the Efficient Control of Citrus Canker in Vivo

**FIGURE 6:**
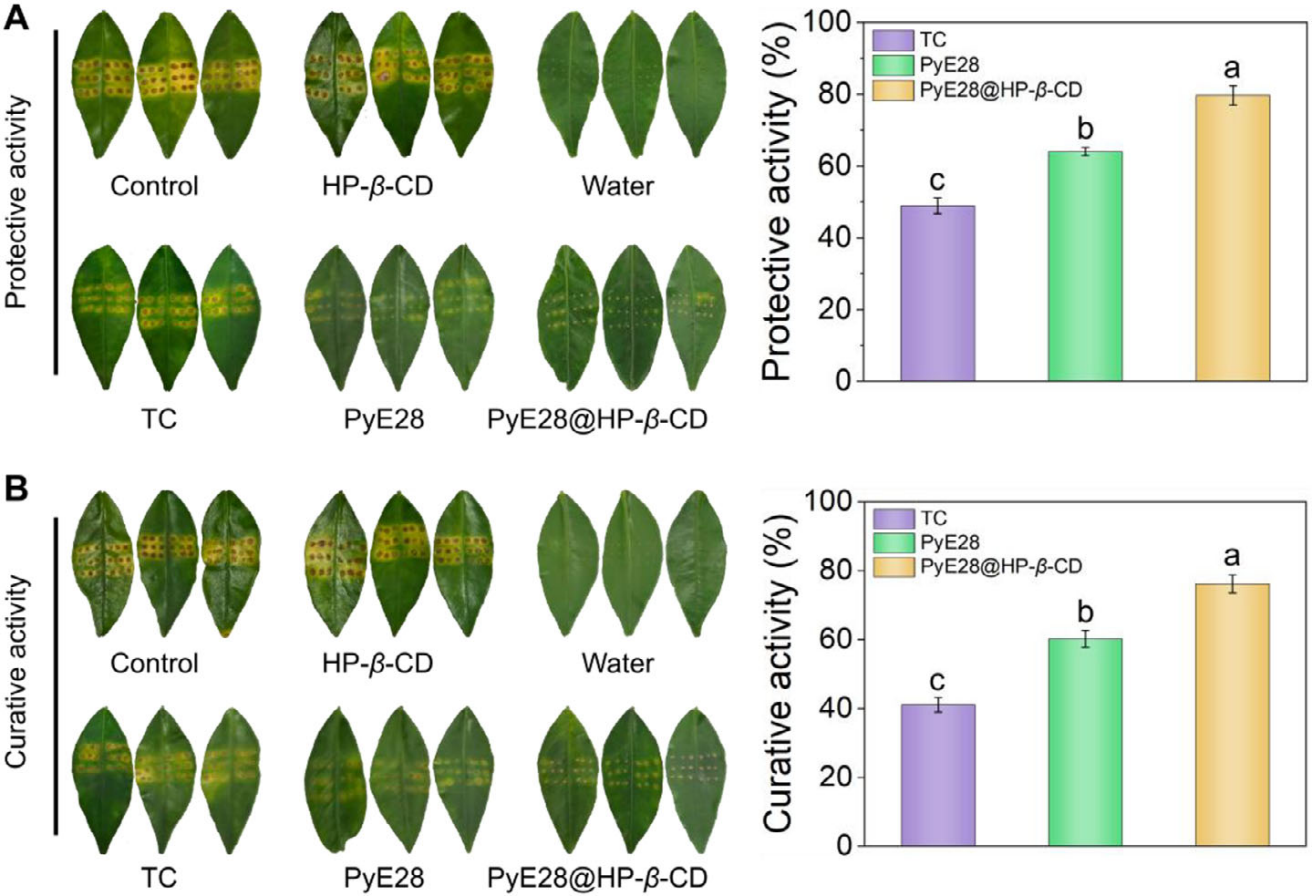
(A) The photographs of protective effects and control efficiencies of PyE28@HP‐*β*‐CD, PyE28, and TC against citrus canker at 200 µg mL^−1^; (B) The photographs of curative effects and control efficiencies of PyE28@HP‐*β*‐CD, PyE28, and TC against citrus canker at 200 µg mL^−1^. Different lowercase letters denote statistically significant differences (*p* < 0.05, *n* ≥ 3) as analyzed by one‐way ANOVA followed by Waller–Duncan post hoc test.

Given its potent anti‐biofilm and antibacterial activity against *Xac*, coupled with its excellent leaf surface deposition, we subsequently evaluated the in vivo control efficacy of PyE28@HP‐*β*‐CD against citrus canker. As shown in Figure 6A,B, citrus leaves in the blank control group developed extensive yellow lesions with halos following *Xac* inoculation. In contrast, the TC‐treated group displayed a reduction in disease severity, with protective and curative efficacies of 48.97% and 41.10%, respectively, confirming the moderate activity of this commercial bactericide. The PyE28‐treated group provided better control, with protective and curative efficacies rising to 64.09% and 60.25%. Most notably, PyE28@HP‐*β*‐CD demonstrated the highest performance, achieving protective and curative efficacies of 79.73% and 76.17%, respectively. These results indicate that the biocompatible sugar‐armored PyE28@HP‐*β*‐CD supramolecular system is a highly promising candidate for effectively managing citrus canker caused by *Xanthomonas*.

### 2.7 Extension to Staple Crop Protection: Superior Deposition on Rice Leaves and Effective Control of Bacterial Blight

Building on its efficacy against citrus canker, we extended the application of PyE28@HP‐*β*‐CD to control rice bacterial leaf blight (BLB), a major threat to staple food production. We first evaluated the in vitro antibacterial activity of all compounds against *Xanthomonas oryzae* pv. *oryzae* (*Xoo*), the causal agent of BLB. PyE28 again demonstrated the highest potency, with an EC_50_ value of 2.03 µg mL^−1^, confirming its broad‐spectrum potential (Table S8).

**FIGURE 7:**
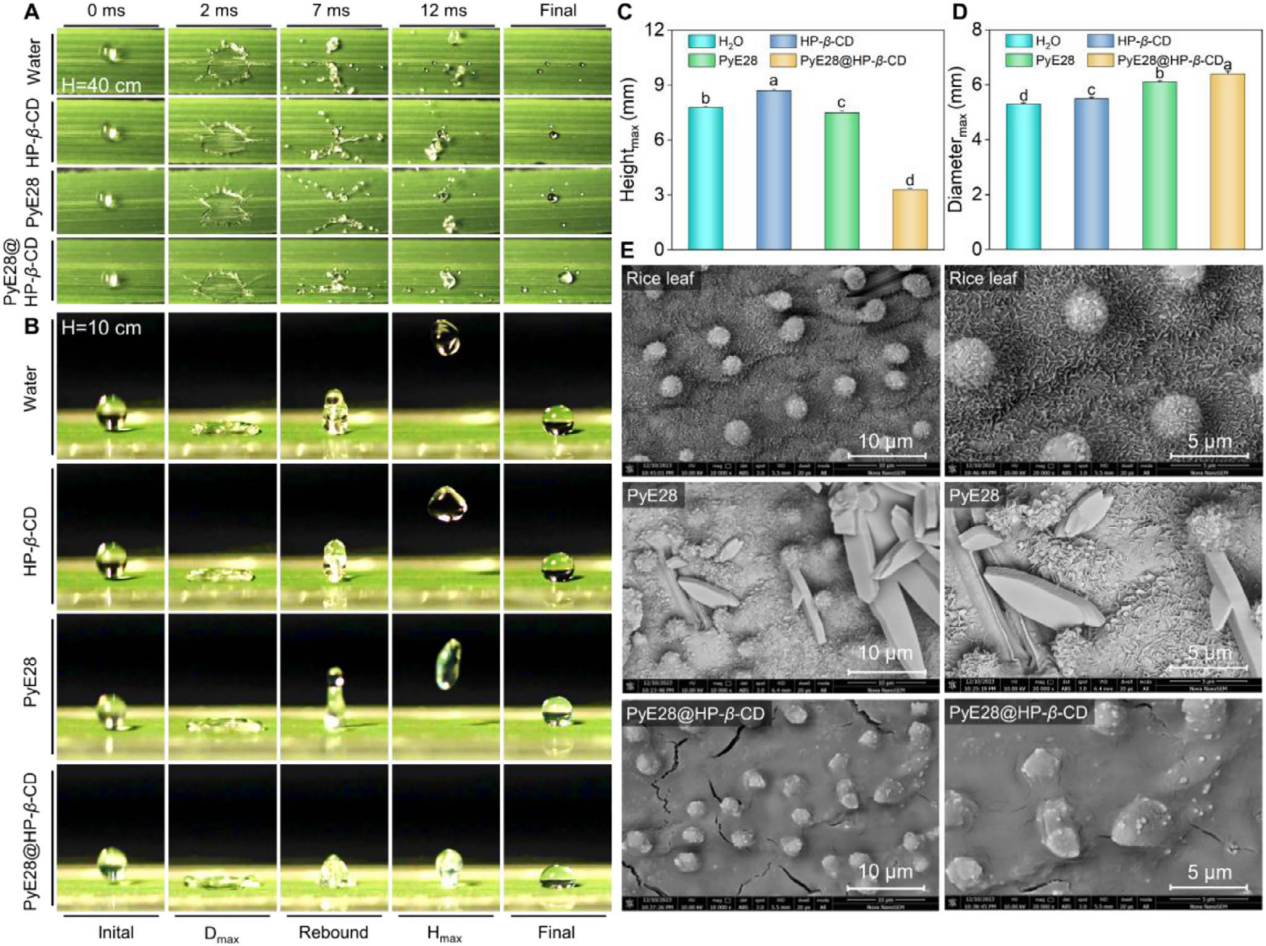
Dynamic behaviors of droplets on rice leaf surfaces. (A) Splashing phenomena of droplets containing water, HP‐*β*‐CD, PyE28, and PyE28@HP‐*β*‐CD (200 µg mL^−1^) released from a height of 40 cm. (B) Bouncing performance of the same droplet systems dropped from a vertical distance of 10 cm. (C,D) Quantitative comparison of the maximum rebound height (H_m__a__x_) and spreading diameter (D_m__a__x_) of the droplets obtained from Video S3. (E) SEM micrographs showing the surface deposition of PyE28 and PyE28@HP‐*β*‐CD on rice leaves at 200 µg mL^−1^. For (C,D), different lowercase letters denote statistically significant differences (*p* < 0.05, *n* ≥ 3) as analyzed by one‐way ANOVA followed by Waller–Duncan post hoc test.

Prior to in vivo trials, we investigated the wetting and deposition performance of PyE28@HP‐*β*‐CD on superhydrophobic rice leaves, which are characterized by microstructures like papillae and protrusions. High‐speed video analysis (Video S2) captured droplet impact dynamics from a height of 40 cm. At 2 ms post‐impact, all droplets reached maximum spreading, converting potential energy to kinetic energy and resulting in splash and volume loss. Notably, PyE28@HP‐*β*‐CD droplets retained most of their volume on the leaf (Figure 7A), suggesting enhanced retention and adhesion. To test the potential of improved viscoelasticity, we reduced the release height to 10 cm to minimize splashing (Figure 7B and Video S3). While the maximum spreading diameter (D_m__a__x_) was similar across all treatments (5.3–6.5 mm; Figure 7D), the maximum bouncing height (H_m__a__x_) of PyE28@HP‐*β*‐CD was only 3.2 mm, significantly lower than the 7.5–8.8 mm observed for other formulations (Figure 7C). Furthermore, PyE28@HP‐*β*‐CD droplets stabilized promptly upon contact with only slight contraction, whereas others underwent multiple rebounds. Subsequently, we further tested the sliding behavior of the supramolecular droplets on rice leaves tilted at 45° (Figure S13 and Video S4). The results demonstrated that, after 200 ms, both the water droplet and PyE28 droplet completely slid off the leaf surface. In contrast, the PyE28@HP‐*β*‐CD droplet only slid a short distance before remaining on the leaf surface, indicating its superior retention on tilted surfaces and highlighting its potential for long‐lasting foliar retention even under challenging conditions.

SEM imaging (Figure 7E) visually confirmed the superior deposition. Untreated leaves showed a waxy surface with abundant papillae. PyE28 droplets formed sparse, spindle‐shaped residues, leaving much of the surface exposed. In contrast, PyE28@HP‐*β*‐CD formed a dense, uniform, and continuous film, promoting better stomatal absorption of the active ingredient. Similarly, to test the supramolecular retention on rice leaves, we conducted LHC tests. The results showed that the supramolecular formulation had a retention of 8.23 mg/cm^2^, significantly higher than PyE28 and other control groups (2.18–3.50 mg/cm^2^), indicating superior retention on the leaf surface (Figure S14). In general, the sugar‐armored PyE28@HP‐*β*‐CD significantly enhances droplet adhesion on superhydrophobic leaves by optimizing viscoelasticity, thereby effectively reducing bounce and splash. This improvement increases pesticide deposition efficiency, promotes crop absorption, and provides valuable technological support for high‐efficiency, low‐loss agrochemical applications.

**FIGURE 8:**
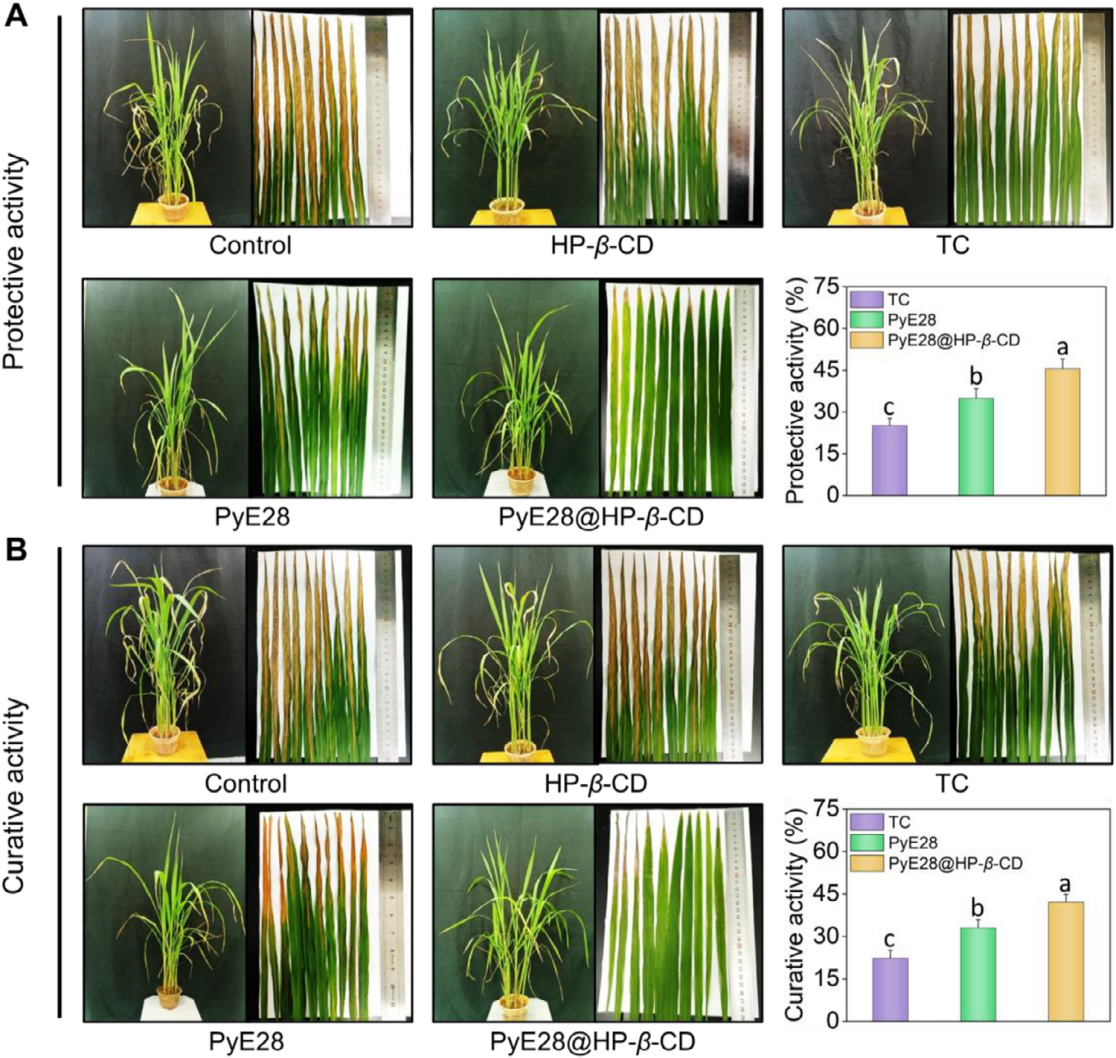
Representative photographs showing the (A) protective and (B) curative activities, as well as the corresponding control efficacies, of PyE28@HP‐*β*‐CD, PyE28, and the technical compound (TC) against rice bacterial blight at 200 µg mL^−1^. Different lowercase letters denote statistically significant differences (*p* < 0.05, *n* ≥3), as determined by one‐way ANOVA followed by the Waller–Duncan post hoc test.

Capitalizing on its enhanced deposition, the in vivo efficacy of PyE28@HP‐*β*‐CD against rice bacterial leaf blight was assessed. Disease symptoms were severe in the control group (Figure 8A,B). While the commercial standard TC showed moderate efficacy (22.31% curative; 25.20% protective), PyE28@HP‐*β*‐CD was substantially more effective, achieving 42.15% curative and 45.53% protective efficacy and significantly outperforming all counterparts. This extended study confirms that the superior in vivo performance of PyE28@HP‐*β*‐CD stems from the synergistic combination of its intrinsic antibacterial activity and its engineered surface deposition capability. This dual advantage establishes it as a promising next‐generation bactericide for practical agricultural application.

### 2.8 Sugar‐Armored PyE28@HP‐*β*‐CD Exhibits Good Biosafety

In the context of sustainable modern agriculture, the environmental friendliness and ecological safety of agrochemicals are of great importance. In this study, the potential toxicity of PyE28@HP‐*β*‐CD on target crops and non‐target organisms was evaluated. First, 200 µg mL^−1^ of PyE28@HP‐*β*‐CD was sprayed on citrus leaves, and no phytotoxic symptoms were observed (Figure S15A). Subsequent chlorophyll measurements revealed no significant differences between the treatment and control groups (Figure S15D). Furthermore, rice seeds treated with 200 µg mL^−1^ of PyE28@HP‐*β*‐CD showed germination rates exceeding 95%, with no significant difference compared to the control group (Figure S15B,E). As the seeds continued to germinate, the shoot and root lengths of rice were measured, and no significant differences were found between treatment groups (Figure S15F,G). Considering that most agrochemicals eventually enter the soil after application, the toxicity of PyE28@HP‐*β*‐CD to non‐target soil organisms was assessed using earthworm acute toxicity tests (Figure S15C,H). Results showed that at a concentration of 10 µg mL^−1^, PyE28@HP‐*β*‐CD did not cause mortality in earthworms after 48 h of exposure, indicating extremely low toxicity. These findings suggest that the constructed oligosaccharide‐coated supramolecular material is a safe, green, and biocompatible bactericidal agent.

## 3 Conclusion

In summary, we successfully developed a multifunctional supramolecular bactericide, PyE28@HP‐*β*‐CD, by incorporating the hydrophobic molecule PyE28 into HP‐*β*‐CD via host‐guest interactions. This strategy effectively overcame the intrinsic hydrophobicity of PyE28, forming stable spherical aggregates with an average size of 0.743–1.741 µm. The resulting system exhibited excellent aqueous stability, improved dispersibility, sustained release profiles, and enhanced biofilm‐penetrating capability, which collectively maximized its bioavailability. At a low dose of 11.64 µg mL^−1^, PyE28@HP‐*β*‐CD achieved a 74.08% inhibition rate against *Xac* biofilms, markedly outperforming PyE28 alone. Mechanistic investigations revealed a multi‐target action: it disrupts biofilms by reducing extracellular polysaccharides and induces bacterial death via oxidative stress from excessive ROS accumulation. Transcriptomic analysis confirmed that it perturbs critical cellular processes, including energy metabolism, membrane integrity, and biofilm‐related pathways. Furthermore, PyE28@HP‐*β*‐CD significantly enhanced droplet wettability and retention on superhydrophobic rice leaves. This superior formulation property translated into outstanding in vivo efficacy, achieving 79.73% control of citrus canker and 45.53% control of rice bacterial blight at 200 µg mL^−1^, surpassing a commercial TC‐SC formulation while maintaining excellent biosafety. This work underscores the potential of supramolecular strategies to transform hydrophobic active ingredients into safer, more effective, and mechanistically well‐defined agrochemicals.

## 4 Experimental Section

### 4.1 Reagents

The solvents and reagents used in the experiments, including hydroxypropyl‐*β*‐cyclodextrin (HP‐*β*‐CD) dichloromethane, methanol, ethyl acetate, petroleum ether, potassium carbonate, ammonium chloride, sodium chloride, sodium hydroxide, hydrochloric acid, sulfuric acid, acetone, 2,3‐dichloropyridine, *p*‐hydroxyphenol, epichlorohydrin, piperidines, piperazines, morpholine, benzylamine with different substitutions, and other nitrogen‐containing heterocyclic compounds, were of analytical grade with purity ≥ 98%. All solvents and reagents were purchased from commercial sources. CAT Content Assay Kit (BC0205, Beijing Solarbio Science & Technology Co., Ltd.).

### 4.2 Instruments

The hydrogen spectrum and carbon spectral data were detected by NMR spectrometer (JEOL‐ECX‐500 (Nippon Electronics Corporation, Musashino, Akishima, Tokyo, Japan) and Bruker Biospin‐AG‐400 (Bruker Spectroscopy Instruments, Ettlingen, Germany)) using DMSO‐*d*_6_ and CDCl_3_ as deuterated solvents and TMS as internal standard solution. Corresponding mass spectral data were scanned and approved by a high‐resolution mass spectrometer (uitu3000, Thermo Scientific, Thermo Fisher Scientific (China) Co., Ltd., Shanghai, China). The OD value of in vitro antibacterial activity was monitored by using Cytation 5 multimode readers (BioTek Instruments, Inc., Vermont, USA). The in vivo bacterial inhibition experiment was carried out in an intelligent artificial climate chamber (RXZ‐436C, Ningbo Jiangnan Instrument Factory, Ningbo, Zhejiang, China). Ultraviolet‐visible (UV–vis) spectra were measured with a UV‐1900 spectrophotometer (Shimadzu Corporation, Sakyo‐ku, Kyoto, Japan). Contact angle experimenting was performed using a JC‐2000D1 instrument (Shanghai Zhongchen Digital Technology Instrument Co., Ltd., Shanghai, China). A highly sensitive DLS analyzer (NanoBrook 90Plus PALS) was used to determine the molecular Zeta potential (Brookhaven Instruments, New York, NY, USA). The droplet bouncing and impact experiments were captured by an i‐SPEED 220 high‐speed camera (Optronis Germany, Kehl, Germany). Compounds and rice samples were visualized and image processed using a FEI Nova NanoSEM 450 scanning electron microscope (SEM) (FEI USA, Hillsboro, Oregon, USA). Biofilm inhibition and eradication samples were processed by 3D visualization photography using a Nikon A1R Confocal Microscope System (Nikon Instruments Inc., Melville, NY, USA). High Performance Liquid Chromatography (HPLC) analysis was carried out with Wooking K2025 HPLC system.

### 4.3 Statistical Analysis

All experiments were conducted with at least three independent replicates. Data are presented as mean ± standard deviation (SD), and the sample size (n) for each experiment is indicated in the corresponding figure legends. Statistical analyses were performed using SPSS 26.0 (IBM, Armonk, NY, USA). For comparisons between two groups, an unpaired two‐tailed Student's t‐test was applied, and significance was denoted as *p* < 0.05 (^*^), *p* < 0.01 (^**^), and *p* < 0.001 (^***^). For comparisons among multiple groups, one‐way analysis of variance (ANOVA) followed by post hoc multiple comparisons was used; different lowercase letters indicate statistically significant differences at *p* <0.05, whereas the same letter indicates no significant difference. Detailed statistical information (test type, n, and significance criteria) is provided in the figure legends.

## Author Contributions

P.W. conceived and supervised the study. J.Y., J.L., and X.W. performed the experiments. J.Y. analyzed and validated the data. J.Y. and J.L. provided technical guidance and valuable assistance throughout the research. J.Y. and J.L. prepared the original draft of the manuscript. P.W. reviewed and revised the manuscript. All authors read and approved the final version of the paper.

## Conflicts of Interest

The authors declare no conflicts of interest.

## Supporting information




**Supporting File 1**: advs74149‐sup‐0001‐SuppMat.docx.

**Supporting File 2**: advs74149‐sup‐0002‐Video S1.mp4.

**Supporting File 3**: advs74149‐sup‐0003‐Video S2.mp4.

**Supporting File 4**: advs74149‐sup‐0004‐Video S3.mp4.

**Supporting File 5**: advs74149‐sup‐0005‐Video S4.mp4.

## Data Availability

The data that support the findings of this study are available in the supplementary material of this article.
