# Supplementary material for: Sugar‐Armored Pesticides: Self‐Assembled System for Enhanced Foliar Adhesion and Sustained Delivery of Hydrophobic Antimicrobials Against Bacterial Diseases
Source: Adv Sci (Weinh). 2026 Jan 28;13(20):e24286. doi: 10.1002/advs.202524286 (PMC13067871; doi:10.1002/advs.202524286)
Supplement: Supplementary file 1 — Supporting File 1: advs74149‐sup‐0001‐SuppMat.docx. [file ADVS-13-e24286-s001.docx]

**Supplementary Information**

**Sugar-Armored Pesticides: Self-Assembled System for Enhanced Foliar Adhesion and Sustained Delivery of Hydrophobic Antimicrobials against Bacterial Diseases**

*Jinghan Yang, Juan Liu, Xiaohui Wang, Peiyi Wang**

State Key Laboratory of Green Pesticide, Key Laboratory of Green Pesticide and Agricultural Bioengineering, Ministry of Education, Center for Research and Development of Fine Chemicals of Guizhou University, Guiyang, 550025, China.

*Corresponding author E-mail: pywang@gzu.edu.cn; pywang888@126.com (P.-Y. Wang).

**Contents**

[1. Experimental Section 4](#_Toc219223643)

[1.1 *In Vitro* Antibacterial Evaluation 4](#_Toc219223644)

[1.2 UV-vis Titration Experiments 4](#_Toc219223645)

[1.3 Job's Curve Test 5](#_Toc219223646)

[1.4 Zeta Potential Testing 5](#_Toc219223647)

[1.5 Particle Size Analysis 5](#_Toc219223648)

[1.6 ^1^H NMR Titration Experiment 5](#_Toc219223649)

[1.7 Scanning Electron Microscope Experiments (SEM) 6](#_Toc219223650)

[1.8 Release Behavior of PyE28 and PyE28@HP-*β*-CD 6](#_Toc219223651)

[1.9 Tyndall Effect Experiment for PyE28@HP-*β*-CD 7](#_Toc219223652)

[1.10 Contact Angle, Surface Tension, Rebound, Splash and Spray Assays 7](#_Toc219223653)

[1.11 Anti-rain Wash-off Resistance Experiment 7](#_Toc219223654)

[1.12 Liquid-Holding Capacity (LHC) Test 8](#_Toc219223655)

[1.13 Leaf Surface Sliding Test 8](#_Toc219223656)

[1.14 Biofilm Inhibition and Eradication Experiments 8](#_Toc219223657)

[1.15 Confocal Laser Scanning Microscopy (CLSM) 3D Imaging 9](#_Toc219223658)

[1.16 *Xac* Colonies on Agar Plates for Biofilm-Enclosed Bacteria 9](#_Toc219223659)

[1.17 Quantitative Determination of Exopolysaccharide (EPS) 10](#_Toc219223660)

[1.18 Measurement of the Relative Electrical Conductivity 10](#_Toc219223661)

[1.19 ROS Analysis 10](#_Toc219223662)

[1.20 CAT and SOD Enzyme Activity Analysis 11](#_Toc219223663)

[1.21 *In Vivo* Antibacterial Against Citrus Canker 11](#_Toc219223664)

[1.22 *In Vivo* Efficacy Assessment Against Rice Bacterial Leaf Blight 12](#_Toc219223665)

[2. Synthesis of Target Compounds and Characterization Data 13](#_Toc219223667)

[3. Supplementary Tables and Figures 28](#_Toc219223668)

[3.1 Results of ^1^H NMR Titration Experiments of PyE28 28](#_Toc219223669)

[3.2 Sequencing Data and Alignment Results to the Reference Genome 28](#_Toc219223670)

[3.3 *In Vitro* Antibacterial Activity of Compounds against *Xoo* 33](#_Toc219223671)

[3.4 HRMS Spectrum of the Supramolecular Complex PyE28@HP-*β*-CD 34](#_Toc219223672)

[3.5 Assembly Mechanisms of PyE28 34](#_Toc219223673)

[3.6 HPLC Calibration Curve of PyE28 34](#_Toc219223674)

[3.7 Stability of PyE28@HP-*β*-CD 35](#_Toc219223675)

[3.8 Rain Wash-Off Resistance of PyE28@HP-*β*-CD on Rice Leaves 35](#_Toc219223676)

[3.9 Growth Curve of *Xac* 36](#_Toc219223677)

[3.10 Analysis of Biofilm Inhibition Rate Using the Agar Diffusion Method 36](#_Toc219223678)

[3.11 Analysis of Biofilm Inhibition Rate Using the CLSM 37](#_Toc219223679)

[3.12 Impact of PyE28@HP-*β*-CD on Extracellular Polysaccharide Production 37](#_Toc219223680)

[3.13 Analysis of Biofilm Eradication Rate Using the CLSM 38](#_Toc219223681)

[3.14 Differentially Expressed Genes (DEGs) Analysis 38](#_Toc219223682)

[3.15 The Possible Bactericidal Mechanisms 39](#_Toc219223683)

[3.16 Drop Behavior on Inclined Rice Leaf with PyE28@HP-*β*-CD 40](#_Toc219223684)

[3.17 Liquid-Holding Capacity of the Formulations on Rice Leaves 40](#_Toc219223685)

[3.18 Biosafety Evaluation 41](#_Toc219223686)

[3.19 ^1^H NMR, ^13^C NMR, ^19^F NMR and HRMS Spectra of Target Compounds 42](#_Toc219223687)

# 1. Experimental Section

## 1.1 *In Vitro* Antibacterial Evaluation

The *in vitro* turbidimetric method was used to evaluate the antimicrobial activity of compounds PyE1-PyE33 against phytopathogenic strains, *Xac* and *Xoo*. Compounds were added to 4.0 mL NB liquid medium (1.5 g beef extract, 2.5 g peptone, 0.5 g yeast powder, 5.0 g glucose, and 500 mL distilled water; pH=7.2). Starting from 100 *μ*g mL^-1^, the concentrations of compounds were stepwise reduced by 2-times. Finally, 40 *μ*L of bacterial suspension (OD_595_=0.6-0.8) were added to each tube. The inoculated test tubes were incubated at 28 ± 1 °C and continuously shaken at 180 rpm for 24−48 h until bacteria were incubated on the logarithmic growth phase. After that, 200 *μ*L samples were transferred to 96-well plates, the OD_595_ of the solution was measured with an enzyme calibration. Dimethyl sulfoxide was used as a blank control and thiodiazole-copper (TC) was used as a positive control.

The OD_595_ value is calculated according to the following formula:

OD_595 (turbidity-corrected values)_ = OD_bacterial wilt_ − OD_no bacterial wilt_

And the inhibition rate I is calculated according to the following formula:

$$\text{I (\%) = (C - T) ∕ C × 100}$$

where C is the corrected turbidity values of bacterial growth on untreated NB (blank control), and T is the corrected turbidity values of bacterial growth on treated NB. Compounds concentration was converted to log (x), and inhibition rate data was converted to odds (y). The toxicity regression equation ($y=kx+b$) and correlation coefficient (R^2^) were obtained using excel calculations to determine the median effective concentration value (EC_50_).

## 1.2 UV-vis Titration Experiments

UV-vis spectra were tested by preparing a 3.0 mL aqueous solution of 0.16 mM PyE28 and aqueous solutions of HP-*β*-CD at varying concentrations, with the molar ratios of PyE28 to HP-*β*-CD being 1:0, 1:0.25, 1:0.5, 1:0.75, 1:1, 1:1.25, 1:1.5, 1:1.75, and 1:2, respectively. The Benesi-Hildebrand (B-H) equation was used to determine the binding constants (*K*_a_).

1/*Δ*A=1/a×*K*_a_×c(HP-*β*-CD) + 1/a

Where *Δ*A is the difference between one pair of absorbance values before and after the measurement addition, c is the total concentration of HP-*β*-CD, and a is a constant. *K*_a_ was obtained by plotting 1/*Δ*A against 1/c (HP-*β*-CD) and the slope and intercept of the resulting line were calculated.

## 1.3 Job's Curve Test

A 0.12 mM solution of PyE28 and HP-*β*-CD was prepared to configure a mixed solution with a total volume of 3.0 mL in different ratios. In this process, PyE28 was present in different molar fractions (*Xa*). The difference in UV absorption at each concentration was determined, resulting in a change in ∆*Xa*. A Job's curve was generated by plotting ∆*Xa* versus N_HP-_*_β_*_-CD_/N _PyE28+HP-_*_β_*_-CD_ (molar ratio).

## 1.4 Zeta Potential Testing

PyE28 and PyE28@HP-*β*-CD at a concentration of 200 *μ*g mL^-1^ were tested for zeta potential using a dynamic light scatterometer.

## 1.5 Particle Size Analysis

PyE28 and PyE28@HP-*β*-CD were prepared at a concentration of 200 *μ*g mL^-1^. The corresponding images were captured using a scanning electron microscope (SEM). Particle size analysis was then performed by quantifying the particles in the SEM images using ImageJ software.

## 1.6 ^1^H NMR Titration Experiment

PyE28 (8.05 mg) and HP-*β*-CD (11.34 mg) were dissolved in 500 *μ*L DMSO and 1.0 mL D_2_O, respectively. PyE28 and HP-*β*-CD masterbatches with concentrations of 50 mM and 10 mM were obtained. Then, solutions containing 5.0 mM of PyE28, HP-*β*-CD, and their inclusion complexes were prepared in varying molar ratios (1:0, 1:1, and 1:1.5, respectively). Finally, all solutions were measured by nuclear magnetic resonance instrument.

## 1.7 Scanning Electron Microscope Experiments (SEM)

PyE28 and PyE28@HP-*β*-CD were prepared at a concentration of 200 *μ*g mL^-1^ and allowed to dry naturally on a silicon wafer for microstructural characterization under a scanning electron microscope (SEM). To investigate the deposition effects on leaf surfaces, the samples were applied to the surfaces of citrus leaves and rice leaves before imaging under the SEM.

## 1.8 Release Behavior of PyE28 and PyE28@HP-*β*-CD

The release behaviors of PyE28 and PyE28@HP-*β*-CD were evaluated through dialysis membranes with molecular weight cut-offs of 1 kDa and 3.5 kDa. The release medium was prepared by mixing 70% methanol with 30% water. PyE28 and PyE28@HP-*β*-CD samples were placed in dialysis bags. The dialysis bags were maintained at room temperature (approximately 25 °C) and stirred at 100 rpm to ensure proper mixing. At specified time intervals, 1.0 mL aliquots were withdrawn from the release medium and replaced with fresh medium. The withdrawn samples were analyzed by high-performance liquid chromatography (HPLC). The HPLC conditions used for analysis were as follows: Eclipse XDB-C_18_ column (150 mm × 4.6 mm, 5 *μ*m), mobile phase consisting of 45% water (0.1% formic acid) and 55% acetonitrile, a flow rate of 1.0 mL/min, and detection at 223 nm. The injection volume was 5 *μ*L, and the column was maintained at room temperature.

## 1.9 Tyndall Effect Experiment for PyE28@HP-*β*-CD

In this experiment, a 200 *μ*g mL^-1^ solution of PyE28@HP-*β*-CD was prepared in water. A red laser pointer (650 nm) was directed through the solution, and the scattering of light was observed at a 90° angle.

## 1.10 Contact Angle, Surface Tension, Rebound, Splash and Spray Assays

First, aqueous solutions of PyE28 and PyE28@HP-*β*-CD were prepared at a concentration of 200 *μ*g mL^-1^. After that, the slip, bounce, and splash behaviors of compounds and complexes on citrus and rice leaves were captured using a high-speed camera and quantitatively analyzed with the i-SPEED software. Subsequently, the contact angle and surface tension of these agents on the blade was determined by the goniometric method and hanging drop method, respectively. Finally, the solutions were evenly sprayed on rice leaves, and photos were taken to observe droplet dispersion and size. The ddH_2_O and HP-*β*-CD solutions were used as controls throughout the assays.

## 1.11 Anti-rain Wash-off Resistance Experiment

Optical images of droplet distribution before and after wash-off. Droplets (10 *μ*L) of PyE28 and PyE28@HP-*β*-CD (200 μg mL^-1^), mixed with 0.1% Rhodamine 6G, were deposited onto rice leaf surfaces. For the simulated rain wash-off, the leaf was tilted at 45° and rinsed with deionized water (1.0 mL per rinse) delivered by a 1.0 mL pipette, directly impacting the droplet area; the rinsing was repeated three times. Optical images were recorded under natural light and under UV illumination before and after wash-off to visualize droplet distribution and retention on the leaf surface. For SEM analysis, the rice leaf surfaces before and after the above wash-off procedure were collected, dried, and sputter-coated with gold prior to imaging. SEM images were then acquired to examine surface deposition/retention and wash-off–induced changes in the distribution of PyE28 and PyE28@HP-*β*-CD.

## 1.12 Liquid-Holding Capacity (LHC) Test

For both rice and citrus leaves, a 1 cm^2^ section of leaf was first weighed to obtain the initial weight. The leaf was then immersed in a 200 *μ*g mL^-1^ solution of PyE28 or PyE28@HP-*β*-CD for 10 s. After immersion, the leaf was removed and allowed to dry until no droplets remained on the surface. The final weight of the leaf was then measured. The LHC was calculated by subtracting the initial weight from the final weight.

## 1.13 Leaf Surface Sliding Test

A solution of PyE28 or PyE28@HP-*β*-CD was applied to the surface of rice or citrus leaves. After treatment, a droplet was released from a height of 3.0 cm onto the leaf surface. The leaf was then tilted to 45° (rice) or 60° (citrus) relative to the horizontal plane, and droplet sliding was recorded using a high-speed camera.

## 1.14 Biofilm Inhibition and Eradication Experiments

Inhibition of biofilm: *Xac* cells in NB liquid medium, with an OD_595_ of 0.6-0.8, were incubated overnight and then adjusted to an OD of 0.1. Subsequently, 200 *μ*L of the bacterial suspension was added to each well of a 96-well plate. Different concentrations (0-23.28 *µ*g mL^-1^) of PyE28, PyE28@HP-*β*-CD, and HP-*β*-CD were added using semi-dilution, with six replicates per treatment group. The cultures were incubated stationary at 28 °C for 72 hours. After incubation, the suspended *Xac* cells were carefully removed and washed twice with sterile water. Then, 200 *μ*L of a 0.1% crystal violet solution was added to each well for 30 minutes. After staining, the excess crystal violet solution was removed, and the wells were rinsed three times with sterile water. The plates were then dried in an oven at 37 °C for 1 hour. The crystal violet was completely dissolved using a 95% ethanol solution. Finally, the biofilm content was determined by measuring the OD at 570 nm using an enzyme marker.

For the eradication assay, two hundred microliters of each bacterial suspension (OD_595 nm_=0.1) were taken into a 96-well plate. Plates were sealed and placed in an incubator (28 ℃) for static incubation 36 and 48 hours. After biofilm formation, the bacterial suspensions were discarded and 200 *μ*L fresh culture medium and different concentrations (0-93.12 *µ*g mL^-1^) of the solution were added into the plate. Plates were sealed and placed in an incubator (28 ℃) for static incubation again 24 hours. staining of bacterial biofilm using 200 *μ*L crystal violet dye (10 mg mL^-1^) for 30 minutes. Then, the density of bacterial biofilm at 570 nm were measured to calculate the biofilm eradication rate. Each experiment was repeated three times and the mean value was calculated.

## 1.15 Confocal Laser Scanning Microscopy (CLSM) 3D Imaging

Conductive glass soaked in ethanol, washed, and dried, were sterilized by autoclaving pot and then placing in well of a 6-well plate. For the biofilm inhibition experiment, the bacterial solution (OD_595_= 0.1) was incubated with PyE28 and PyE28@HP-*β*-CD (0-23.28 *µ*g mL^-1^) for 72 h at 28˚C. Then, discard the medium and wash the wells with PBS three times to remove planktonic cells. Finally, biofilm was stained with Acridine Orange (0.1% AO) at room temperature.

For the biofilm eradication experiment, *Xac* cells (OD_595_= 0.1) were richly cultured for 48 h at 28˚C to yield adequate mature biofilms, which were then co-incubated with PyE28 and PyE28@HP-*β*-CD (0-23.28 *μ*g mL^-1^) for another 72 h at 28˚C. Then, discard the medium and wash the wells with PBS three times to remove planktonic cells. Biofilm was stained with Acridine Orange (0.1% AO) for 15 min at room temperature. The samples were then examined by CLSM using a 488 nm laser.

## 1.16 *Xac* Colonies on Agar Plates for Biofilm-Enclosed Bacteria

First, *Xac* cells were harvested by centrifugation (6500 rpm, 2 min) and then washed twice with phosphate-buffered saline (PBS, 10 mM, pH=7.2). The supernatant was discarded, and the remaining *Xac* cells were resuspended in PBS and diluted to an optical density (OD_595_ = 0.1). The experimental and control groups were incubated at 28°C for 9 hours and then diluted 1 × 10^4^ folds with PBS, respectively. Then, 10 μL of

## 1.17 Quantitative Determination of Exopolysaccharide (EPS)

*Xac* cell suspension was cultured with NB culture containing different concentrations (1.46, 2.91, 5.82, 11.64 *µ*g mL^-1^) of PyE28, PyE28@HP-*β*-CD and HP-*β*-CD. This culture was maintained at 28 °C and 180 rpm on a shaker for 2 days. After incubation, the supernatant was obtained by centrifugation, and the supernatant was taken (0.5 mL) and added to (50 g L^-1^) phenol solution (0.5 mL) and 95% H_2_SO_4_ (2.5 mL), respectively, and then, the values of OD_490_ were determined, respectively.

## 1.18 Measurement of the Relative Electrical Conductivity

In brief, *Xac* cells in NB solution (OD_595_ = 0.6−0.8) were collected and washed with 5% glucose until the conductivity was close to that of the 5% glucose solution, which was used as the bacterial isotonic solution. Furthermore, the bacterial isotonic solution was boiled for 5 min, and the conductivity was marked as L0. Meanwhile, diﬀerent doses (0.00, 2.91, 5.82, 11.64 and 23.28 *μ*g mL^-1^) of compound PyE28 and PyE28@HP-*β*-CD were added to the 5% glucose solution, and the conductivity was marked as L1. In addition, diﬀerent doses (0.00, 2.91, 5.82, 11.64 and 23.28 *μ*g mL^-1^) of compound PyE28 and PyE28@HP-*β*-CD were added to the bacterial isotonic solution and cultured in a shaker (180 rpm, 28 °C, 8 h). The conductivity was detected every hour and marked as L2. Finally, the ratio of (L2 − L1)/L0 indicated the relative electric conductivity.

## 1.19 ROS Analysis

ROS levels were determined using a Reactive Oxygen Species Assay Kit purchased from Beijing Solarbio Science & Technology Co., Ltd., following the manufacturer’s instructions. *Xac* cells were cultured to logarithmic growth phase (OD_595_= 0.6-0.8), and the bacterial solution was diluted with NB liquid medium to OD_595_ = 0.1. Then *Xac* cells was co-incubated with different concentrations of the PyE28 and PyE28@HP-*β*-CD (the control group was *β*-CD and 0.01% DMSO solution) at 28 °C and 220 rpm for 12 h. The *Xac* cells were collected by centrifugation (10000 g, 4.0 min, 4 °C), washed twice with sterile water, and the bacterial cells were resuspended in sterile water. After 100 *μ*L of bacteria and 1.0 *μ*L of DCFH-DA staining solution (10 mM) were fully mixed under dark conditions, and Fluoromax-4cp fluorescence spectrophotometer was used to measure fluorescence absorption (excitation wavelength: 488 nm). ROS Content Assay Kit (CA1410) were all obtained from Beijing Solarbio Science & Technology Co., Ltd.

## 1.20 CAT and SOD Enzyme Activity Analysis

*Xac* cells were collected and washed with pre-cooled PBS buffer solution as described in Section 1.15. After the extract provided by the kit was added to the obtained pathogens, ultrasonic crushing (power 30%, ultrasonic 3 s, interval 10 s, repeated 60 times at 0 °C). After centrifugation at 10000 g for 10 min at 4 °C, the supernatant was taken to determine the activity of catalase (CAT) and superoxide dismutase (SOD) according to the kit instructions. CAT content assay kit (BC0205) and SOD content assay kit (BC0175) were all obtained from Beijing Solarbio Science & Technology Co., Ltd. Protein concentration was determined by the Bradford method.

## 1.21 *In Vivo* Antibacterial against Citrus Canker

PyE28, PyE28@HP-*β*-CD and commercially available thiodiazole copper (20% TC SC) were prepared with aqueous solution with a concentration of 200 *μ*g mL^-1^. Firstly, the front of the citrus leaves is evenly punctured with 12 sterile needles. Then, the medicinal liquid is applied to the filter paper on either the first day (for protective activity) or the third day (for curative activity), and the filter paper is left to dry naturally. After 24 h, the *Xac* solution with an OD_595 nm_ range of 0.6-0.8 was centrifuged and adjusted to OD_595_=0.01 with water. The bacterial solution was then applied directly to the wound after wetting the filter paper. An equal amount of DMSO was used as a control without any added agents. Each treatment was examined for onset after 14 d and had more than 6 replicates. The leaves were cut uniformly at the site of injury. Each fraction weighing 100 mg was immersed in a 10 mL mixture of 95% acetone and 95% ethanol in a 1:1 ratio. The absorbance values of OD_663 nm_ and OD_645 nm_ were measured after 24 h, respectively, and the formulas were calculated as follows:

$$\text{Chlorophyll a = (12.7×}\text{OD}_{\text{663 nm}} \text{- 2.69×}\text{OD}_{\text{645}}\text{)×0.1}$$

$$\text{Chlorophyll b = (22.9×}\text{OD}_{\text{645 nm }}\text{- 4.68×}\text{OD}_{\text{663}}\text{)×0.1}$$

$$\text{Total chlorophyll=(8.02×}\text{OD}_{\text{663 nm}}\text{+20.21×}\text{OD}_{\text{645 nm}}\text{)×0.1}$$

$$\text{Infected area=Total chlorophyll of water-Total chlorophyll of component}$$

$$\text{Control efficiency I }\left( \text{\%} \right)\text{=}\frac{\text{C-T}}{\text{C}}\text{×100}$$

Where C is the infected area of the negative control and T is the treatment group.

## 1.22 *In Vivo* Efficacy Assessment against Rice Bacterial Leaf Blight

The efficacy of agents against rice bacterial leaf blight, caused by *Xoo* infection, was evaluated using the leaf-cutting method. Rice leaves were uniformly sprayed with 200 *μ*g mL^-1^ solutions of PyE28, PyE28@HP-*β*-CD, and 20% TC SC in a water formulation. After 24 h, the tips of the leaves were trimmed 1-2 cm using sterilized scissors, and the cut ends were subsequently dipped into a suspension of *Xoo* cells (OD_595 nm_ = 0.8). The treated plants were then incubated for 14 days in a controlled artificial climate at 28 °C and 90% relative humidity Disease index and control efficacy were calculated by disease area. Additionally, the disease index for each treatment (C or T) was calculated using the designated formula:

$$\text{Disease index (C or T)=}\frac{\sum\text{(Number of leaves at each grade×corresponding grade)}}{\text{Total number of leaves×superlative grade}}$$

The control efficiency I for the anti-*Xoo* activity were calculated by the following equation:

$$\text{Control efficiency I }\left( \text{\%} \right)\text{=}\frac{\text{C-T}}{\text{C}}\text{×100}$$

In the equation, C represents the disease index of the negative control, while T denotes that of the treatment group.

# 2. Synthesis of Target Compounds and Characterization Data

**Preparation of Intermediate 1.**

2,3-Dichloropyridine (3.4 mmol, 503.1 mg), hydroquinone (4.05 mmol, 380.6 mg), K_2_CO_3_ (8.1 mmol, 1119.5 mg), and 10 mL DMSO were added to a 15 mL reaction flask and heated at 100°C for 5 hours. After the reaction was complete, the mixture was cooled in an ice bath, and the pH was adjusted to 7. The solid precipitate was allowed to settle, then filtered to yield a white solid (Intermediate 1) with a 58.43% yield.

**Preparation of Intermediate 2.**

Intermediate 1, 4-((3-chloropyridin-2-yl)oxy)phenol (2.26 mmol, 458.0 mg), epichlorohydrin (2.93 mmol, 269.7 mg), KOH (2.71 mmol, 151.5 mg), and 8 mL DMF were added to a 25 mL round-bottom flask and stirred at room temperature for 8 hours. After the reaction was complete, the mixture was extracted with ethyl acetate, washed with saturated NH_4_Cl aqueous solution, and the solvent was removed. The product (Intermediate 2) was purified by column chromatography to yield a white solid with a 41.2% yield.

**Preparation of target compounds (PyE1-PyE33).**

In a 15 mL pressure tube, 3-chloro-2-(4-(epoxy-2-methoxy)phenoxy)pyridine (1.0804 mmol), various types of amine molecules (3.24 mmol), K_2_CO_3_ (0.54 mmol), and 8.0 mL isopropanol (IPA) solvent were added and heated at 60 ℃. After about 36 hours, thin-layer chromatography (TLC) was used to monitor the reaction. The mixture was then extracted with dichloromethane (3-4 times), dried over anhydrous sodium sulfate, and the solvent was removed under reduced pressure at 40 ℃. The 33 target molecules were purified by column chromatography in yields ranging from 17.8% to 95.6% using a dichloromethane/methanol gradient (100/1-50/1, v/v) as eluent.

***N*-(3-(4-((3-chloropyridin-2-yl)oxy)phenoxy)-2-hydroxypropyl)cyanamide (PyE1)**

Yellow solid, yield 45.5%; ^1^H NMR (500 MHz, CDCl_3_ ) *δ* 8.00 (dd, *J* = 4.9, 1.7 Hz, 1H, pyridine-4-H), 7.74 (dd, *J* = 7.7, 1.7 Hz, 1H, pyridine-6-H), 7.11-7.06 (m, 2H, diphenyl-2,6-H), 6.97-6.91 (m, 3H, diphenyl-3,5-H & pyridine-5-H), 4.96-4.84 (m, 1H, CH-OH), 4.11-4.01 (m, 2H, OCH_2_), 3.93 (dd, *J* = 12.3, 9.3 Hz, 1H, NH-CH_2_-CH), 2.65-3.59 (m, 1H, NH-CH_2_-CH). ^13^C NMR (126 MHz, CDCl_3_) *δ* 160.4, 159.5, 155.7, 147.4, 145.2, 139.3, 122.8, 119.1, 118.9, 115.6, 78.5, 69.7, 54.8. HRMS (ESI) [M+H^+^] calcd for C_15_H_15_ClN_3_O_3_: 320.0802, found: 320.0806.

**1-(4-((3-chloropyridin-2-yl)oxy)phenoxy)-3-(ethylamino)propan-2-ol (PyE2)**

Yellowish oily substance, yield 34.4%; ^1^H NMR (500 MHz, CDCl_3_ ) *δ* 8.00 (d, *J* = 3.1 Hz, 1H, pyridine-4-H), 7.73 (d, *J* = 7.4 Hz, 1H, pyridine-6-H), 7.08 (d, *J* = 8.9 Hz, 2H, diphenyl-2,6-H), 6.93 (dd , *J* = 14.9, 6.7 Hz, 3H, diphenyl-3,5-H & pyridine-5-H), 4.09 (d, *J* = 4.2 Hz, 1H, -CH-OH), 3.98 (d, *J* = 4.2 Hz, 2H, OCH_2_), 2.83-2.68 (m, 4H, NH-CH_2_ & NH-CH_2_CH_3_), 1.46-0.88 (m, 3H, -NH-CH_2_-CH_3_). ^13^C NMR (126 MHz, CDCl_3_) *δ* 159.5, 156.0, 147.2, 145.2, 139.3, 122.7, 119.0, 118.9, 115.5, 70.7, 67.5, 57.0, 49.4, 11.8. HRMS (ESI) [M+H^+^] calcd for C_16_H_20_ClN_2_O_3_: 323.1162, found: 323.1157.

**1-(4-((3-chloropyridin-2-yl)oxy)phenoxy)-3-((2,2,2-trifluoroethyl)amino)propan-2-ol (PyE3)**

Yellowish solid, yield 67.4%; ^1^H NMR (500 MHz, CDCl_3_) *δ* 8.01 (dd, *J* = 4.8, 1.7 Hz, 1H, pyridine-4-H), 7.74 (dd, *J* = 7.7, 1.7 Hz, 1H, pyridine-6-H), 7.11-7.08 (m, 2H, diphenyl-2,6-H), 6.96-6.92 (m, 3H, diphenyl-3,5-H & pyridine-5-H), 4.08-4.04 (m, 1H, -CH-OH), 4.00 (dd, *J* = 5.1, 1.8 Hz, 2H, OCH_2_), 3.30-3.21 (m, 2H, CH-CH_2_), 3.01 (dd, *J* = 12.3, 3.8 Hz, 1H, NH-CH_2_-C), 2.92 (d, *J* = 7.3 Hz, 1H, NH-CH_2_-C). ^13^C NMR (126 MHz, CDCl_3_) *δ* 159.5, 155.8, 147.3, 145.2, 139.3, 122.8, 119.1, 118.9, 115.4, 70.5, 68.9, 53.6, 51.6, 50.8 (d, ^2^*J_C-F_* = 93.2 Hz). ^19^F NMR (471 MHz, CDCl_3_) *δ* -71.68 (s, -F). HRMS (ESI) [M+H^+^] calcd for C_16_H_17_ClF_3_N_2_O_3_: 377.0880, found: 377.0888.

**1-(4-((3-chloropyridin-2-yl)oxy)phenoxy)-3-(isopropylamino)propan-2-ol (PyE4)**

White solid, yield 54.8%; ^1^H NMR (500 MHz, CDCl_3_) *δ* 8.00 (dd, *J* = 11.6, 2.4 Hz, 1H, pyridine-4-H), 7.75-7.72 (m, 1H, pyridine-6-H), 7.08 (d, *J* = 8.8 Hz, 2H, diphenyl-2,6-H), 6.97-6.91 (m, 3H, diphenyl-3,5-H & pyridine-5-H), 4.11-4.05 (m, 1H, CH-OH), 4.01-3.95 (m, 2H, OCH_2_), 2.95-2.89 (m, 2H, N-CH_2_), 2.76 (dd, *J* = 12.1, 8.2 Hz, 1H, CH-CH_3_), 1.12 (d, *J* = 6.3 Hz, 6H, -CH_3_). ^13^C NMR (126 MHz, CDCl_3_) *δ* 159.5, 156.0, 147.2, 145.2, 139.3, 122.7, 119.0, 118.9, 115.5, 71.0, 68.3, 49.3, 49.3, 23.0, 22.8. HRMS (ESI) [M+H^+^] calcd for C_17_H_22_ClN_2_O_3_: 337.1319, found: 337.1372.

**1-(4-((3-chloropyridin-2-yl)oxy)phenoxy)-3-((cyclopropylmethyl)amino)propan-2-ol (PyE5)**

Brown solid, yield 46.4%; ^1^H NMR (500 MHz, CDCl_3_ ) *δ* 8.00 (dd, *J* = 4.8, 1.7 Hz, 1H, pyridine-4-H), 7.73 (dd, *J* = 7.7, 1.7 Hz, 1H, pyridine-6-H), 7.10-7.06 (m, 2H, diphenyl-2,6-H), 6.96-6.91 (m, 3H, diphenyl-3,5-H & pyridine-5-H), 4.10-4.05 (m, 1H, CH-OH), 3.97 (d, *J* = 5.2 Hz, 2H, OCH_2_), 2.98-2.83 (m, 2H, CH-CH_2_-NH), 2.21-2.17 (m, 1H, triangle-1-H), 0.52-0.35 (m, 4H, ttiangle-2,3-H). ^13^C NMR (101 MHz, CDCl_3_) *δ* 159.5, 156.0, 147.1, 145.2, 139.3, 122.7, 119.0, 118.9, 115.4, 71.0, 68.2, 51.9, 30.6, 6.9, 6.8, 6.2. HRMS (ESI) [M+H^+^] calcd for C_18_H_22_ClN_2_O_3_: 349.1319, found: 349.1313.

**1-(tert-butylamino)-3-(4-((3-chloropyridin-2-yl)oxy)phenoxy)propan-2-ol (PyE6)**

White solid, yield 56.7%; ^1^H NMR (500 MHz, CDCl3) *δ* 8.00 (dd, *J* = 4.7, 1.8 Hz, 1H, pyridine-4-H), 7.73 (dd, *J* = 7.9, 1.6 Hz, 1H, pyridine-6-H), 7.09-7.06 (m, 2H, diphenyl-2,6-H), 6.96-6.91 (m, 3H, diphenyl-3,5-H & pyridine-5-H), 4.00-3.94 (m, 3H, CH-OH & OCH_2_), 2.87-2.67 (m, 2H, NH-CH_2_), 1.13 (s, 9H, -CH_3_).^13^C NMR (126 MHz, CDCl3) *δ* 159.5, 156.1, 147.1, 145.2, 139.3, 122.3, 119.0, 118.9, 115.5, 71.0, 68.6, 50.6, 44.8, 29.2. HRMS (ESI) [M+H^+^] calcd for C_18_H_24_ClN_2_O_3_: 351.1475, found: 351.1470.

**1-(sec-butylamino)-3-(4-((3-chloropyridin-2-yl)oxy)phenoxy)propan-2-ol (PyE7)**

White solid, yield 73.0%; ^1^H NMR (500 MHz, CDCl_3_) *δ* 8.00 (dd, *J* = 4.9, 1.7 Hz, 1H, pyridine-4-H), 7.73 (dd, *J* = 7.7, 1.7 Hz, 1H, pyridine-6-H)), 7.09-7.06 (m, 2H, diphenyl-2,6-H), 6.96-6.91 (m, 3H, diphenyl-3,5-H & pyridine-5-H), 4.07-4.03 (m, 1H, -CH-OH), 4.00-3.94 (m, 2H, OCH_2_), 2.95-2.87 (m, 1H, NH), 2.79-2.58 (m, 5H, NH-CH_2_ & NH-CH & CH_2_CH_3_), 1.08 (d, *J* = 6.3 Hz, 3H, CH-CH_3_), 0.91 (t, *J* = 7.5 Hz, 3H, CH_2_CH_3_). ^13^C NMR (126 MHz, CDCl_3_) *δ* 159.5, 156.0, 147.1, 145.2, 139.3, 122.7, 119.0, 118.9, 115.4, 70.9, 68.5, 68.2, 55.1, 54.8, 49.3, 49.0, 29.6, 19.9, 10.4. HRMS (ESI) [M+H^+^] calcd for C_18_H_24_ClN_2_O_3_: 351.1475, found: 351.1510.

**1-(4-((3-chloropyridin-2-yl)oxy)phenoxy)-3-((2(dimethylamino)ethyl)amino)propan-2-ol (PyE8)**

Yellowish oily substance, yield 34.9%; ^1^H NMR (500 MHz, CDCl_3_) *δ* 7.99 (dd, *J* = 4.9, 1.7 Hz, 1H, pyridine-4-H), 7.72 (dd, *J* = 7.7, 1.7 Hz, 1H, pyridine-6-H), 7.08-7.05 (m, 2H, diphenyl-2,6-H), 6.96-6.91 (m, 3H, diphenyl-3,5-H & pyridine-5-H), 4.08-4.04 (m, 1H, CH-OH), 3.99-3.93 (m, 2H, OCH_2_), 2.89 (dd, *J* = 12.4, 3.6 Hz, 1H, NH-CH_2_-CH), 2.79-2.73 (m, 3H, NH-CH_2_-CH & NH-CH_2_-CH_2_), 2.50-2.39 (m, 2H, N-CH_2_), 2.24 (s, 6H, CH_3_). ^13^C NMR (126 MHz, CDCl_3_) *δ* 159.5, 156.1, 147.0, 145.2, 139.3, 122.7, 119.0, 118.8, 115.4, 70.9, 68.4, 59.0, 52.0, 47.0, 45.5. HRMS (ESI) [M+H^+^] calcd for C_18_H_25_ClN_3_O_3_: 366.1584, found: 366.1628.

**1-(4-((3-chloropyridin-2-yl)oxy)phenoxy)-3-(2,6-dimethylmorpholino)propan-2-ol (PyE9)**

White solid, yield 26.3%; ^1^H NMR (500 MHz, CDCl_3_ ) *δ* 8.00 (dd, *J* = 4.6, 1.7 Hz, 2H, pyridine-4-H), 7.73 (dd, *J* = 7.5, 1.7 Hz, 2H, pyridine-6-H), 7.10-7.06 (m, 2H, diphenyl-2,6-H), 6.97-6.91 (m, 3H, diphenyl-3,5-H & pyridine-5-H), 4.14-4.09 (m, 1H, CH-OH), 3.98 (d, *J* = 5.3 Hz, 2H, OCH_2_), 3.74-3.64 (m, 2H, morpholine-3,5-H), 2.77 (dd, *J* = 70.6, 10.9 Hz, 2H, morpholine-2-H), 2.57-2.48 (m, 2H, morpholine-6-H), 2.07-2.02 (m, 1H, N-CH_2_-CH), 1.82-1.78 (m, 1H, N-CH_2_-CH), 1.18-1.16 (m, 6H, -CH_3_). ^13^C NMR (126 MHz, CDCl_3_) *δ* 159.5, 156.0, 147.2, 145.2, 139.3, 122.7, 119.0, 118.9, 115.5, 72.0,71.8, 70.7, 65.5, 60.8,60.7, 58.4, 19.2,19.1. HRMS (ESI) [M+H^+^] calcd for C_20_H_26_ClN_2_O_4_: 393.1581, found: 393.1576.

**1-(4-((3-chloropyridin-2-yl)oxy)phenoxy)-3-morpholinopropan-2-ol (PyE10)**

White solid, yield 79.6%; ^1^H NMR (500 MHz, CDCl_3_ ) *δ* 8.00 (dd, *J* = 5.3, 1.1 Hz, 1H, pyridine-4-H), 7.74-7.72 (m, 1H, pyridine-6-H), 7.09-7.06 (m, 2H, diphenyl-2,6-H), 6.97-6.91 (m, 3H, diphenyl-3,5-H & pyridine-5-H), 4.13-4.09 (m, 1H, CH-OH), 3.99 (d, *J* = 5.0 Hz, 2H, OCH_2_), 3.77-3.70 (m, 4H, morpholine-3,5-H), 2.69-2.65 (m, 2H, CH-CH_2_), 2.59-2.45 (m, 4H, morpholine-2,6-H). ^13^C NMR (126 MHz, CDCl_3_) *δ* 159.5, 156.0, 147.2, 145.2, 139.3, 122.7, 119.0, 118.9, 115.5, 70.7, 67.17, 65.6, 61.2, 53.9. HRMS (ESI) [M+H^+^] calcd for C_18_H_22_ClN_2_O_4_: 365.1268, found: 365.1263.

**1-(4-((3-chloropyridin-2-yl)oxy)phenoxy)-3-(3-methylpiperidin-1-yl)propan-2-ol (PyE11)**

White solid, yield 82.3%; ^1^H NMR (500 MHz, CDCl_3_) *δ* 8.00 (dd, *J* = 4.7, 1.7 Hz, 1H, pyridine-4-H), 7.73 (dd, *J* = 7.9, 1.7 Hz, 1H, pyridine-6-H), 7.09-7.06 (m, 2H, diphenyl-2,6-H), 6.97-6.91 (m, 3H, diphenyl-3,5-H & pyridine-5-H), 4.13-4.07 (m, 1H, -CH-OH), 4.00-3.94 (m, 2H, OCH_2_), 2.95-2.73 (m, 2H, N-CH_2_), 2.54-2.45 (m, 2H, piperidine-6-H), 2.24-1.91 (m, 2H, piperidine-2-H), 1.73-1.55 (m, 4H, piperidine-4,5-H), 1.25-1.21 (m, 1H, piperidine-3-H), 0.87 (d, *J* = 6.3 Hz, 3H, -CH_3_). ^13^C NMR (101 MHz, CDCl_3_) *δ* 159.5, 156.2, 147.1, 145.2, 139.3, 122.6, 119.0, 118.9, 115.5, 70.9, 65.4, 61.0, 55.7, 53.0, 32.9, 31.3, 25.5, 19.7. HRMS (ESI) [M+H^+^] calcd for C_20_H_26_ClN_2_O_3_: 377.1632, found: 377.1627.

**1-(4-((3-chloropyridin-2-yl)oxy)phenoxy)-3-(2-methylpiperidin-1-yl)propan-2-ol**

**(PyE12)**

Yellow solid, yield 40.8%; ^1^H NMR (500 MHz, CDCl_3_) *δ* 8.00 (dd, *J* = 4.9, 1.7 Hz, 1H, pyridine-4-H), 7.73 (dd, *J* = 7.7, 1.7 Hz, 1H, pyridine-6-H), 7.09-7.05 (m, 2H, diphenyl-2,6-H), 6.97-6.90 (m, 3H, diphenyl-3,5-H & pyridine-5-H), 4.08-3.93 (m, 3H, -CH-OH & OCH_2_), 2.89-2.65 (m, 2H, N-CH_2_), 2.51-2.30 (m, 2H, piperidine-6-H), 1.71-1.24 (m, 7H, piperidine-2,3,4,5-H), 1.08 (t, *J* = 6.5 Hz, 3H, -CH_3_). ^13^C NMR (101 MHz, CDCl_3_) *δ* 159.5, 156.2, 147.1, 145.2, 139.3, 122.6, 119.0, 110.9, 115.5, 71.0, 66.4, 65.1, 55.7, 52.6, 34.7, 33.2, 26.1, 25.8. HRMS (ESI) [M+H^+^] calcd for C_20_H_26_ClN_2_O_3_: 377.1632, found: 377.1684.

**1-(4-((3-chloropyridin-2-yl)oxy)phenoxy)-3-(4-methylpiperidin-1-yl)propan-2-ol (PyE13)**

Yellow solid, yield 63.5%; ^1^H NMR (500 MHz, CDCl_3_ ) *δ* 8.01 (dd, *J* = 4.9, 1.7 Hz, 1H, pyridine-4-H), 7.73 (dd, *J* = 7.7, 1.7 Hz, 1H, pyridine-6-H), 7.10-7.05 (m, 2H, diphenyl-2,6-H), 6.99-6.91 (m, 3H, diphenyl-3,5-H & pyridine-5-H), 4.11-4.07 (m, 1H, CH-OH), 4.01-3.94 (m, 2H, OCH_2_), 3.01-2.98 (m, 1H, N-CH_2_), 2.82 (d, *J* = 12.0 Hz, 1H, N-CH_2_), 2.55-2.48 (m, 2H, piperazine-2-H), 2.33-2.27 (m, 1H, piperazine-6-H), 2.03-1.98 (m, 1H, piperazine-6-H), 1.66-1.62 (m, 2H, piperazine-3-H), 1.45-1.35 (m, 1H, piperazine-4-H), 1.33-1.24 (m, 2H, piperazine-5-H), 0.93 (d, *J* = 6.5 Hz, 3H, -CH_3_). ^13^C NMR (101 MHz, CDCl_3_) *δ* 159.5, 156.2, 147.1, 145.3, 139.3, 122.7, 119.0, 118.9, 115.5, 70.9, 65.5, 60.9, 55.8, 52.8, 34.7, 34.4, 30.8, 22.0. HRMS (ESI) [M+H^+^] calcd for C_20_H_26_ClN_2_O_3_: 377.1632, found: 377.1667.

**1-(4-((3-chloropyridin-2-yl)oxy)phenoxy)-3-(4-methylpiperazin-1-yl)propan-2-ol (PyE14)**

Dark green solid, yield 52.8%; ^1^H NMR (500 MHz, CDCl_3_ ) *δ* 7.99 (dd, *J* = 4.7, 1.7 Hz, 1H, pyridine-4-H), 7.72 (dd, *J* = 7.9, 1.7 Hz, 1H, pyridine-6-H), 7.09-7.05 (m, 2H, diphenyl-2,6-H), 6.96-6.90 (m, 3H, diphenyl-3,5-H & pyridine-5-H), 4.11-4.06 (m, 1H, -CH-OH), 3.97 (d, *J* = 4.9 Hz, 2H, OCH_2_), 2.72-2.69 (m, 2H, N-CH_2_), 2.59-2.50 (m, 6H, piperazine-2,3,6-H), 2.29 (s, 3H, N-CH_3_), 1.24 (s, 2H, piperazine-5-H). ^13^C NMR (126 MHz, CDCl_3_) *δ* 159.5, 156.1, 147.2, 145.2, 139.3, 122.6, 119.0, 118.9, 115.5, 70.8, 65.6, 60.5, 55.3, 46.1, 29.8. HRMS (ESI) [M+H^+^] calcd for C_19_H_25_ClN_3_O_3_: 378.1584, found: 378.1606.

**4-(3-(4-((3-chloropyridin-2-yl)oxy)phenoxy)-2-hydroxypropyl)piperazin-2-one**

**(PyE15)**

White solid, yield 70.1%; ^1^H NMR (400 MHz, CDCl_3_) *δ* 8.00 (dd, *J* = 4.9, 1.7 Hz, 1H, pyridine-4-H), 7.74 (dd, *J* = 7.7, 1.7 Hz, 1H, pyridine-6-H), 7.11-7.07 (m, 2H, diphenyl-2,6-H), 6.97-6.92 (m, 3H, diphenyl-3,5-H & pyridine-5-H), 6.42 (s, 1H, CONH), 4.16-4.10 (m, 1H, -CH-OH), 4.03-3.99 (m, 2H, OCH_2_), 3.48-3.32 (m, 3H, COCH_2_ & OH), 3.24-3.17 (m, 2H, NHCH_2_), 2.91-2.61 (m, 4H, CHCH_2_N & CH_2_N).^13^C NMR (101 MHz, CDCl_3_) *δ* 169.0, 159.4, 155.8, 147.2, 145.1, 139.2, 122.7, 119.0, 118.9, 115.4, 70.3, 66.2, 59.7, 57.1, 49.4, 41.3. HRMS (ESI) [M+H^+^] calcd for C_19_H_24_ClN_4_O_4_: 407.1486, found: 407.1481.

**1-(4-((3-chloropyridin-2-yl)oxy)phenoxy)-3-(4-ethylpiperazin-1-yl)propan-2-ol (PyE16)**

Yellow solid, yield 72.1%; ^1^H NMR (500 MHz, CDCl_3_) *δ* 7.99 (d, *J* = 4.5 Hz, 1H, pyridine-4-H), 7.72 (d, *J* = 7.5 Hz, 1H, pyridine-6-H), 7.07 (d, *J* = 8.5 Hz, 2H, diphenyl-2,6-H), 6.95-6.90 (m, 3H, diphenyl-3,5-H & pyridine-5-H), 4.08 (dd, *J* = 9.0, 4.4 Hz, 1H, CH-OH), 3.97 (d, *J* = 4.6 Hz, 2H, OCH_2_), 2.73 (s, 2H, N-CH_2_), 2.57-2.40 (m, 8H, piperazine-2,3,5,6-H), 1.24 (s, 2H, -N-CH_2_-CH_3_), 1.09 (t, *J* = 7.0 Hz, 3H, -N-CH_2_-CH_3_). ^13^C NMR (101 MHz, CDCl_3_) *δ* 159.5, 156.1, 147.1, 145.2, 139.3, 122.7, 119.0, 118.9, 115.5, 70.8, 65.6, 60.5, 58.4, 52.9, 52.4, 29.8, 18.6, 12.0. HRMS (ESI) [M+H^+^] calcd for C_20_H_27_ClN_3_O_3_: 392.1741, found: 392.1736.

**1-(4-(3-(4-((3-chloropyridin-2-yl)oxy)phenoxy)-2-hydroxypropyl)piperazin-1-yl)ethan-1-one (PyE17)**

Brownish-yellow crystal, yield 76.7%; ^1^H NMR (500 MHz, CDCl_3_ ) *δ* 7.98 (dd, *J* = 5.0, 1.7 Hz, 1H, pyridine-4-H), 7.72 (dd, *J* = 7.9, 1.7 Hz, 1H, pyridine-6-H), 7.08-7.05 (m, 2H, diphenyl-2,6-H), 6.95-6.90 (m, 3H, diphenyl-3,5-H & pyridine-5-H), 4.13-4.08 (m, 1H, CH-OH), 4.00-3.95 (m, 2H, OCH_2_), 3.68-3.46 (m, 4H, piperazine-3,5-H), 2.66-2.52 (m, 4H, piperazine-2,6-H), 2.50-2.41 (m, 2H, CH-CH_2_), 2.08 (s, 3H, -CH_3_). ^13^C NMR (126 MHz, CDCl_3_) *δ* 169.1, 159.4, 155.9, 147.2, 145.2, 139.3, 122.6, 119.0, 118.9, 115.4, 70.6, 65.9, 60.6, 53.6, 53.1, 46.3, 41.5, 21.4. HRMS (ESI) [M+H^+^] calcd for C_20_H_25_ClN_3_O_4_: 406.1534, found: 406.1528.

**1-(4-(tert-butyl)piperazin-1-yl)-3-(4-((3-chloropyridin-2-yl)oxy)phenoxy)propan-2-ol (PyE18)**

Brown solid, yield 33.7%; ^1^H NMR (500 MHz, CDCl_3_ ) *δ* 7.99 (dd, *J* = 4.9, 1.7 Hz, 1H, pyridine-4-H), 7.72 (dd, *J* = 7.7, 1.7 Hz, 1H, pyridine-6-H), 7.08-7.05 (m, 2H, diphenyl-2,6-H), 6.96-6.90 (m, 3H, diphenyl-3,5-H & pyridine-5-H), 4.10-4.06 (m, 1H, CH-OH), 3.97 (d, *J* = 4.9 Hz, 2H, OCH_2_), 2.71-2.47 (m, 10H, N-CH_2_ & piperazine-2,3,5,6-H), 1.07 (s, 9H, -CH_3_). ^13^C NMR (126 MHz, CDCl_3_) *δ* 159.5, 156.1, 147.2, 145.2, 139.3, 122.7, 119.0, 118.9, 115.5, 70.9, 65.6, 60.5, 53.9, 45.9, 29.8, 26.0. HRMS (ESI) [M+H^+^] calcd for C_22_H_31_ClN_3_O_3_: 420.2054, found: 420.2049.

**1-(4-((3-chloropyridin-2-yl)oxy)phenoxy)-3-(4-isopropylpiperazin-1-yl)propan-2-ol (PyE19)**

Brown solid, yield 95.6%; ^1^H NMR (500 MHz, CDCl_3_ ) *δ* 7.99 (dd, *J* = 4.8, 1.7 Hz, 1H, pyridine-4-H), 7.72 (dd, *J* = 7.7, 1.7 Hz, 1H, pyridine-6-H), 7.08-7.05 (m, 2H, diphenyl-2,6-H), 6.96-6.90 (m, 3H, diphenyl-3,5-H & pyridine-5-H), 4.11-4.06 (m, 1H, CH-OH), 3.97 (d, *J* = 4.9 Hz, 2H, OCH_2_), 2.71-2.49 (m, 11H, N-CH_2_ & piperazine-2,3,5,6-H & N-CH), 1.05 (d, *J* = 6.5 Hz, 6H, -CH_3_). ^13^C NMR (126 MHz, CDCl_3_) *δ* 159.5, 156.1, 147.1, 145.2, 139.3, 122.6, 119.0, 118.9, 115.5, 70.8, 65.6, 60.6, 54.5, 53.7, 48.9, 18.8, 18.7. HRMS (ESI) [M+H^+^] calcd for C_21_H_29_ClN_3_O_3_: 406.1897, found: 406.1941.

**4-(3-(4-((3-chloropyridin-2-yl)oxy)phenoxy)-2-hydroxypropyl)piperazine-1-carbaldehyde (PyE20)**

Pink solid, yield 60.2%; ^1^H NMR (500 MHz, CDCl_3_ ) *δ* 8.02 (s, 1H, N-CHO), 7.99 (dd, *J* = 4.8, 1.7 Hz, 1H, pyridine-4-H), 7.73 (dd, *J* = 7.7, 1.7 Hz, 1H, pyridine-6-H), 7.09-7.06 (m, 2H, diphenyl-2,6-H), 6.96-6.91 (m, 3H, diphenyl-3,5-H & pyridine-5-H), 4.14-4.09 (m, 1H, CH-OH), 4.02-3.96 (m, 2H, OCH_2_), 3.63-3.38 (m, 4H, piperazine-3,5-H), 2.69-2.56 (m, 4H, piperazine-2,6-H), 2.53-2.43 (m, 2H, N-CH_2_). ^13^C NMR (126 MHz, CDCl_3_) *δ* 160.8, 159.4, 155.9, 147.2, 145.2, 139.3, 122.7, 119.1, 118.9, 115.5, 70.5, 66.0, 60.7, 53.9, 52.8, 45.7, 40.0. HRMS (ESI) [M+H^+^] calcd for C_19_H_23_ClN_3_O_4_: 392.1377, found: 392.1381.

**tert-butyl4-(3-(4-((3-chloropyridin-2-yl)oxy)phenoxy)-2-hydroxypropyl)piperazine-1-carboxylate (PyE21)**

Yellow solid, yield 66.2%; ^1^H NMR (500 MHz, CDCl_3_ ) *δ* 8.00 (dd, *J* = 4.7, 1.7 Hz, 1H, pyridine-4-H), 7.73 (dd, *J* = 7.5, 1.7 Hz, 1H, pyridine-6-H), 7.09-7.06 (m, 2H, diphenyl-2,6-H), 6.96-6.91 (m, 3H, diphenyl-3,5-H & pyridine-5-H), 4.12-4.08 (m, 1H, CH-OH), 3.99 (d, *J* = 5.0 Hz, 2H, OCH_2_), 3.47-3.41 (m, 4H, piperazine-3,5-H), 2.61-2.51 (m, 4H, piperazine-2,6-H) ,2.44-2.39 (m, 2H, N-CH_2_), 1.46 (s, 9H, -CH_3_). ^13^C NMR (126 MHz, CDCl_3_) *δ* 159.5, 156.0, 154.8, 147.2, 145.2, 139.3, 122.7, 119.0, 118.9, 115.5, 79.9, 76.9, 70.6, 65.7, 60.7, 53.3, 28.5. HRMS (ESI) [M+H^+^] calcd for C_23_H_31_ClN_3_O_5_: 464.2001, found: 464. 1963.

**1-(4-((3-chloropyridin-2-yl)oxy)phenoxy)-3-(4-phenylpiperazin-1-yl)propan-2-ol**

**(PyE22)**

Yellow solid, yield 57.4%; ^1^H NMR (500 MHz, CDCl_3_ ) *δ* 8.02 (dd, *J* = 5.0, 1.7 Hz, 1H, pyridine-4-H), 7.74 (dd, *J* = 7.9, 1.7 Hz, 1H, pyridine-6-H), 7.30-7.26 (m, 2H, benzene-3,5-H), 7.12-7.09 (m, 2H, diphenyl-2,6-H), 7.00-6.92 (m, 5H, benzene-2,4,6-H & diphenyl-3,5-H), 6.88 (t, *J* = 7.4 Hz, 1H, pyridine-5-H), 4.18-4.14 (m, 1H, CH-OH), 4.03 (d, *J* = 4.8 Hz, 2H, OCH_2_), 3.27-3.19 (m, 4H, piperazine-3,5-H), 2.88-2.83 (m, 2H, N-CH_2_), 2.68-2.58 (m, 4H, piperazine-2,6-H). ^13^C NMR (126 MHz, CDCl_3_) *δ* 159.5, 156.1, 147.1, 145.2, 139.3, 122.6, 119.0, 118.9, 115.5, 70.8, 65.6, 60.6, 54.5, 53.7, 48.9, 18.8, 18.7. HRMS (ESI) [M+H^+^] calcd for C_24_H_27_ClN_3_O_3_: 440.1741, found: 440.1735.

**1-(4-((3-chloropyridin-2-yl)oxy)phenoxy)-3-(4-(pyrimidin-2-yl)piperazin-1-yl)propan-2-ol (PyE23)**

Yellow solid, yield 77.7%; ^1^H NMR (500 MHz, CDCl_3_ ) *δ* 8.31 (d, *J* = 4.6 Hz, 2H, pyrimidine-3,5-H), 8.01 (dd, *J* = 4.8, 1.7 Hz, 1H, pyridine-4-H), 7.73 (dd, *J* = 7.6, 1.7 Hz, 1H, pyridine-6-H), 7.10-7.07 (m, 2H, diphenyl-2,6-H), 6.98-6.91 (m, 3H, diphenyl-3,5-H & pyridine-5-H), 6.50 (t, *J* = 4.7 Hz, 1H, pyrimidine-4-H), 4.18-4.14 (m, 1H, CH-OH), 4.02 (d, *J* = 4.8 Hz, 2H, OCH_2_), 3.90-3.82 (m, 4H, piperazine-3,5-H), 2.76-2.72 (m, 2H, N-CH_2_), 2.66-2.52 (m, 4H, piperazine-2,6-H). ^13^C NMR (126 MHz, CDCl_3_) *δ* 161.8, 159.58, 157.9, 156.1, 147.2, 145.3, 139.3, 122.7, 119.1, 118.9, 115.5, 110.2, 70.7, 65.7, 60.8, 53.4, 43.9. HRMS (ESI) [M+H^+^] calcd for C_22_H_25_ClN_5_O_3_: 442.1646, found: 442.1665.

**1-(4-((3-chloropyridin-2-yl)oxy)phenoxy)-3-((4,6-dichloropyrimidin-2-yl)amino)propan-2-ol (PyE24)**

White solid, yield 38.2%; ^1^H NMR (400 MHz, CDCl_3_ ) *δ* 8.01 (dd, *J* = 4.9, 1.7 Hz, 1H, pyridine-4-H), 7.74 (dd, *J* = 7.7, 1.7 Hz, 1H, pyridine-6-H), 7.26 (s, 1H, pyrimidine-4-H), 7.11-7.07 (m, 2H, diphenyl-2,6-H), 6.98-6.92 (m, 3H, diphenyl-3,5-H & pyridine-5-H), 4.22 (dd, *J* = 11.0, 3.2 Hz, 1H, CH-OH), 3.96 (dd, *J* = 11.0, 5.7 Hz, 1H, OCH_2_), 3.39-3.35 (m, 1H, OCH_2_), 2.93-2.91 (m, 1H, NH-CH_2_), 2.77 (dd, *J* = 4.9, 2.7 Hz, 1H, NH-CH_2_). ^13^C NMR (101 MHz, CDCl_3_) *δ* 162.3, 159.5, 155.8, 147.4, 145.3, 139.3, 122.8, 119.1, 118.9, 115.6, 110.5, 106.0, 69.3, 50.3, 44.9. HRMS (ESI) [M+H^+^] calcd for C_18_H_16_Cl_3_N_4_O_3_: 441.0288, found: 441.0282.

**1-(4-((3-chloropyridin-2-yl)oxy)phenoxy)-3-(pyrimidin-2-ylamino)propan-2-ol (PyE25)**

White solid, yield 41.2%; ^1^H NMR (400 MHz, CDCl_3_ ) *δ* 8.27 (d, *J* = 4.9 Hz, 2H, pyrimidine-3,5-H), 8.00 (dd, *J* = 4.9, 1.7 Hz, 1H, pyridine-4-H), 7.73 (dd, *J* = 7.7, 1.7 Hz, 1H, pyridine-6-H), 7.10-7.06 (m, 2H, diphenyl-2,6-H), 6.96-6.91 (m, 3H, diphenyl-3,5-H & pyridine-5-H), 6.58 (t, *J* = 4.9 Hz, 1H, pyrimidine-4-H), 5.86 (t, *J* = 5.7 Hz, 1H, OH), 4.24-4.19 (m, 1H, CH-OH), 4.04-3.97 (m, 2H, OCH_2_), 3.82-3.76 (m, 1H, NH-CH_2_), 3.65-3.58 (m, 1H, NH-CH_2_). ^13^C NMR (101 MHz, CDCl_3_) *δ* 163.0, 159.5, 158.1, 155.9, 147.2, 145.2, 139.3, 122.8, 119.0, 118.9, 115.4, 111.2, 70.6, 69.8, 45.4. HRMS (ESI) [M+H^+^] calcd for C_18_H_18_ClN_4_O_3_: 373.1067, found: 373.1071.

**1-(4-((3-chloropyridin-2-yl)oxy)phenoxy)-3-((4-fluoro-3-(trifluoromethyl)benzyl)amino)propan-2-ol (PyE26)**

Brown solid, yield 56.1%; ^1^H NMR (500 MHz, CDCl_3_) *δ* 8.03-8.00 (m, 1H, pyridine-4-H), 7.77-7.74 (m, 1H, pyridine-6-H), 7.13-7.08 (m, 2H, diphenyl-2,6-H), 7.04 (q, *J* = 9.4, 8.3 Hz, 1H, benzene-2-H), 6.98-6.93 (m, 3H, diphenyl-3,5-H & pyridine-5-H), 6.87-6.80 (m, 2H, benzene-5,6-H), 5.32-5.29 (m, 1H, CH-OH), 4.30-4.25 (m, 1H, CH-OH), 4.11-4.03 (m, 2H, OCH_2_), 3.43-3.26 (m, 2H, CH_2_-benzene). ^13^C NMR (101 MHz, CDCl_3_) *δ* 159.3, 155.4, 151.6, 147.4, 145.1, 143.7, 139.3, 122.8, 122.7 (d, ^1^*J_C-F_* = 273.7 Hz), 119.1, 118.8, 118.2 (d, ^3^*J_C-F_* = 6.8 Hz), 117.7 (d, ^2^*J_C-F_* = 22.2 Hz), 115.4, 111.14 (d, ^4^*J_C-F_* = 4.8 Hz), 70.3, 68.5, 62.2, 60.9, 47.3. ^19^F NMR (471 MHz, CDCl_3_) *δ* -61.37 (s, -3F), -129.12 (s, -F). HRMS (ESI) [M+H^+^] calcd for C_22_H_20_ClF_4_N_2_O_3_: 471.1099, found: 471.1093.

**1-((4-(tert-butyl)phenyl)amino)-3-(4-((3-chloropyridin-2-yl)oxy)phenoxy)propan-2-ol (PyE27)**

Yellow solid, yield 57.2%; ^1^H NMR (400 MHz, CDCl_3_) *δ* 8.02 (dd, *J* = 4.9, 1.7 Hz, 1H, pyridine-4-H), 7.75 (dd, *J* = 7.7, 1.7 Hz, 1H, pyridine-6-H), 7.25-7.22 (m, 2H, benzene-2,6-H), 7.13-7.09 (m, 2H, diphenyl-2,6-H), 6.98-6.93 (m, 3H, diphenyl-3,5-H & pyridine-5-H), 6.68-6.64 (m, 2H, benzene-3,5-H), 4.28-4.22 (m, 1H, CH-OH), 4.10-4.03 (m, 2H, OCH_2_), 3.45-3.27 (m, 2H, CH-CH_2_-NH), 1.30 (s, 9H, CH_3_). ^13^C NMR (126 MHz, CDCl_3_) *δ* 159.5, 155.8, 147.3, 145.8, 145.2, 141.1, 139.3, 126.2, 122.8, 119.1, 118.9, 115.5, 113.2, 70.6, 68.9, 47.0, 34.0, 31.6. HRMS (ESI) [M+H^+^] calcd for C_24_H_28_ClN_2_O_3_: 427.1788, found: 427.1796.

**1-((4-chlorobenzyl)amino)-3-(4-((3-chloropyridin-2-yl)oxy)phenoxy)propan-2-ol (PyE****28)**

Yellow solid, yield 48.6%; ^1^H NMR (500 MHz, CDCl_3_) *δ* 8.00 (dd, *J* = 4.9, 1.7 Hz , 1H, pyridine-4-H), 7.74 (dd, *J* = 7.7, 1.7 Hz, 1H, pyridine-6-H), 7.31-7.26 (m, 4H, -NH-CH_2_-benzene-H), 7.10-7.07 (m, 2H, diphenyl-2,6-H), 6.95-6.92 (m, 3H, diphenyl-3,5-H & pyridine-5-H), 4.08 (dt, *J* = 8.9, 5.0 Hz, 1H, CH-OH), 3.98 (d, *J* = 5.1 Hz, 2H, OCH_2_), 3.81 (d, *J* = 3.6 Hz, 2H, -NH-CH_2_-benzene), 2.87 (dd, *J* = 12.2, 3.9 Hz, 1H, -CH-CH_2_-NH-), 2.78 (dd, *J* = 12.2, 7.7 Hz, 1H, -CH-CH_2_-NH-). ^13^C NMR (101 MHz, CDCl_3_) *δ* 159.5, 155.9, 147.3, 145.2, 139.3, 138.5, 133.0, 129.6, 128.8, 122.8, 119.1, 118.9, 115.5, 70.9, 68.6, 53.2, 51.3. HRMS (ESI) [M+H^+^] calcd for C_21_H_21_Cl_2_N_2_O_3_: 419.0929, found: 419.0924.

**1-(4-((3-chloropyridin-2-yl)oxy)phenoxy)-3-((2,4-dichlorobenzyl)(methyl)amino)propan-2-ol (PyE29)**

Yellow oily substance, yield 75.5%; ^1^H NMR (500 MHz, CDCl_3_) *δ* 8.01 (dd, *J* = 5.0, 1.6 Hz, 1H, pyridine-4-H), 7.74 (dd, *J* = 7.6, 1.8 Hz, 1H, pyridine-6-H), 7.39 (d, *J* = 2.2 Hz, 1H, benzene-3-H), 7.32 (d, *J* = 8.5 Hz, 1H, benzene-5-H), 7.23 (dd, *J* = 8.3, 2.2 Hz, 1H, benzene-6-H), 7.10-7.06 (m, 2H, diphenyl-2,6-H), 6.95-6.92 (m, 3H, diphenyl-3,5-H & pyridine-5-H), 4.12 (dd, *J* = 9.4, 4.8 Hz, 1H, CH-OH), 4.01-3.95 (m, 2H, OCH_2_), 3.73 (d, *J* = 13.5 Hz, 1H, -N-CH_2_-benzene), 3.62 (d, *J* = 13.6 Hz, 1H, -N-CH_2_-benzene), 2.69-2.61 (m, 2H, -CH-CH_2_-N-), 2.30 (s, 3H, -CH_3_). ^13^C NMR (126 MHz, CDCl_3_) *δ* 159.5, 156.1, 147.2, 145.2, 139.3, 135.3, 134.7, 133.8, 132.1, 129.7, 127.2, 122.7, 119.0, 118.9, 115.4, 70.6, 66.4, 60.2, 59.3, 42.3. HRMS (ESI) [M+H^+^] calcd for C_22_H_22_Cl_3_N_2_O_3_: 467.0696, found: 467.0691.

**1-(4-((3-chloropyridin-2-yl)oxy)phenoxy)-3-((4-phenoxyphenyl)amino)propan-2-ol (PyE30)**

Brown oily substance, yield 17.8%; ^1^H NMR (500 MHz, CDCl_3_ ) *δ* 8.01-8.00 (m, 1H, pyridine-4-H), 7.76-7.73 (m, 1H, pyridine-6-H), 7.30-7.27 (m, 2H, benzene-3,5-H), 7.12-7.09 (m, 2H, benzene-2',6'-H), 7.04-7.00 (m, 1H, benzene-4-H), 6.98-6.92 (m, 7H, pyridine-5-H & diphenyl-2,6-H & benzene-3',5'-H & benzene-2,6-H), 6.74-6.71 (m, 2H, diphenyl-3,5-H), 5.30 (d, *J* = 1.2 Hz, 1H, -CH-OH), 4.31-4.27 (m, 1H, -CH-OH), 4.11-4.04 (m, 2H, OCH_2_), 3.49-3.41 (m, 1H, NH-CH_2_), 3.33-3.28 (m, 1H, NH-CH_2_).^13^C NMR (126 MHz, CDCl_3_) *δ* 159.5, 158.9, 155.7, 148.7, 147.4, 145.2, 144.1, 139.3, 129.7, 122.9, 122.3, 121.3, 119.1, 118.9, 117.4, 115.4, 115.0, 70.5, 68.7, 47.7. HRMS (ESI) [M+H^+^] calcd for C_27_H_26_ClN_2_O_4_: 477.1581, found: 477.1576.

**1-(benzyl((*S*)-1-phenylethyl)amino)-3-(4-((3-chloropyridin-2-yl)oxy)phenoxy)propan-2-ol (PyE31)**

Brown oily substance, yield 57.0%; ^1^H NMR (400 MHz, CDCl_3_ ) *δ* 8.02 (dd, *J* = 4.9, 1.7 Hz, 1H, pyridine-4-H), 7.74 (dd, *J* = 7.7, 1.7 Hz, 1H, pyridine-6-H), 7.38-7.27 (m, 10H, CH_2_-benzene-2,3,4,5,6 & CH-benzene-2,3,4,5,6-H), 7.09-7.05 (m, 2H, diphenyl-2,6-H), 6.95-6.85 (m, 3H, diphenyl-3,5-H & pyridine-5-H), 4.05-3.97 (m, 2H, OCH_2_), 3.89-3.83 (m, 1H, CH-OH), 3.81-3.73 (m, 2H, CH_2_-benzene), 3.63 (d, *J* = 13.4 Hz, 1H, CH-benzene), 2.78 (dd, *J* = 13.0, 4.3 Hz, 1H, CH-CH_2_), 2.55 (dd, *J* = 13.0, 9.1 Hz, 1H, CH-CH_2_), 1.49-1.40 (m, 3H, -CH_3_).^13^C NMR (126 MHz, CDCl_3_) *δ* 159.5, 156.0,147.0, 145.2, 142.8, 139.5, 139.2, 129.1, 128.7, 128.4, 128.0, 127.4,127.3, 122.6, 119.0,118.9, 115.3, 70.8, 66.4, 57.4, 55.1, 52.1, 12.1. HRMS (ESI) [M+H^+^] calcd for C_29_H_30_ClN_2_O_3_: 489.1945, found: 489.1971.

**1-(4-((3-chloropyridin-2-yl)oxy)phenoxy)-3-((thiophen-2-ylmethyl)amino)propan-2-ol (PyE32)**

Brown oily substance, yield 64.5%; ^1^H NMR (500 MHz, CDCl_3_) *δ* 8.00 (dd, *J* = 4.8, 1.7 Hz, 1H, pyridine-4-H), 7.73 (dd, *J* = 7.6, 1.7 Hz, 1H, pyridine-6-H), 7.22 (dd, *J* = 4.9, 1.4 Hz, 1H, thiophene-5-H), 7.10-7.06 (m, 2H, diphenyl-2,6-H), 6.96-6.92 (m, 5H, diphenyl-3,5-H & pyridine-5-H & thiophene-3,4-H), 4.10-4.04 (m, 3H, CH-OH & OCH_2_), 3.98 (d, *J* = 5.2 Hz, 2H, NH-CH_2_-thiophene), 2.91 (dd, *J* = 12.2, 3.9 Hz, 1H, CH-CH_2_), 2.81 (dd, *J* = 12.2, 7.7 Hz, 1H, CH-CH_2_). ^13^C NMR (126 MHz, CDCl_3_) *δ* 159.5, 155.9, 147.1, 145.2, 143.7, 139.3, 126.8, 125.2, 124.7, 122.7, 119.0, 118.8, 115.4, 70.9, 68.5, 51.0, 48.4. HRMS (ESI) [M+H^+^] calcd for C_19_H_20_ClN_2_O_3_S: 391.0883, found: 391.0899.

**1-(4-((3-chloropyridin-2-yl)oxy)phenoxy)-3-((furan-2-ylmethyl)amino)propan-2-ol (PyE33)**

Yellow solid, yield 38.6%; ^1^H NMR (400 MHz, CDCl_3_) *δ* 8.00 (dd, *J* = 4.8, 1.6 Hz, 1H, pyridine-4-H), 7.73 (dd, *J* = 7.7, 1.6 Hz, 1H, pyridine-6-H), 7.37 (d, *J* = 0.7 Hz, 1H, furan-5-H), 7.09-7.05 (m, 2H, diphenyl-2,6-H), 6.95-6.91 (m, 3H, diphenyl-3,5-H & pyridine-5-H), 6.32-6.31 (m, 1H, furan-4-H), 6.20-6.19 (m, 1H, furan-3-H), 4.09-4.03 (m, 1H, CH-OH), 3.96 (d, *J* = 4.9 Hz, 2H, OCH_2_), 3.82 (s, 2H, CH_2_-furan), 2.88-2.73 (m, 2H, CH-CH_2_-NH). ^13^C NMR (126 MHz, CDCl_3_) *δ* 159.5, 155.9, 153.6, 147.1, 145.2, 142.1, 139.3, 122.7, 119.0, 118.8, 115.4, 110.3, 107.3, 70.9, 68.5, 51.1, 46.1. HRMS (ESI) [M+H^+^] calcd for C_19_H_20_ClN_2_O_4_: 375.1112, found: 375.1125.

# 3. Supplementary Tables and Figures

## 3.1 Results of ^1^H NMR Titration Experiments of PyE28

**Table S1.** Results of ^1^H NMR titration experiments.

| Chemicals | H_a_ | H_b_ | H_c_ | H_d_ |
| --- | --- | --- | --- | --- |
| PyE28 | 7.911 | 7.837 | 7.427 | 7.411 |
| HP-*β*-CD | / | / | / | / |
| PyE28:HP-*β*-CD=1:0.5 | 7.932 (*∆δ* = +0.021 ppm) | 7.895 (*∆δ* = +0.058 ppm) | 7.426 (*∆δ* = -0.002 ppm) | 7.411 (*∆δ* = +0.002 ppm) |
| PyE28:HP-*β*-CD=1:1.0 | 7.958 (*∆δ* = +0.047 ppm) | 7.939 (*∆δ* = +0.102 ppm) | 7.427 (∆*δ* = -0.002 ppm) | 7.414 (∆δ = +0.005 ppm) |
| PyE28:HP-*β*-CD=1:1.5 | 7.970 (∆*δ* = +0.059 ppm) | 7.955 (∆*δ* = +0.118 ppm) | 7.427 (∆*δ* = -0.002 ppm) | 7.416 (∆δ = +0.007 ppm) |
| Chemicals | H_e_ | H_f_ | H_g_ | H_i_ |
| PyE28 | 7.074 | 7.063 | 6.971 | 4.048 |
| HP-*β*-CD | / | / | / | / |
| PyE28:HP-*β*-CD=1:0.5 | 7.103 (*∆δ* = +0.029 ppm) | 7.037 (*∆δ* = -0.026 ppm) | 6.967 (*∆δ* = -0.004 ppm) | 4.046 (*∆δ* = -0.002 ppm) |
| PyE28:HP-*β*-CD=1:1.0 | 7.133 (∆*δ* = +0.059 ppm) | 7.021 (∆*δ* = -0.042 ppm) | 6.966 (∆*δ* = -0.005 ppm) | 4.036 (∆*δ* = -0.012 ppm) |
| PyE28:HP-*β*-CD=1:1.5 | 7.153 (∆*δ* = +0.079 ppm) | 7.010 (∆*δ* = -0.053 ppm) | 6.964 (∆*δ* = -0.007 ppm) | 4.029 (∆*δ* = -0.019 ppm) |

## 3.2 Sequencing Data and Alignment Results to the Reference Genome

**Table S2.** Summary of Sequencing Reads and Alignment to the Selected Reference Genome.

| Sample | Total Reads | Mapped Reads |
| --- | --- | --- |
| PyE28@*β*-CD (replicate 1) | 15,308,982 | 15,218,911 (99.41%) |
| PyE28@*β*-CD (replicate 2) | 18,153,464 | 18,006,168 (99.19%) |
| PyE28@*β*-CD (replicate 3) | 20,585,378 | 20,455,040 (99.37%) |
| Control (replicate 1) | 17,209,878 | 17,028,391 (98.95%) |
| Control (replicate 2) | 16,692,622 | 16,512,895 (98.92%) |
| Control (replicate 3) | 16,664,074 | 16,460,986 (98.78%) |

**Table S3.** Detailed information for the Top 20 upregulated GO terms (Biological Process, BP) with the smallest *q*-values.

| No. | GO.ID | Term description | q-value | Contained |
| --- | --- | --- | --- | --- |
| 1 | GO:0000105 | histidine biosynthetic process | 8.59E-04 | up |
| 2 | GO:0015628 | protein secretion by the type II secretion system | 4.27E-02 | down |
| 3 | GO:0006412 | translation | 4.88E-02 | up |
| 4 | GO:0006817 | phosphate ion transport | 8.95E-02 | up&down |
| 5 | GO:0035435 | phosphate ion transmembrane transport | 8.95E-02 | down |
| 6 | GO:0007165 | signal transduction | 1.18E-01 | up&down |
| 7 | GO:0009088 | threonine biosynthetic process | 2.66E-01 | up |
| 8 | GO:0042773 | ATP synthesis coupled electron transport | 2.79E-01 | up |
| 9 | GO:0009405 | pathogenesis | 2.79E-01 | down |
| 10 | GO:0006935 | chemotaxis | 2.79E-01 | up&down |
| 11 | GO:0045493 | xylan catabolic process | 2.79E-01 | up&down |
| 12 | GO:0006066 | alcohol metabolic process | 2.79E-01 | down |
| 13 | GO:0015990 | electron transport coupled proton transport | 2.79E-01 | up&down |
| 14 | GO:0016036 | cellular response to phosphate starvation | 2.79E-01 | down |
| 15 | GO:0019290 | siderophore biosynthetic process | 2.79E-01 | down |
| 16 | GO:0019751 | polyol metabolic process | 2.79E-01 | down |
| 17 | GO:0030259 | lipid glycosylation | 2.79E-01 | up&down |
| 18 | GO:0031667 | response to nutrient levels | 2.79E-01 | down |
| 19 | GO:0031669 | cellular response to nutrient levels | 2.79E-01 | down |
| 20 | GO:0033517 | myo-inositol hexakisphosphate metabolic process | 2.79E-01 | down |

**Table S4.** Detailed information for the Top 20 upregulated GO terms (Cellular Component, CC) with the smallest *q*-values.

| No. | GO.ID | Term description | q-value | Contained |
| --- | --- | --- | --- | --- |
| 1 | GO:0009279 | cell outer membrane | 7.28E-04 | up&down |
| 2 | GO:0005840 | ribosome | 7.39E-03 | up |
| 3 | GO:0005576 | extracellular region | 7.39E-03 | up&down |
| 4 | GO:0015627 | type II protein secretion system complex | 8.87E-03 | down |
| 5 | GO:0009295 | nucleoid | 1.04E-01 | up |
| 6 | GO:0019867 | outer membrane | 1.82E-01 | up&down |
| 7 | GO:0015934 | large ribosomal subunit | 3.27E-01 | up |
| 8 | GO:0032153 | cell division site | 4.44E-01 | up |
| 9 | GO:0070069 | cytochrome complex | 5.64E-01 | up&down |
| 10 | GO:0005960 | glycine cleavage complex | 5.64E-01 | up |
| 11 | GO:0009276 | Gram-negative-bacterium-type cell wall | 5.64E-01 | down |
| 12 | GO:0009358 | polyphosphate kinase complex | 5.64E-01 | down |
| 13 | GO:0009420 | bacterial-type flagellum filament | 5.64E-01 | up |
| 14 | GO:0030256 | type I protein secretion system complex | 5.64E-01 | down |
| 15 | GO:0045239 | tricarboxylic acid cycle enzyme complex | 5.64E-01 | down |
| 16 | GO:0045277 | respiratory chain complex IV | 5.64E-01 | down |
| 17 | GO:0046930 | pore complex | 5.64E-01 | down |
| 18 | GO:0005887 | integral component of plasma membrane | 5.71E-01 | up&down |
| 19 | GO:0030257 | type III protein secretion system complex | 8.91E-01 | down |
| 20 | GO:0009289 | pilus | 9.10E-01 | down |

**Table S5.** Detailed information for the Top 20 upregulated GO terms (Molecular Function, MF) with the smallest *q*-values.

| No. | GO.ID | Term description | q-value | Contained |
| --- | --- | --- | --- | --- |
| 1 | GO:0003735 | structural constituent of ribosome | 6.60E-02 | up |
| 2 | GO:0005315 | inorganic phosphate transmembrane transporter activity | 1.18E-01 | up&down |
| 3 | GO:0000150 | recombinase activity | 1.18E-01 | down |
| 4 | GO:0008081 | phosphoric diester hydrolase activity | 1.18E-01 | down |
| 5 | GO:0015344 | siderophore uptake transmembrane transporter activity | 1.18E-01 | down |
| 6 | GO:0038023 | signaling receptor activity | 1.18E-01 | down |
| 7 | GO:0051537 | 2 iron, 2 sulfur cluster binding | 2.21E-01 | up&down |
| 8 | GO:0050136 | NADH dehydrogenase (quinone) activity | 2.80E-01 | up |
| 9 | GO:0050568 | protein-glutamine glutaminase activity | 2.88E-01 | up |
| 10 | GO:0042803 | protein homodimerization activity | 4.14E-01 | up&down |
| 11 | GO:0004888 | transmembrane signaling receptor activity | 4.14E-01 | up |
| 12 | GO:0008236 | serine-type peptidase activity | 4.14E-01 | up |
| 13 | GO:0051082 | unfolded protein binding | 4.14E-01 | up&down |
| 14 | GO:0004803 | transposase activity | 4.14E-01 | up&down |
| 15 | GO:0004072 | aspartate kinase activity | 4.14E-01 | up |
| 16 | GO:0004317 | 3-hydroxypalmitoyl-[acyl-carrier-protein] dehydratase activity | 4.14E-01 | up |
| 17 | GO:0008252 | nucleotidase activity | 4.14E-01 | down |
| 18 | GO:0008659 | (3R)-hydroxymyristoyl-[acyl-carrier-protein] dehydratase activity | 4.14E-01 | up |
| 19 | GO:0030257 | 3-hydroxydecanoyl-[acyl-carrier-protein] dehydratase activity | 4.14E-01 | up |
| 20 | GO:0009289 | carbohydrate phosphatase activity | 4.14E-01 | down |

**Table S6.** Detailed information for the Top 20 upregulated KEGG terms with the smallest *q*-values.

| No. | KEGG.ID | Term description | q-value | Contained |
| --- | --- | --- | --- | --- |
| 1 | ko03010 | Ribosome | 3.18E-03 | up |
| 2 | ko02030 | Bacterial chemotaxis | 2.16E-02 | up&down |
| 3 | ko04626 | Plant-pathogen interaction | 6.26E-02 | down |
| 4 | ko00190 | Oxidative phosphorylation | 3.93E-01 | up&down |
| 5 | ko03070 | Bacterial secretion system | 4.35E-01 | up&down |
| 6 | ko00340 | Histidine metabolism | 4.35E-01 | up&down |
| 7 | ko00997 | Biosynthesis of various secondary metabolites - part 3 | 5.33E-01 | down |
| 8 | ko03450 | Non-homologous end-joining | 5.33E-01 | down |
| 9 | ko00562 | Inositol phosphate metabolism | 6.37E-01 | down |
| 10 | ko00401 | Novobiocin biosynthesis | 6.37E-01 | up&down |
| 11 | ko00471 | D-Glutamine and D-glutamate metabolism | 8.94E-01 | up |
| 12 | ko01503 | Cationic antimicrobial peptide (CAMP) resistance | 8.94E-01 | up&down |
| 13 | ko00564 | Glycerophospholipid metabolism | 8.94E-01 | up&down |
| 14 | ko00261 | Monobactam biosynthesis | 8.94E-01 | up |
| 15 | ko02060 | Phosphotransferase system (PTS) | 8.94E-01 | down |
| 16 | ko00500 | Starch and sucrose metabolism | 8.94E-01 | up&down |
| 17 | ko00350 | Tyrosine metabolism | 8.94E-01 | up&down |
| 18 | ko00565 | Ether lipid metabolism | 8.94E-01 | down |
| 19 | ko00232 | Caffeine metabolism | 8.94E-01 | down |
| 20 | ko01053 | Biosynthesis of siderophore group nonribosomal peptides | 8.94E-01 | down |

**Table S7.** COG functional classification of differentially expressed genes.

| **COG ID** | **Class name** | **Number of genes** |
| --- | --- | --- |
| J | Translation, ribosomal structure and biogenesis | 46 |
| A | RNA processing and modification | 0 |
| K | Transcription | 47 |
| L | Replication, recombination and repair | 36 |
| B | Chromatin structure and dynamics | 0 |
| D | Cell cycle control, cell division, chromosome partitioning | 11 |
| Y | Nuclear structure | 0 |
| V | Defense mechanisms | 28 |
| T | Signal transduction mechanisms | 62 |
| M | Cell wall/membrane/envelope biogenesis | 61 |
| N | Cell motility | 39 |
| Z | Cytoskeleton | 0 |
| W | Extracellular structures | 15 |
| U | Intracellular trafficking, secretion, and vesicular transport | 35 |
| O | Posttranslational modification, protein turnover, chaperones | 43 |
| C | Energy production and conversion | 48 |
| G | Carbohydrate transport and metabolism | 45 |
| E | Amino acid transport and metabolism | 59 |
| F | Nucleotide transport and metabolism | 9 |
| H | Coenzyme transport and metabolism | 26 |
| I | Lipid transport and metabolism | 36 |
| P | Inorganic ion transport and metabolism | 83 |
| Q | Secondary metabolites biosynthesis, transport and catabolism | 18 |
| R | General function prediction only | 82 |
| S | Function unknown | 44 |
| X | Mobilome: prophages, transposons | 31 |

## 3.3 *In Vitro* Antibacterial Activity of Compounds against *Xoo*

**Table S8.** EC_50_ values of compounds PyE1-PyE33 against *Xoo in vitro*.

| Compd. | Regression equation | EC_50_  (*μ*g mL^-1^) | Compd. | Regression equation | EC_50_  (*μ*g mL^-1^) |
| --- | --- | --- | --- | --- | --- |
| PyE1 | y = 0.4152x + 4.783 | 3.33 ± 0.18 | PyE18 | / | > 100 |
| PyE2 | / | > 50 | PyE19 | / | > 100 |
| PyE3 | / | > 100 | PyE20 | / | > 100 |
| PyE4 | / | > 50 | PyE21 | y = 0.986x + 4.335 | 4.72 ± 0.17 |
| PyE5 | y = 0.983x + 4.245 | 5.86 ± 0.19 | PyE22 | / | > 100 |
| PyE6 | / | > 50 | PyE23 | y = 1.8274x + 2.2953 | 30.2 ± 0.3 |
| PyE7 | y = 1.27x + 3.9933 | 6.20 ± 0.26 | PyE24 | / | > 100 |
| PyE8 | y = 2.022x + 3.587 | 5.00 ± 0.07 | PyE25 | / | > 100 |
| PyE9 | / | > 100 | PyE26 | / | > 100 |
| PyE10 | / | > 100 | PyE27 | / | > 50 |
| PyE11 | / | > 50 | PyE28 | y = 3.4420x + 3.9407 | 2.03 ± 0.09 |
| PyE12 | / | > 50 | PyE29 | / | > 100 |
| PyE13 | y=4.344x - 2.3136 | 48.3 ± 0.9 | PyE30 | / | > 100 |
| PyE14 | / | > 100 | PyE31 | / | > 100 |
| PyE15 | / | > 100 | PyE32 | y = 1.2627x + 4.4355 | 2.80 ± 0.90 |
| PyE16 | / | > 100 | PyE33 | y = 1.585x + 3.292 | 12.0 ± 0.4 |
| PyE17 | / | > 100 | TC | y = 2.842x - 0.363 | 77.1 ± 1.7 |

Note: EC_50_ values of antibacterial activities are indicated as means ± SD (standard deviation); Commercialized bactericide as the positive control, abbreviation: TC, thiodiazole-copper.

## 3.4 HRMS Spectrum of the Supramolecular Complex PyE28@HP-*β*-CD

**
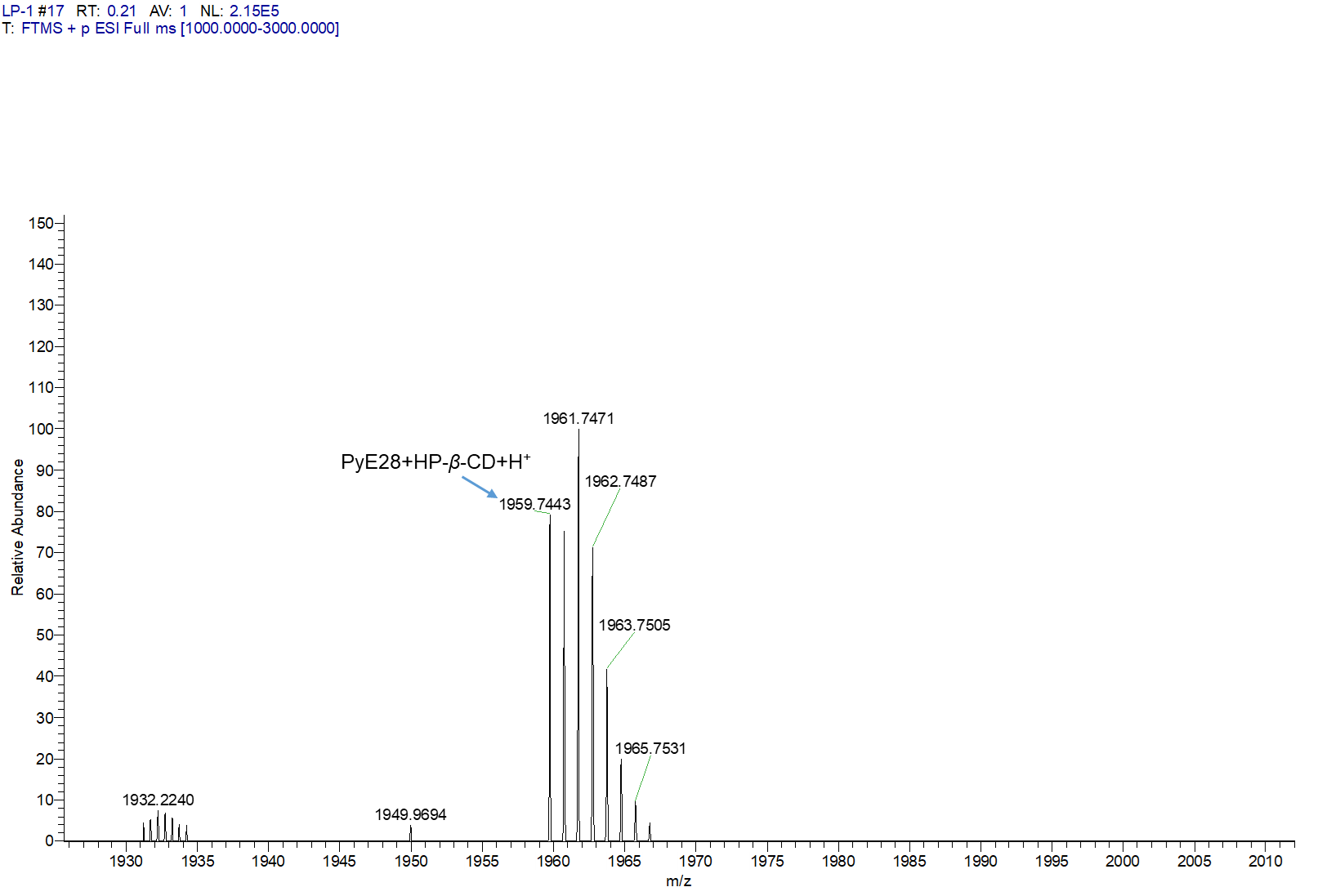
**

**Figure S1.** HRMS Spectrum of compound PyE28@HP-*β*-CD.

## 3.5 Assembly Mechanisms of PyE28

**
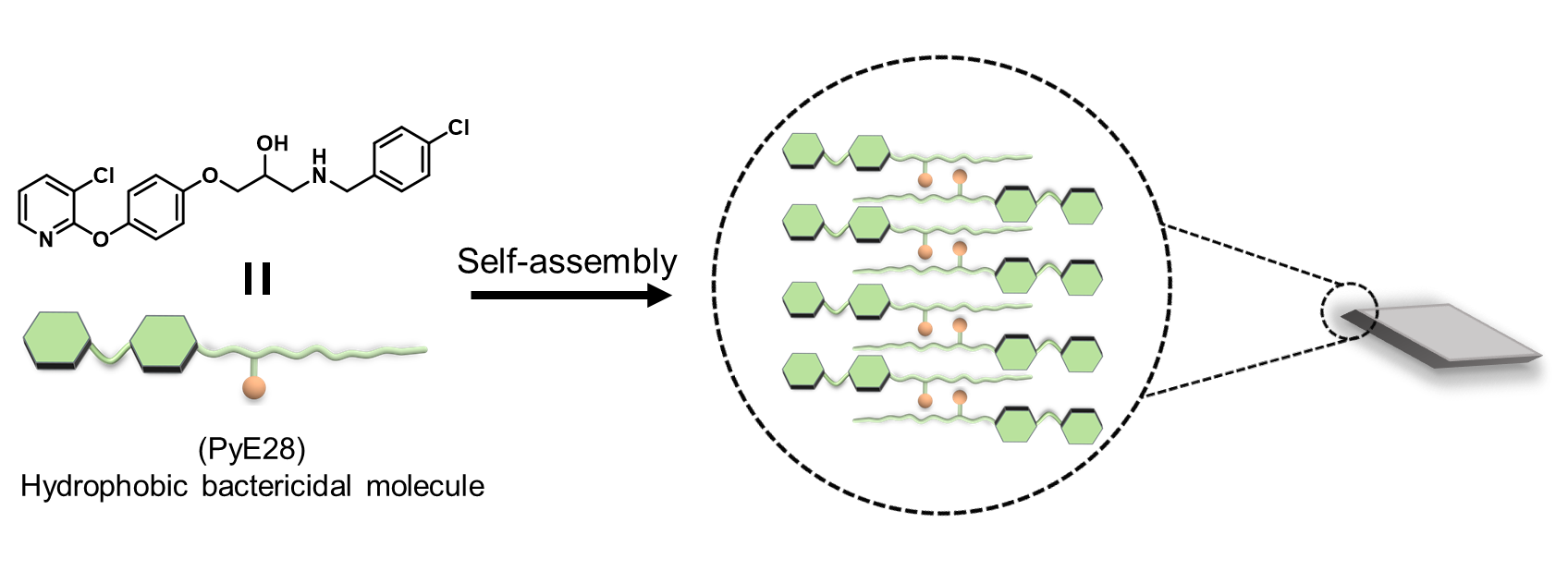
**

**Figure S2.** Self-assembly diagram of PyE28.

## 3.6 HPLC Calibration Curve of PyE28


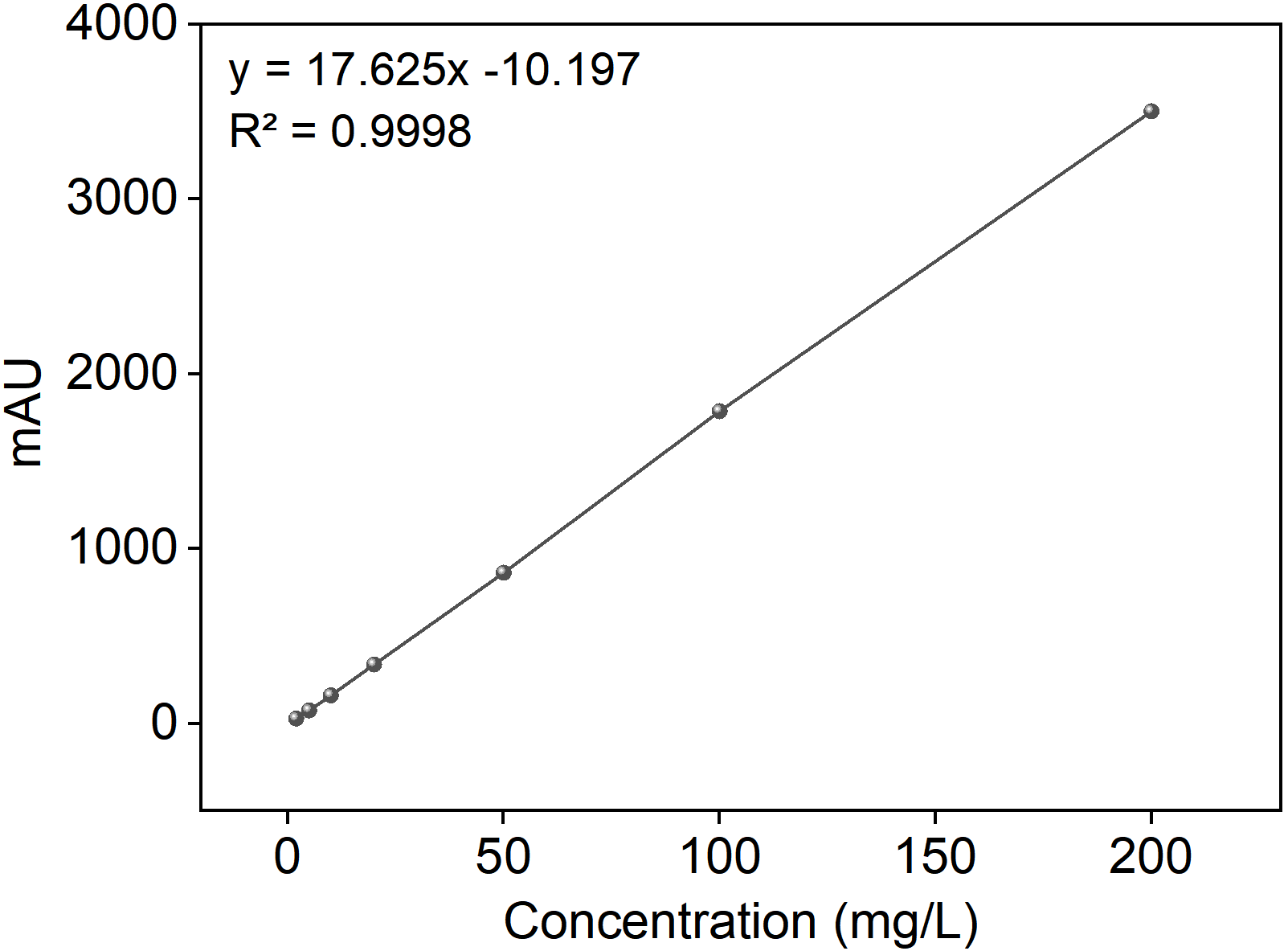


**Figure S3.** The standard curve of compound PyE28 at different concentrations (200, 100, 50, 20, 10, 5 and 2 *μ*g/mL).

## 3.7 Stability of PyE28@HP-*β*-CD


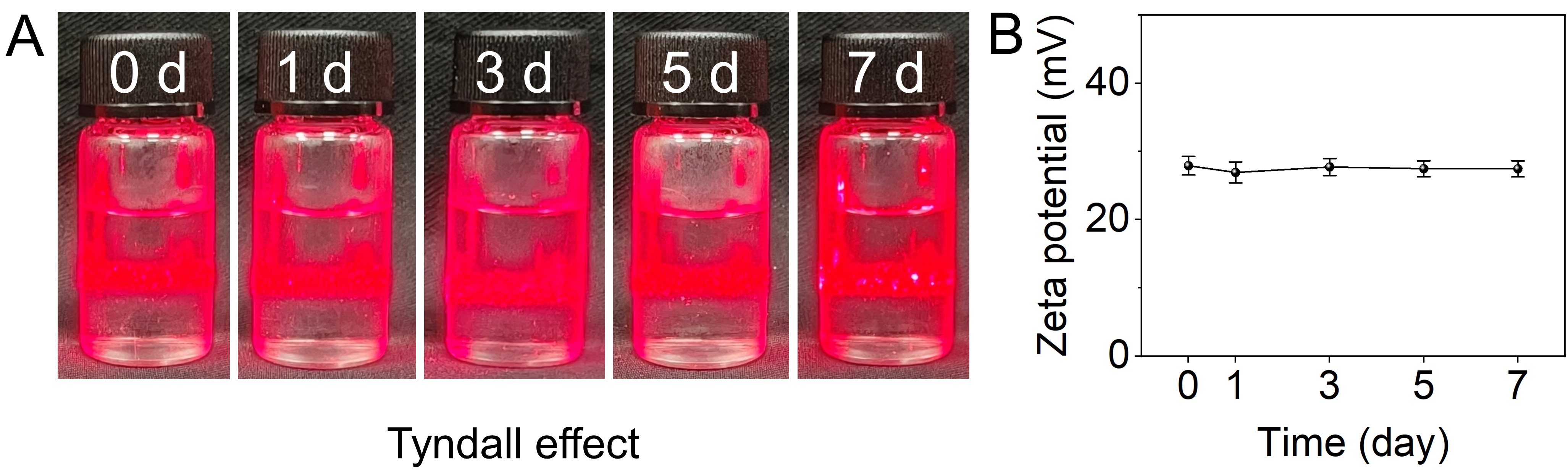


**Figure S4.** Stability of the PyE28@HP-*β*-CD system at an effective concentration of 200 *μ*g mL^-1^. (A) Tyndall effect images of PyE28@HP-*β*-CD aqueous dispersion recorded at 0, 1, 3, 5, and 7 days. (B) Zeta potential of PyE28@HP-*β*-CD aqueous dispersion measured at 0, 1, 3, 5, and 7 days.

## 3.8 Rain Wash-Off Resistance of PyE28@HP-*β*-CD on Rice Leaves


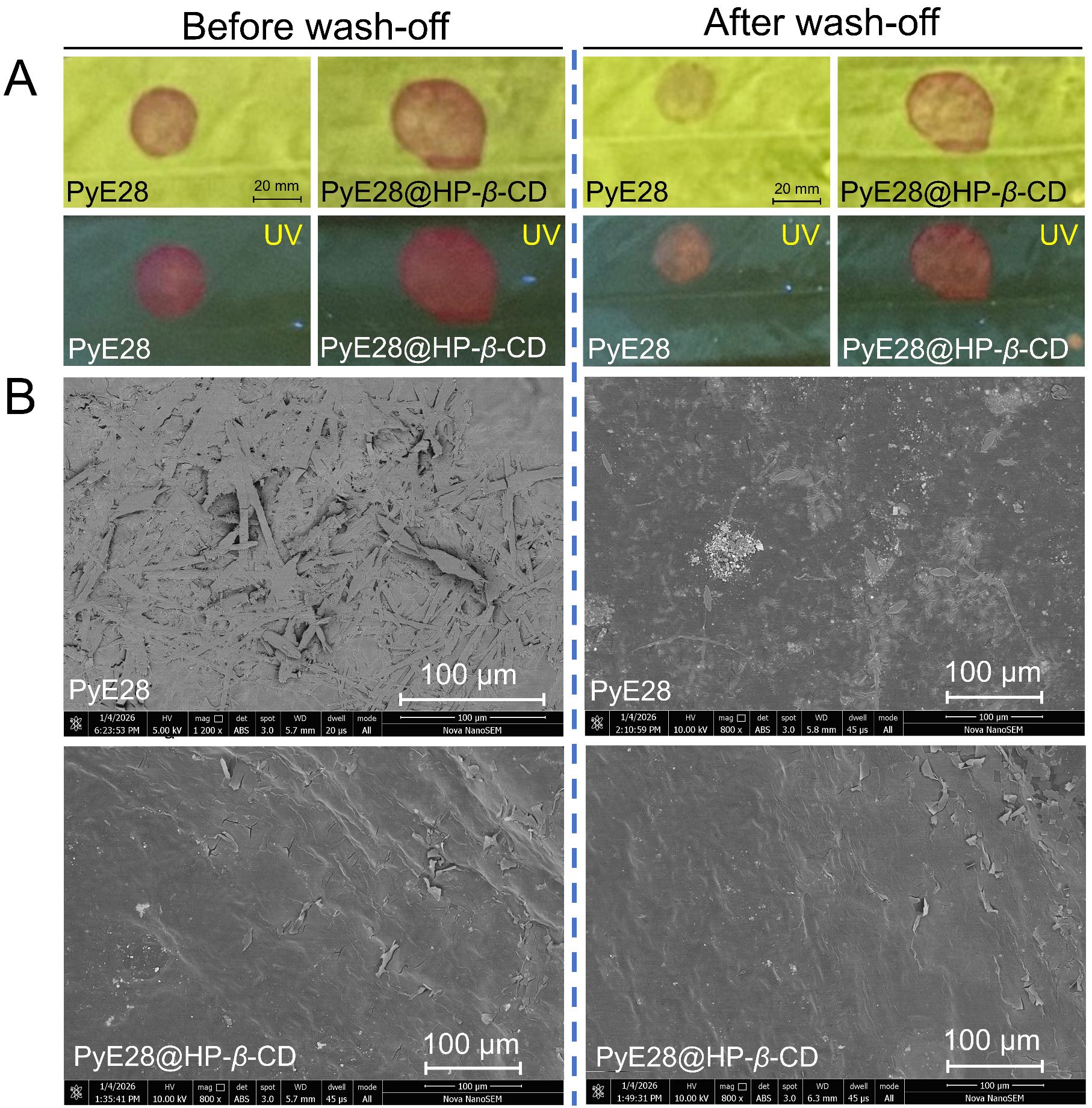


**Figure S5.** Rain wash-off resistance of PyE28 and PyE28@HP-*β*-CD on citrus leaves.

(A) Optical photographs of droplets (10 *μ*L, 200 *μ*g mL^-1^) of PyE28 or PyE28@HP-*β*-CD, mixed with 0.1% Rhodamine 6G, deposited on citrus leaves before/after UV irradiation and before/after simulated rain wash-off. (B) SEM images of citrus leaf surfaces treated with PyE28 or PyE28@HP-*β*-CD (200 *μ*g mL^-1^, 10 *μ*L) before and after simulated rain wash-off.

## 3.9 Growth Curve of *Xac*

**
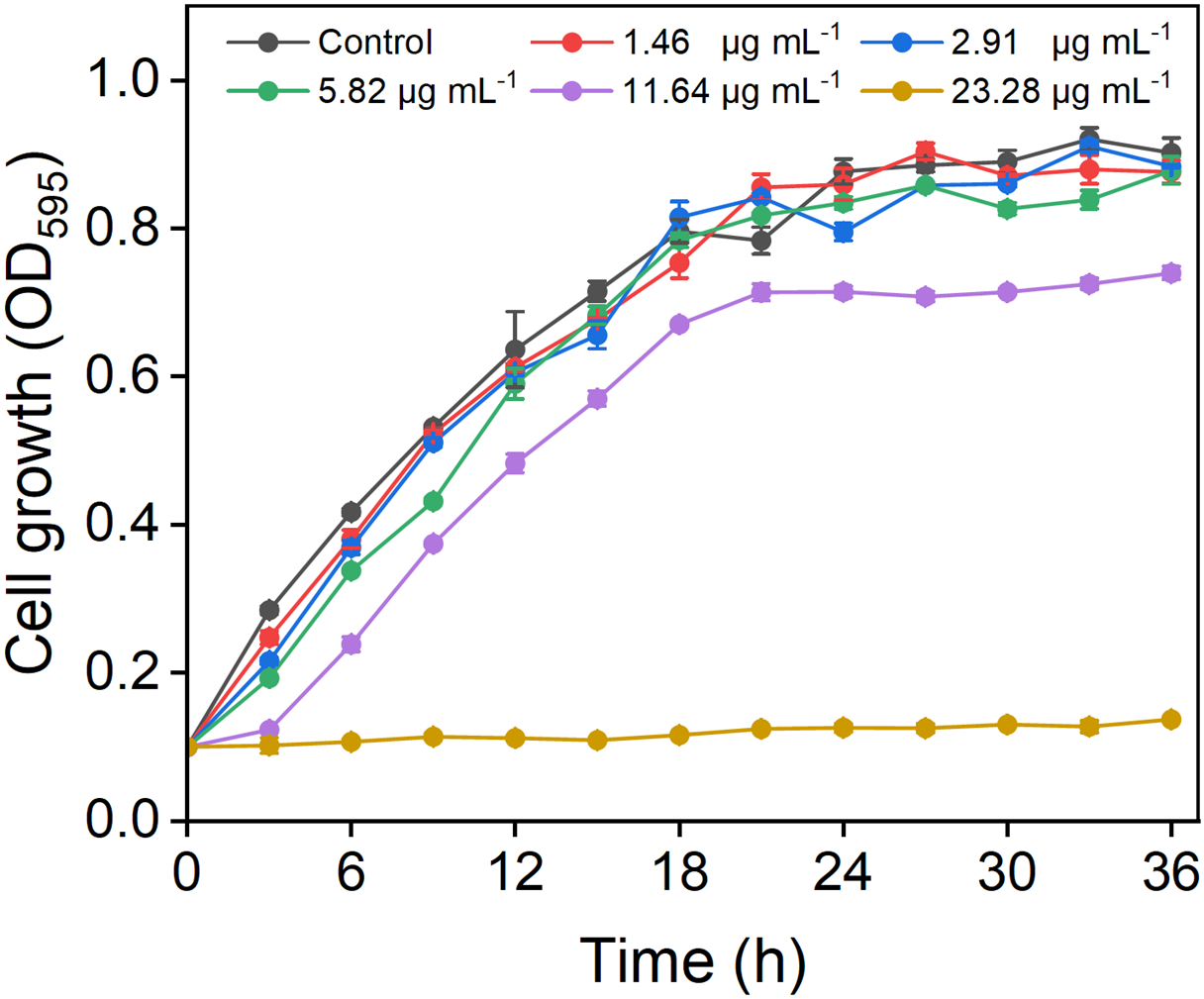
**

**Figure S6.** The growth curves of *Xac* (initial OD_595 nm_ = 0.1) triggered by compound PyE28 at concentrations of 1.46, 2.91, 5.82, 11.64, and 23.28 *μ*g mL^-1^.

## 3.10 Analysis of Biofilm Inhibition Rate Using the Agar Diffusion Method


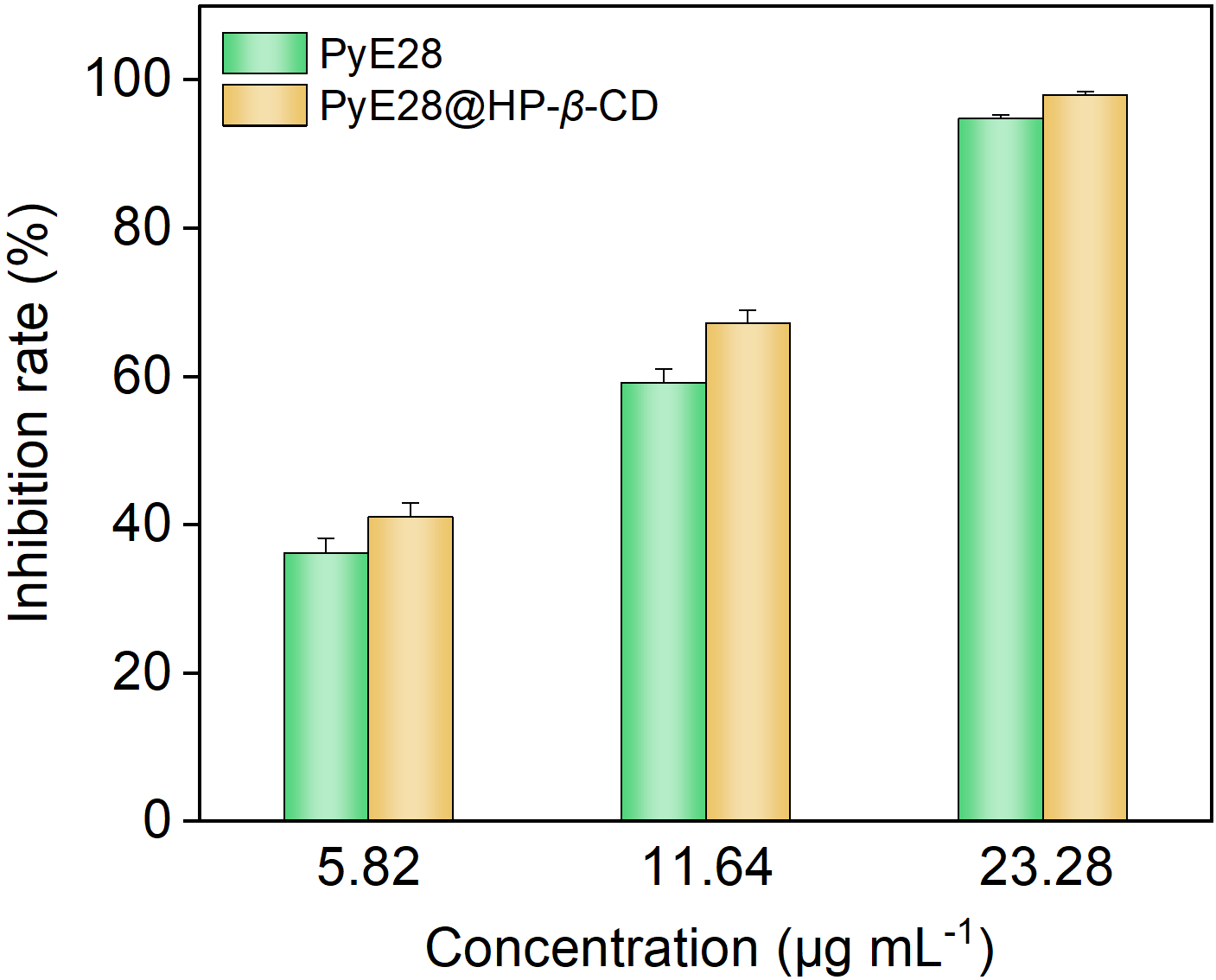


**Figure S7.** Inhibition rates of PyE28 and PyE28@HP-*β*-CD against *Xac* at different concentrations. The inhibition rates were calculated based on colony counts from plate assays (for Figure 3E).

## 3.11 Analysis of Biofilm Inhibition Rate Using the CLSM

**
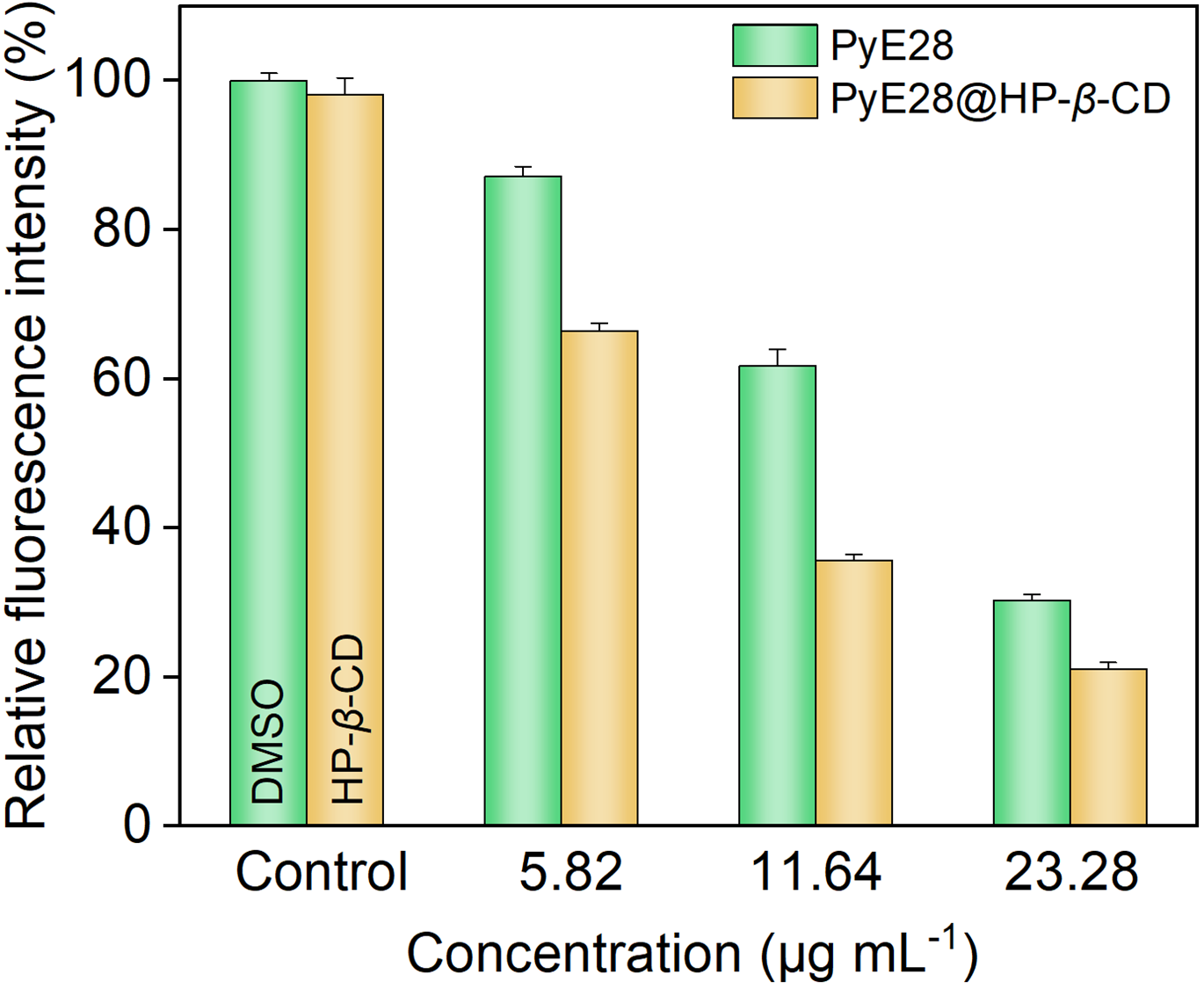
**

**Figure S8.** The statistically averaged fluorescence intensity (for Figure 3F) via image-J software for indicating the biofilm inhibition effect from above CLSM 3D images.

## 3.12 Impact of PyE28@HP-*β*-CD on Extracellular Polysaccharide Production


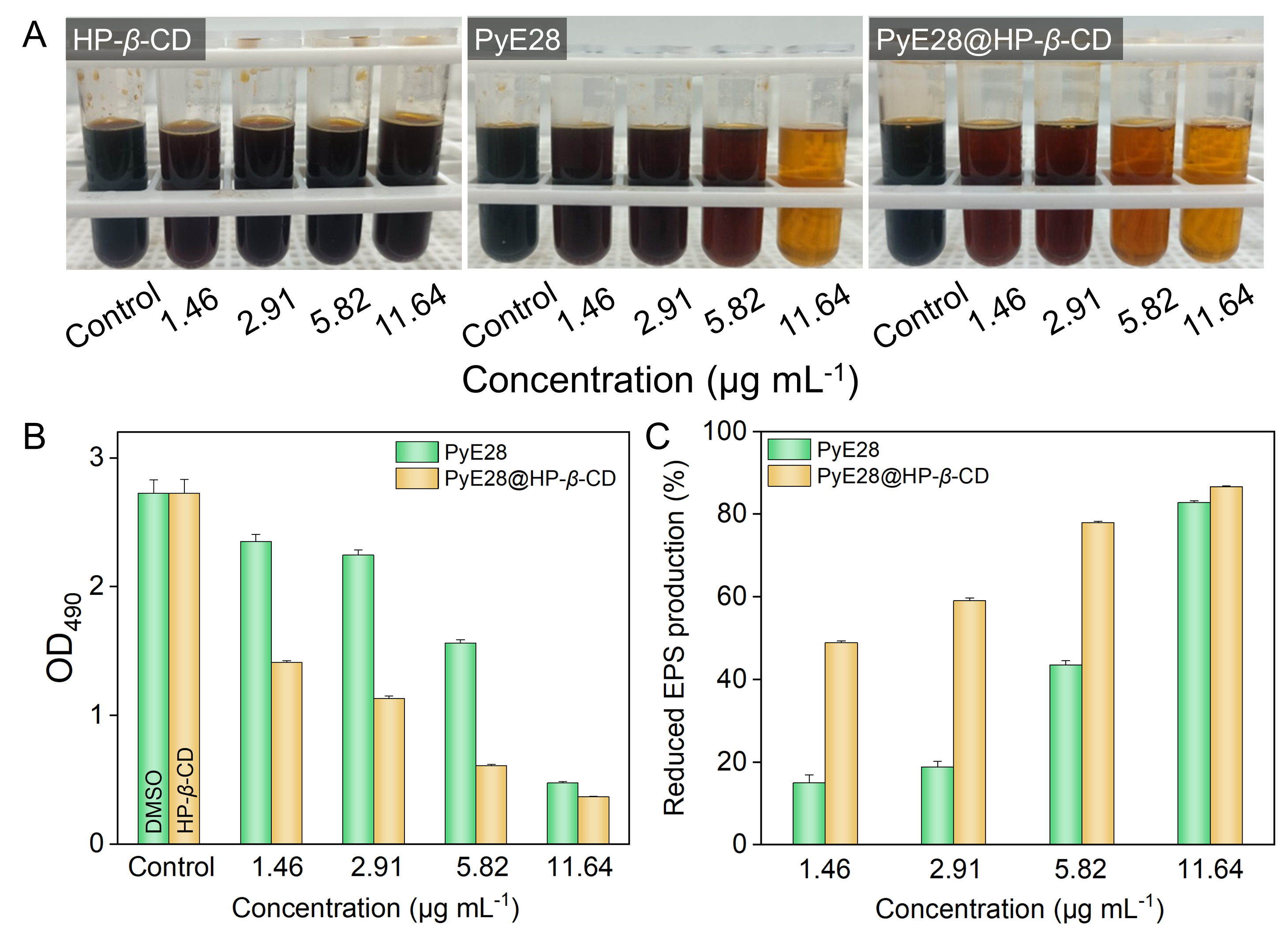


**Figure S9.** Biofilm extracellular polysaccharide (EPS) analysis. (A) Photographic representation of biofilm formation under the treatment of HP-*β*-CD, PyE28, and PyE28@HP-*β*-CD at concentrations of 1.46, 2.91, 5.82, and 11.64 *μ*g mL^-1^. (B) Quantitative measurement of EPS production expressed as OD_490 nm_ absorbance. (C) Calculated inhibition rates of EPS production under the same treatments and concentrations.

## 3.13 Analysis of Biofilm Eradication Rate Using the CLSM

**
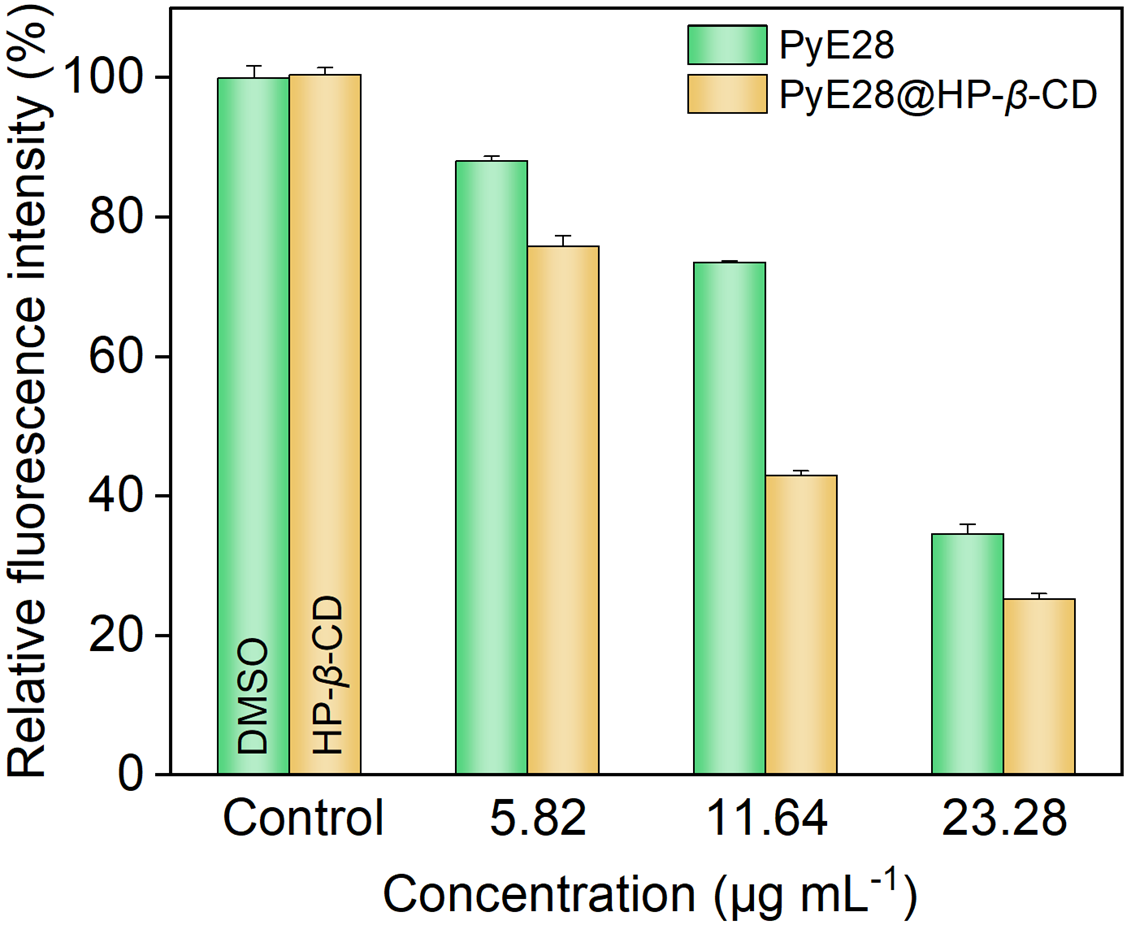
**

**Figure S10.** Statistical fluorescence intensities of *Xac* biofilms treated by PyE28 and PyE28@HP-*β*-CD at concentrations of 5.82, 11.64 and 23.28 *μ*g mL^-1^.

## 3.14 Differentially Expressed Genes (DEGs) Analysis


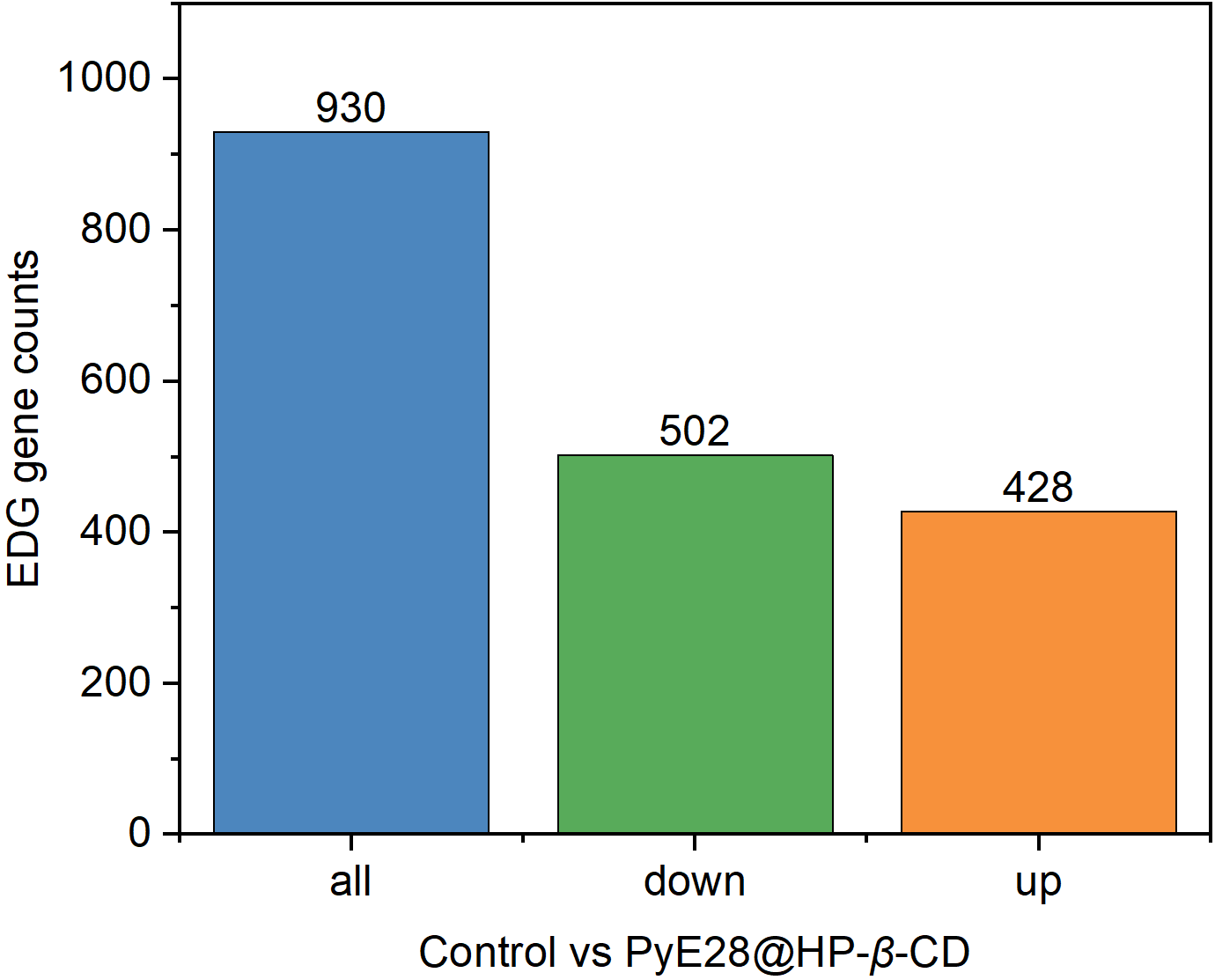


**Figure S11.** Bar chart of differentially expressed gene (DEG) statistics.

## 3.15 The Possible Bactericidal Mechanisms


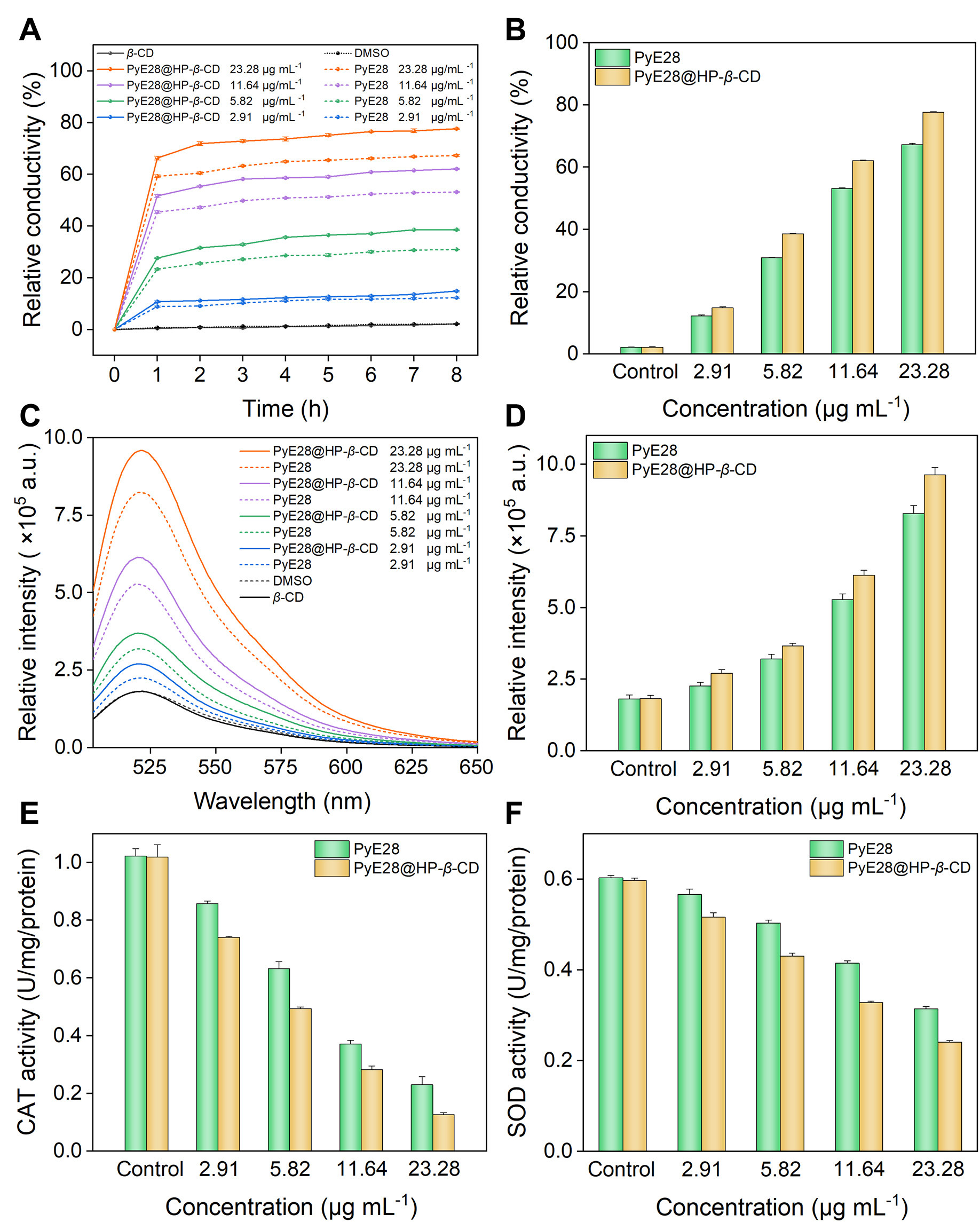


**Figure S12.** The possible bactericidal mechanism of PyE28@HP-*β*-CD. A) The relative electrical conductivity of *Xac* treated with different doses (0, 2.91, 5.82, 11.64, and 23.28 *μ*g mL^-1^) of PyE28 and PyE28@HP-*β*-CD. B) The relative conductivity at the eighth hour. C) ROS accumulation in *Xac* monitored by the ROS detection kit after co-incubation with different concentrations (0, 2.91, 5.82, 11.64, and 23.28 *μ*g mL^-1^) of PyE28 and PyE28@HP-*β*-CD, Ex = 488 nm. D) The fluorescence intensity at 520 nm reflecting the amount of ROS. E) The CAT activity in *Xac* strains affected by different concentrations of PyE28@HP-*β*-CD and PyE28. F) The SOD activity in *Xac* strains affected by different concentrations of PyE28@HP-*β*-CD and PyE28.

## 3.16 Drop Behavior on Inclined Rice Leaf with PyE28@HP-*β*-CD


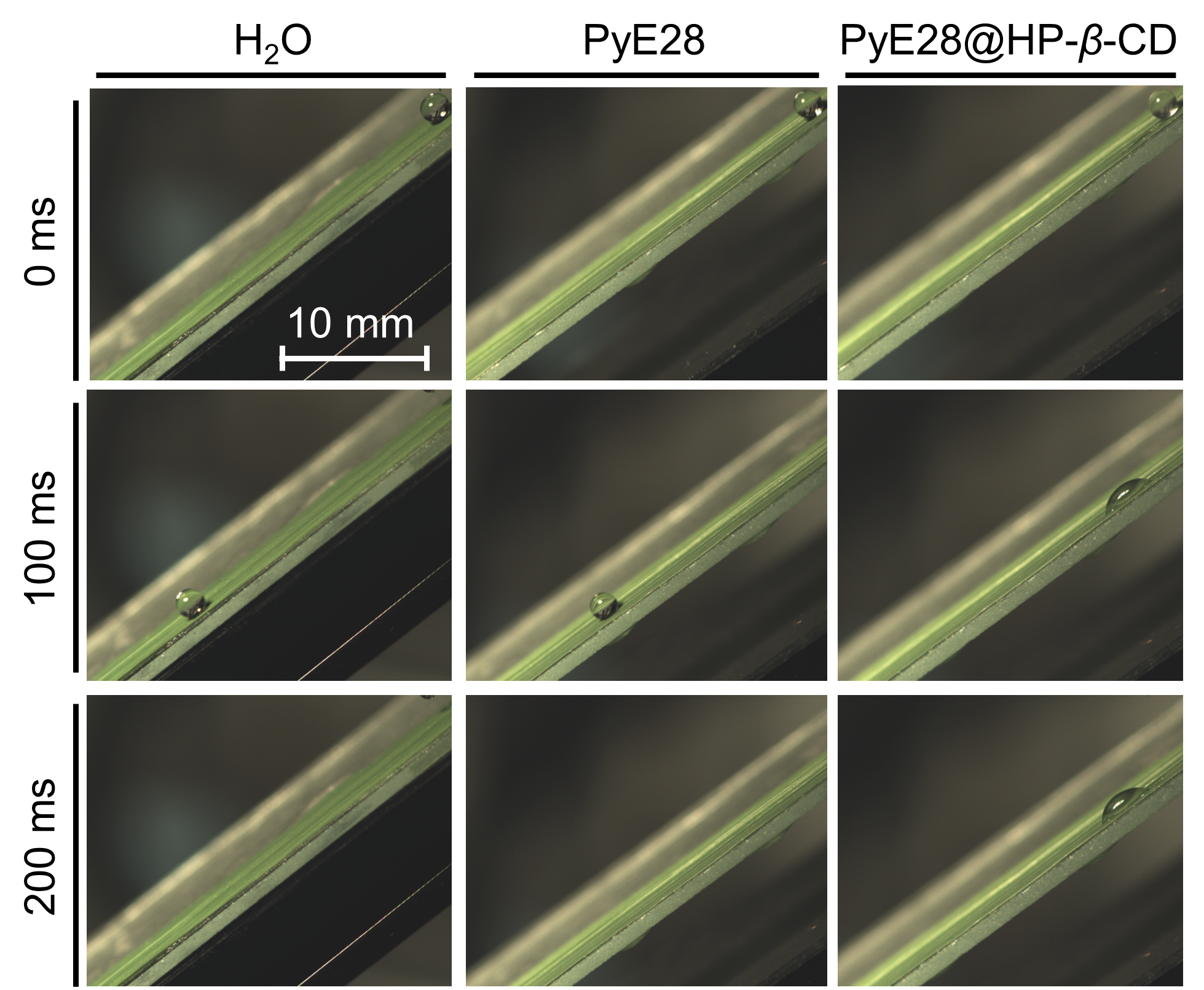


**Figure S13.** Representative time-lapse optical images showing the sliding behavior of droplets (10 *μ*L) of water, PyE28, and PyE28@HP-*β*-CD (200 *μ*g/mL^-1^) on rice leaves at a tilt angle of 45°. Scale bar: 10 mm.

## 3.17 Liquid-Holding Capacity of the Formulations on Rice Leaves


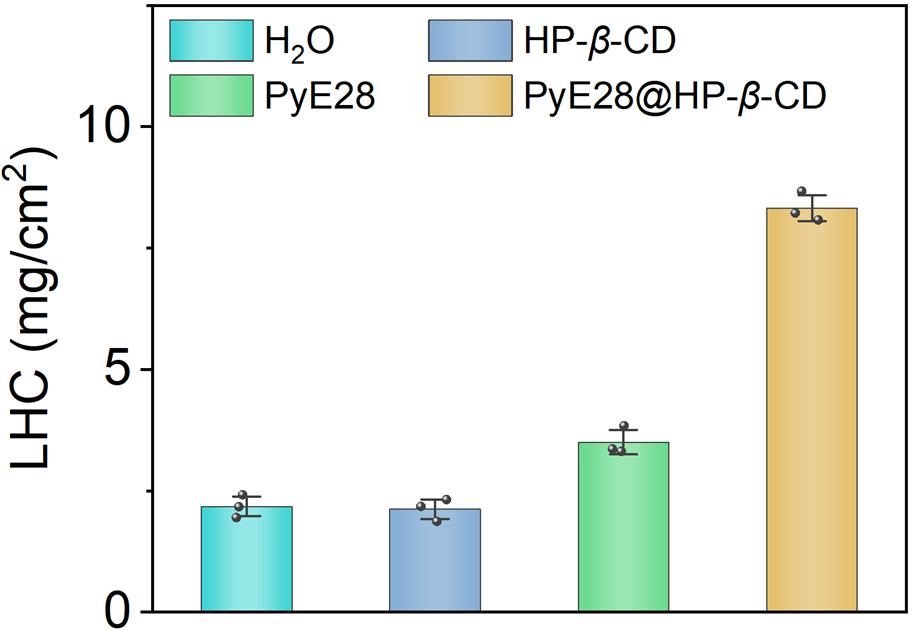


**Figure S14.** Liquid holding capacities (LHC) of rice leaves after immersion in H_2_O, HP-*β*-CD, PyE28, and PyE28@*β*-CD at an effective concentration of 200 *μ*g mL^-1^.

## 3.18 Biosafety Evaluation


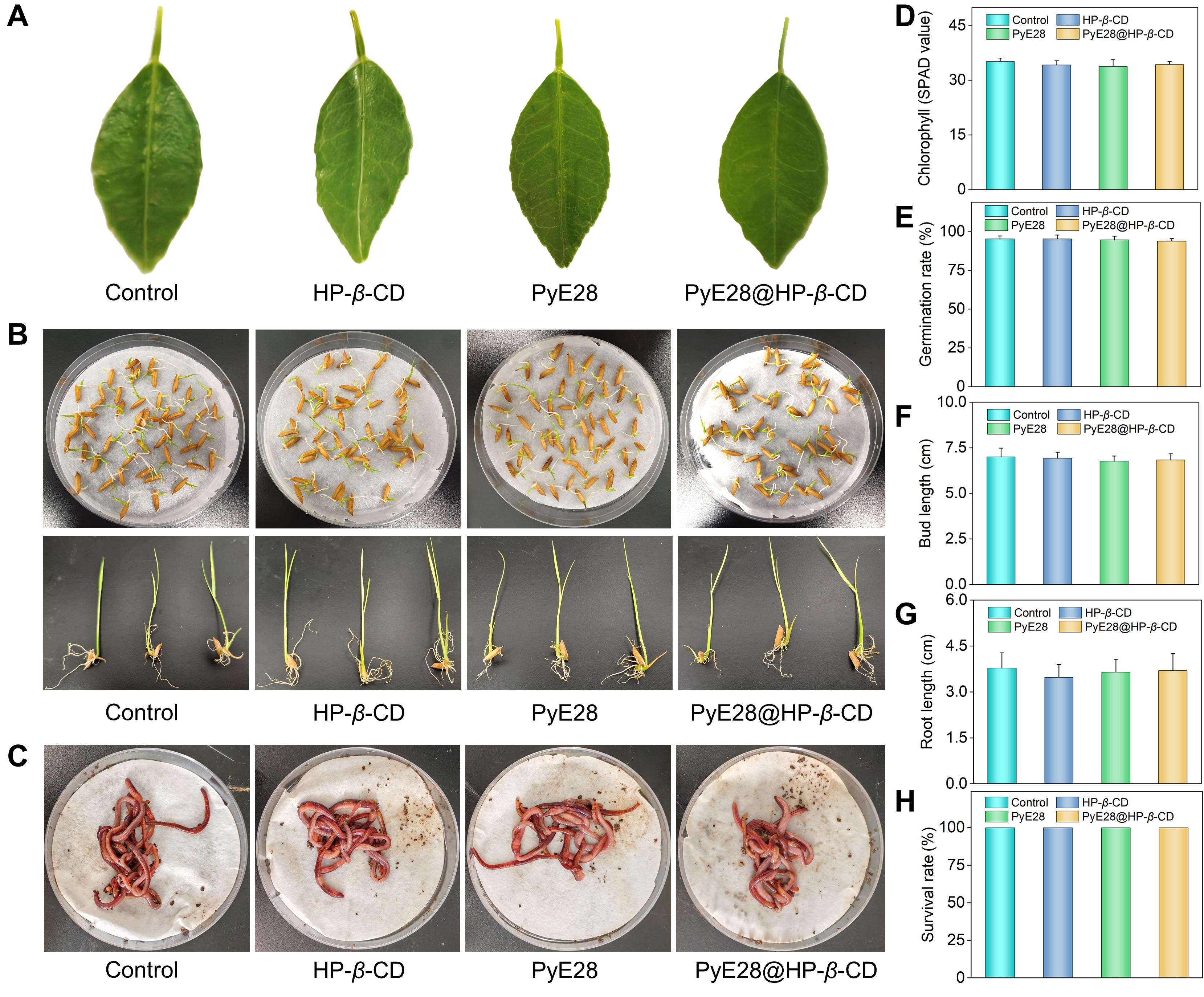


**Figure S15.** Biosafety assessment of PyE28@HP-*β*-CD. (A, D) Effects of PyE28@HP-*β*-CD (200 *μ*g mL^-1^) on phytotoxicity and chlorophyll content in citrus leaves. (B, E) Effects of PyE28@HP-*β*-CD (200 *μ*g mL^-1^) on rice seed germination rate. (F, G) Effects of PyE28@HP-*β*-CD (200 *μ*g mL^-1^) on rice shoot and root lengths. (C, H) Acute toxicity of PyE28@HP-*β*-CD (C_PyE28_ = 10.0 *μ*g mL^-1^) on earthworms. Statistical analysis was conducted using an independent-samples t-test in (D-H), and significance levels are indicated as *p* < 0.05 (*), *p* < 0.01 (**), *p* < 0.001 (***), and not significant (ns), with *n* ≥ 3 for all experiments.

## 3.19 ^1^H NMR, ^13^C NMR, ^19^F NMR and HRMS Spectra of Target Compounds

**
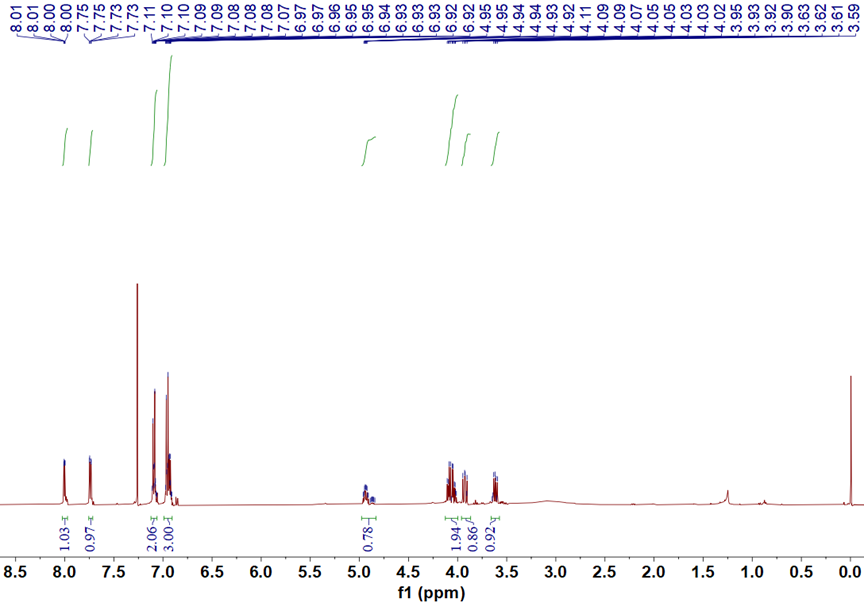
**

**Figure S16.** ^1^H NMR Spectrum (CDCl_3_, 500 MHz) of compound PyE1.

**
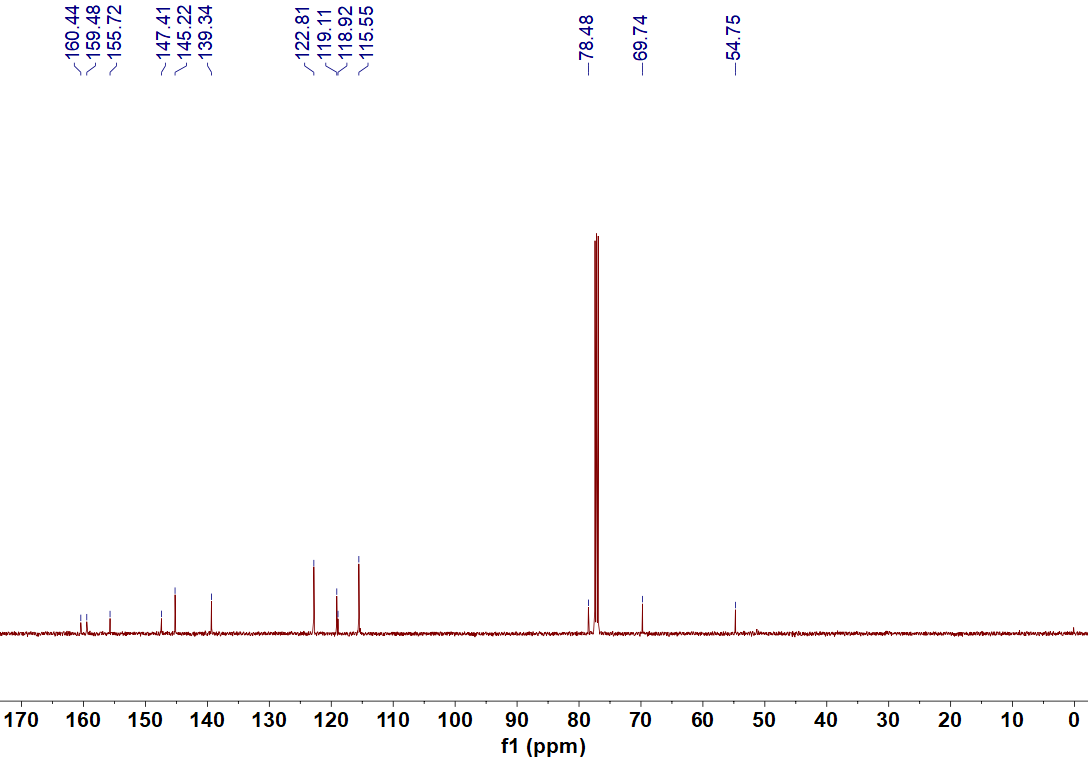
Figure S17.** ^13^C NMR Spectrum (CDCl_3_, 126 MHz) of compound PyE1.

**
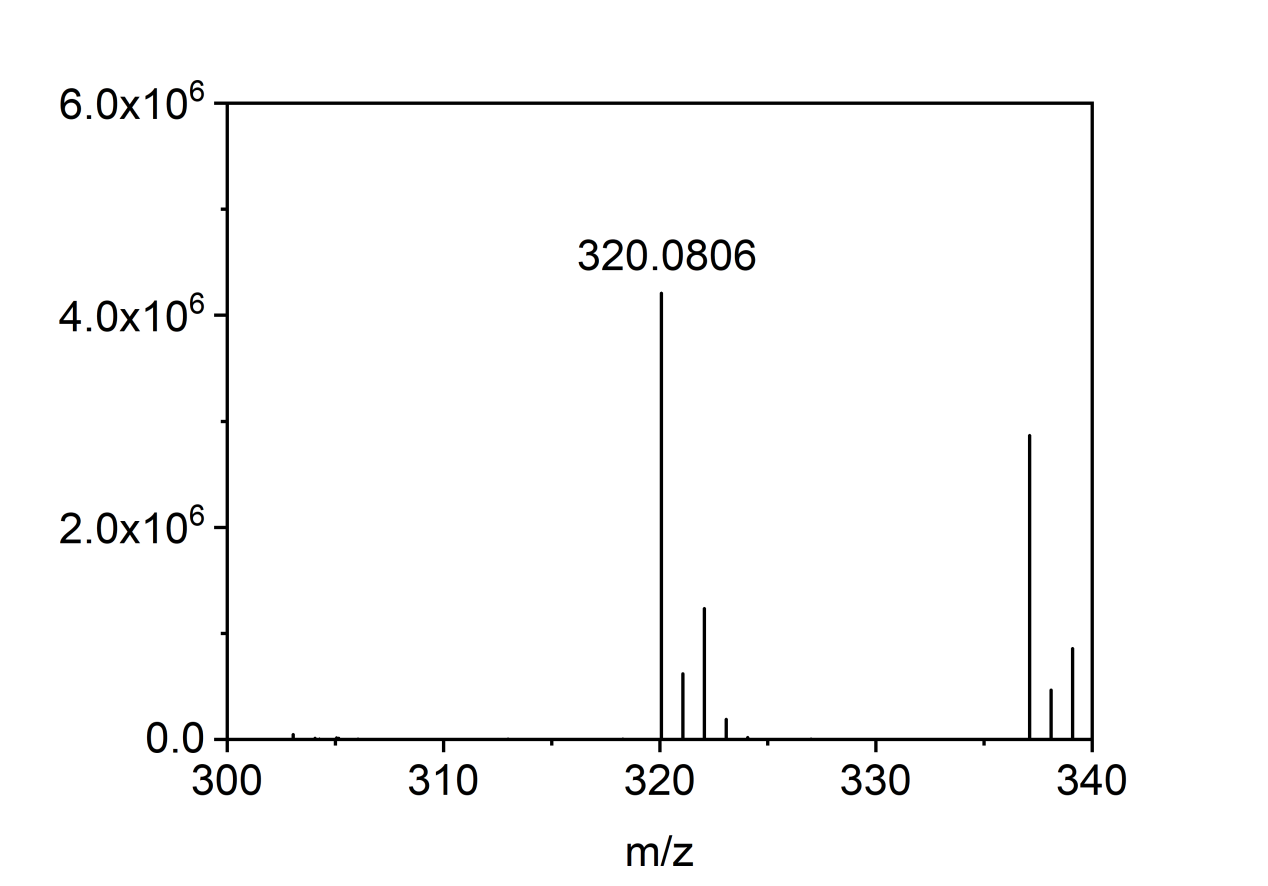
Figure S18.** HRMS Spectrum of compound PyE1.

**
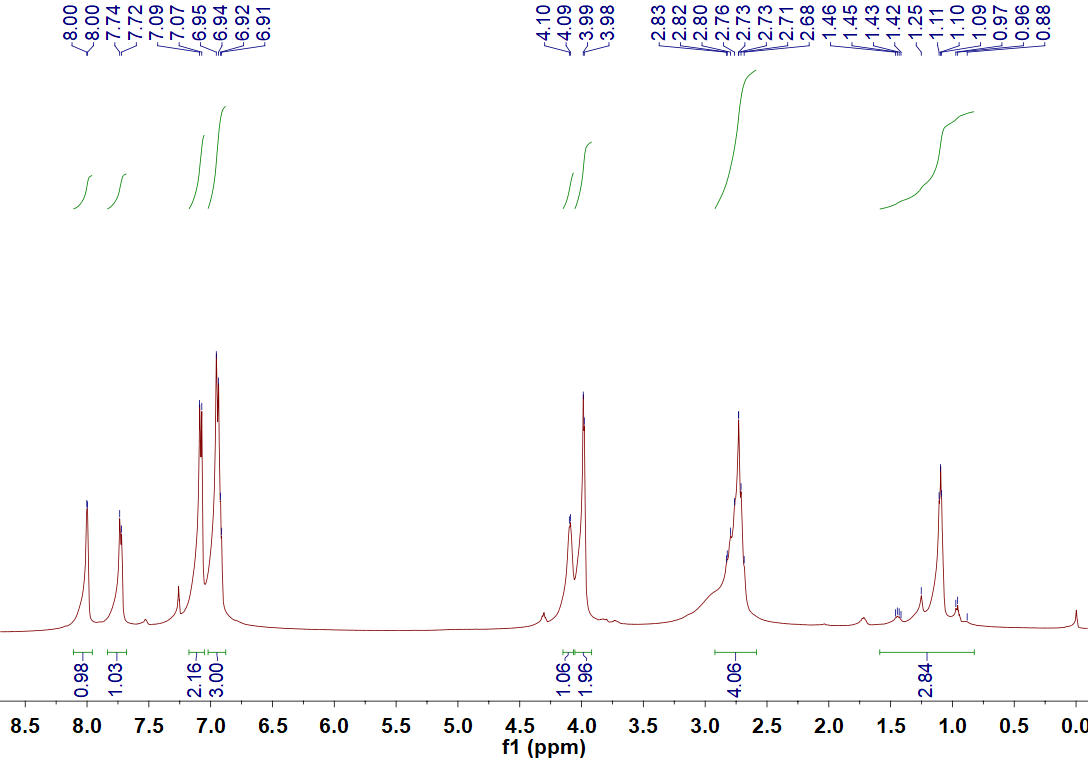
Figure S19.** ^1^H NMR Spectrum (CDCl_3_, 500 MHz) of compound PyE2.

**
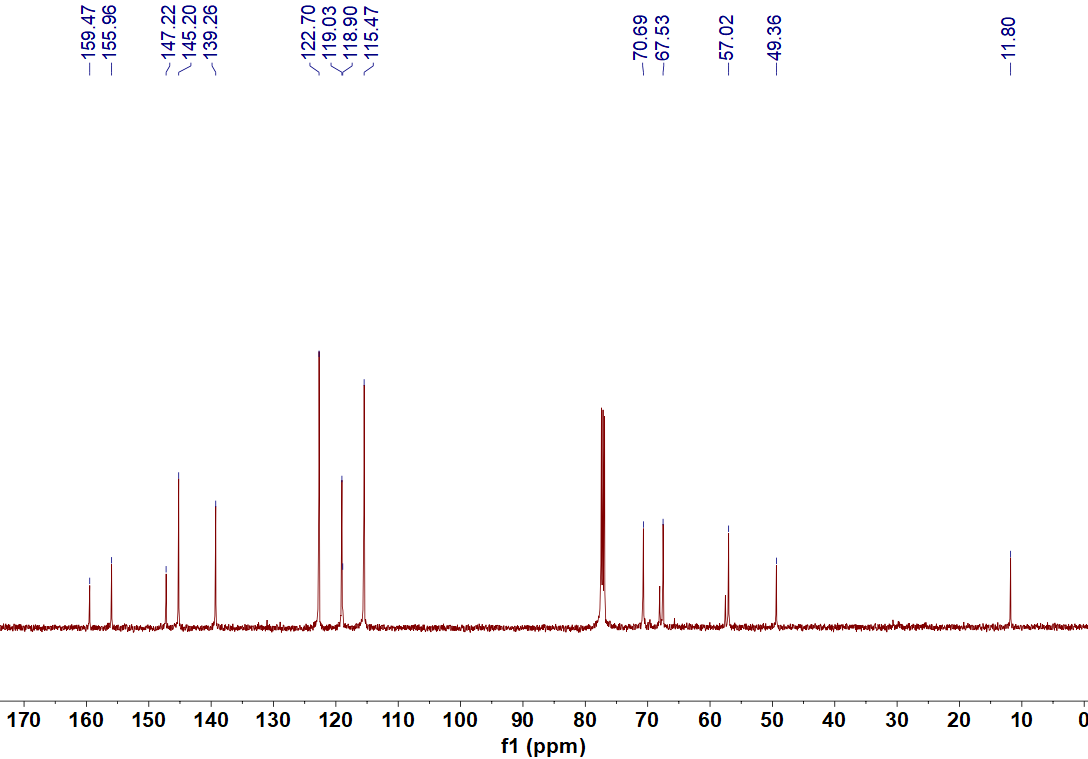
Figure S20.** ^13^C NMR Spectrum (CDCl_3_, 126 MHz) of compound PyE2.

**
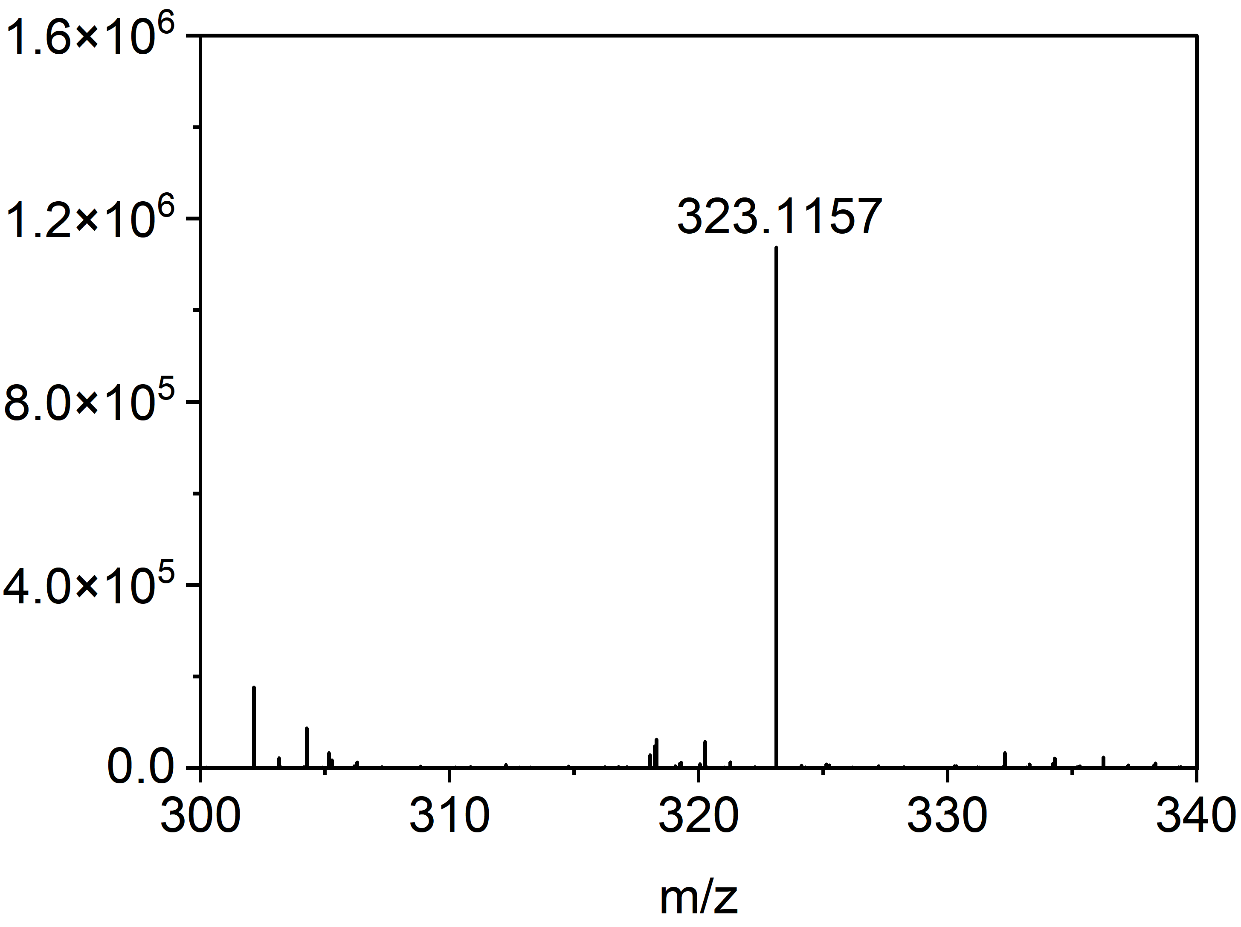
**

**Figure S21.** HRMS Spectrum of compound PyE2.

**
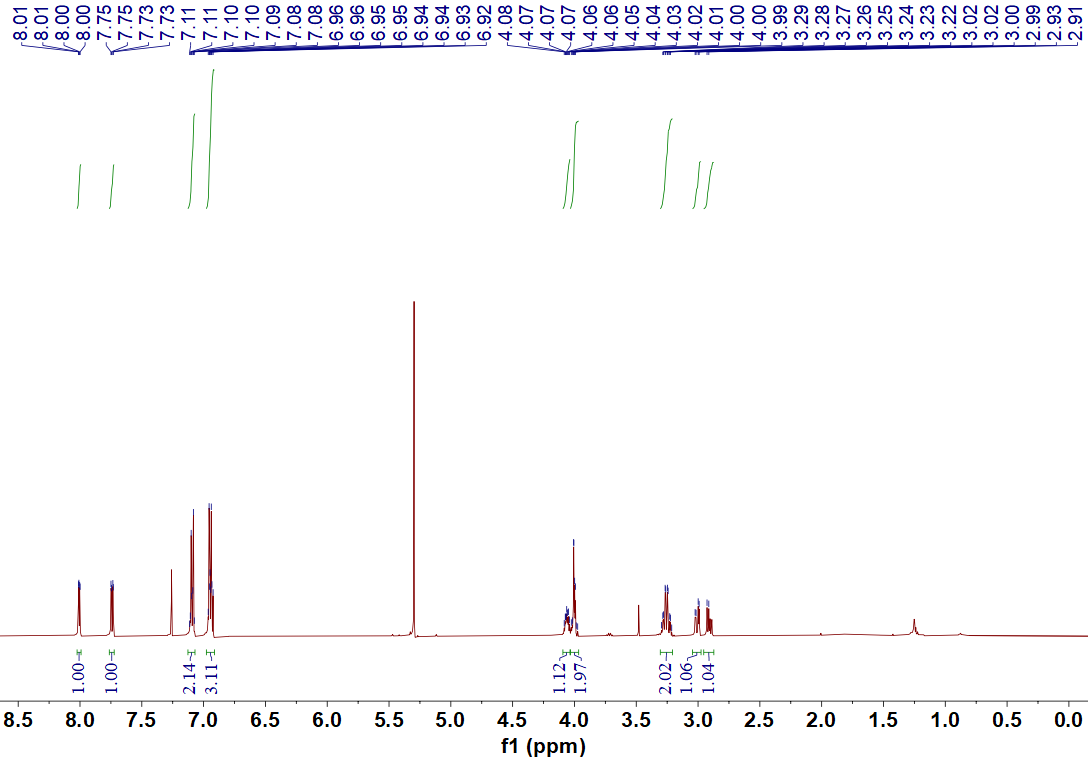
Figure S22.** ^1^H NMR Spectrum (CDCl_3_, 500 MHz) of compound PyE3.

**
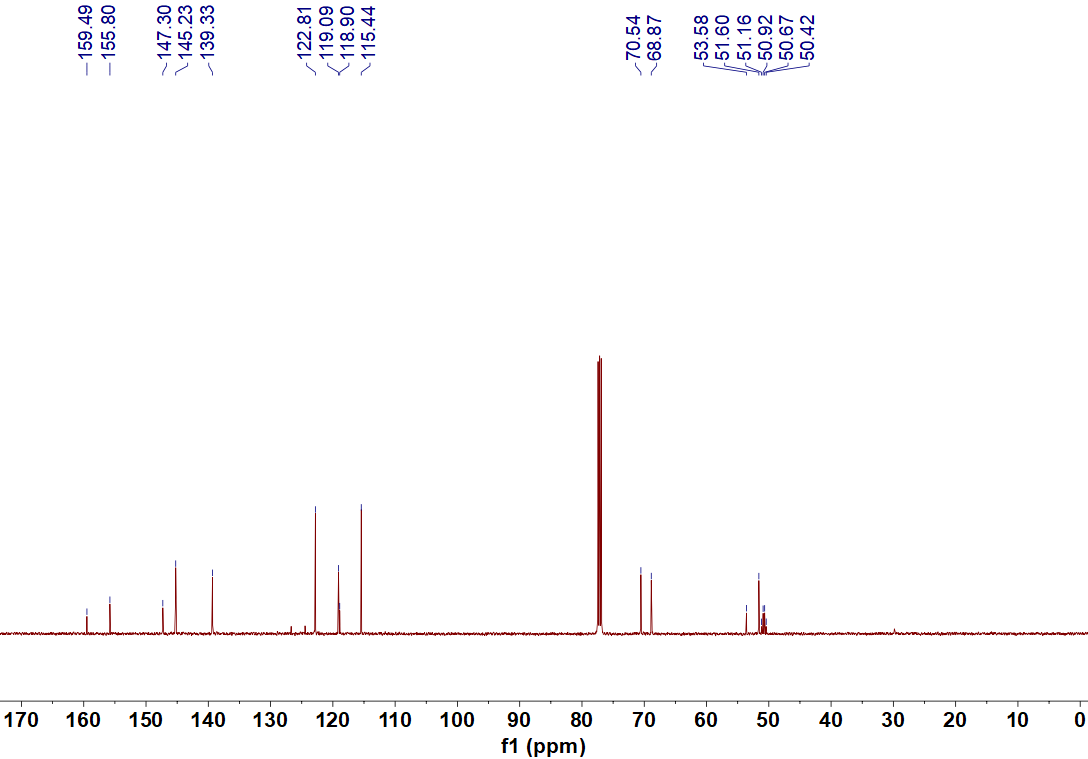
Figure S23.** ^13^C NMR Spectrum (CDCl_3_, 126 MHz) of compound PyE3.

**_
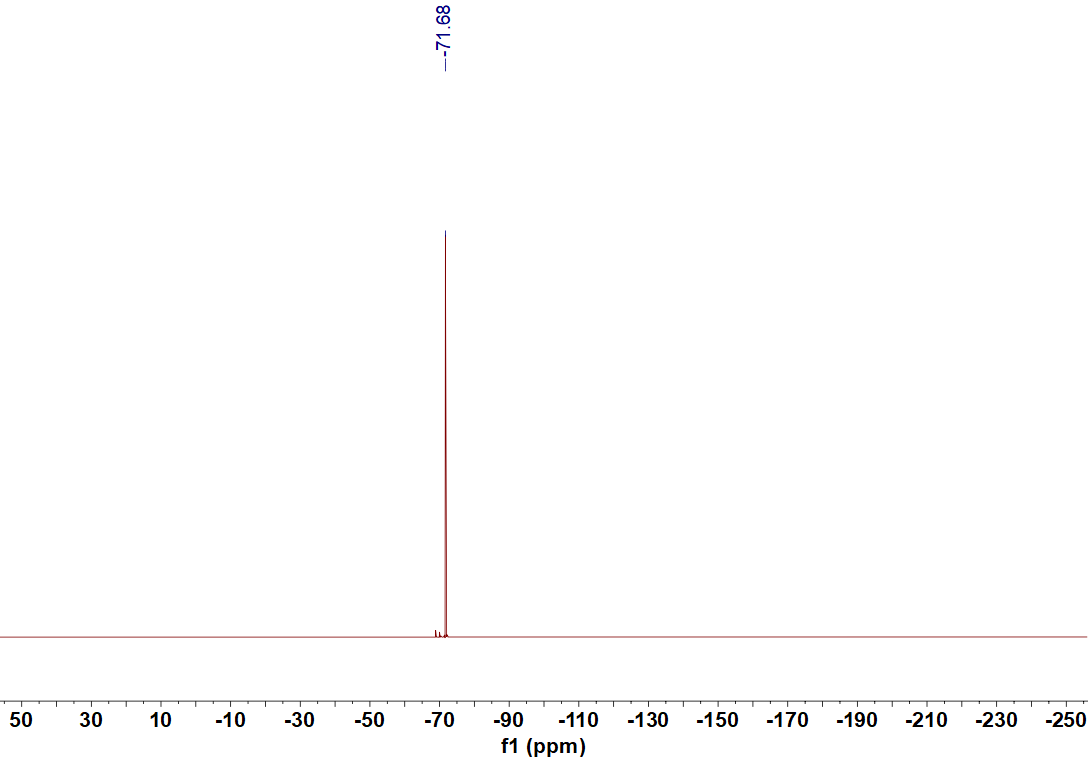
_ Figure S24.** ^19^F NMR Spectrum (CDCl_3_, 471 MHz) of compound PyE3.

**
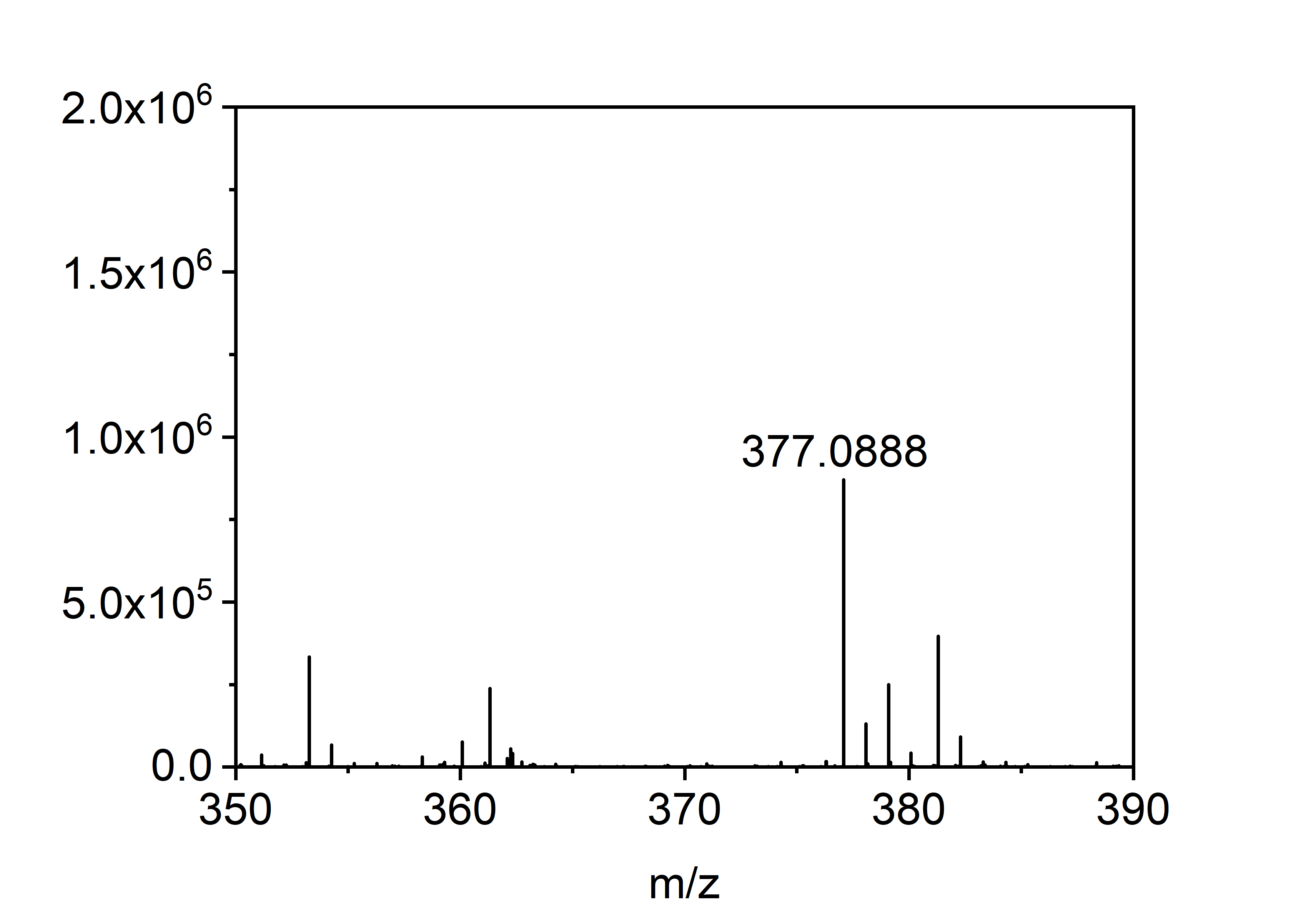
Figure S25.** HRMS Spectrum of compound PyE3.

**
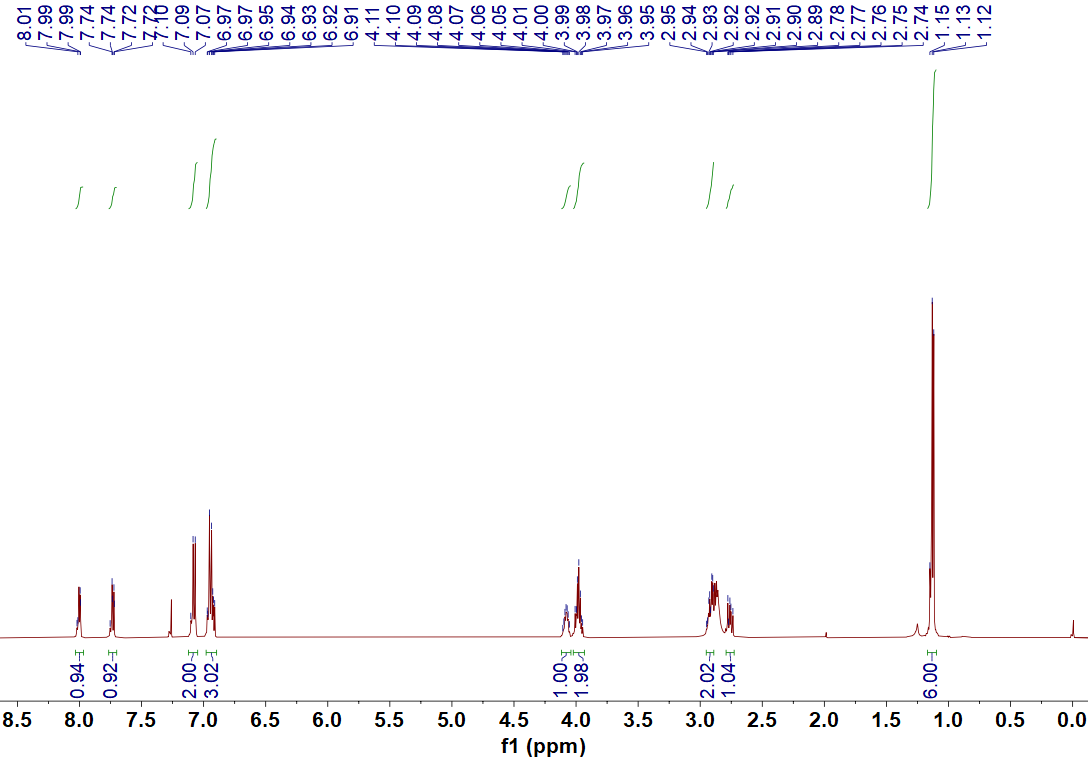
Figure S26.** ^1^H NMR Spectrum (CDCl_3_, 500 MHz) of compound PyE4.

**
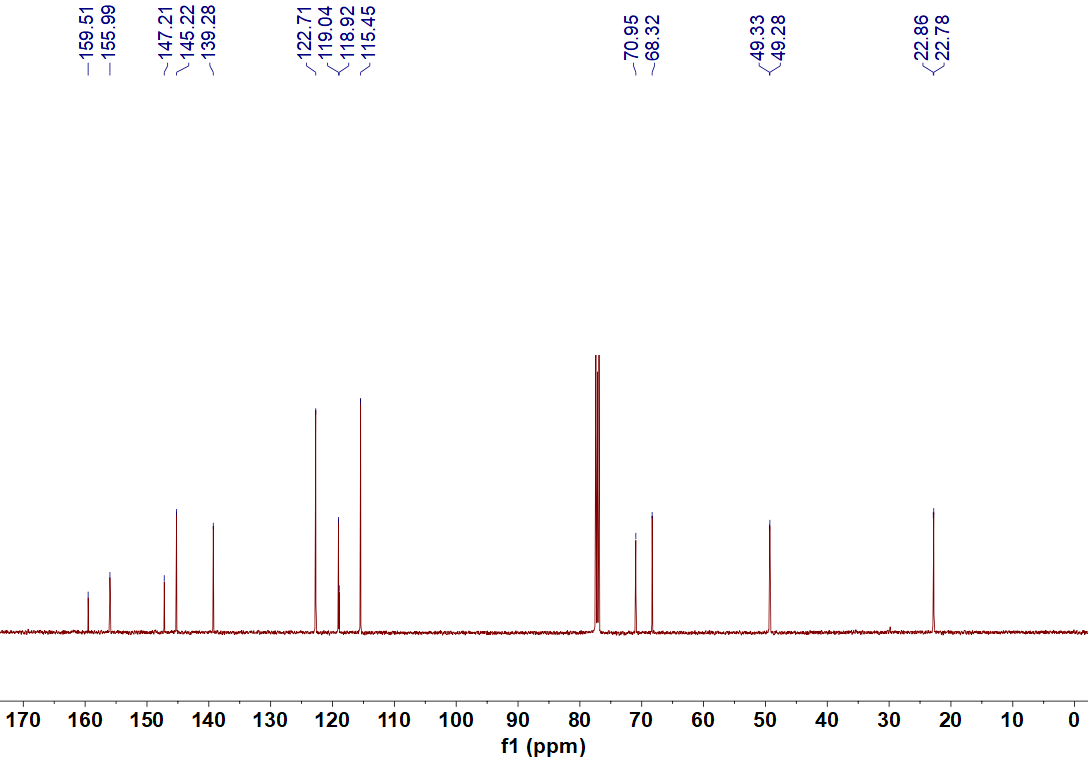
Figure S27.** ^13^C NMR Spectrum (CDCl_3_, 126 MHz) of compound PyE4.

**
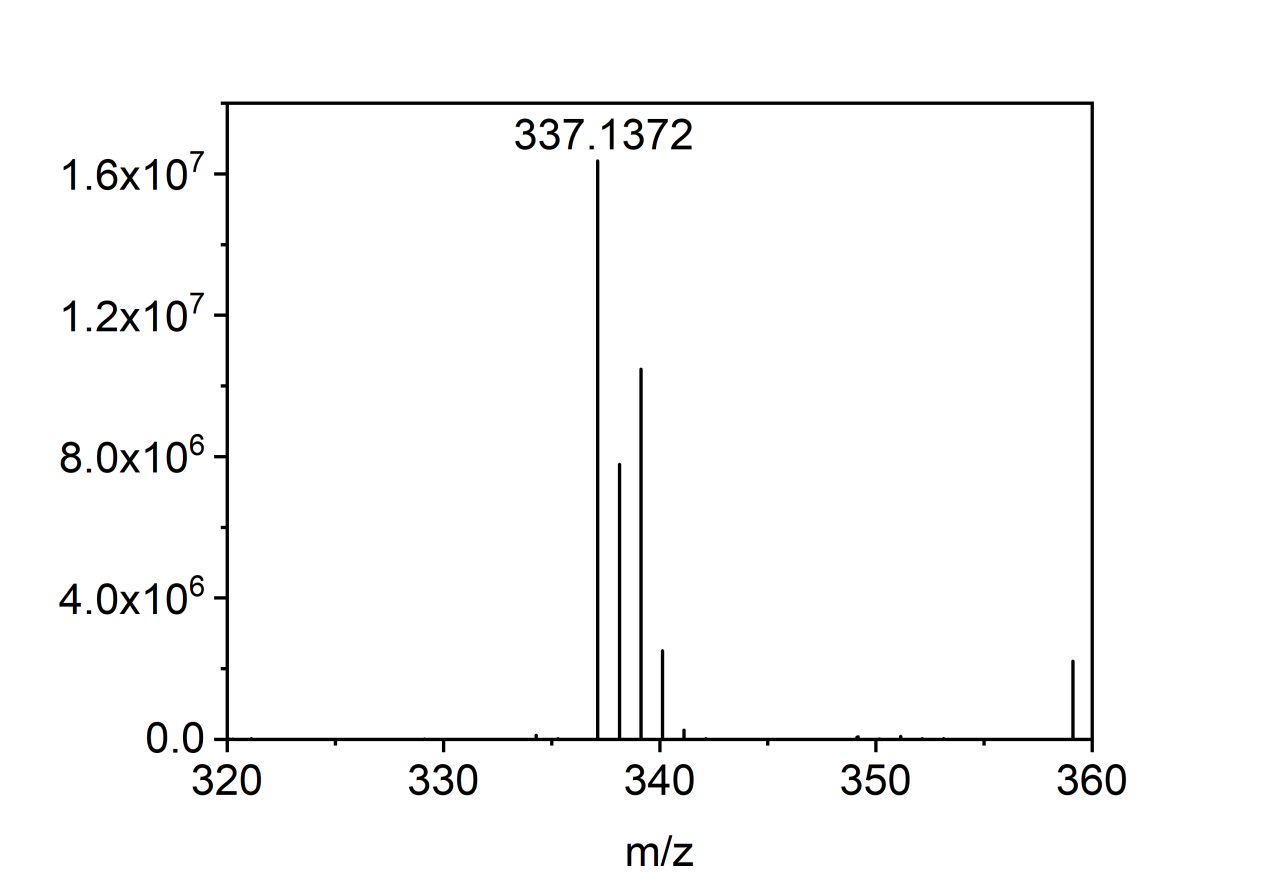
Figure S28.** HRMS Spectrum of compound PyE4.

**
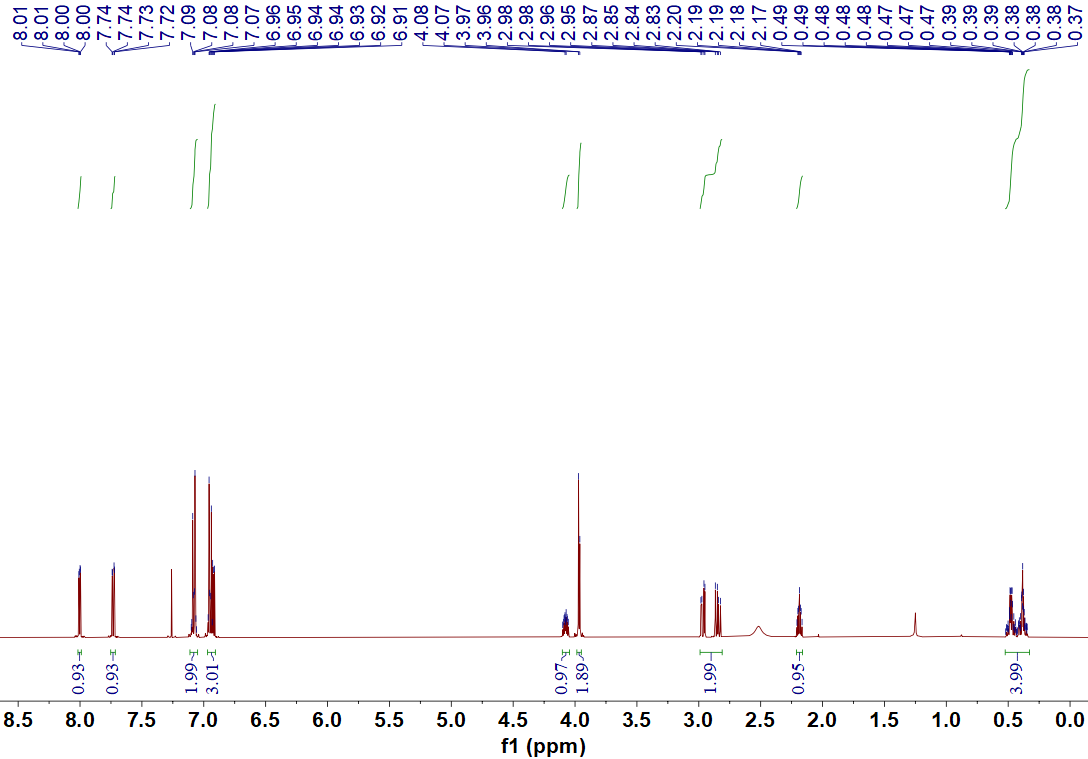
Figure S29.** ^1^H NMR Spectrum (CDCl_3_, 500 MHz) of compound PyE5.

**
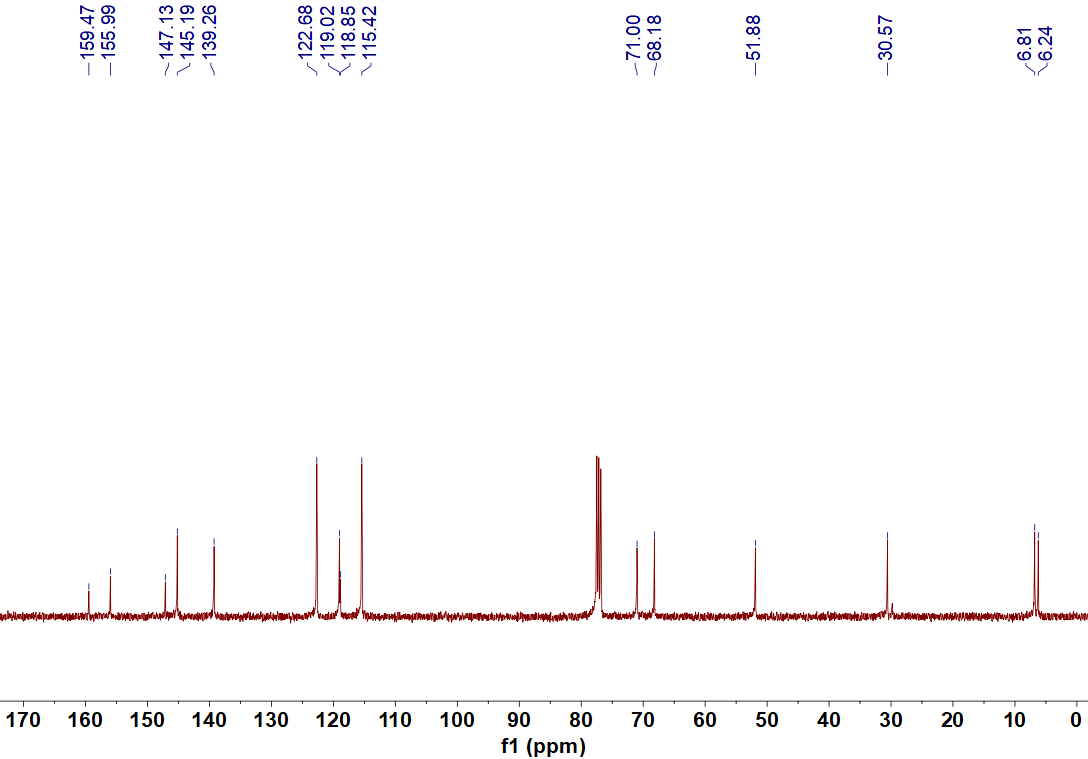
Figure S30.** ^13^C NMR Spectrum (CDCl_3_, 101 MHz) of compound PyE5.

**
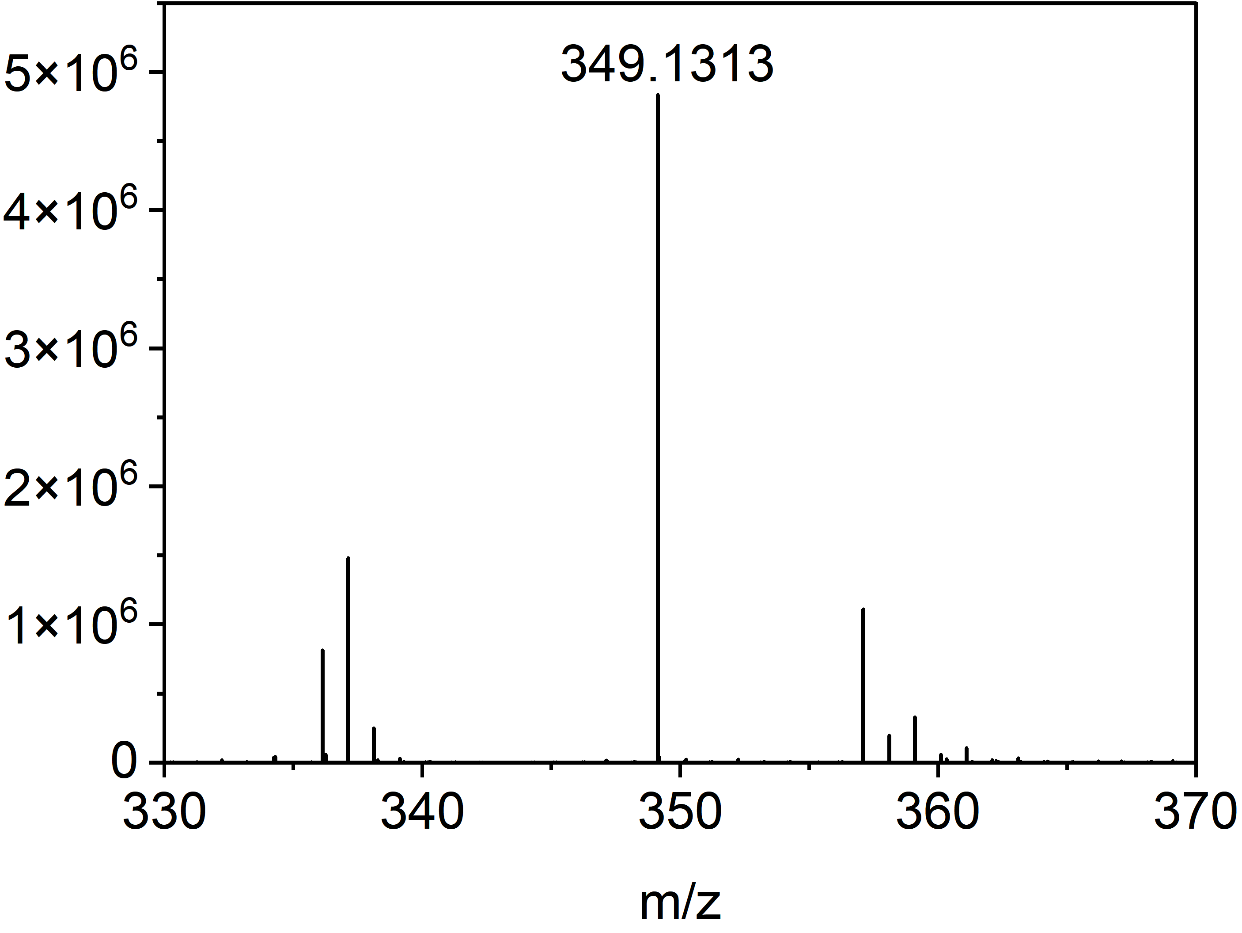
Figure S31.** HRMS Spectrum of compound PyE5.

**
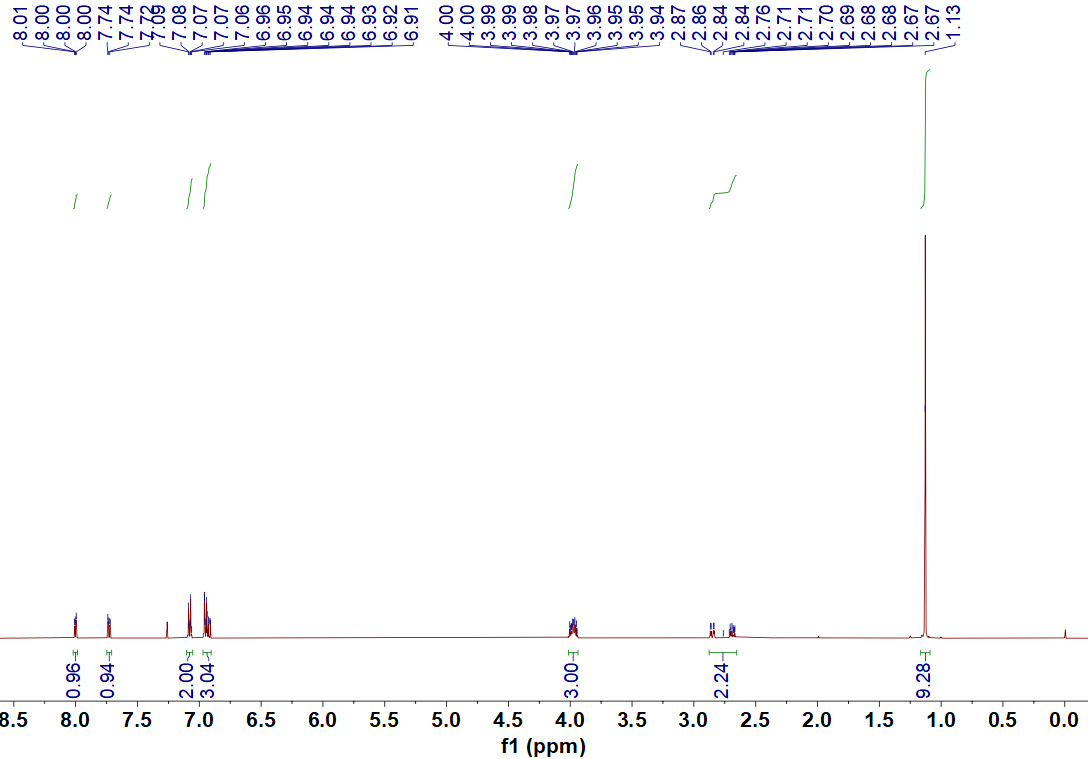
Figure S32.** ^1^H NMR Spectrum (CDCl_3_, 500 MHz) of compound PyE6.

**
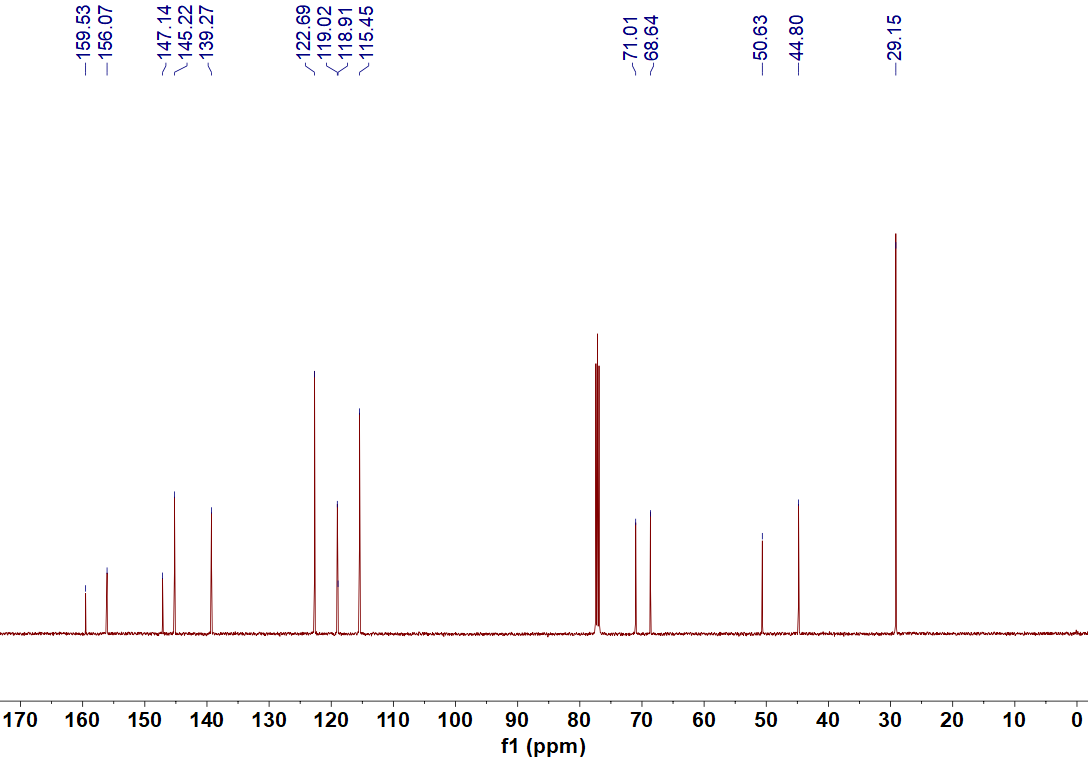
Figure S33.** ^13^C NMR Spectrum (CDCl_3_, 126 MHz) of compound PyE6.

**Figure S34.** HRMS Spectrum of compound PyE6.

**
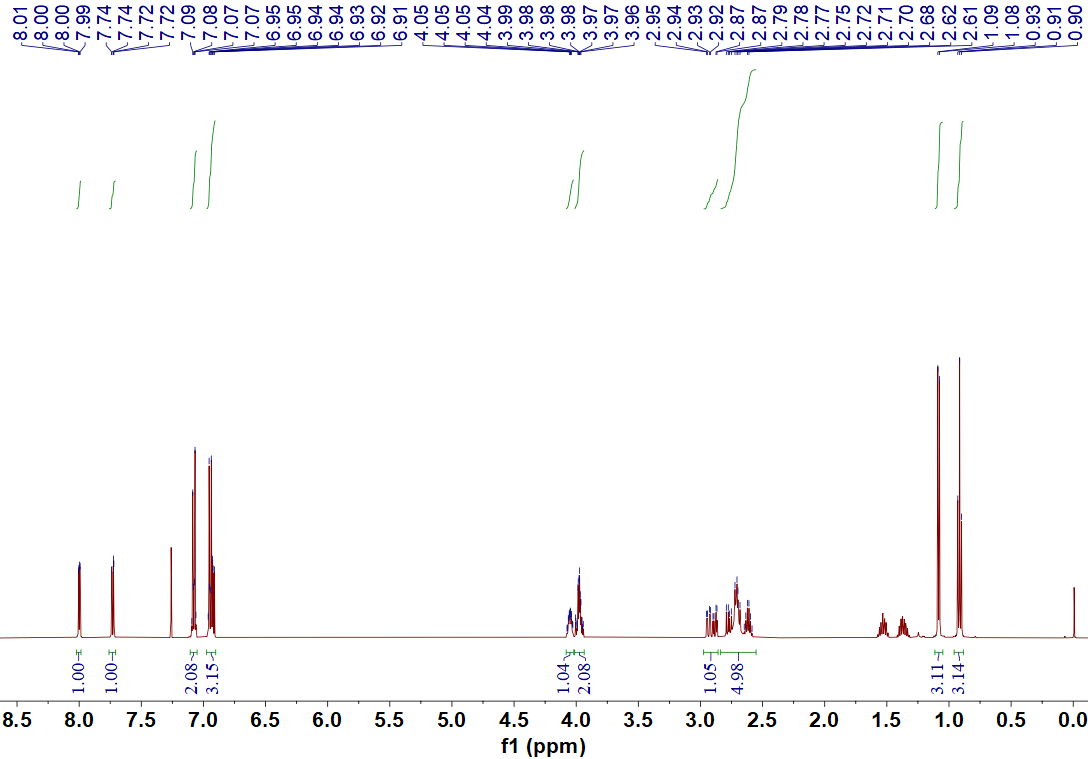
Figure S35.** ^1^H NMR Spectrum (CDCl_3_, 500 MHz) of compound PyE7.

**
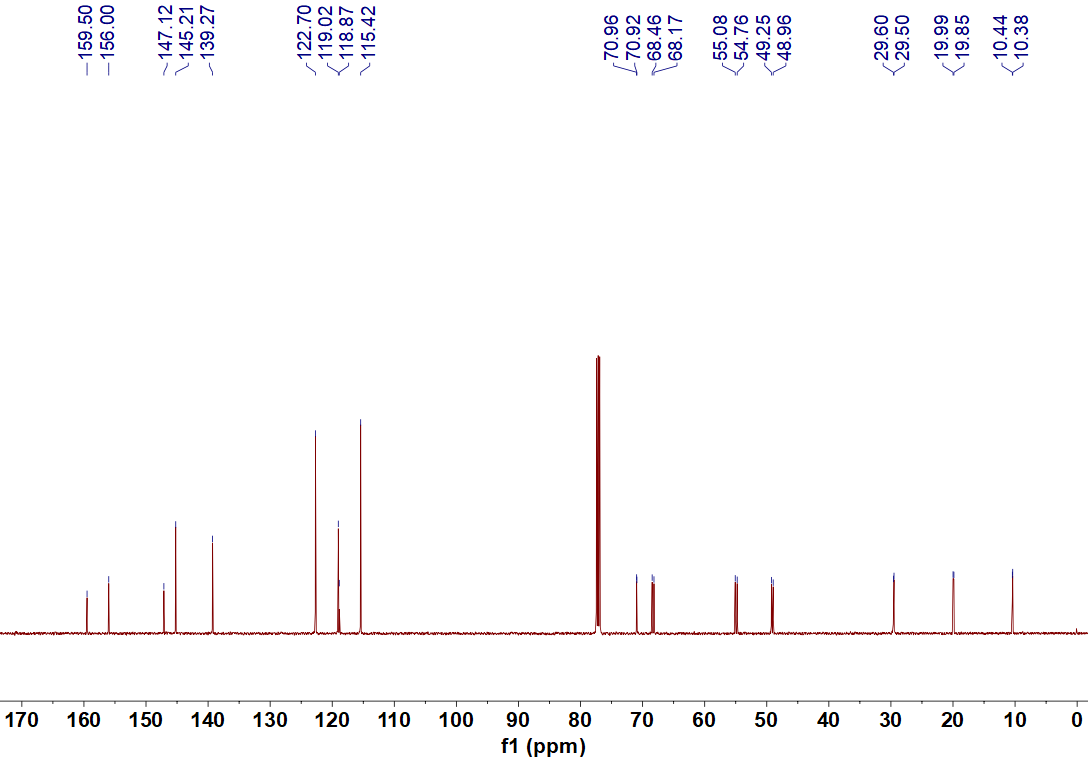
Figure S36.** ^13^C NMR Spectrum (CDCl_3_, 126 MHz) of compound PyE7.

**
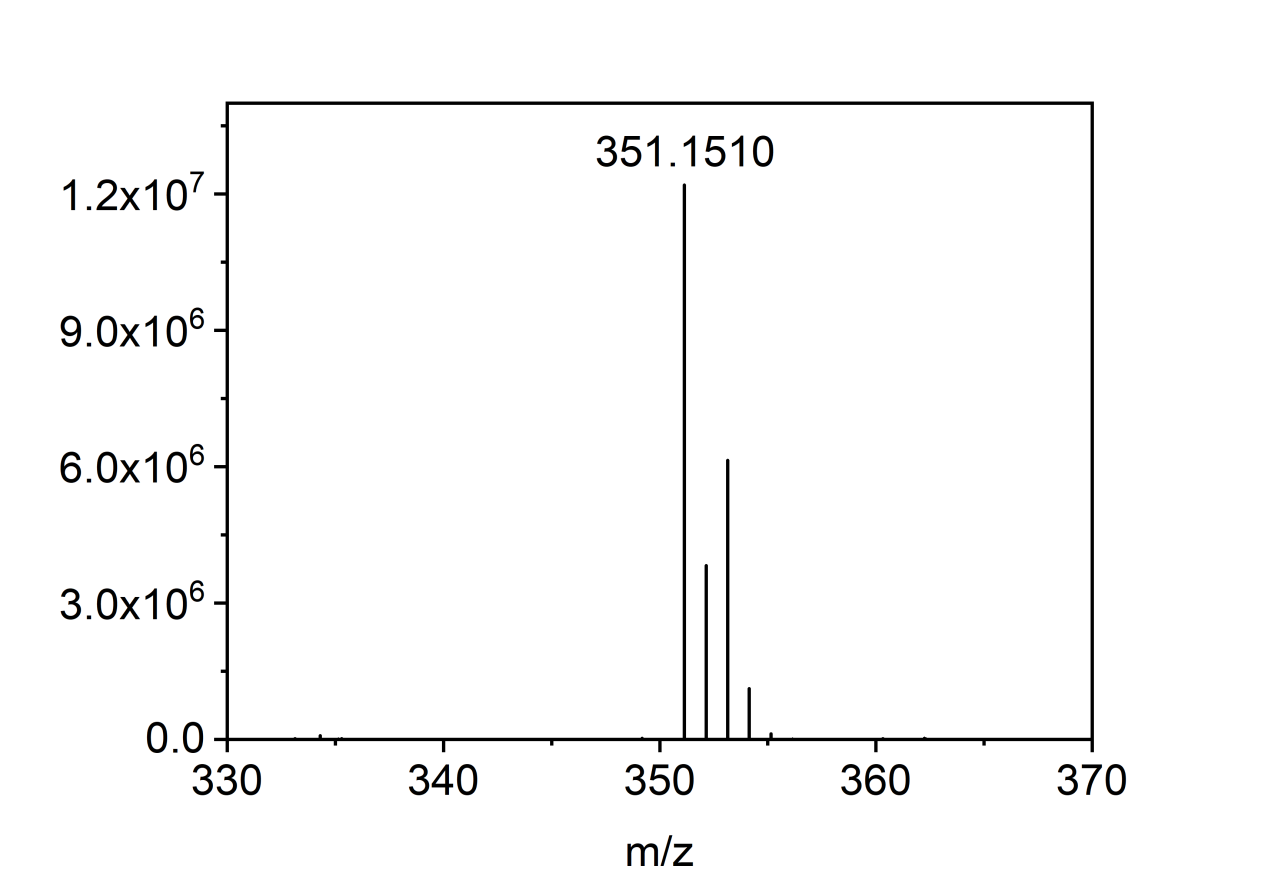
Figure S37.** HRMS Spectrum of compound PyE7.

**
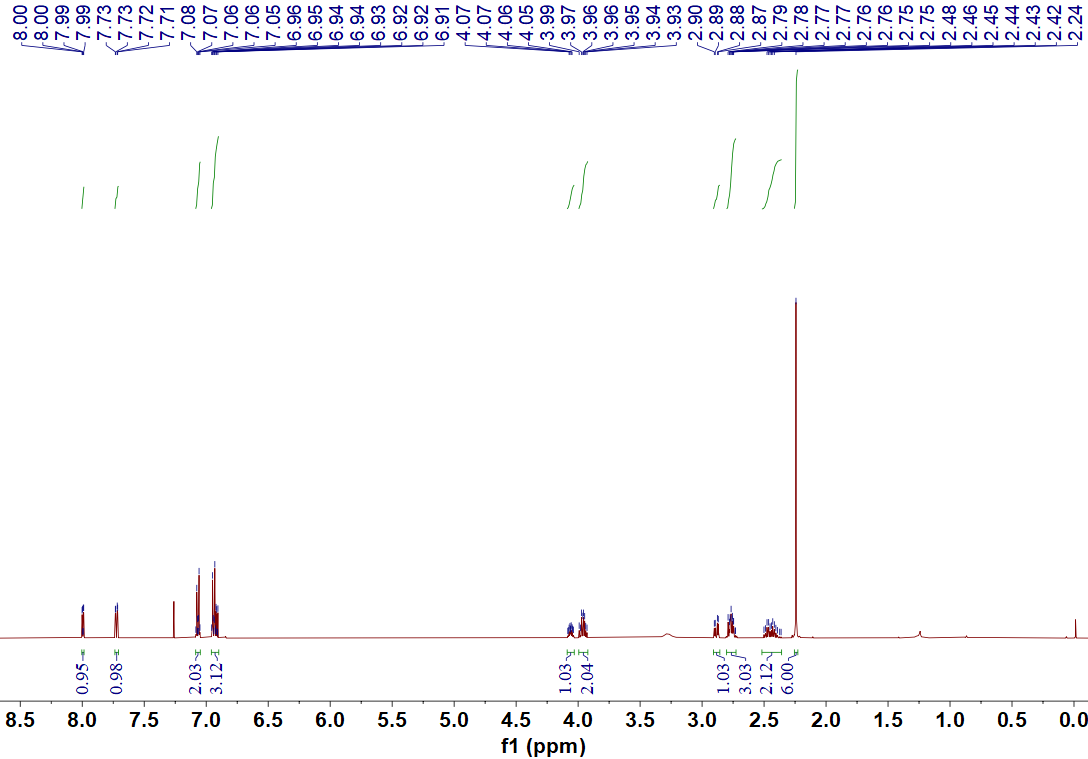
Figure S38.** ^1^H NMR Spectrum (CDCl_3_, 500 MHz) of compound PyE8.

**
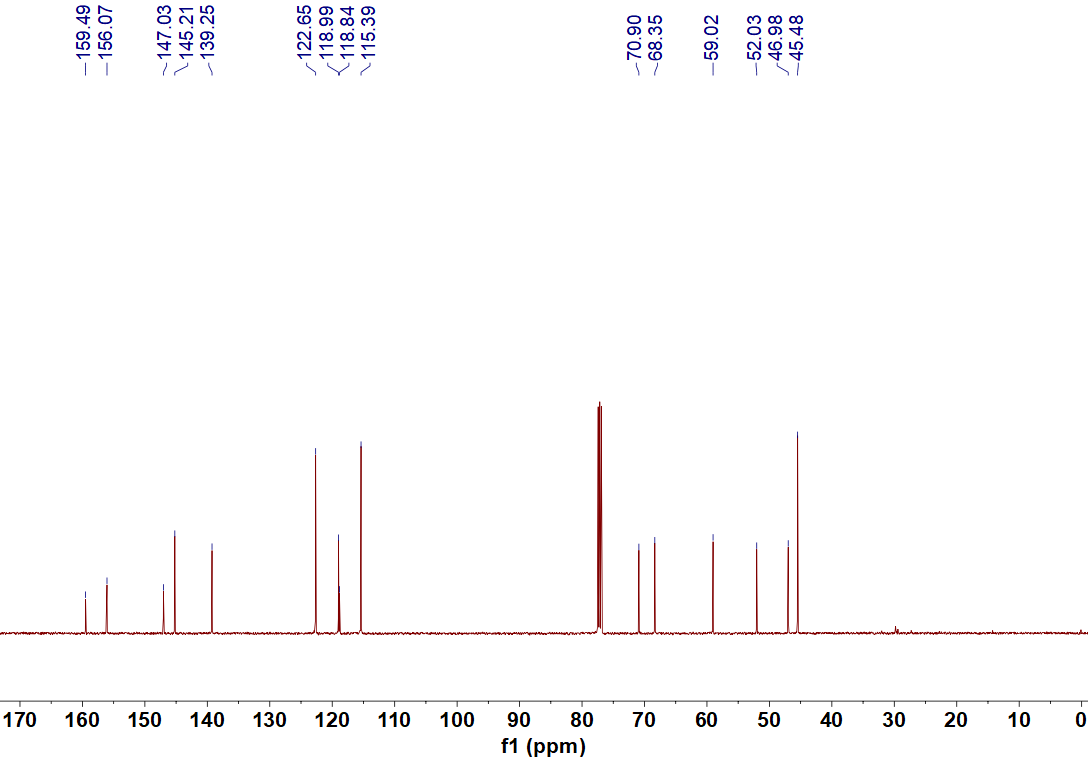
Figure S39.** ^13^C NMR Spectrum (CDCl_3_, 126 MHz) of compound PyE8.

**
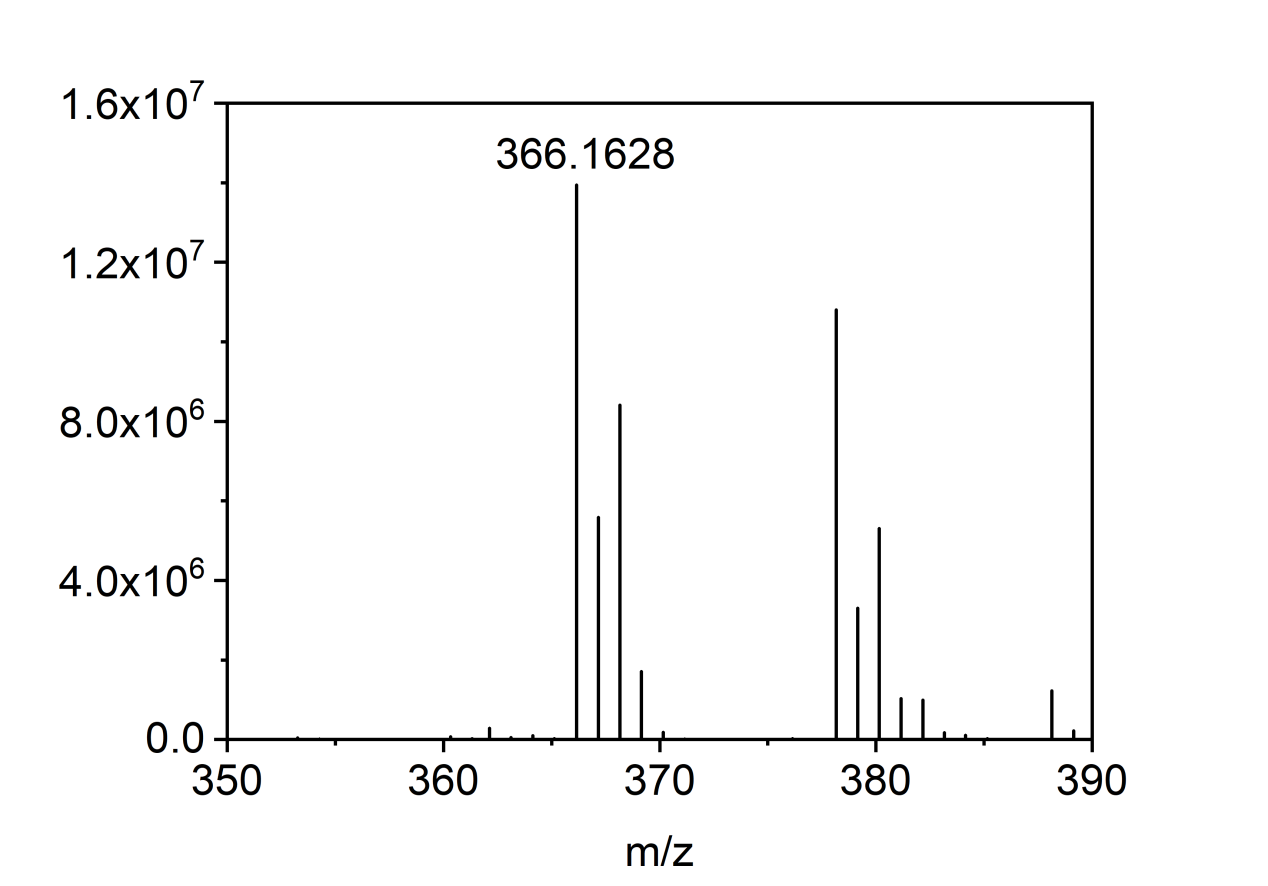
Figure S40.** HRMS Spectrum of compound PyE8.

**
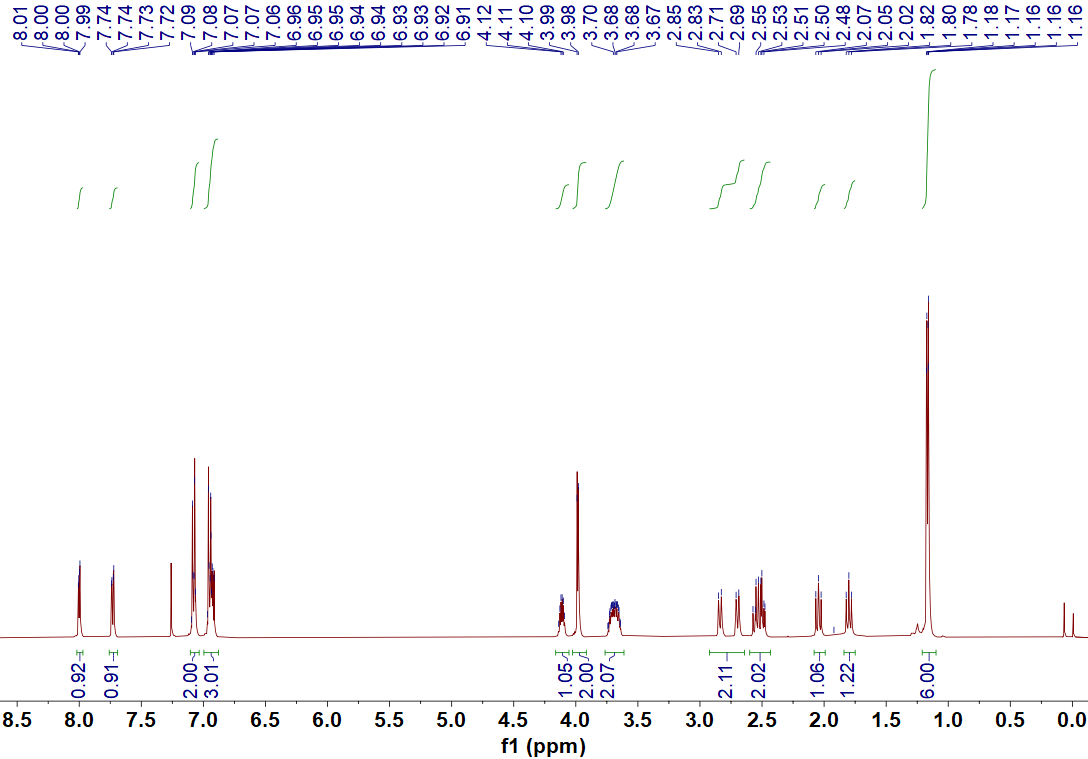
Figure S41.** ^1^H NMR Spectrum (CDCl_3_, 500 MHz) of compound PyE9.

**
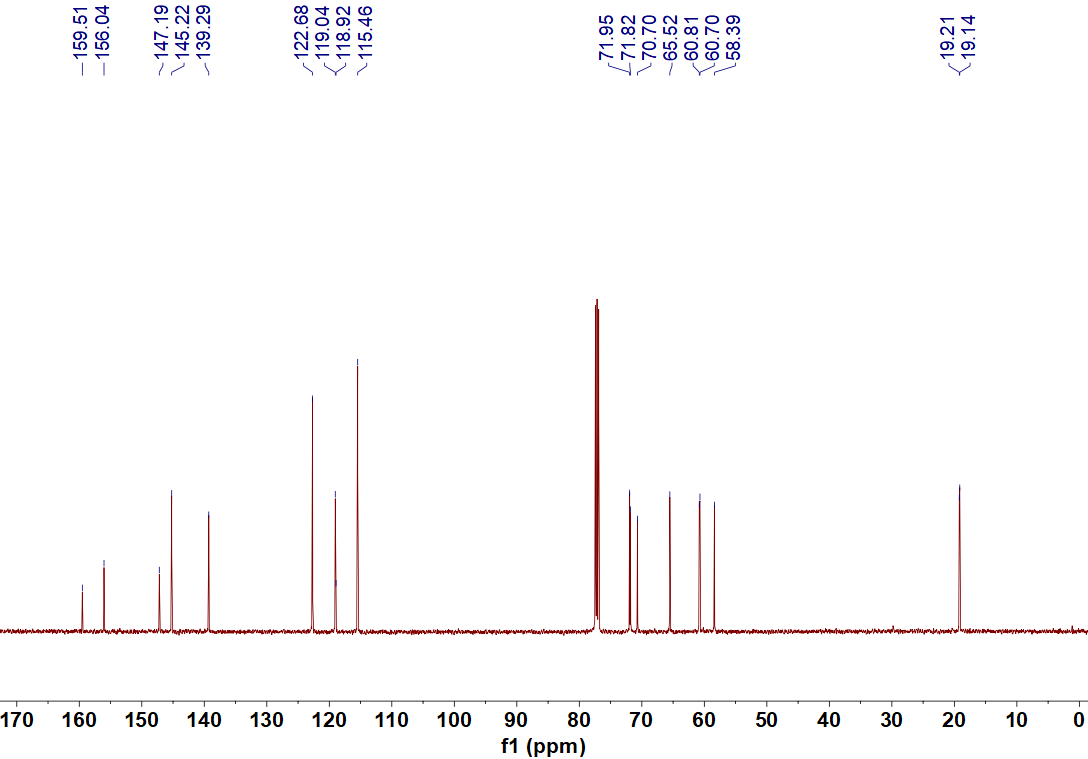
Figure S42.** ^13^C NMR Spectrum (CDCl_3_, 126 MHz) of compound PyE9.

**Figure S43.** HRMS Spectrum of compound PyE9.

**
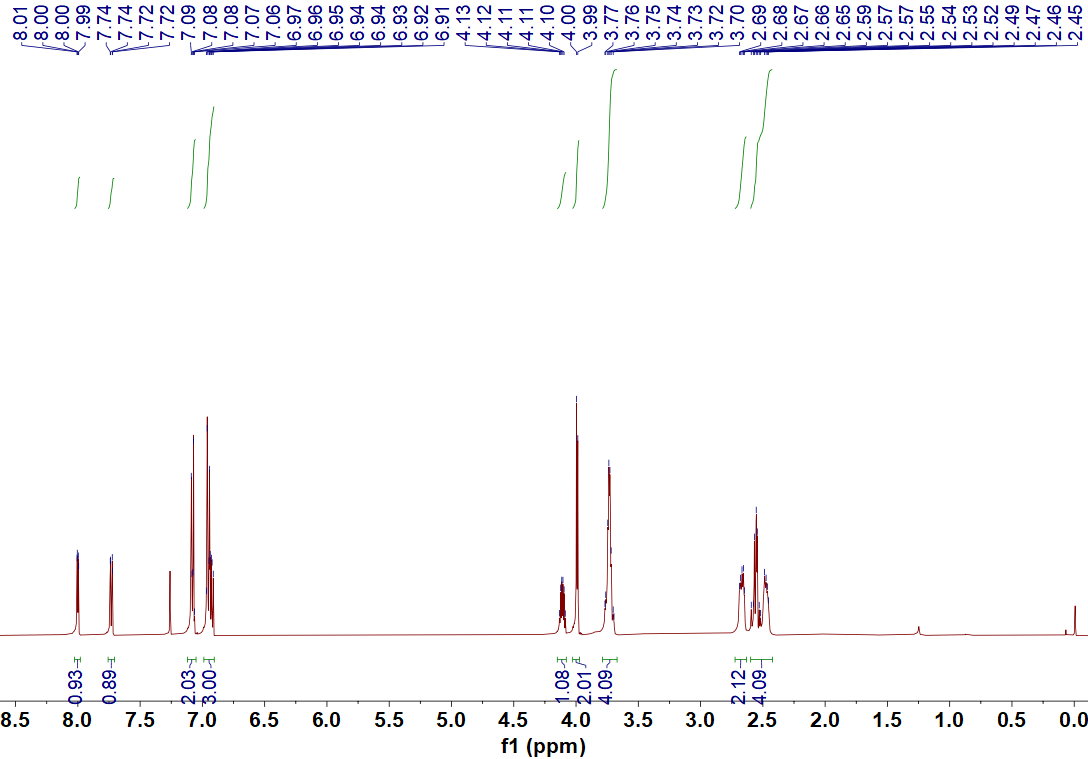
Figure S44.** ^1^H NMR Spectrum (CDCl_3_, 500 MHz) of compound PyE10.

**
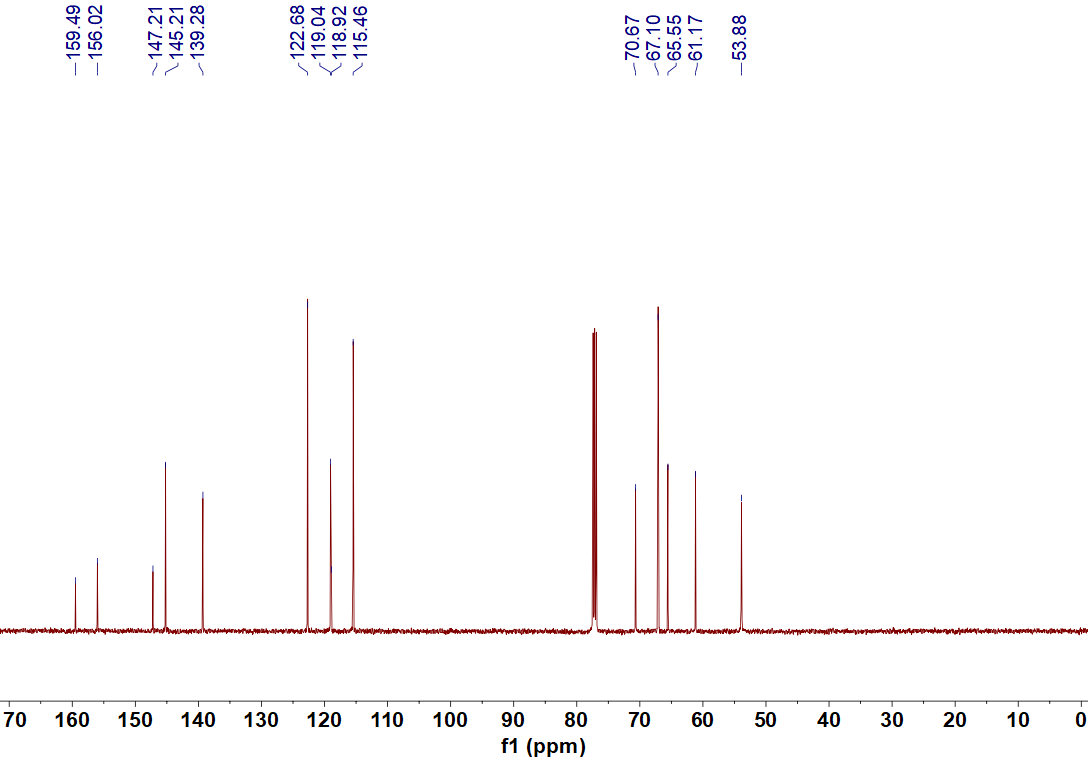
Figure S45.** ^13^C NMR Spectrum (CDCl_3_, 126 MHz) of compound PyE10.

**Figure S46.** HRMS Spectrum of compound PyE10.

**
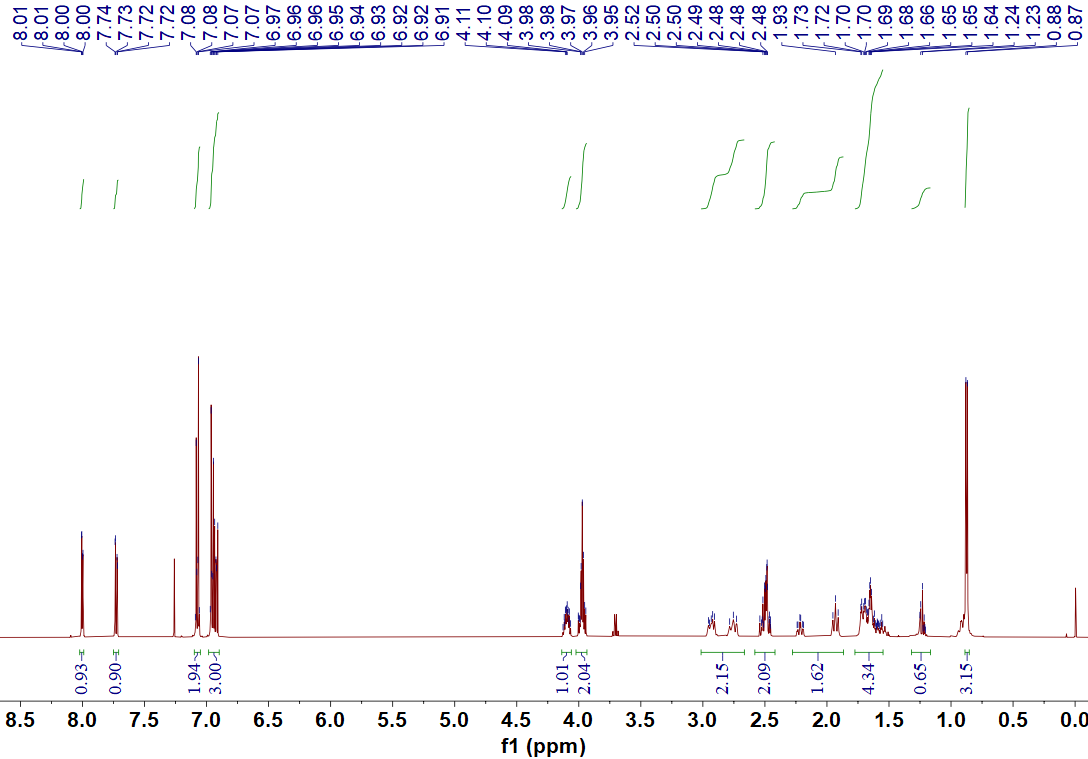
Figure S47.** ^1^H NMR Spectrum (CDCl_3_, 500 MHz) of compound PyE11.

**
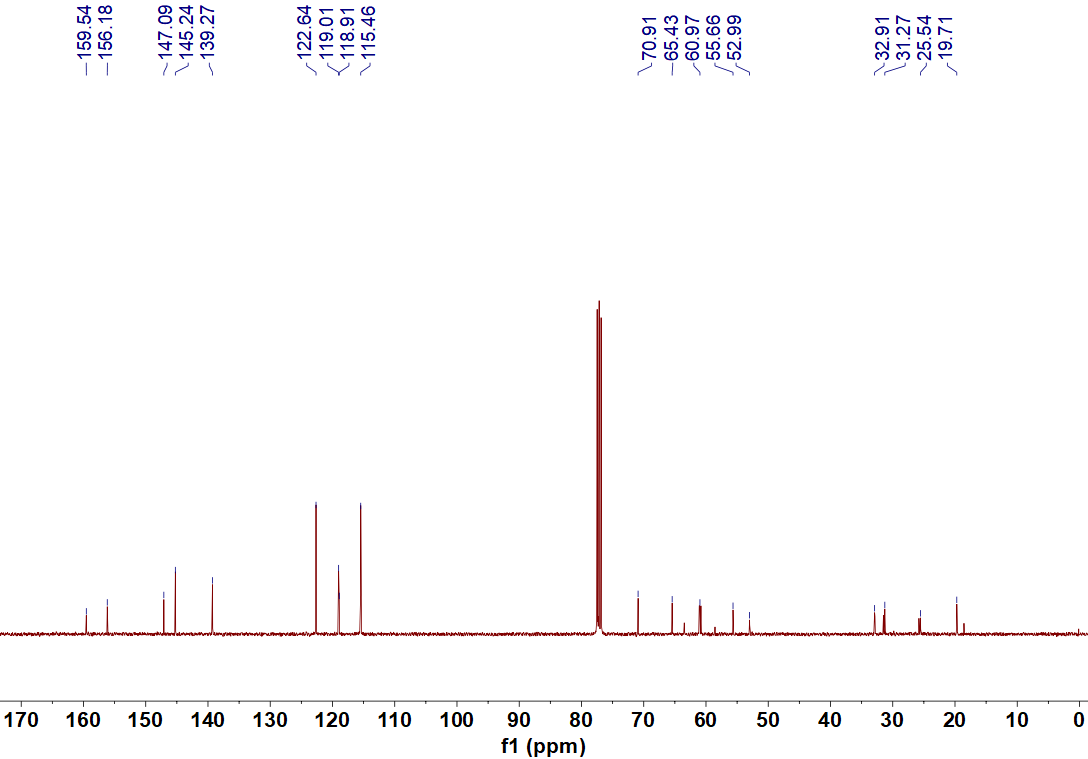
Figure S48.** ^13^C NMR Spectrum (CDCl_3_, 101 MHz) of compound PyE11.

**Figure S49.** HRMS Spectrum of compound PyE11.

**
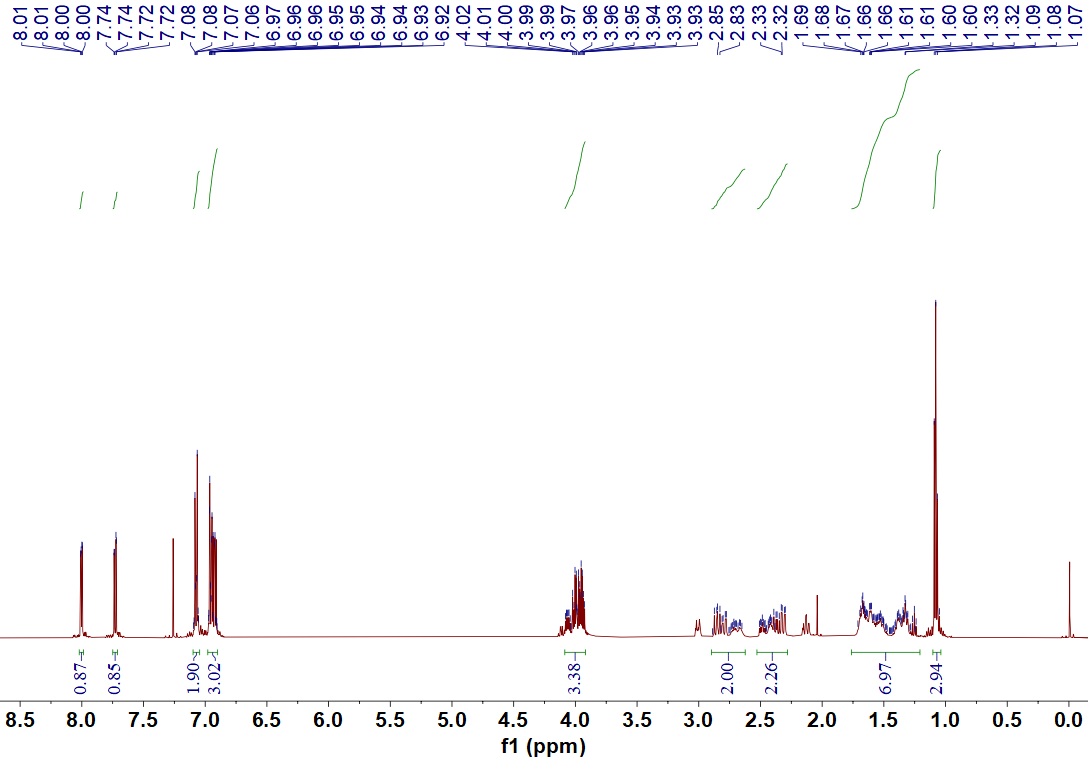
Figure S50.** ^1^H NMR Spectrum (CDCl_3_, 500 MHz) of compound PyE12.

**
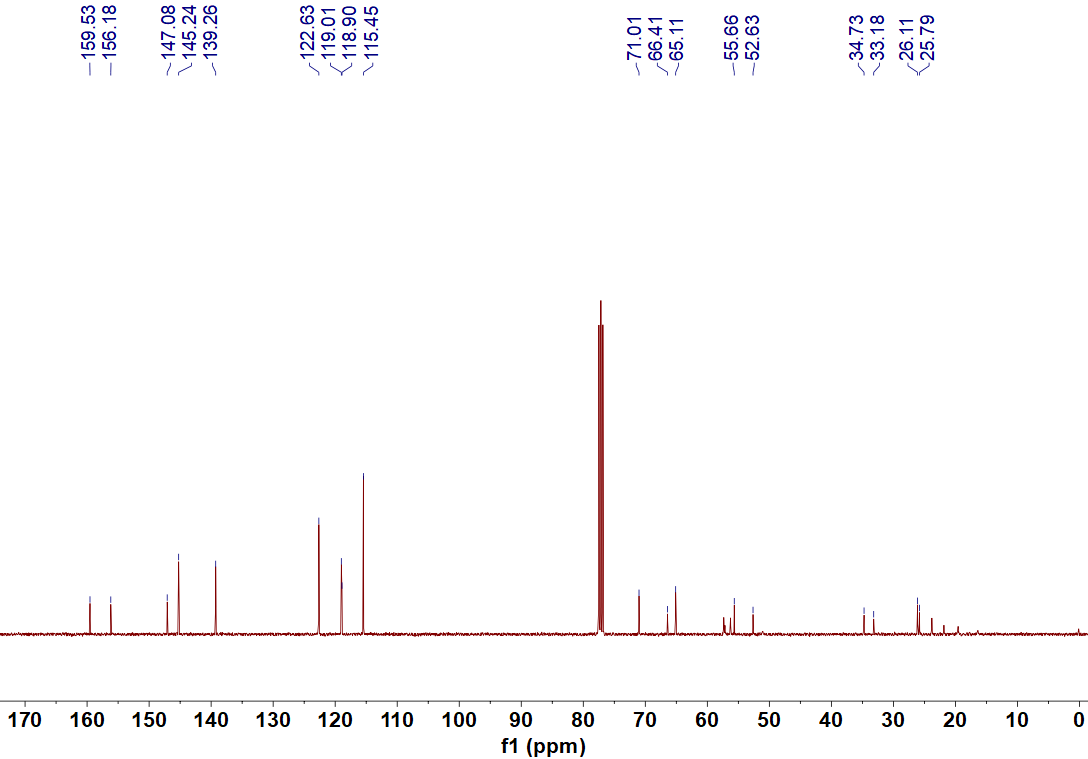
Figure S51.** ^13^C NMR Spectrum (CDCl_3_, 101 MHz) of compound PyE12.

**
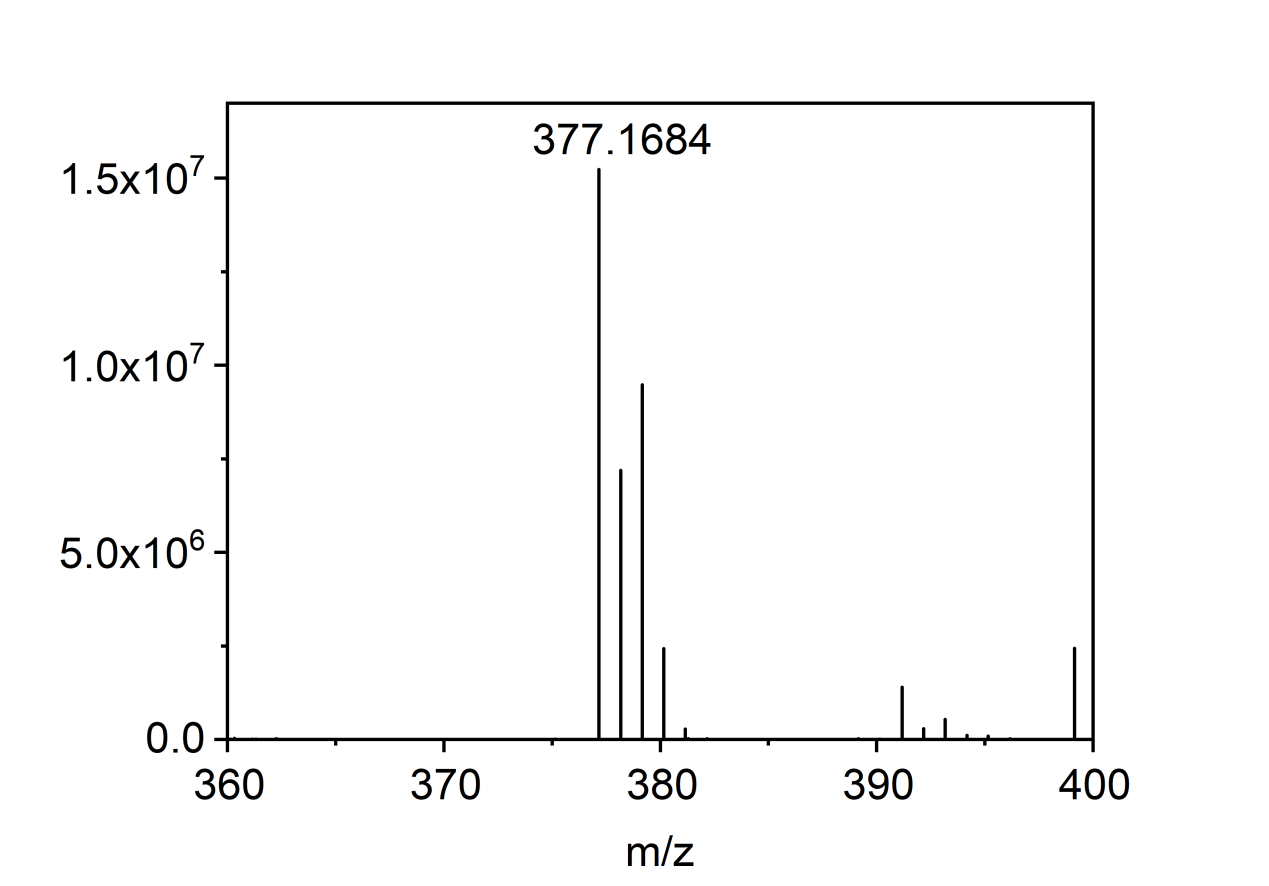
Figure S52.** HRMS Spectrum of compound PyE12.

**
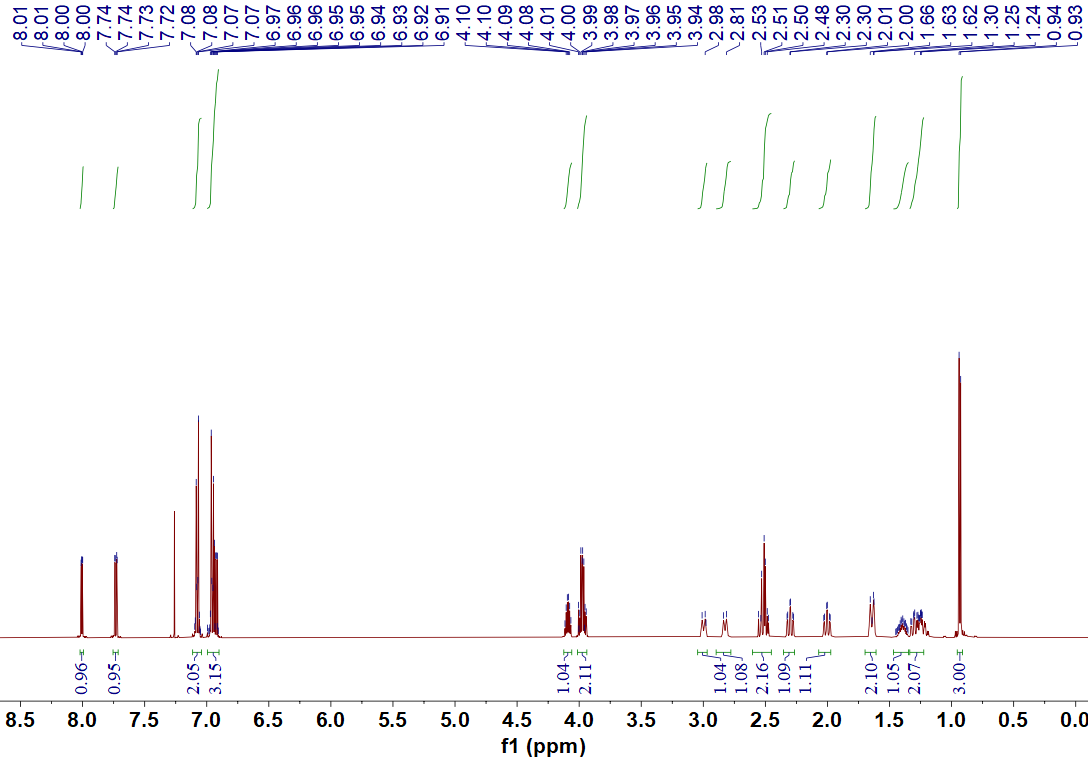
Figure S53.** ^1^H NMR Spectrum (CDCl_3_, 500 MHz) of compound PyE13.

**
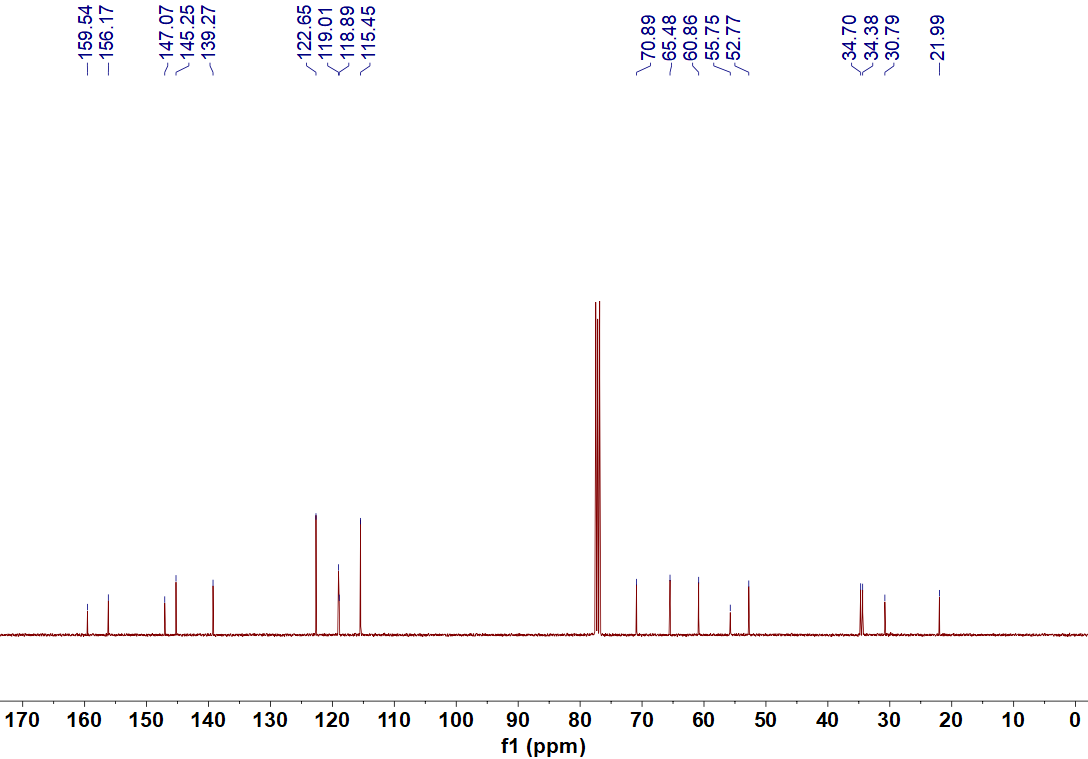
Figure S54.** ^13^C NMR Spectrum (CDCl_3_, 101 MHz) of compound PyE13.

**
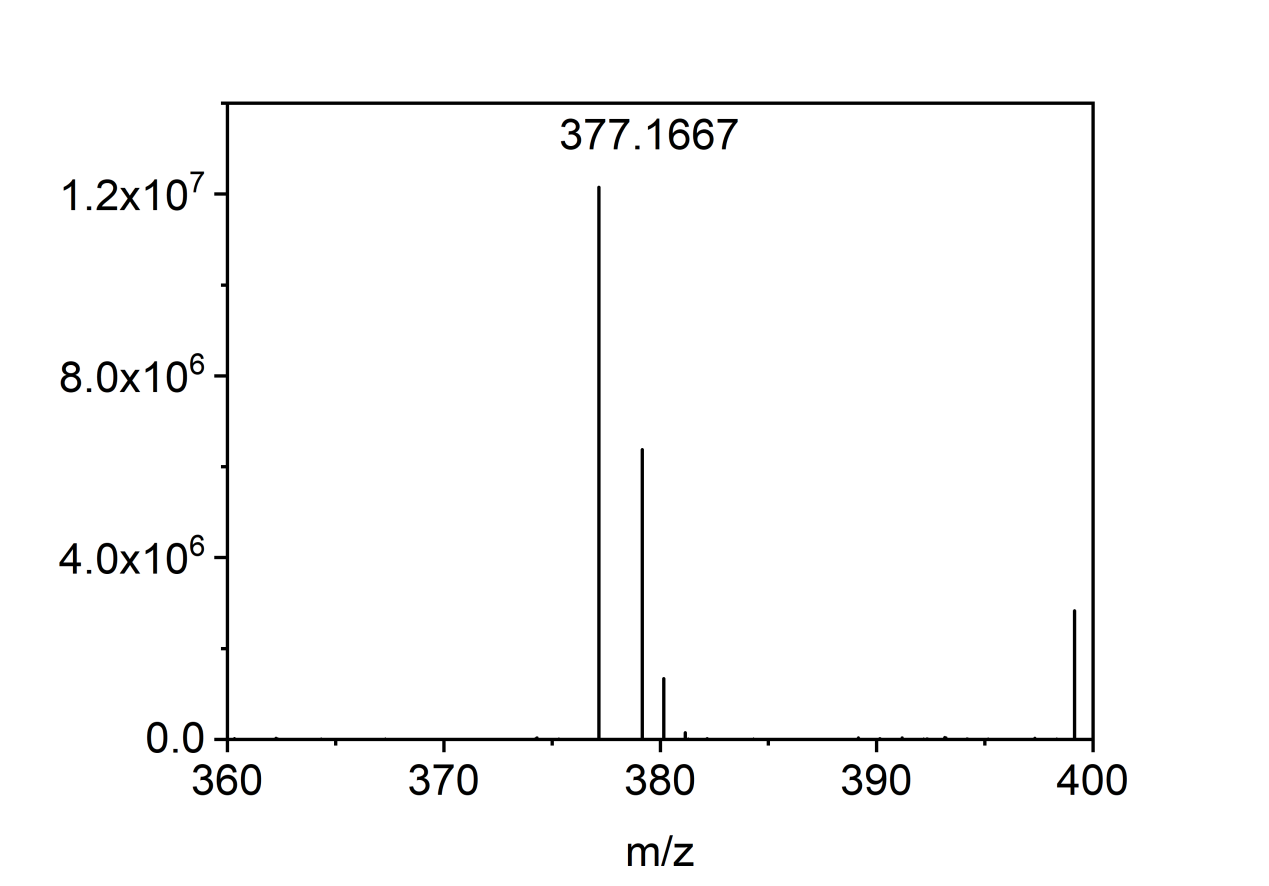
Figure S55.** HRMS Spectrum of compound PyE13.

**
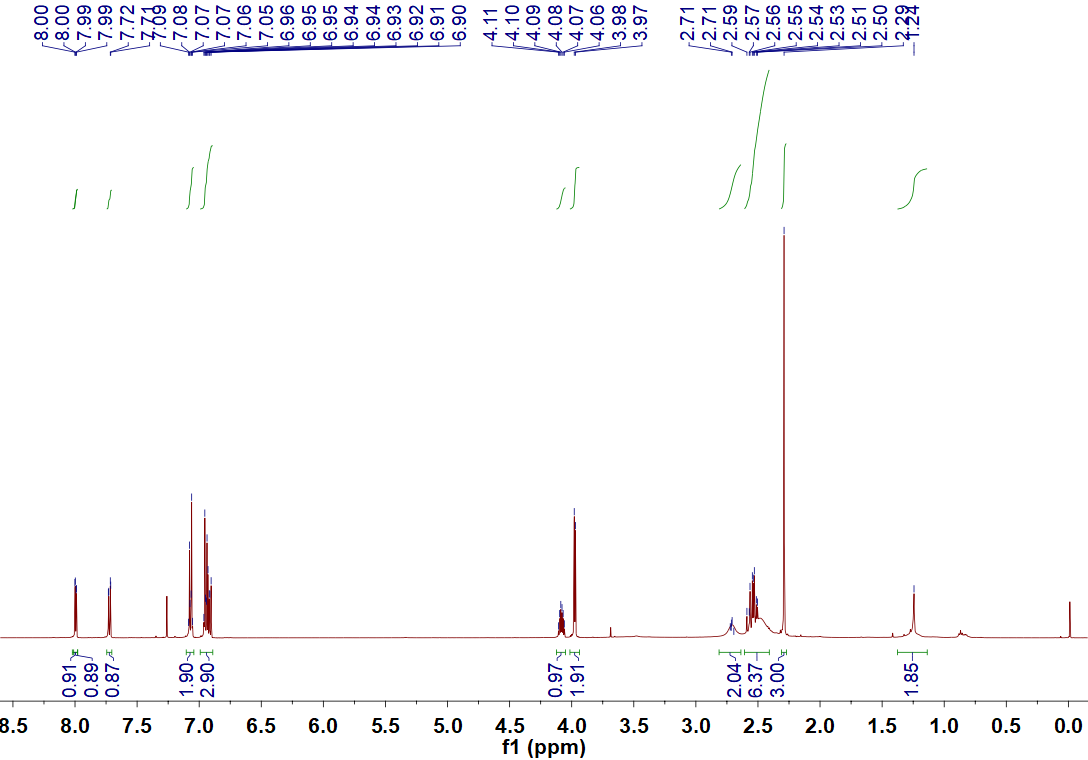
Figure S56.** ^1^H NMR Spectrum (CDCl_3_, 500 MHz) of compound PyE14.

**
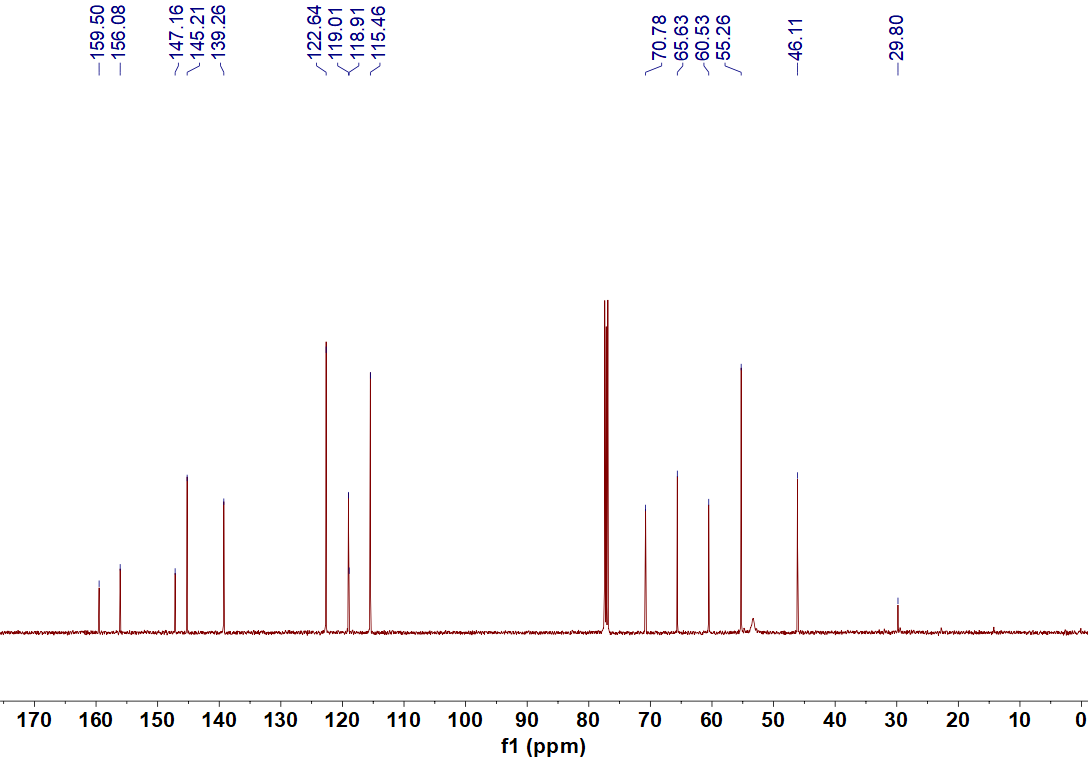
Figure S57.** ^13^C NMR Spectrum (CDCl_3_, 126 MHz) of compound PyE14.

**
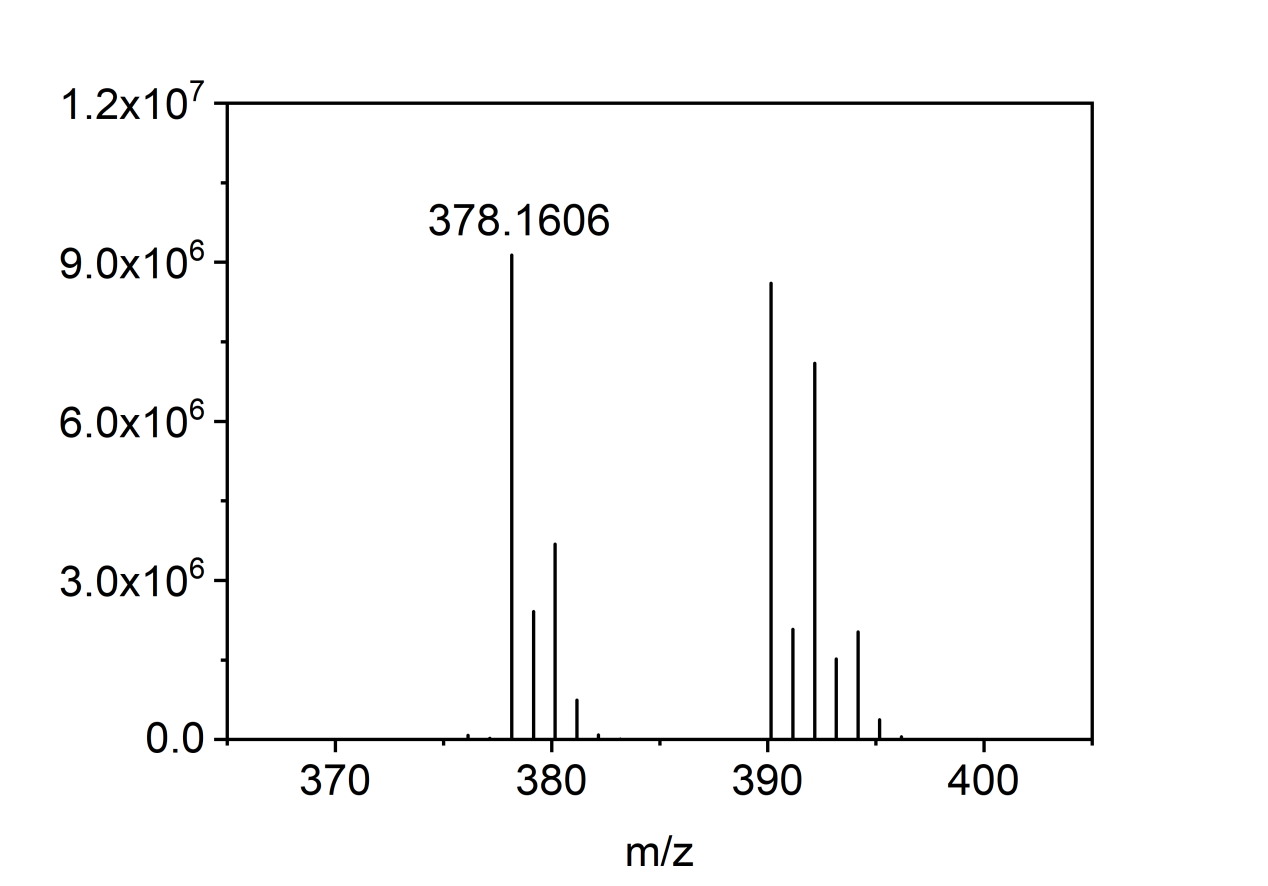
Figure S58.** HRMS Spectrum of compound PyE14.

**
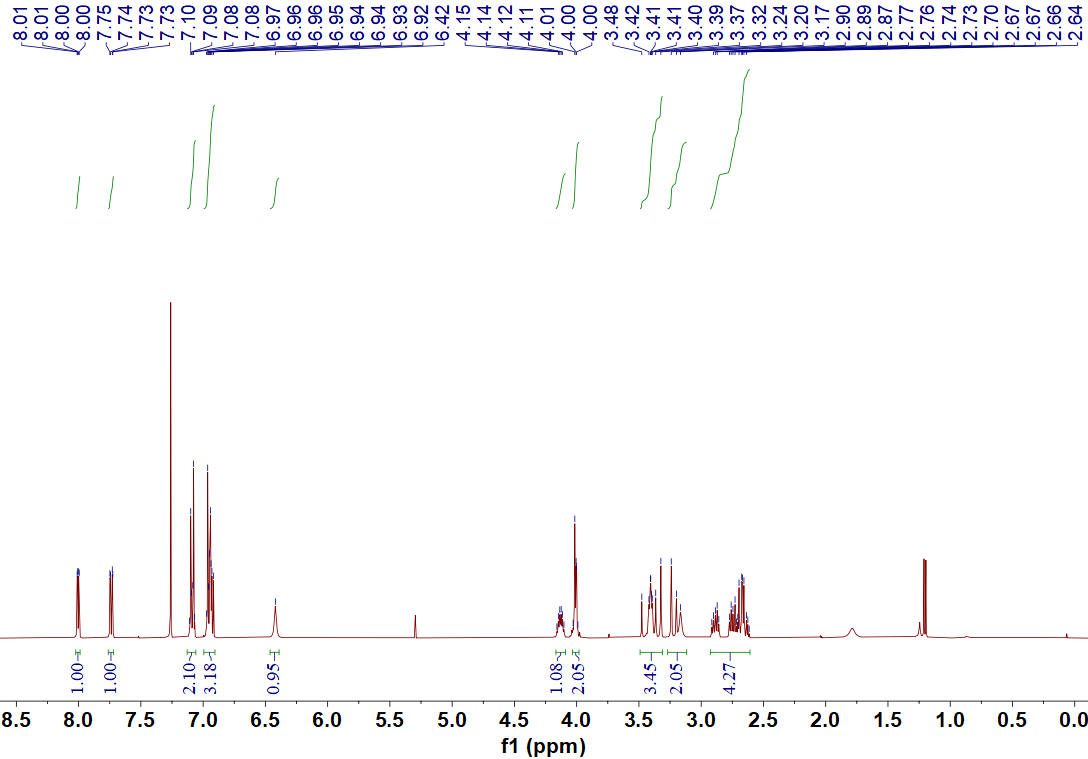
Figure S59.** ^1^H NMR Spectrum (CDCl_3_, 500 MHz) of compound PyE15.

**
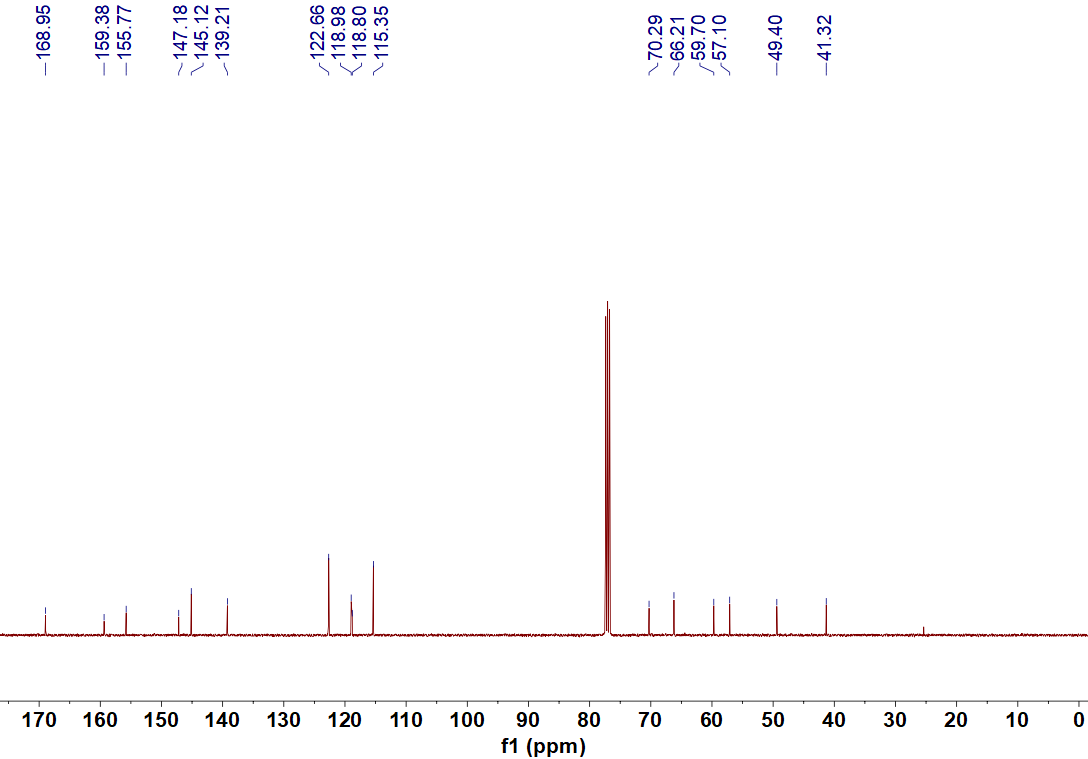
Figure S60.** ^13^C NMR Spectrum (CDCl_3_, 101 MHz) of compound PyE15.

**
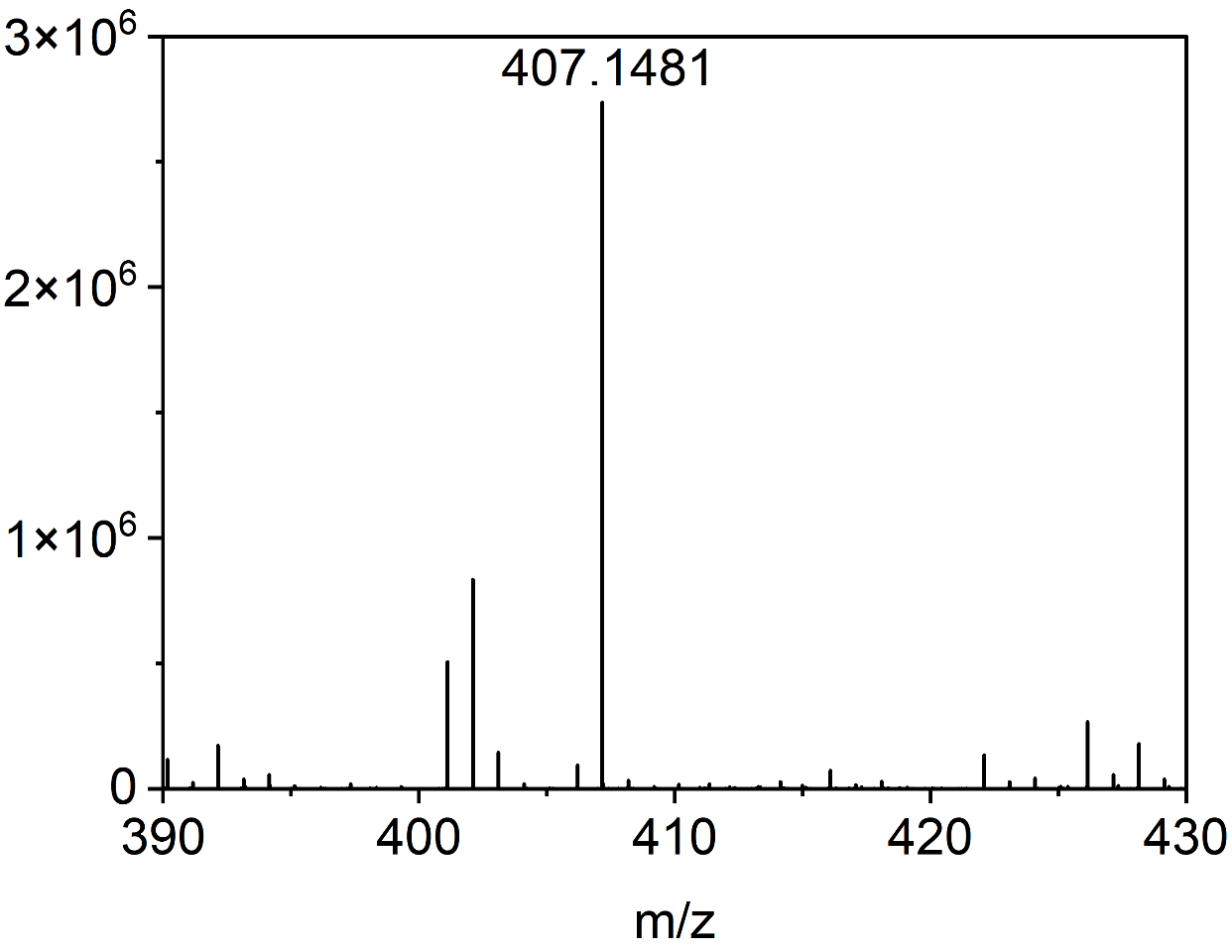
**

**Figure S61.** HRMS Spectrum of compound PyE15.

**
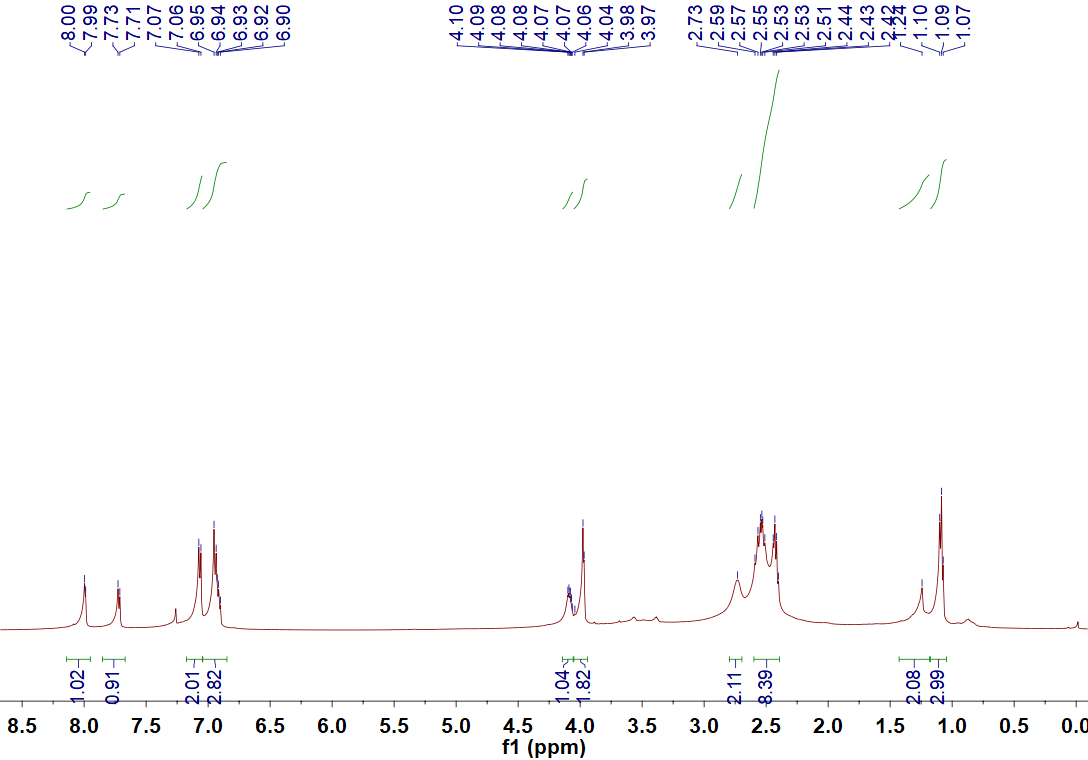
Figure S62.** ^1^H NMR Spectrum (CDCl_3_, 500 MHz) of compound PyE16.

**
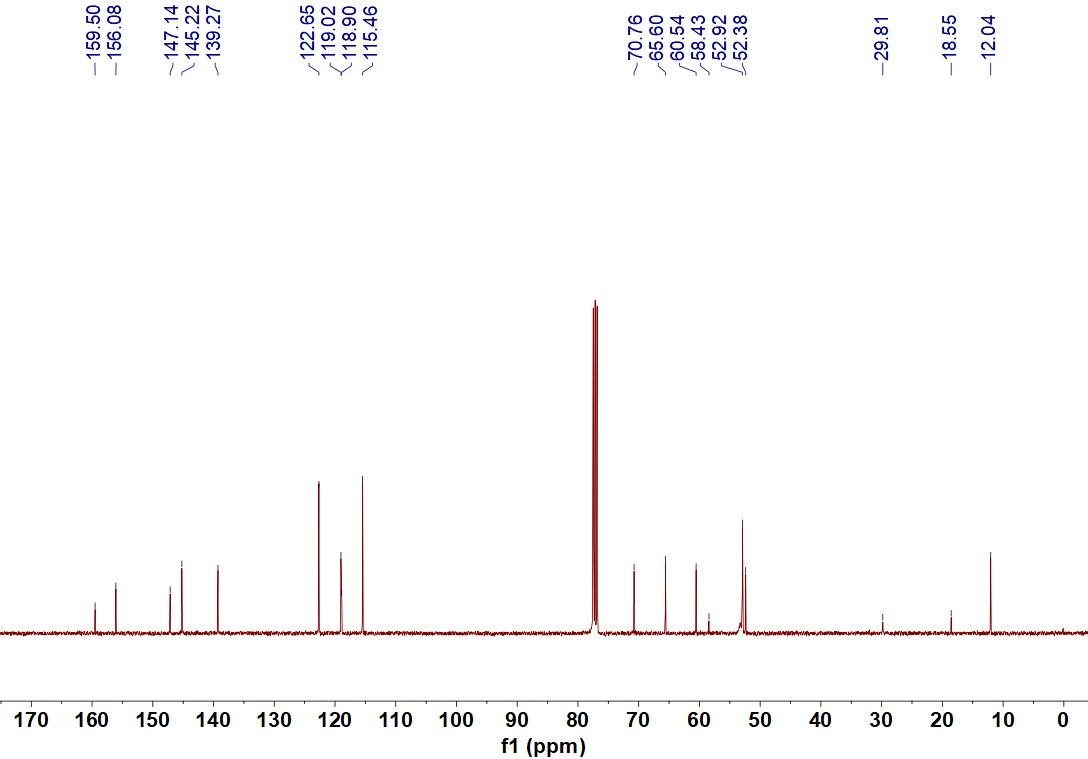
Figure S63.** ^13^C NMR Spectrum (CDCl_3_, 101 MHz) of compound PyE16.

**Figure S64.** HRMS Spectrum of compound PyE16.

**
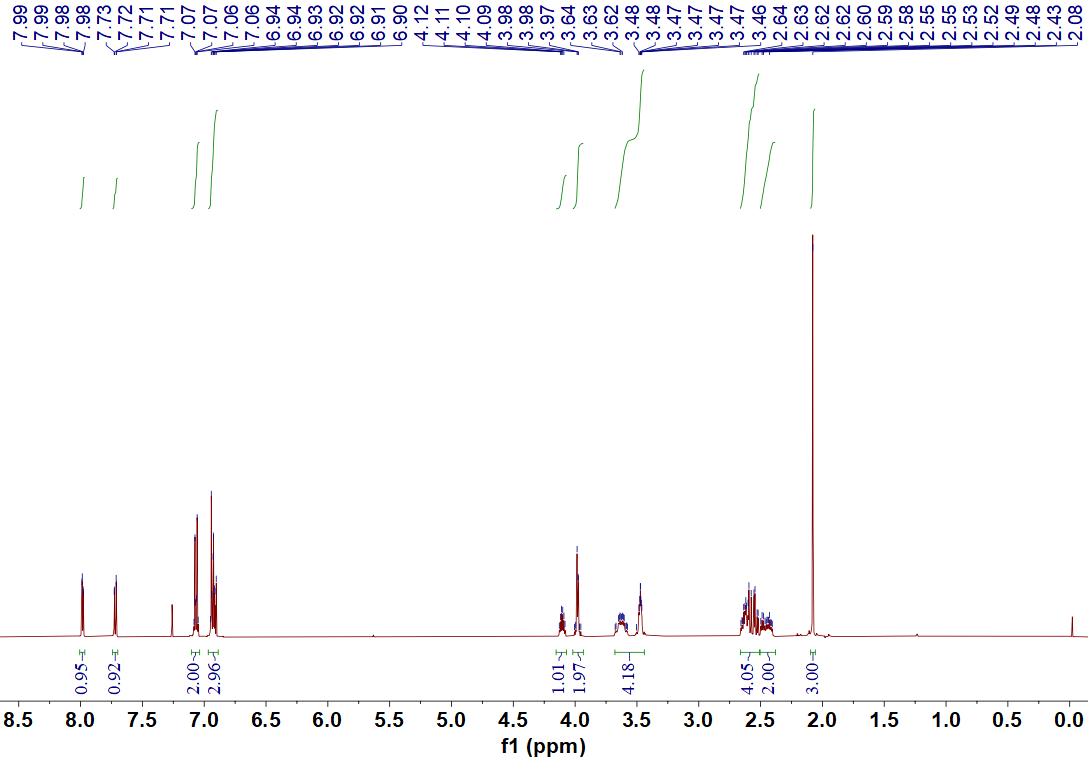
Figure S65.** ^1^H NMR Spectrum (CDCl_3_, 500 MHz) of compound PyE17.

**
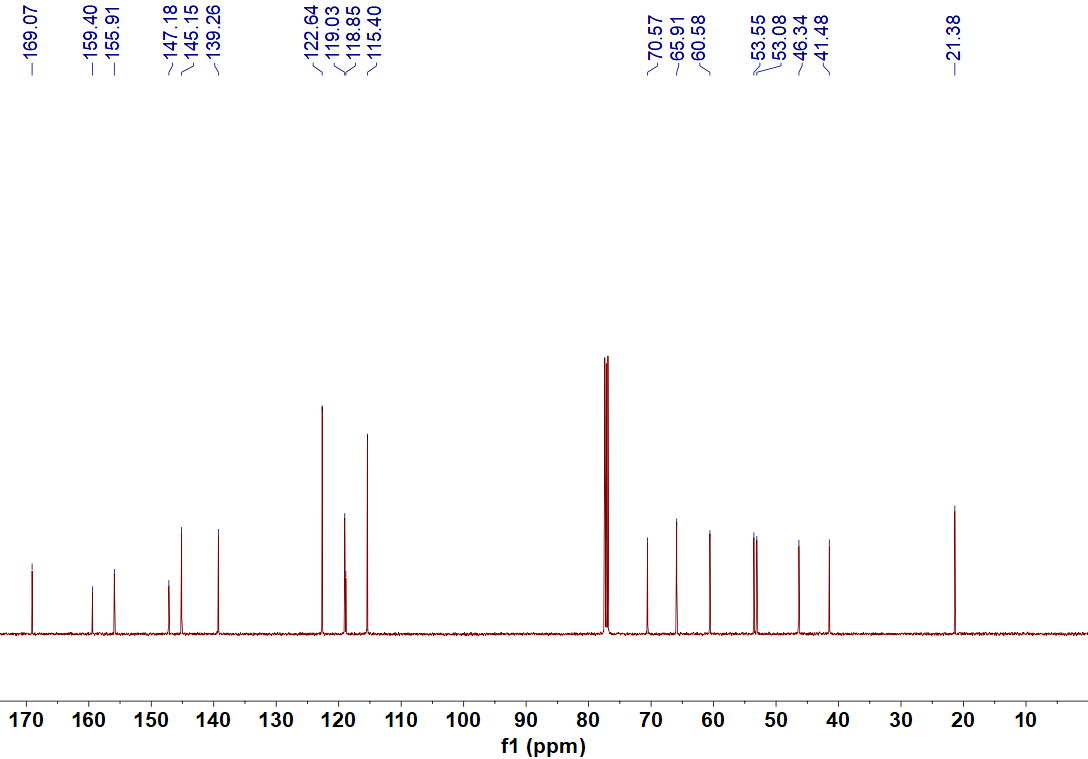
**

**Figure S66.** ^13^C NMR Spectrum (CDCl_3_, 126 MHz) of compound PyE17.

**Figure S67.** HRMS Spectrum of compound PyE17.

**
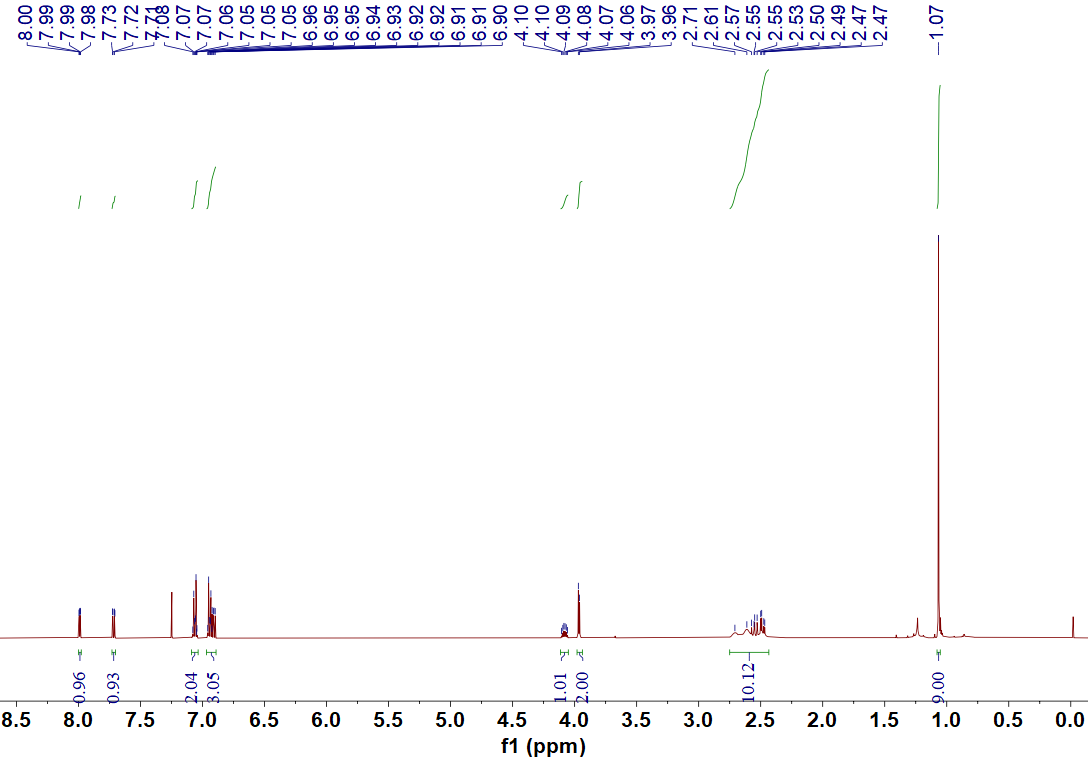
Figure S68.** ^1^H NMR Spectrum (CDCl_3_, 500 MHz) of compound PyE18.

**
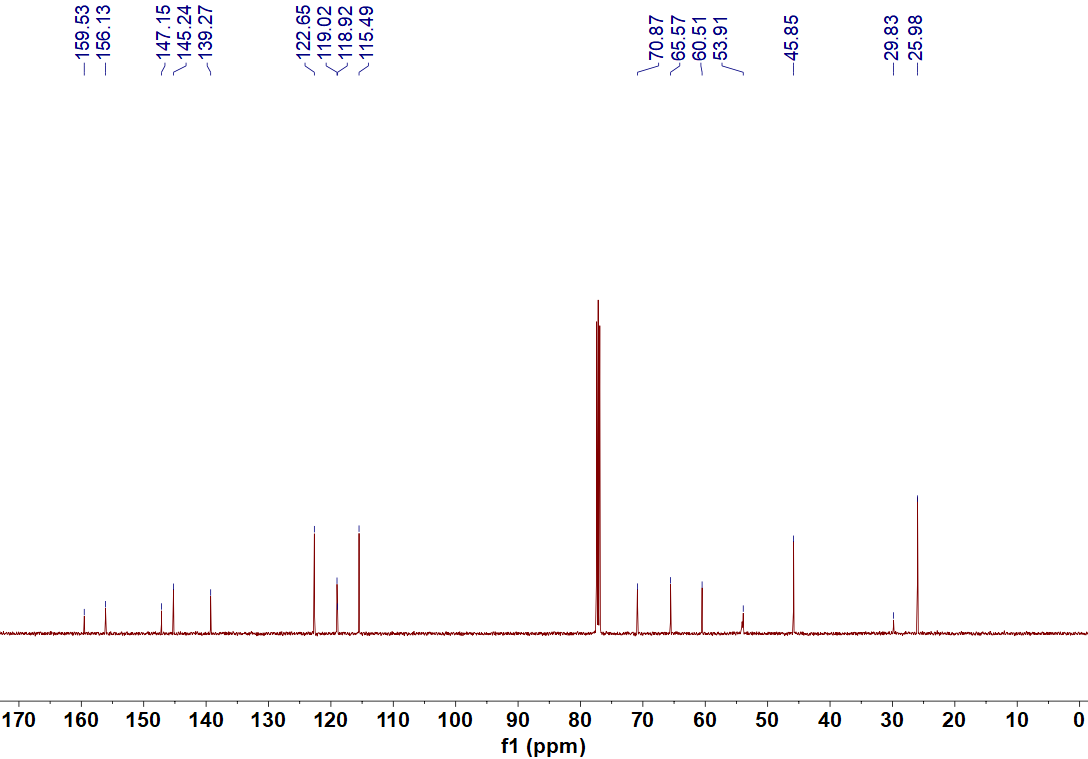
Figure S69.** ^13^C NMR Spectrum (CDCl_3_, 126 MHz) of compound PyE18.

**Figure S70.** HRMS Spectrum of compound PyE18.

**
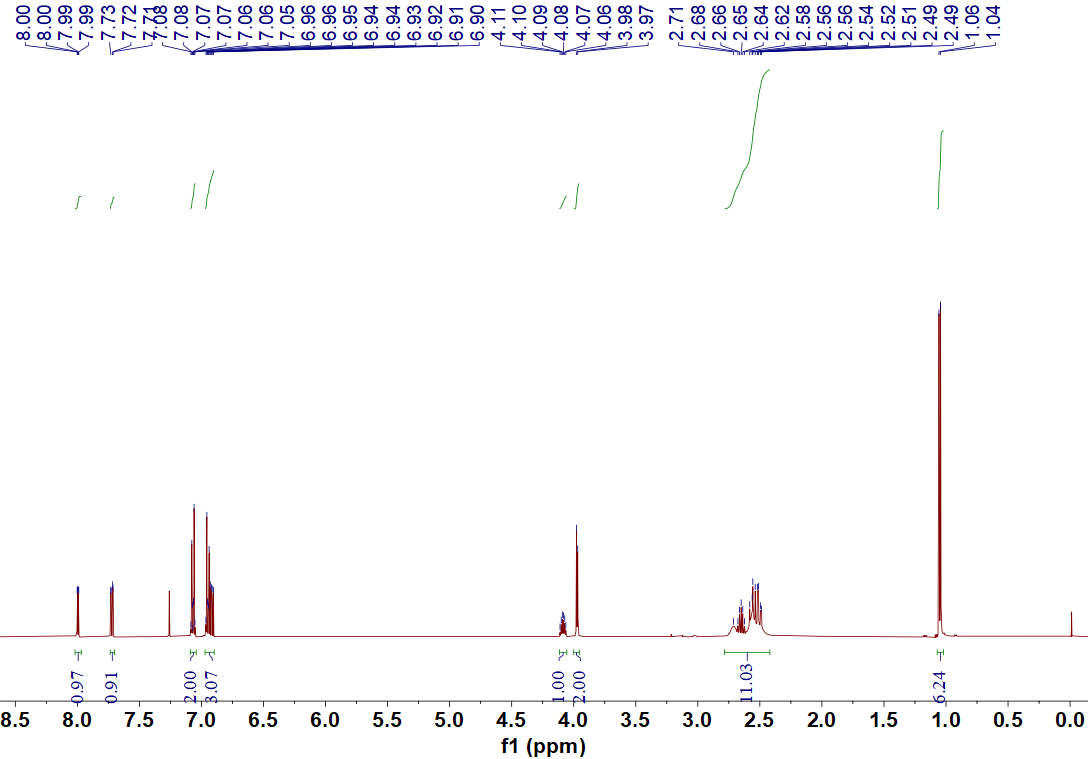
Figure S71.** ^1^H NMR Spectrum (CDCl_3_, 500 MHz) of compound PyE19.

**
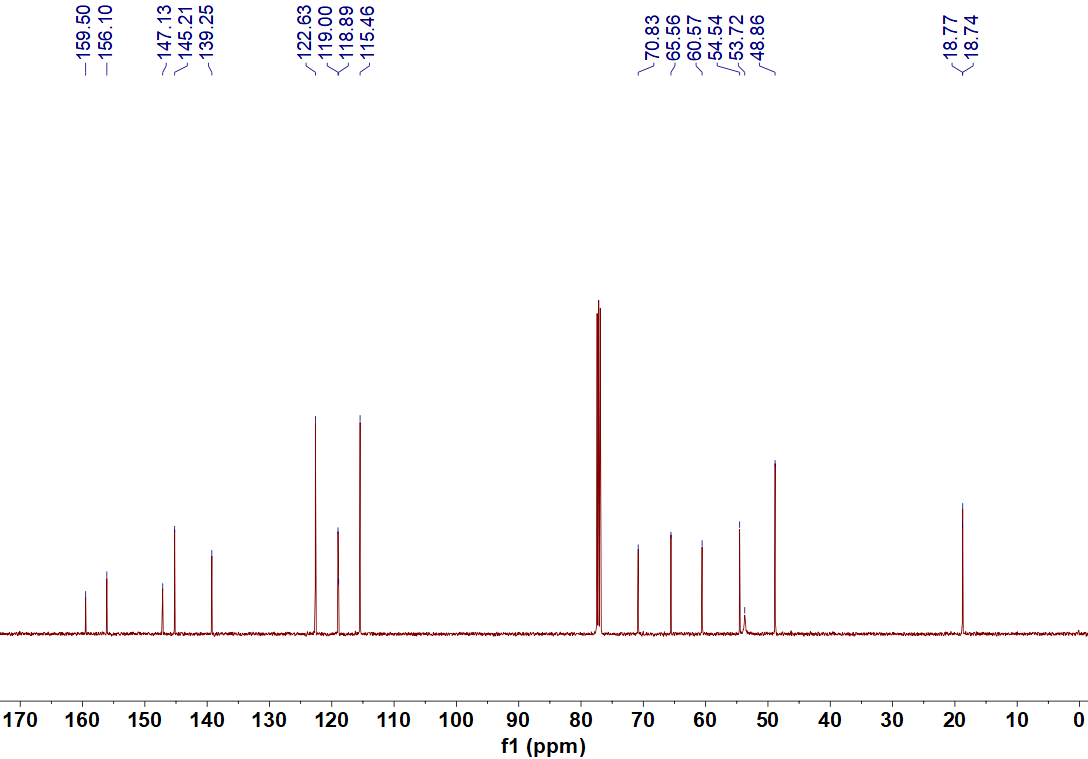
Figure S72.** ^13^C NMR Spectrum (CDCl_3_, 126 MHz) of compound PyE19.

**
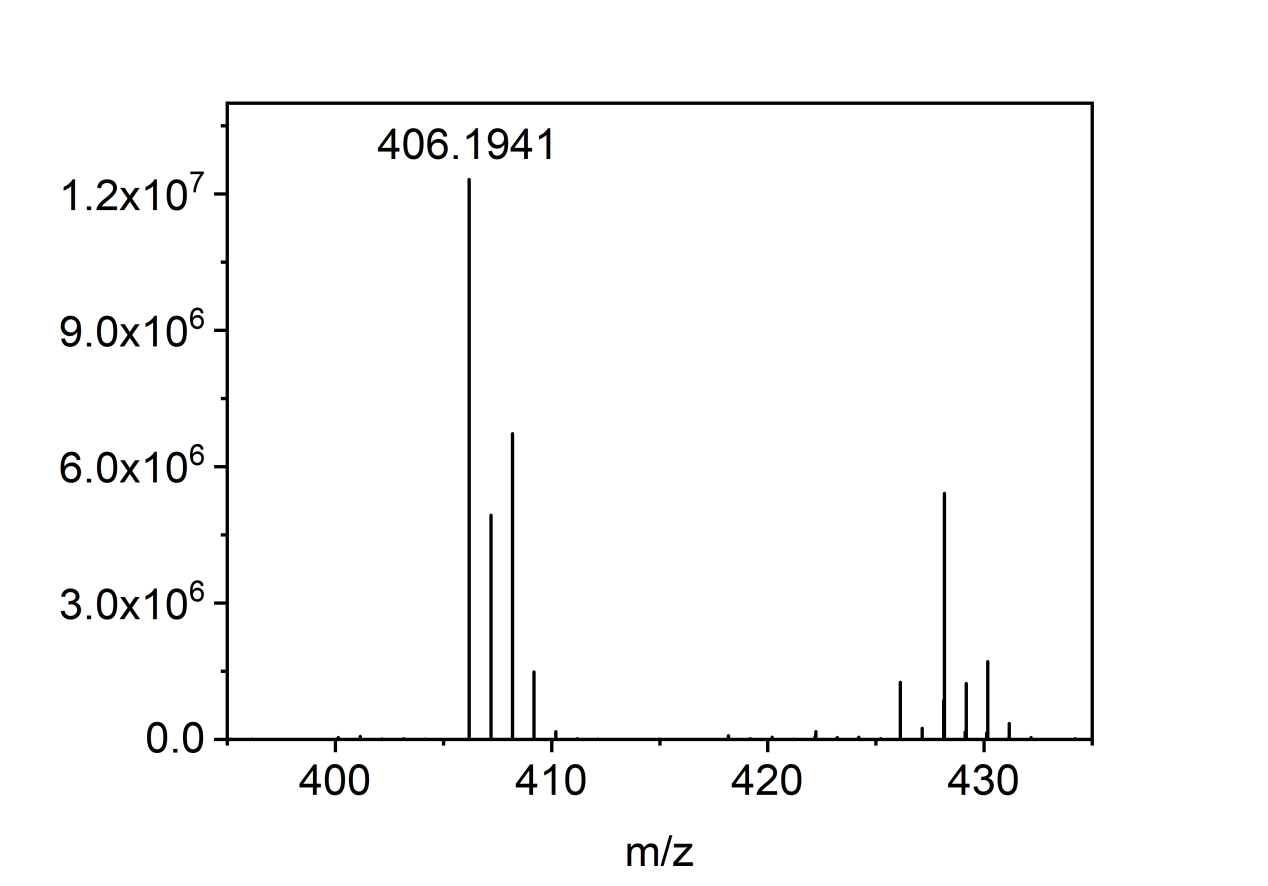
Figure S73.** HRMS Spectrum of compound PyE19.

**
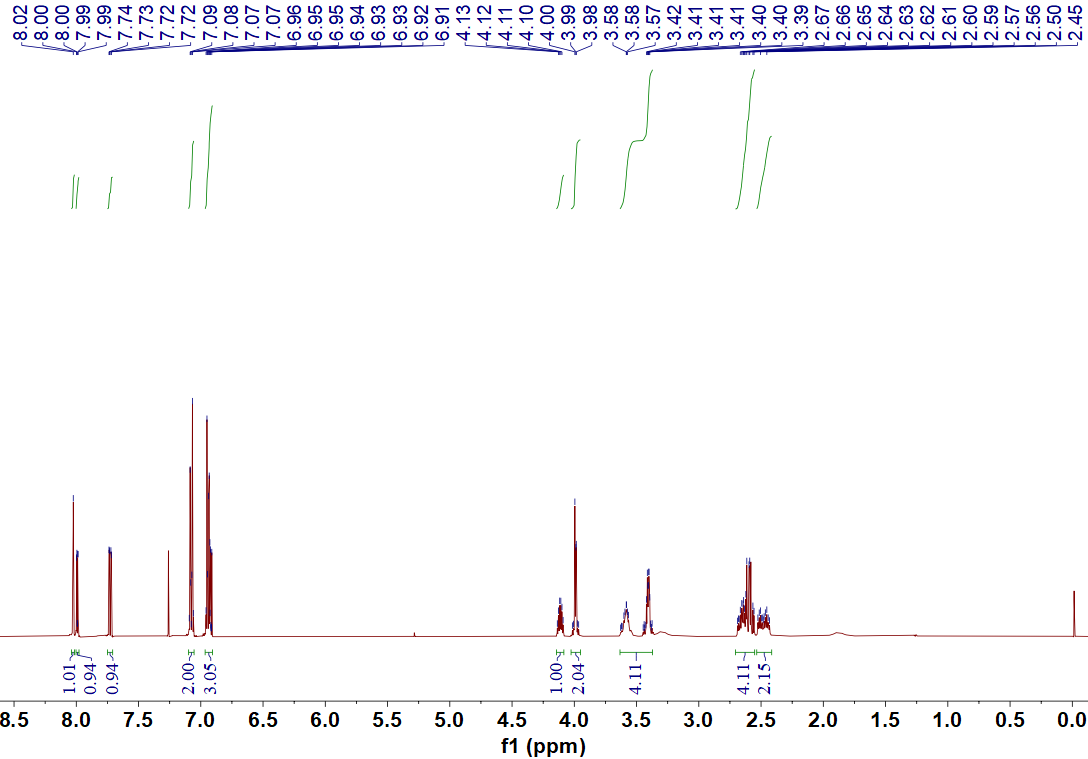
Figure S74.** ^1^H NMR Spectrum (CDCl_3_, 500 MHz) of compound PyE20.

**
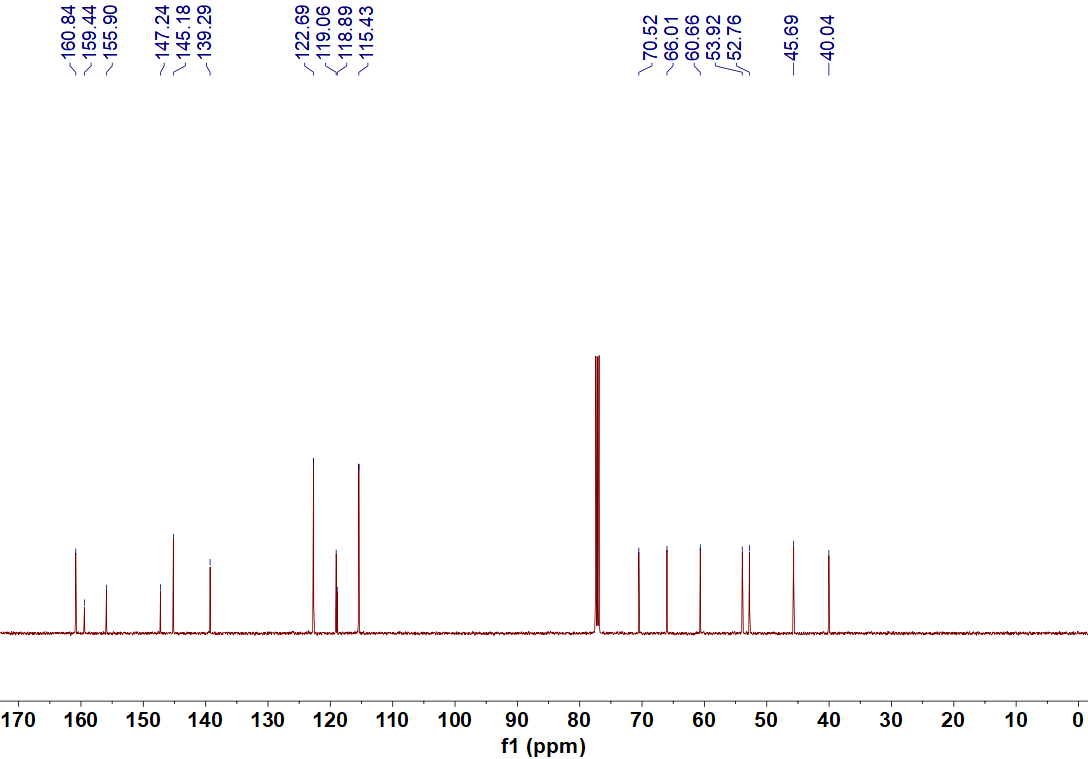
Figure S75.** ^13^C NMR Spectrum (CDCl_3_, 126 MHz) of compound PyE20.

**
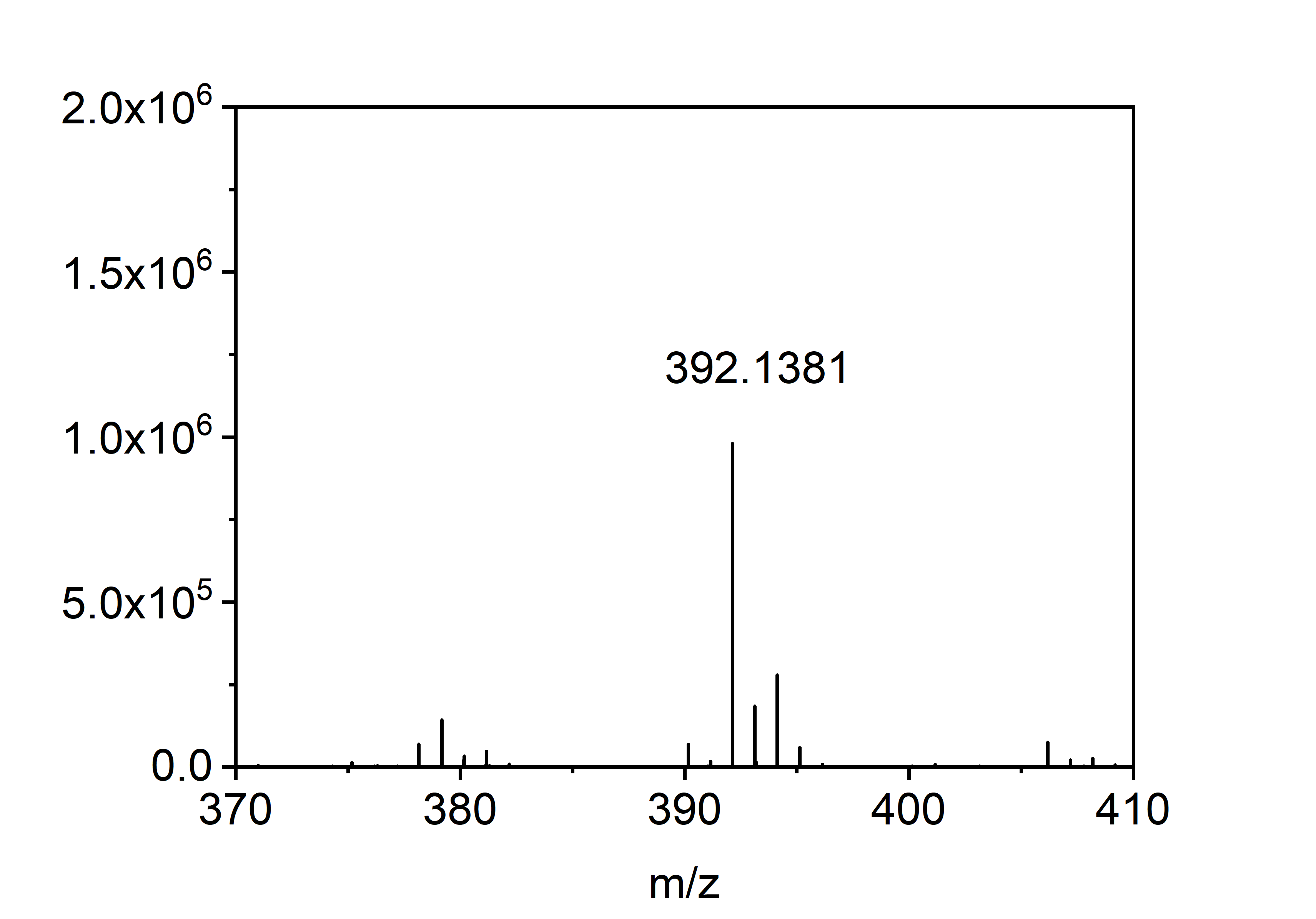
Figure S76.** HRMS Spectrum of compound PyE20.

**
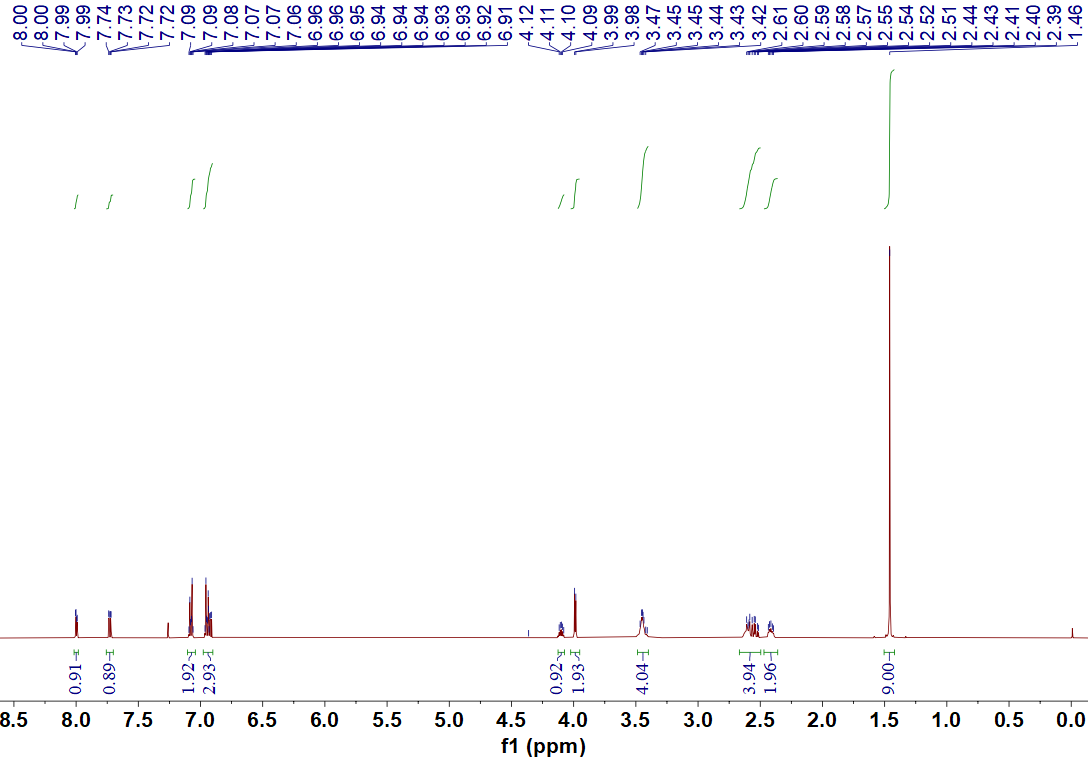
Figure S77.** ^1^H NMR Spectrum (CDCl_3_, 500 MHz) of compound PyE21.

**
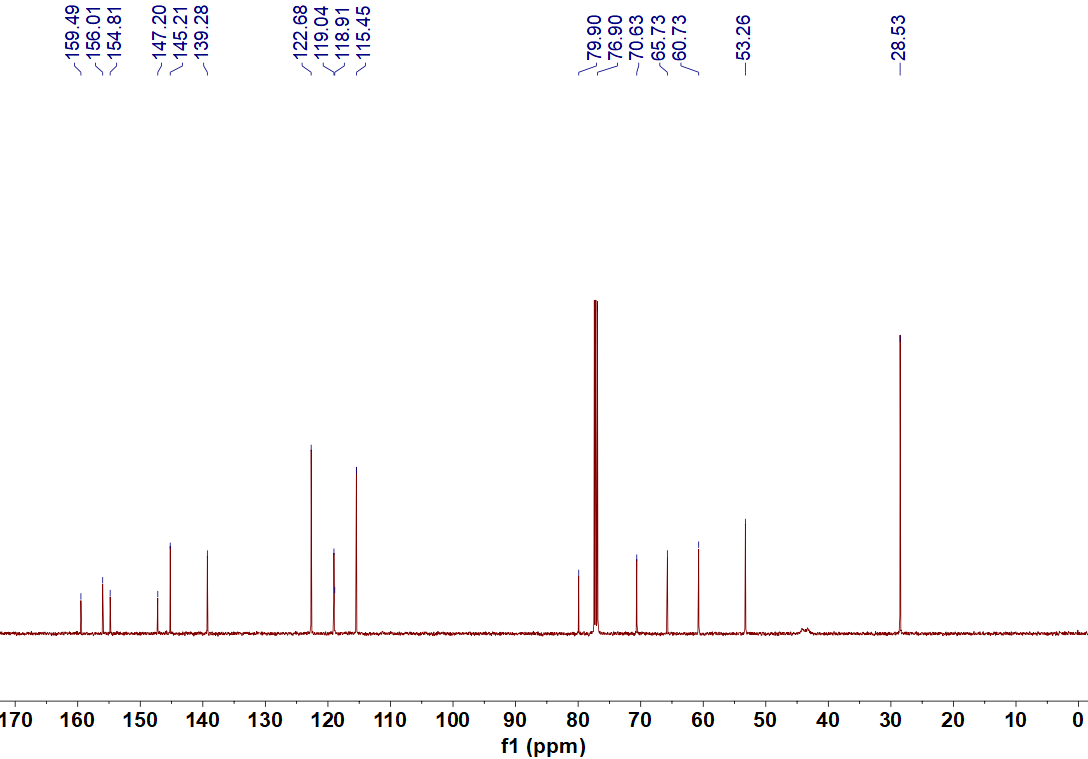
Figure S78.** ^13^C NMR Spectrum (CDCl_3_, 126 MHz) of compound PyE21.

**
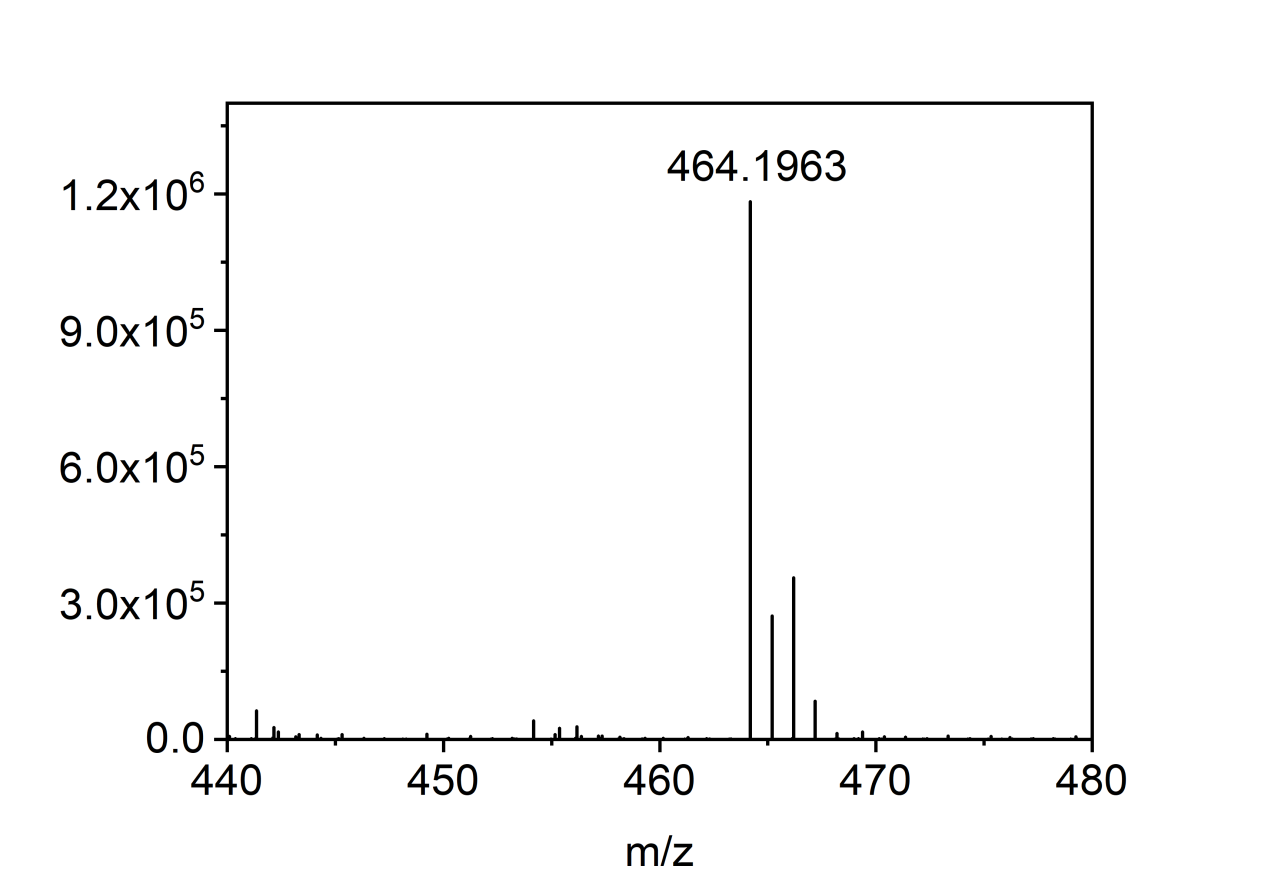
Figure S79.** HRMS Spectrum of compound PyE21.

**
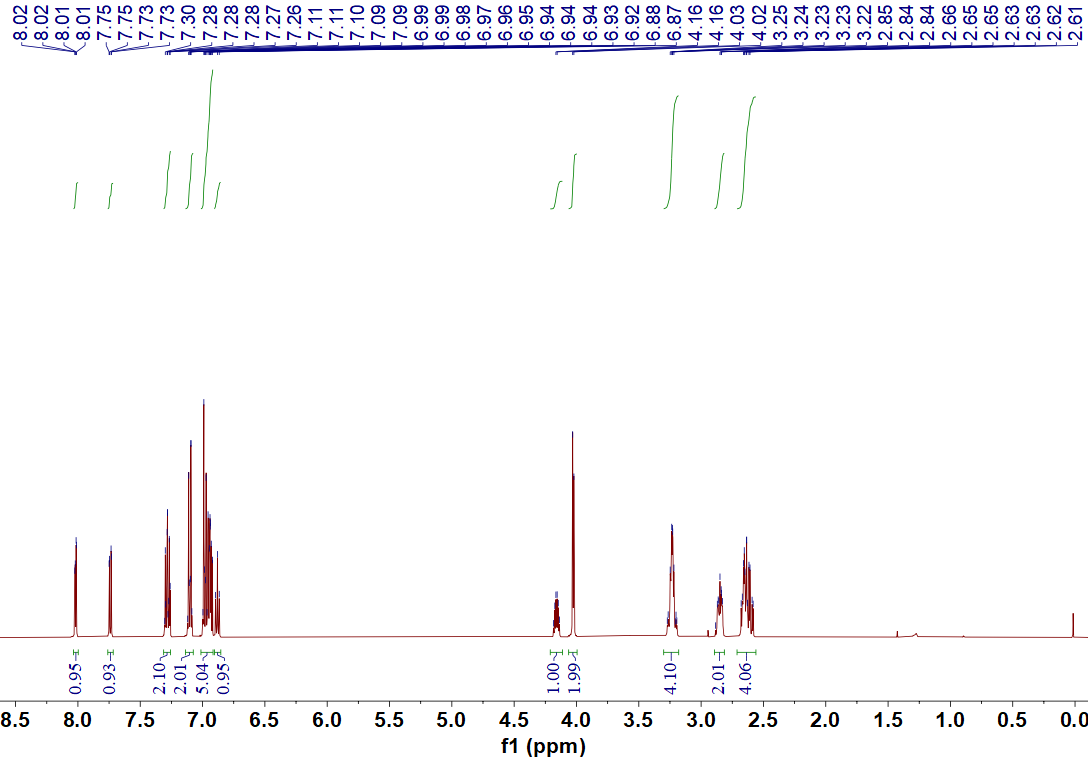
Figure S80.** ^1^H NMR Spectrum (CDCl_3_, 500 MHz) of compound PyE22.

**
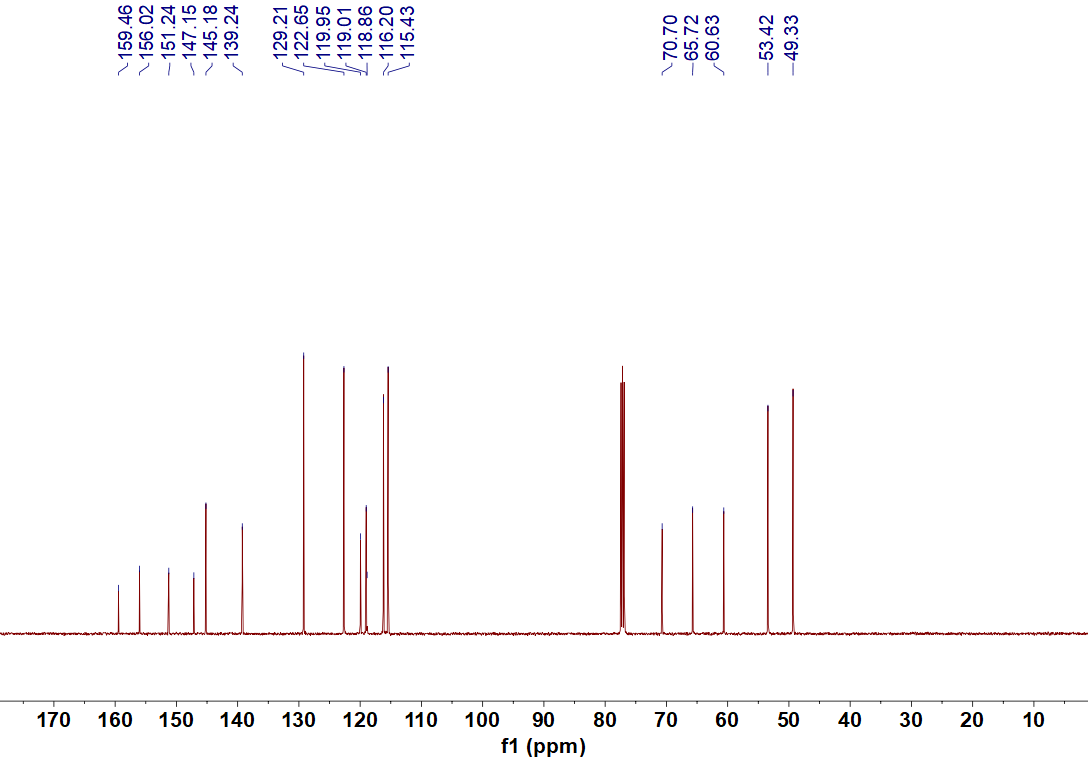
Figure S81.** ^13^C NMR Spectrum (CDCl_3_, 126 MHz) of compound PyE22.

**
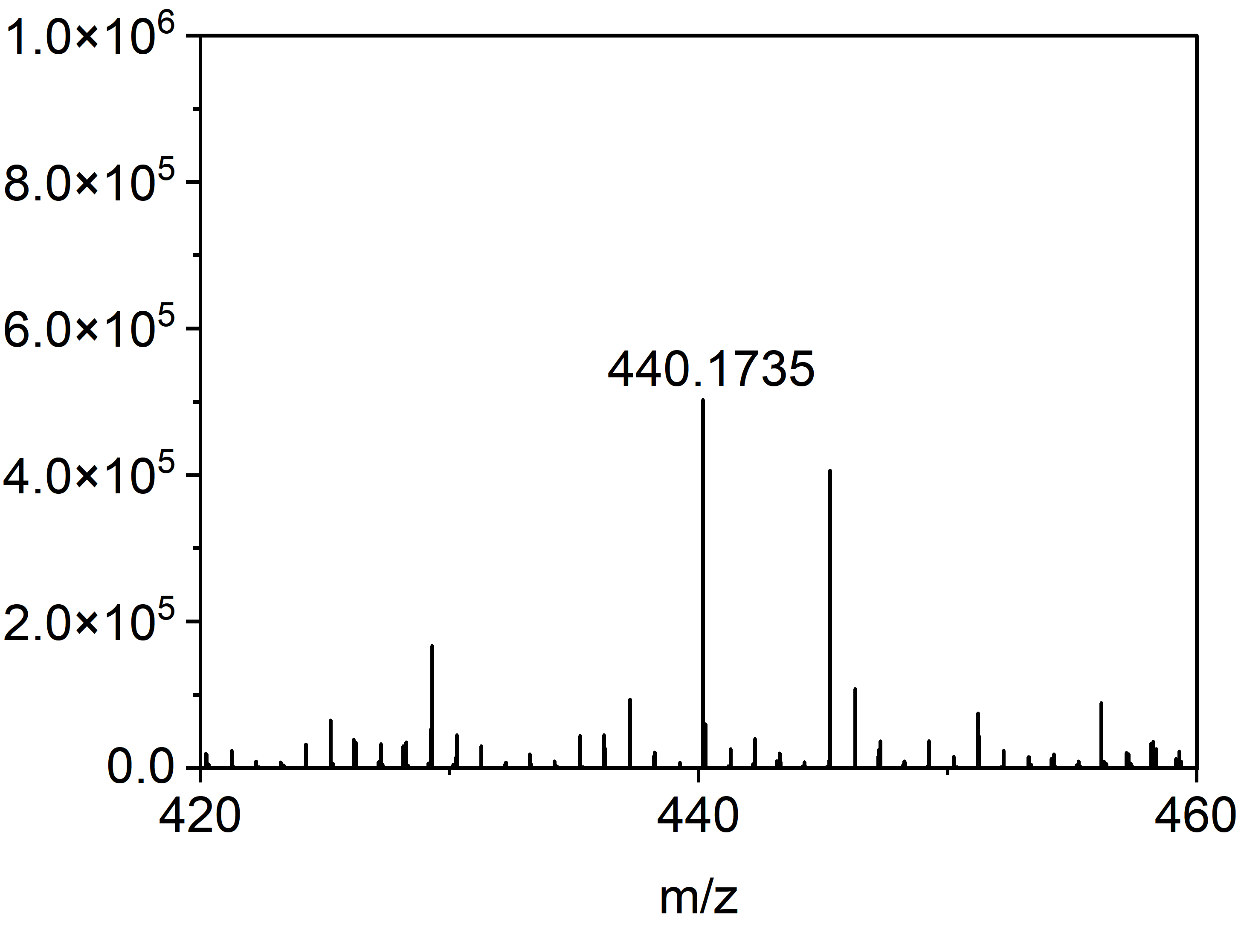
**

**Figure S82.** HRMS Spectrum of compound PyE22.

**
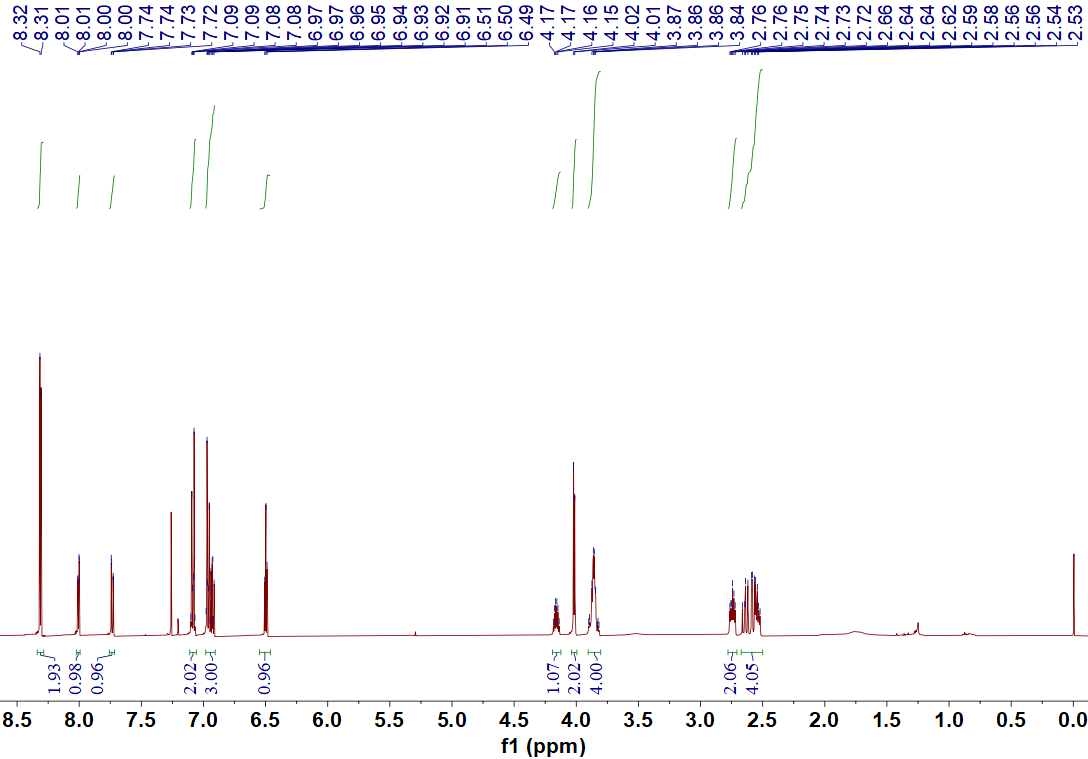
Figure S83.** ^1^H NMR Spectrum (CDCl_3_, 500 MHz) of compound PyE23.

**
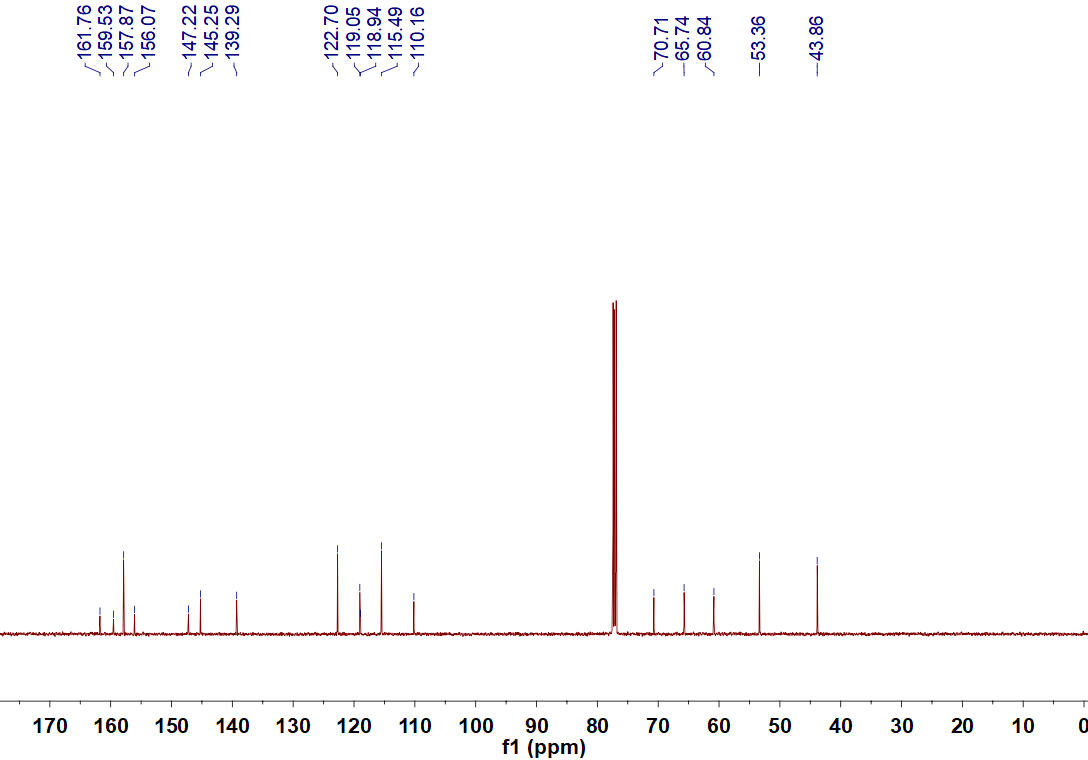
Figure S84.** ^13^C NMR Spectrum (CDCl_3_, 126 MHz) of compound PyE23.

**_
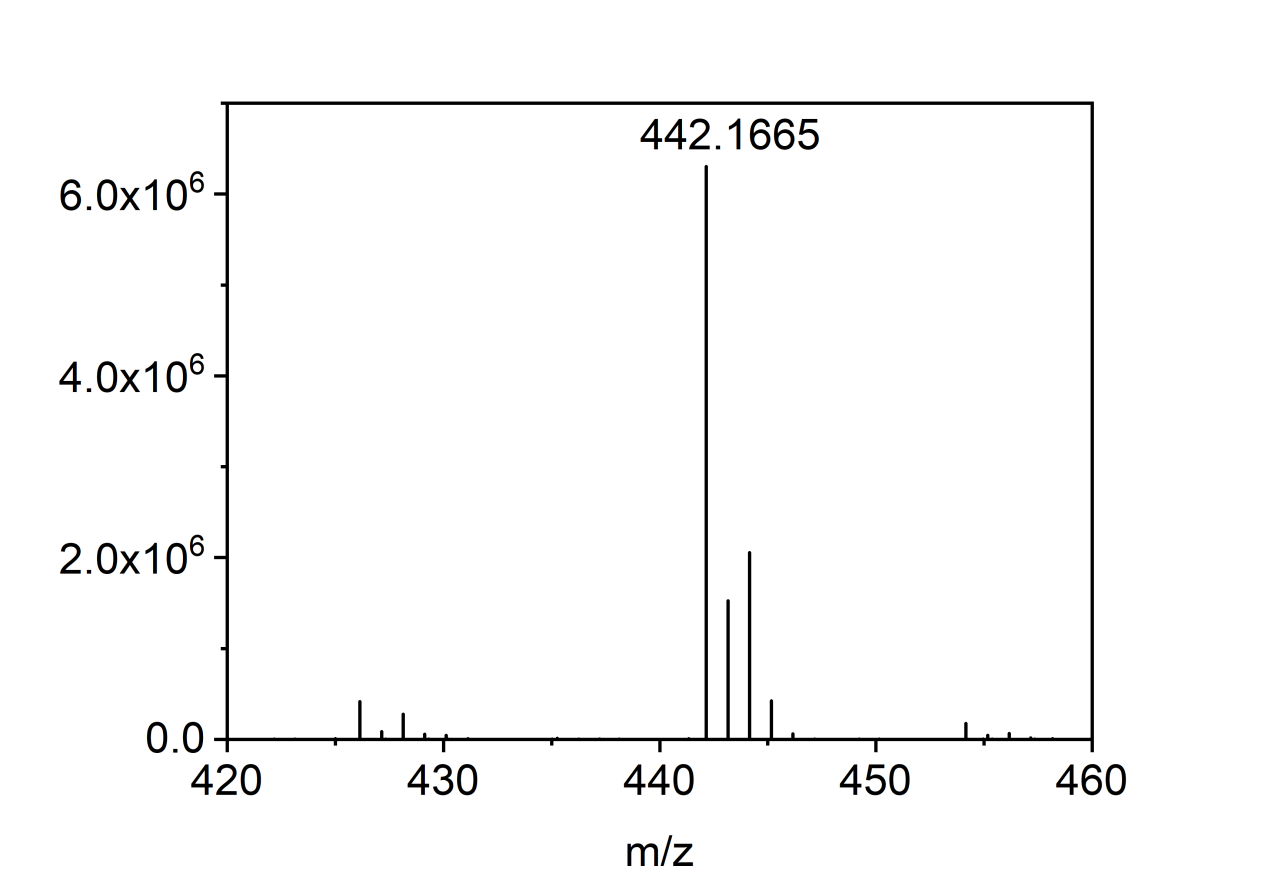
_Figure S85.** HRMS Spectrum of compound PyE23.

**
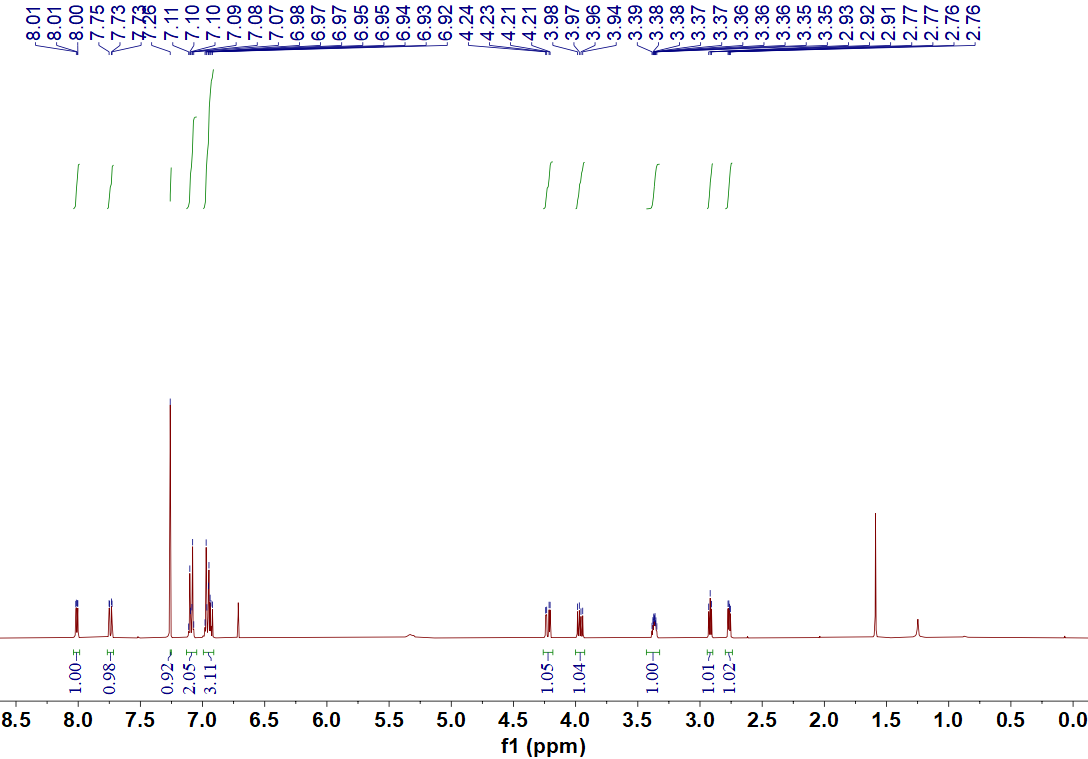
Figure S86.** ^1^H NMR Spectrum (CDCl_3_, 400 MHz) of compound PyE24.

**
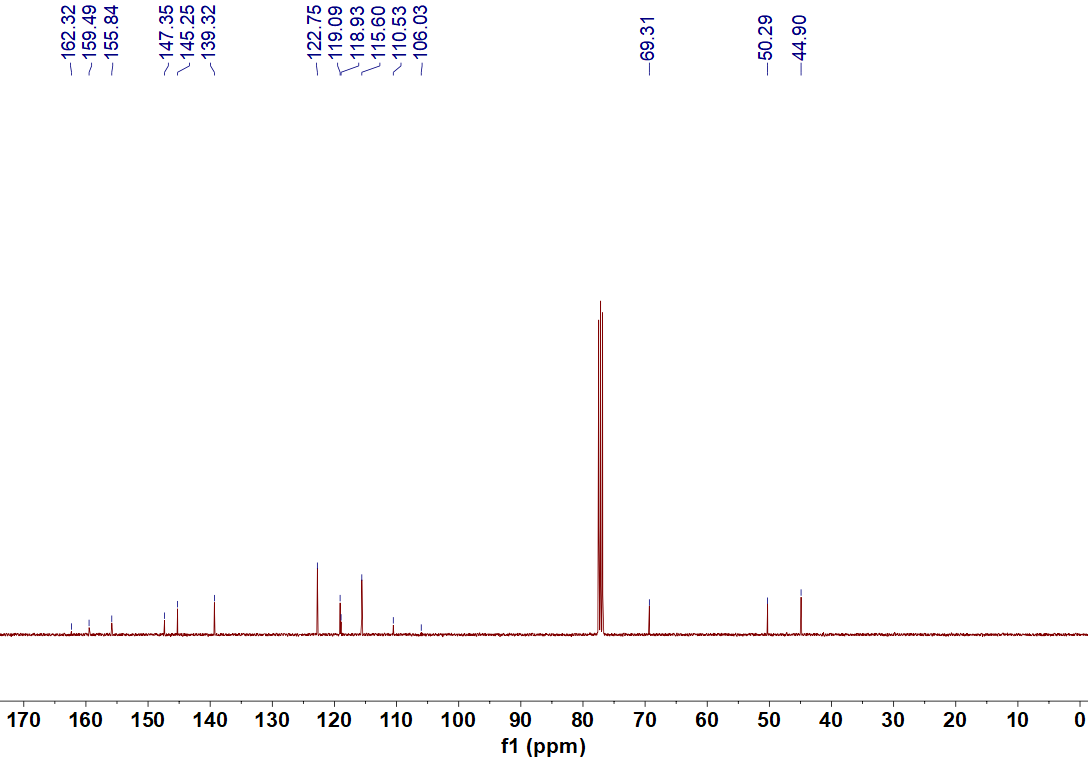
Figure S87.** ^13^C NMR Spectrum (CDCl_3_, 101 MHz) of compound PyE24.

**
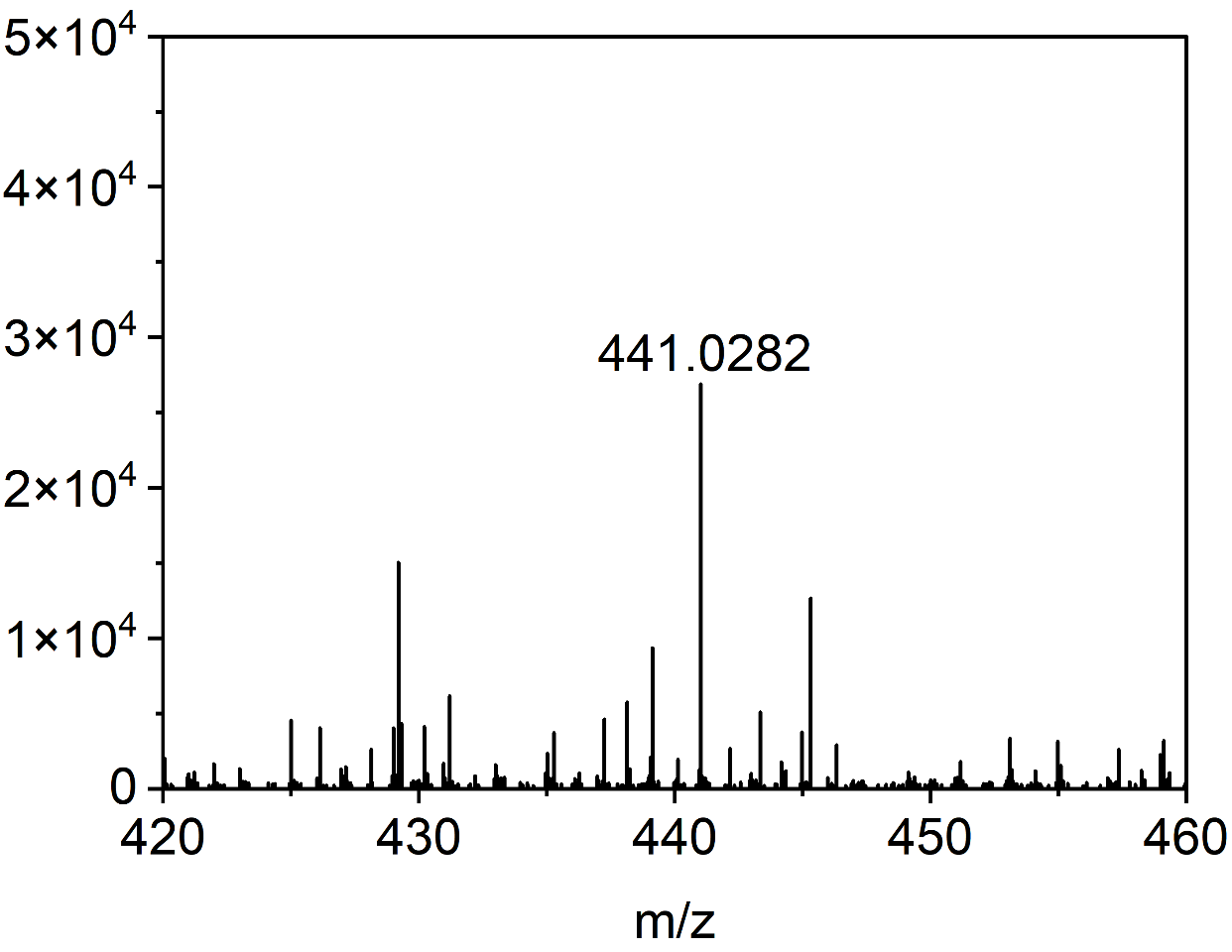
**

**Figure S88.** HRMS Spectrum of compound PyE24.

**
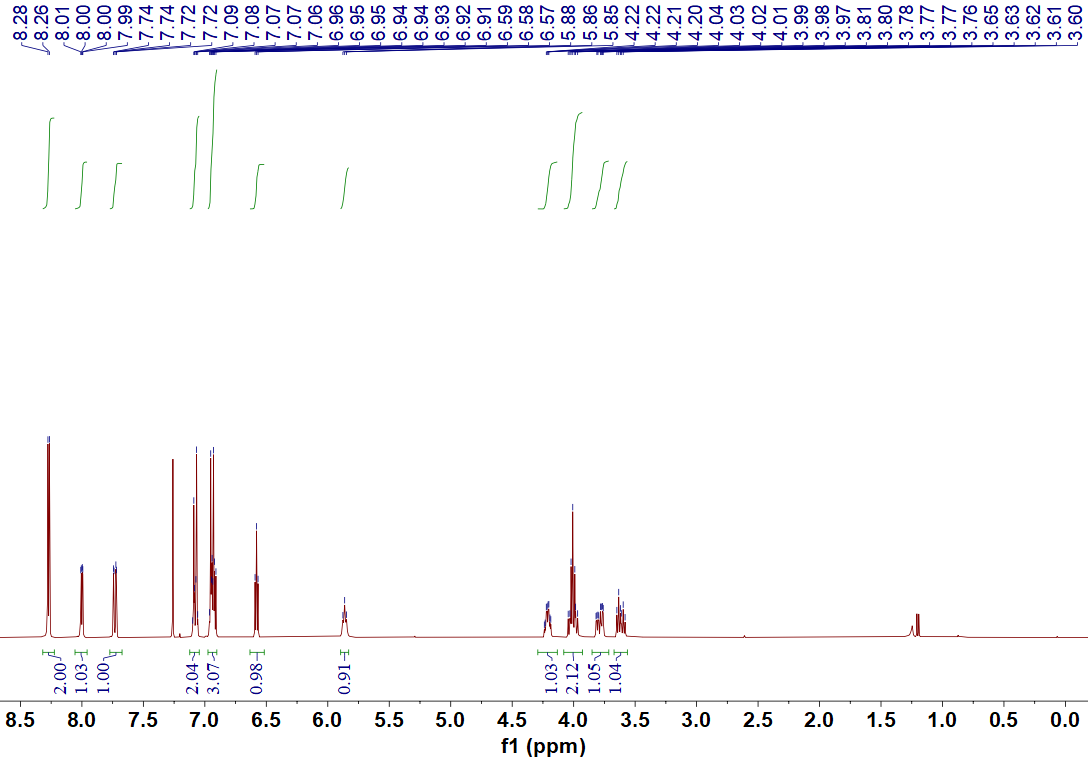
Figure S89.** ^1^H NMR Spectrum (CDCl_3_, 400 MHz) of compound PyE25.

**
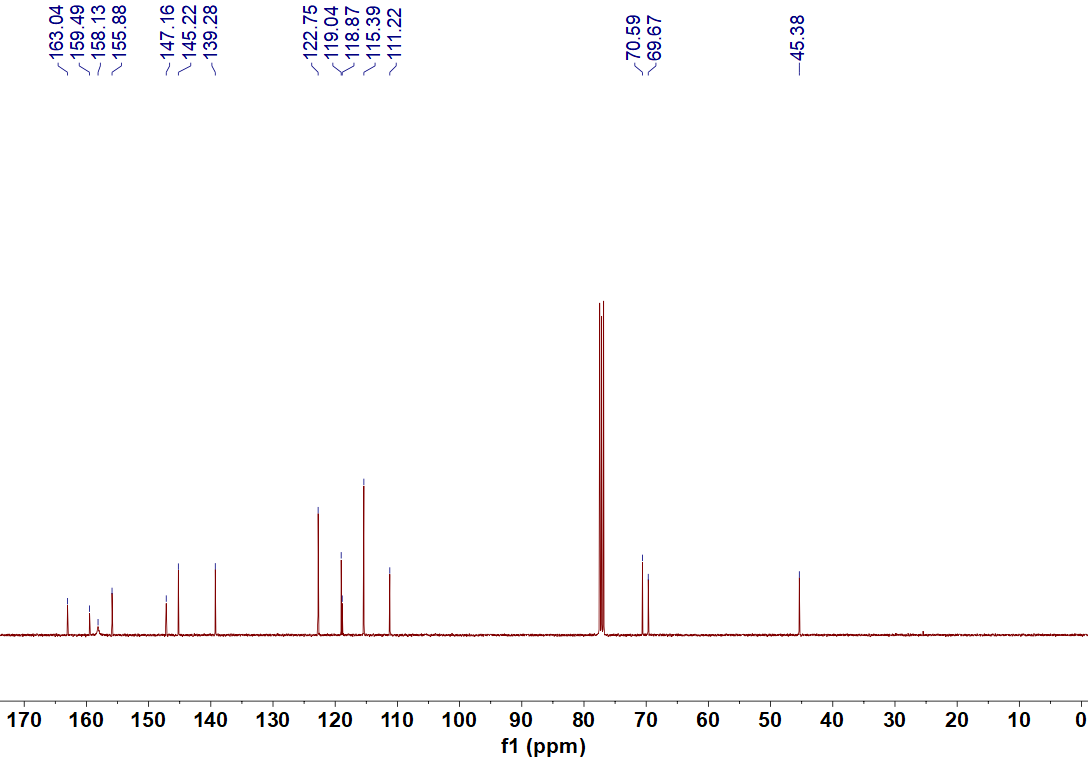
Figure S90.** ^13^C NMR Spectrum (CDCl3, 101 MHz) of compound PyE25.

**
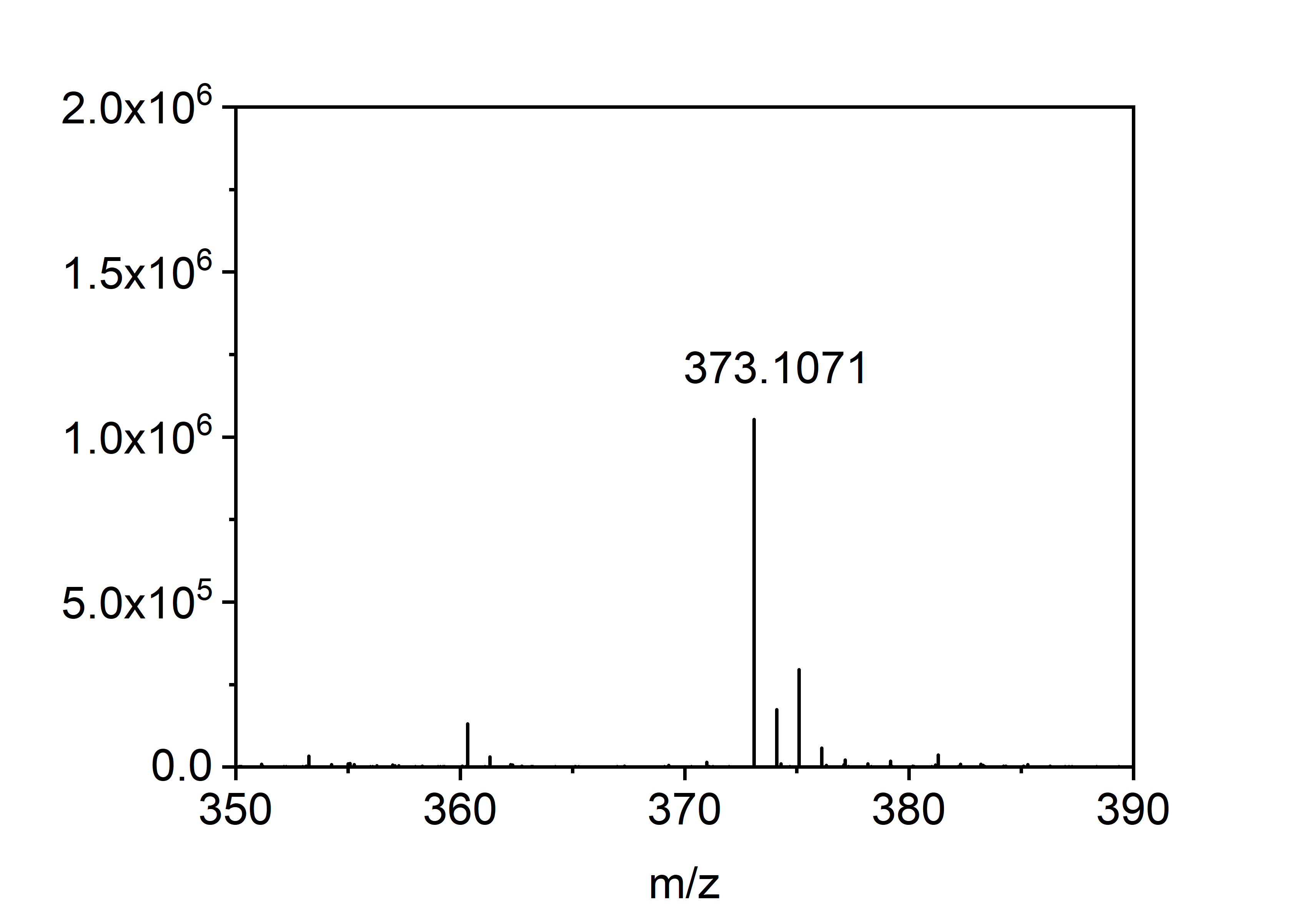
Figure S91.** HRMS Spectrum of compound PyE25.

**
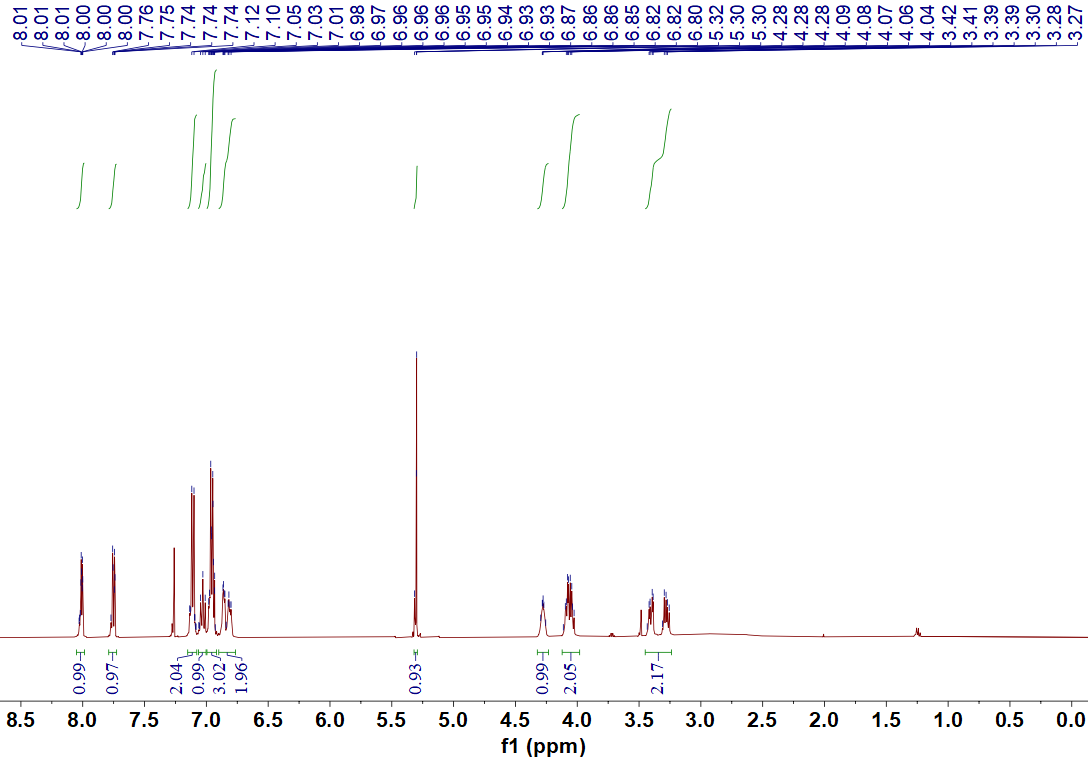
Figure S92.** ^1^H NMR Spectrum (CDCl_3_, 500 MHz) of compound PyE26.

**
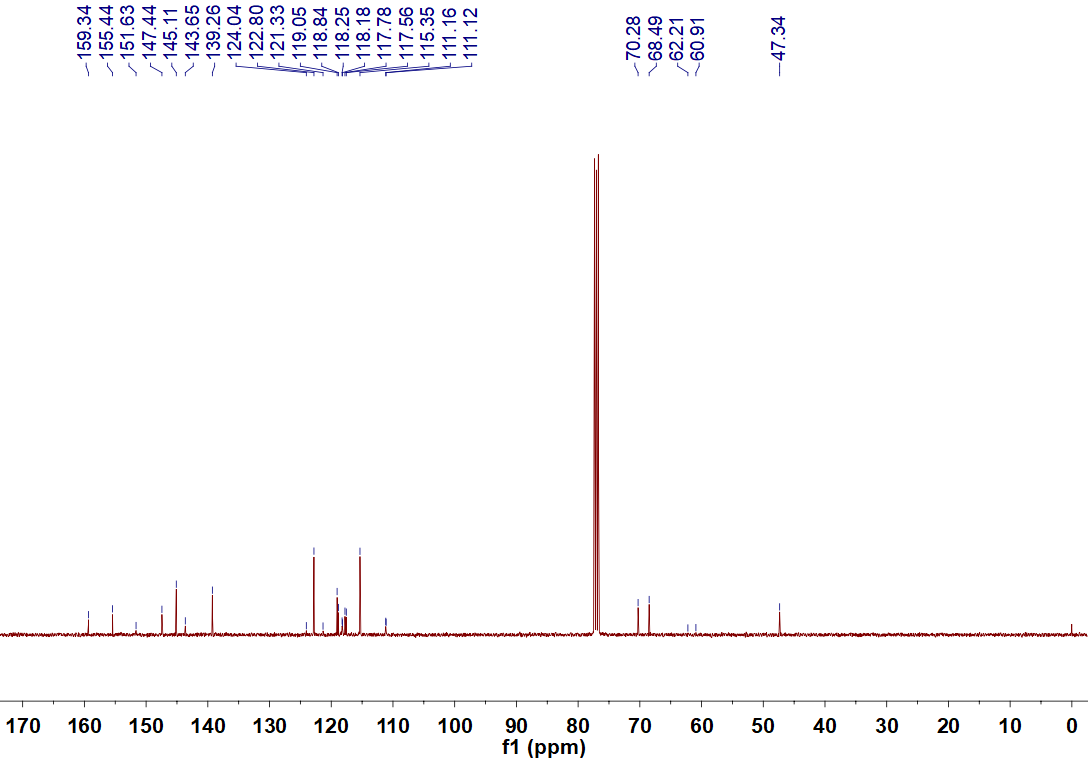
Figure S93.** ^13^C NMR Spectrum (CDCl_3_, 101 MHz) of compound PyE26.

**_
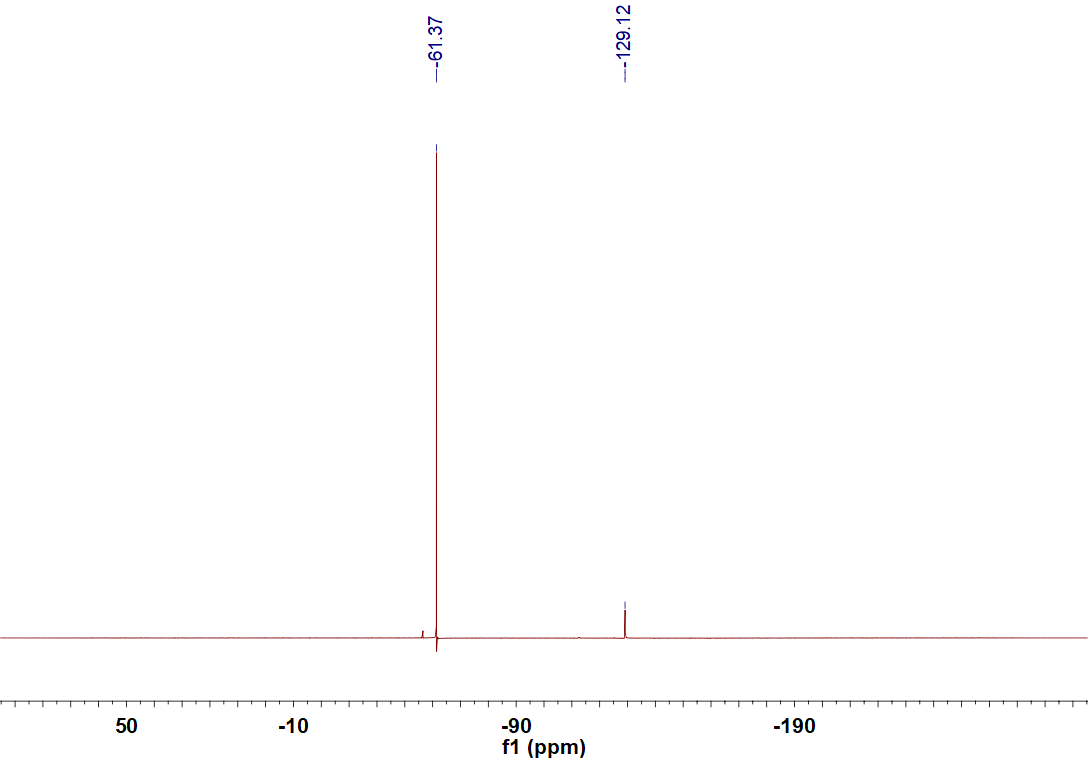
_Figure S94.** ^19^F NMR Spectrum (CDCl_3_, 471 MHz) of compound PyE26.

**
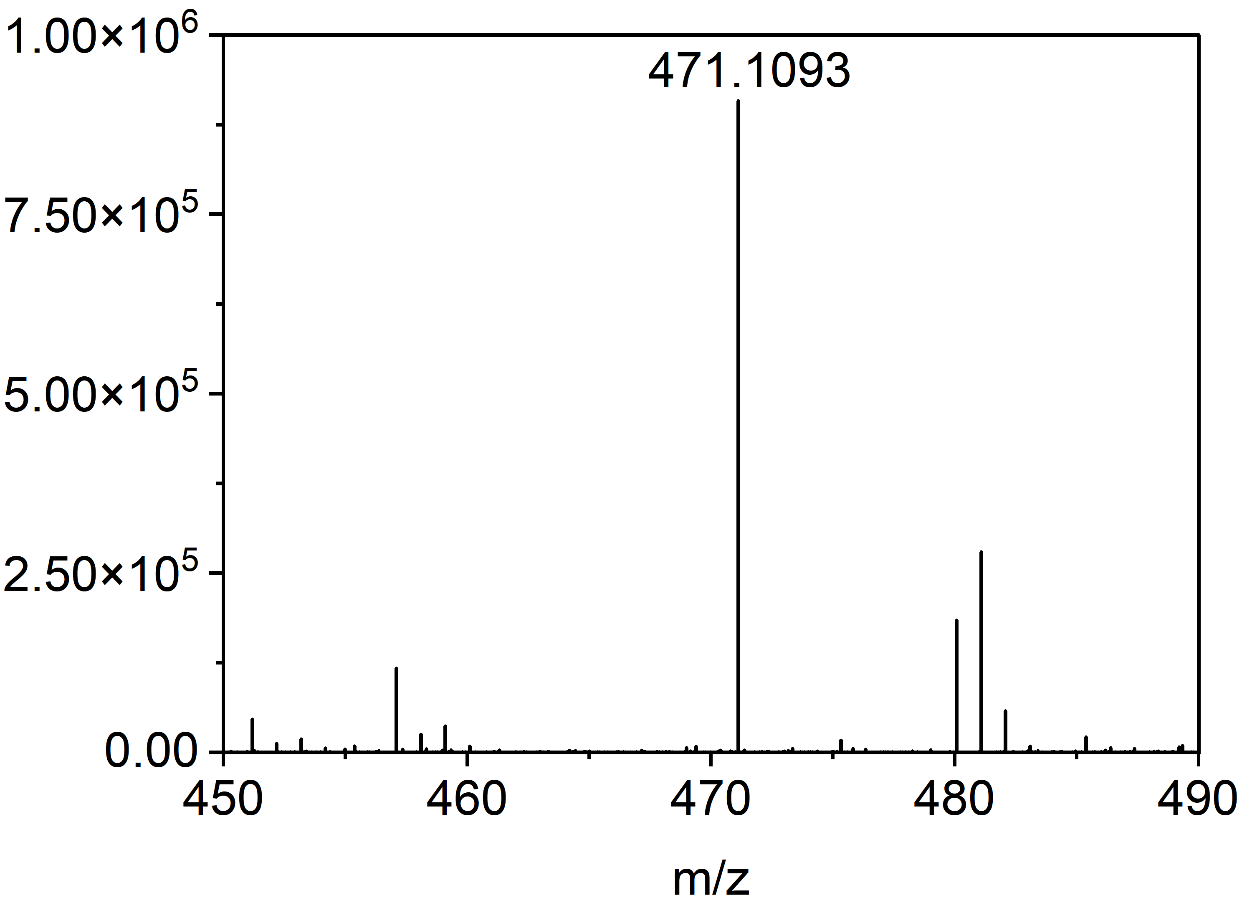
**

**Figure S95.** HRMS Spectrum of compound PyE26.

**
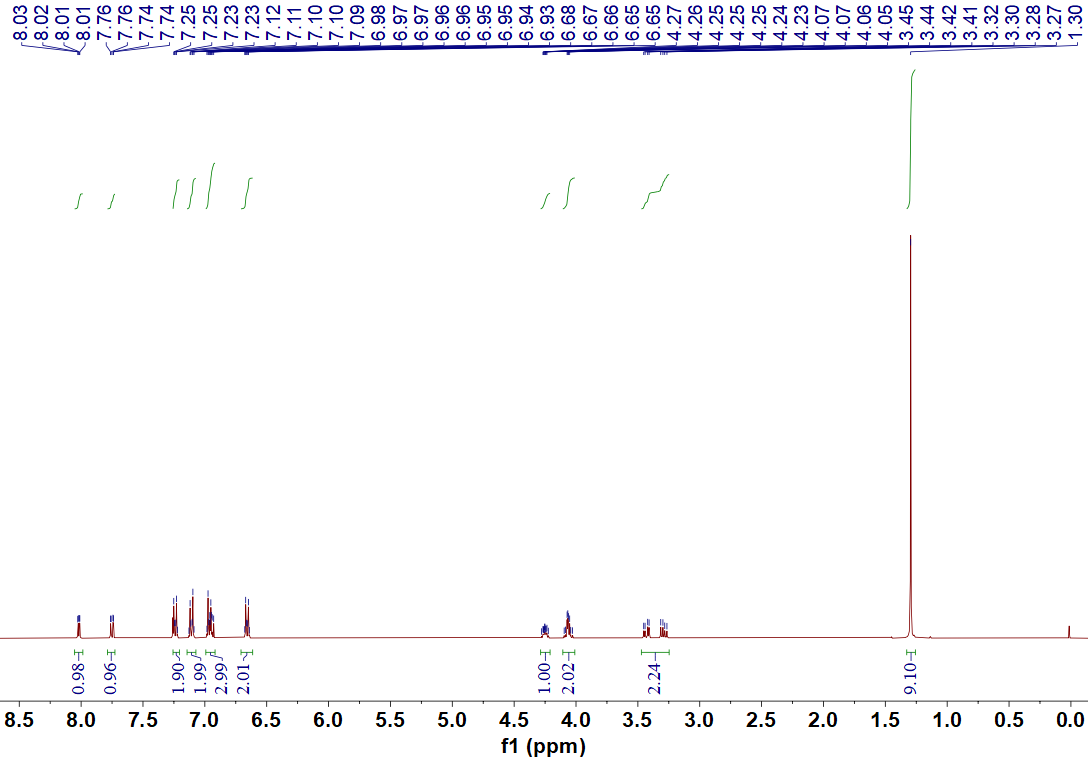
Figure S96.** ^1^H NMR Spectrum (CDCl_3_, 400 MHz) of compound PyE27.

**
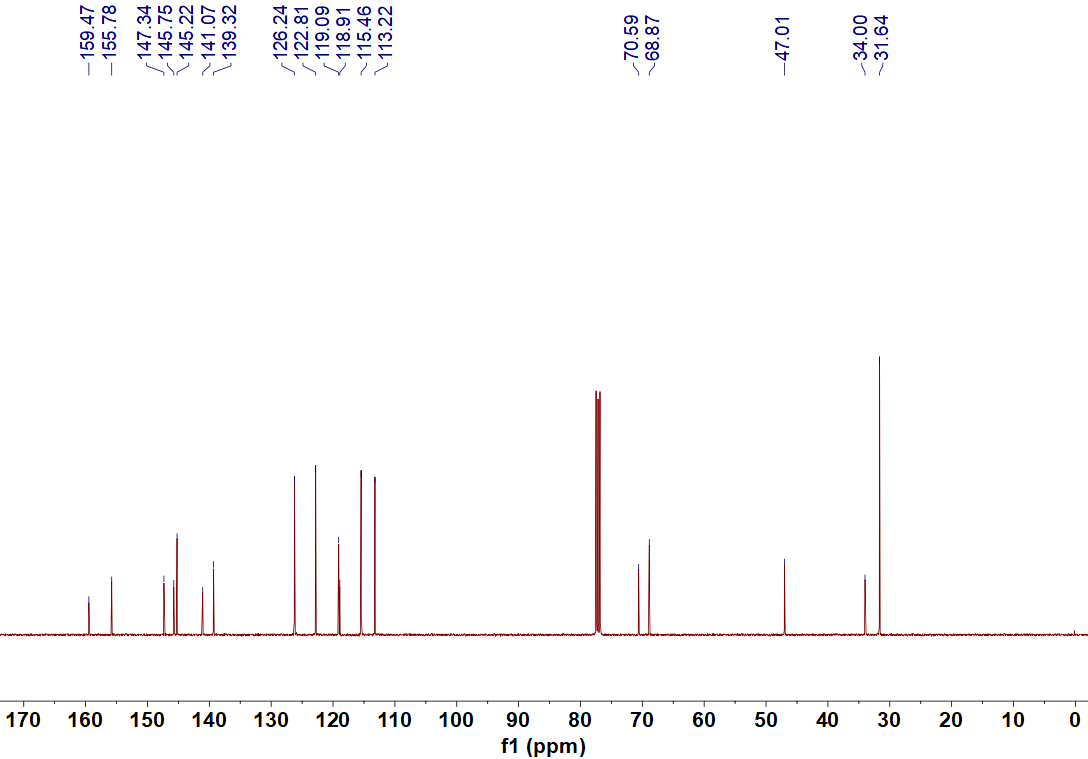
Figure S97.** ^13^C NMR Spectrum (CDCl_3_, 126 MHz) of compound PyE27.

**
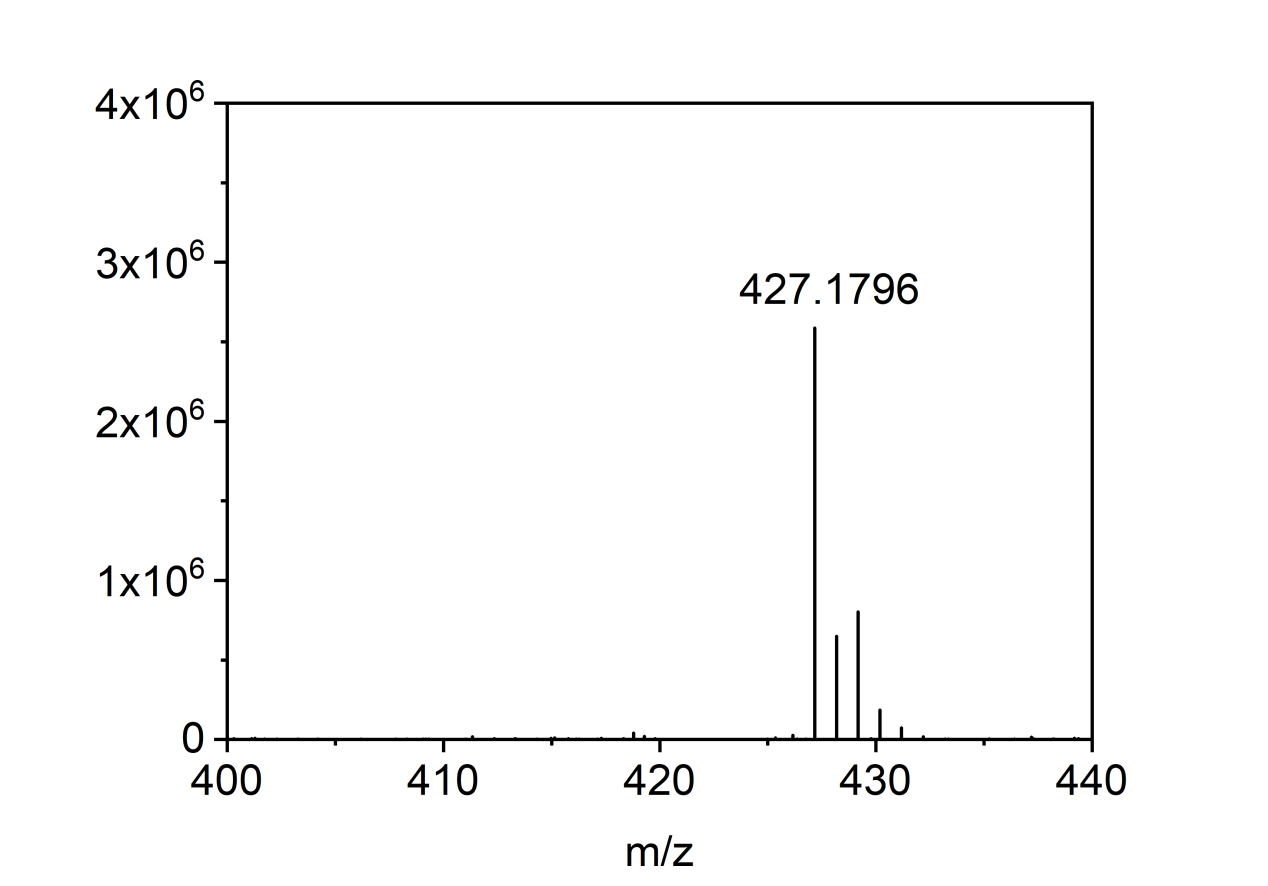
Figure S98.** HRMS Spectrum of compound PyE27.

**
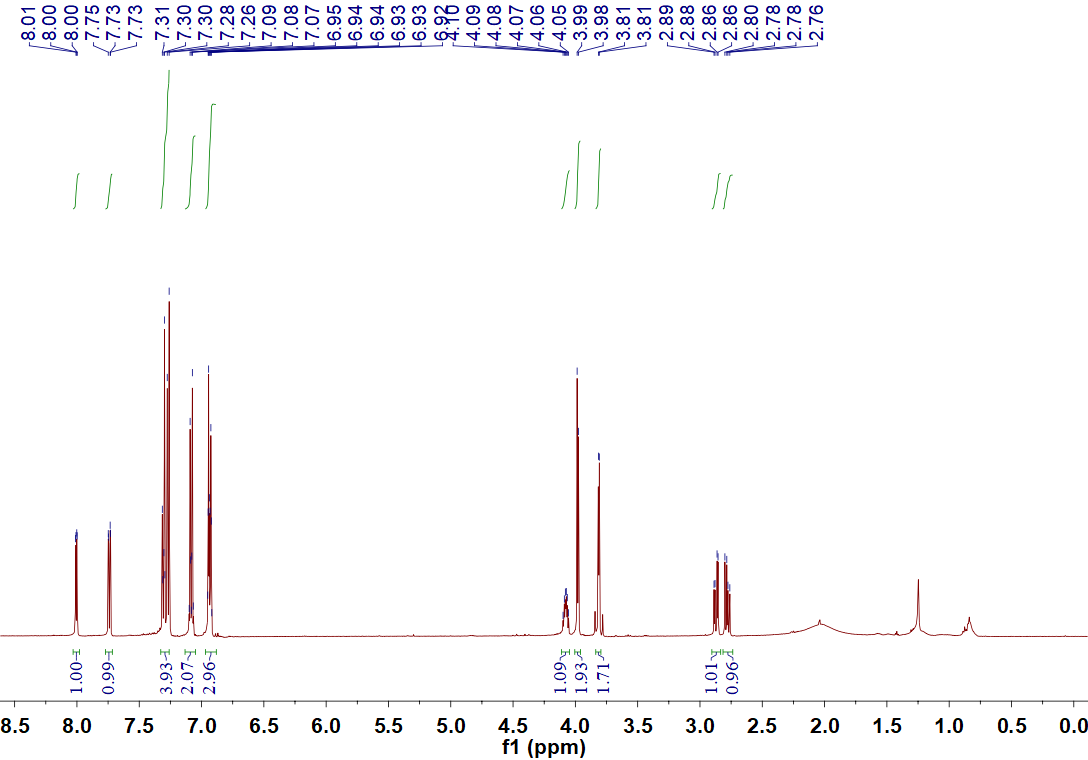
Figure S99.** ^1^H NMR Spectrum (CDCl_3_, 500 MHz) of compound PyE28.

**
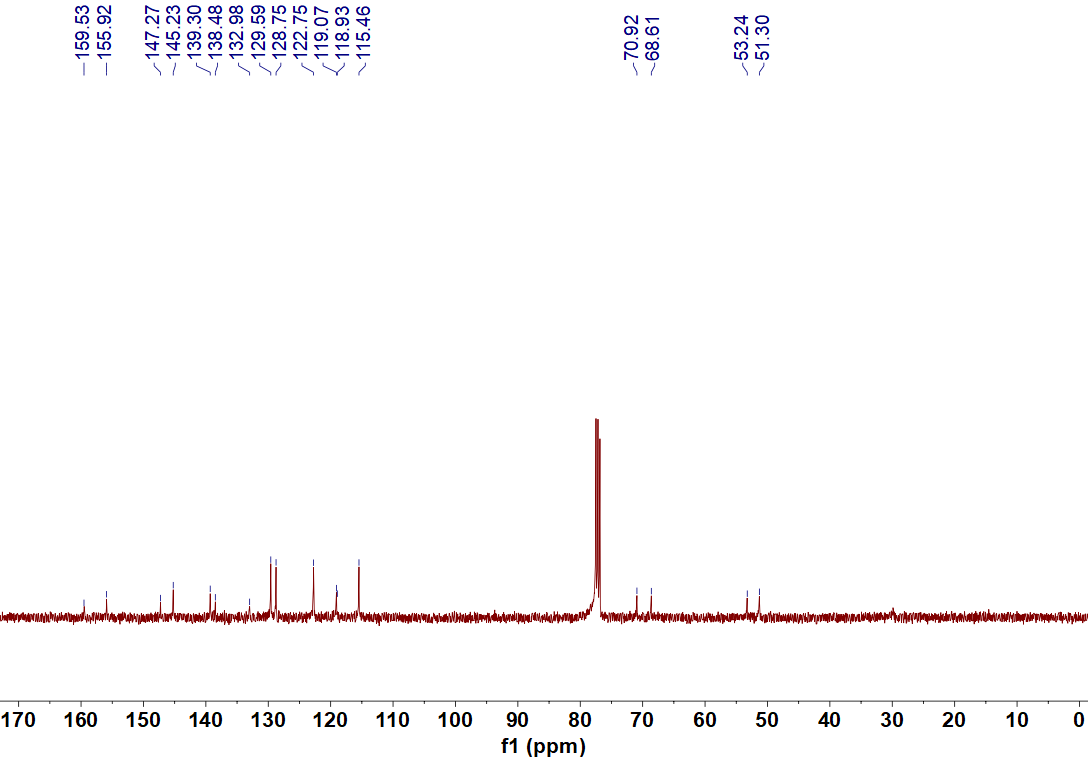
Figure S100.** ^13^C NMR Spectrum (CDCl_3_, 101 MHz) of compound PyE28.

**Figure S101.** HRMS Spectrum of compound PyE28.

**
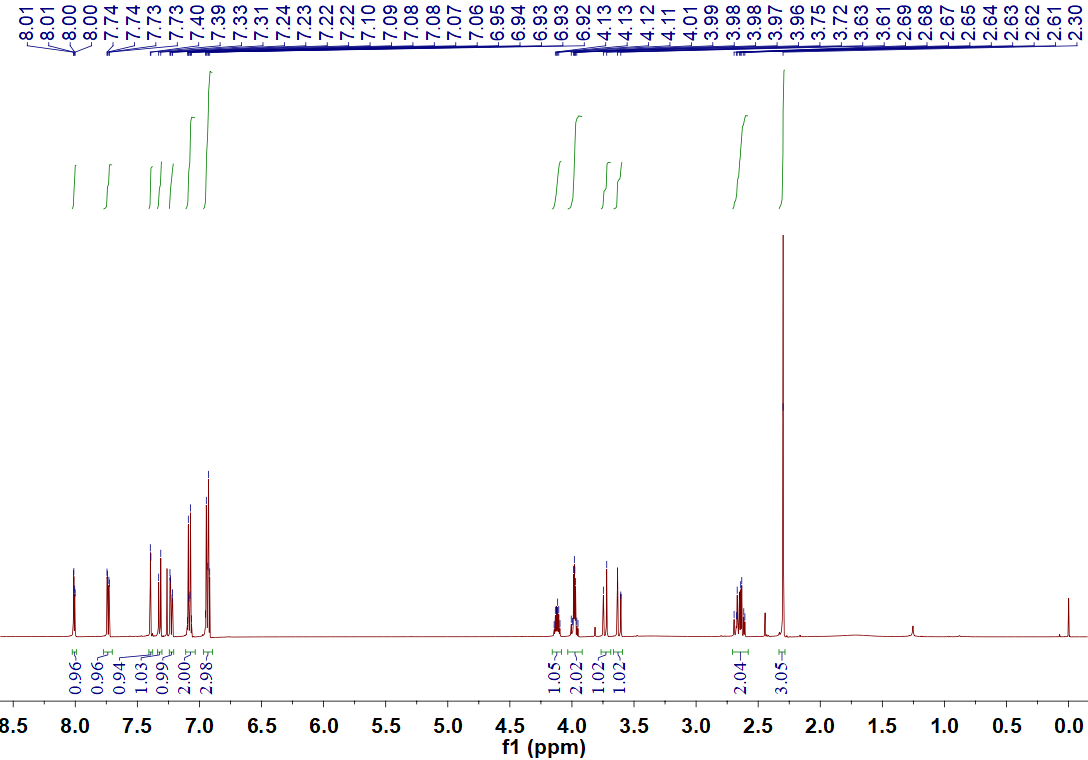
Figure S102.** ^1^H NMR Spectrum (CDCl_3_, 500 MHz) of compound PyE29.

**
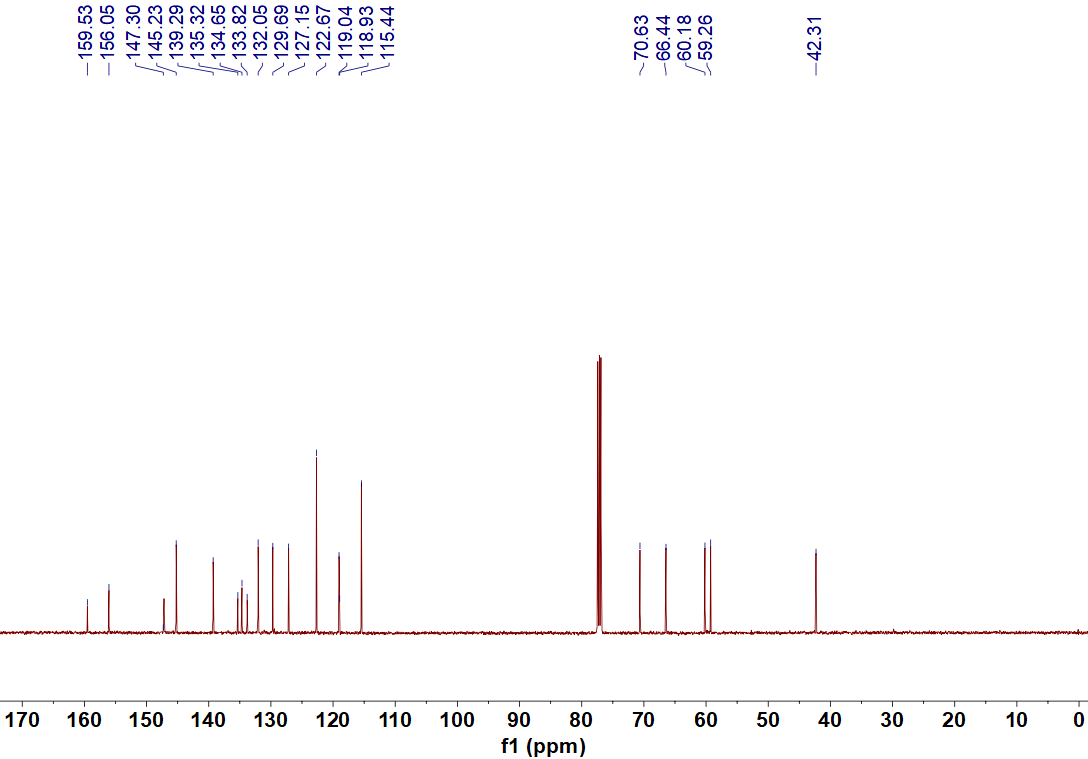
Figure S103.** ^13^C NMR Spectrum (CDCl_3_, 126 MHz) of compound PyE29.

**Figure S104.** HRMS Spectrum of compound PyE29.

**
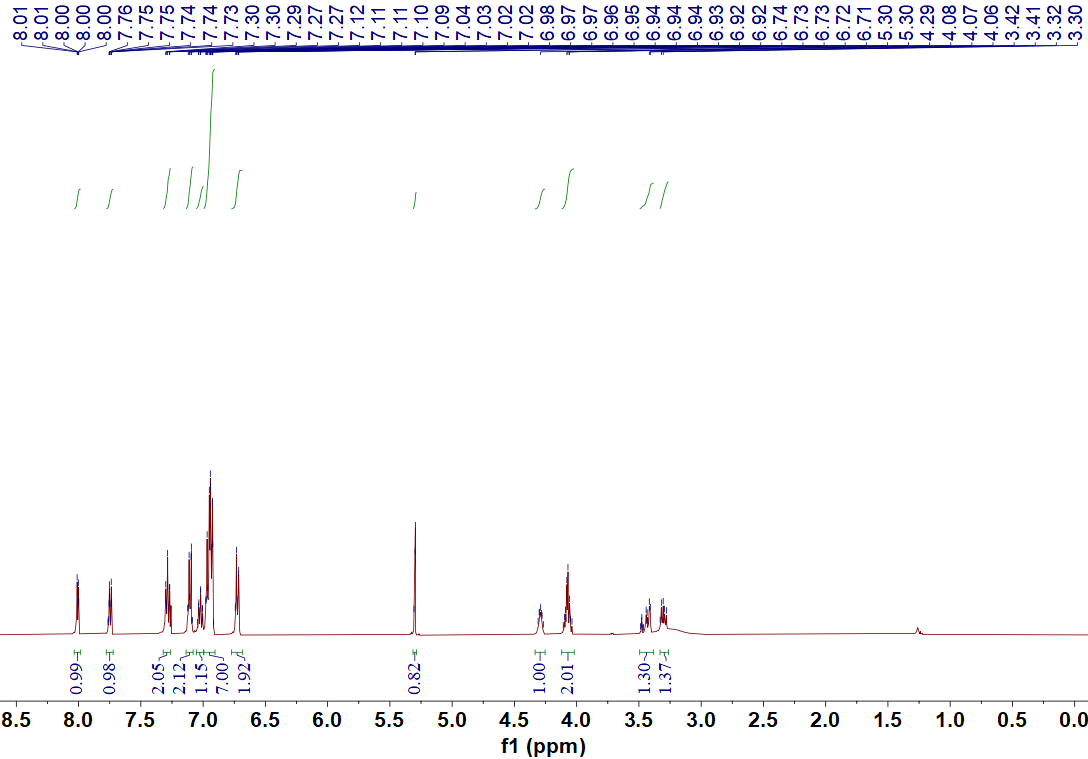
Figure S105.** ^1^H NMR Spectrum (CDCl_3_, 500 MHz) of compound PyE30.

**
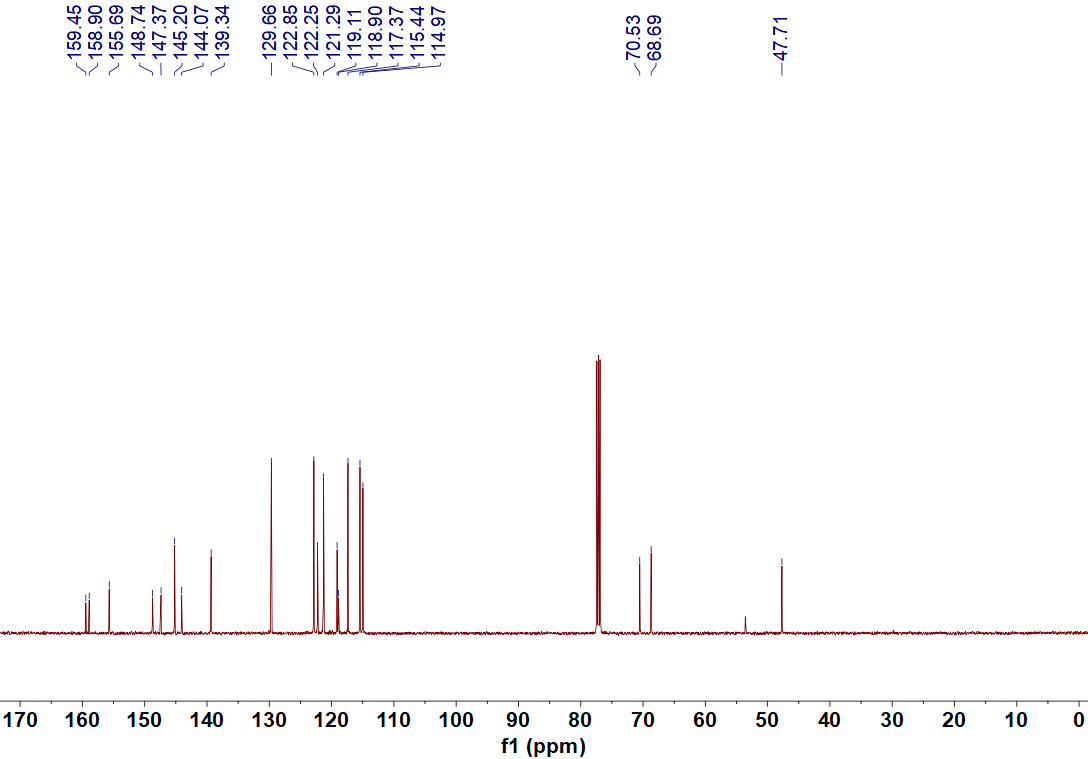
Figure S106.** ^13^C NMR Spectrum (CDCl_3_, 126 MHz) of compound PyE30.

**
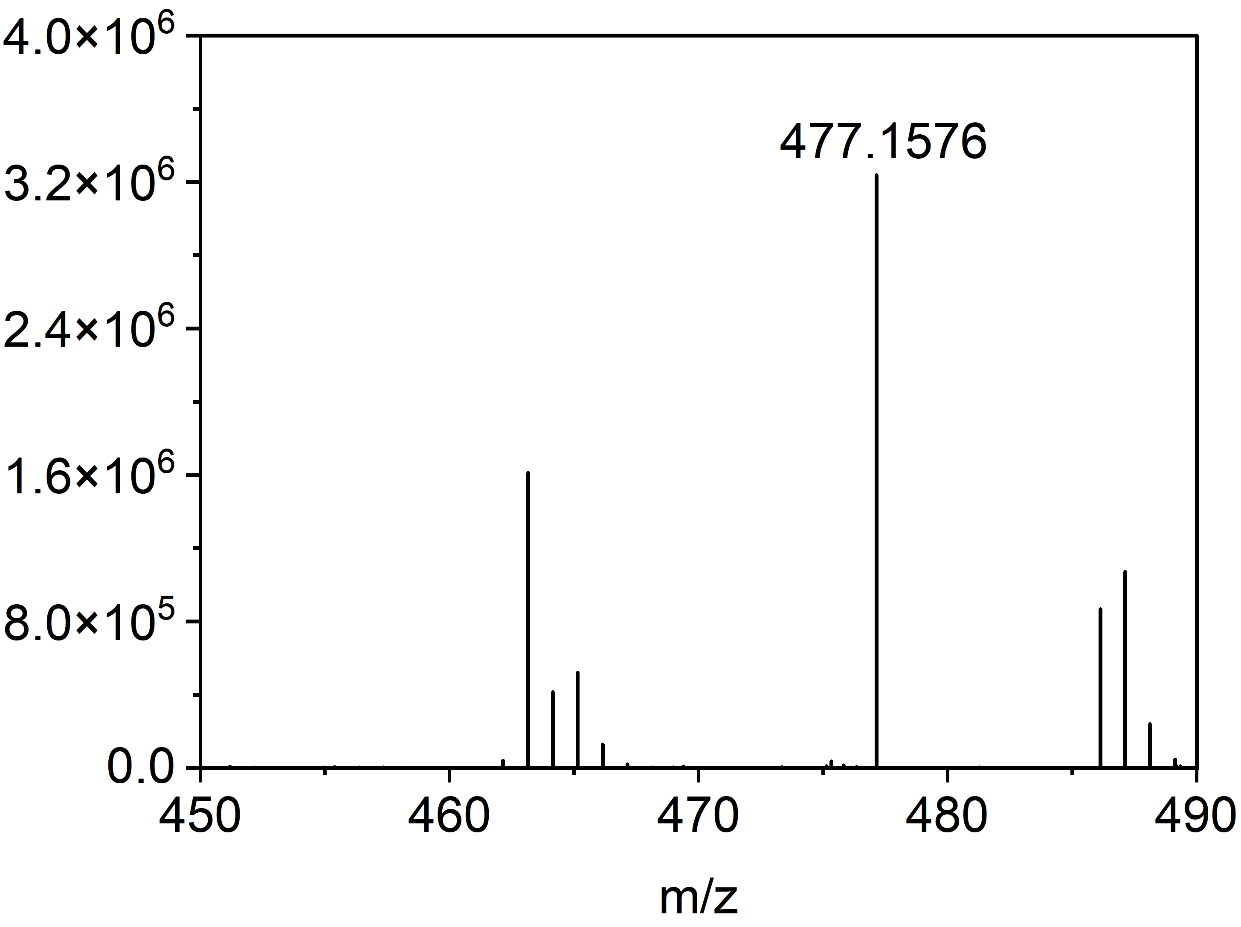
Figure S107.** HRMS Spectrum of compound PyE30.

**
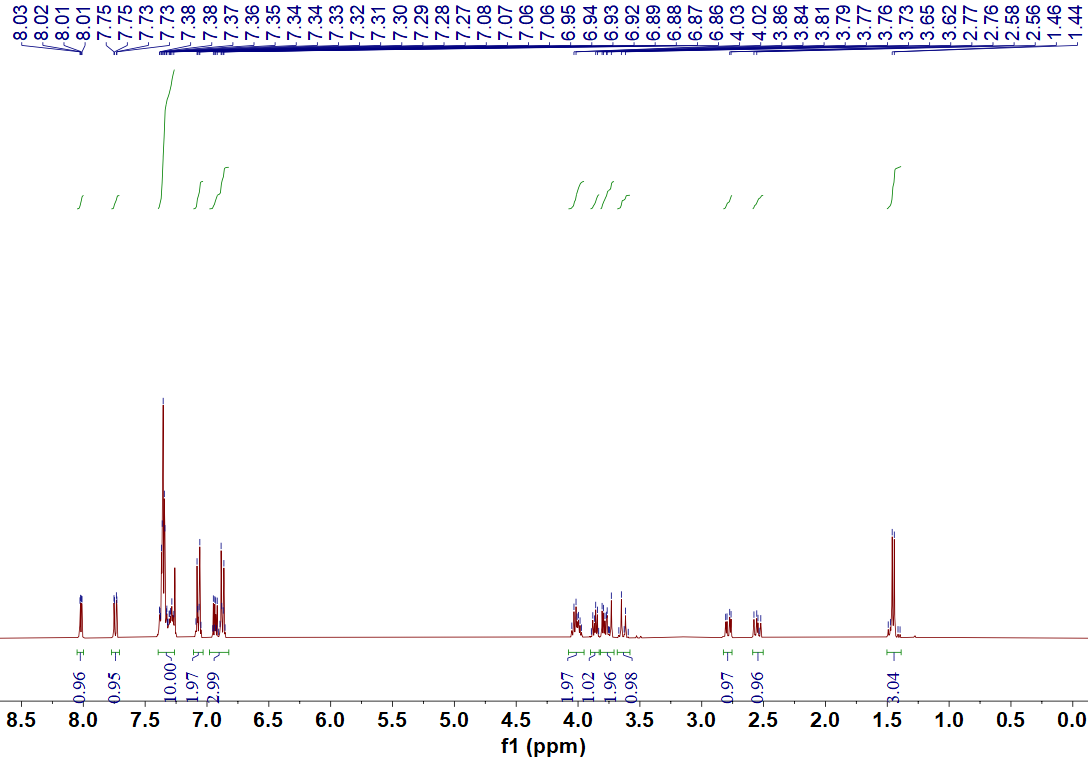
Figure S108.** ^1^H NMR Spectrum (CDCl_3_, 400 MHz) of compound PyE31.

**
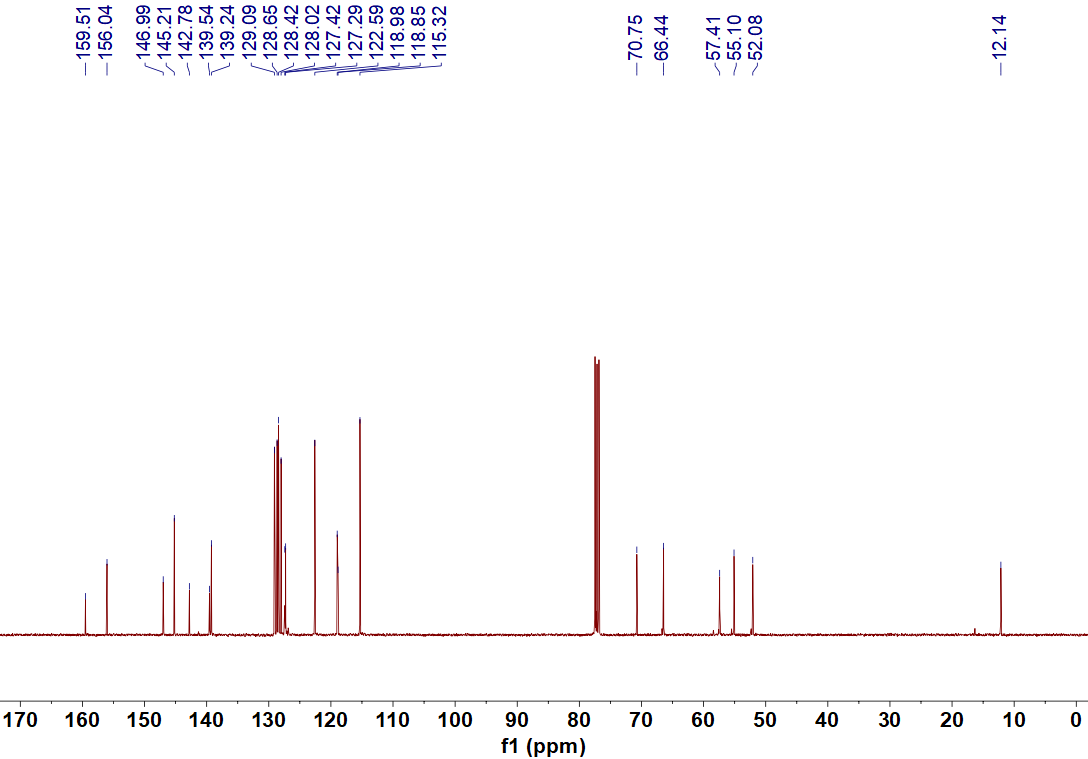
Figure S109.** ^13^C NMR Spectrum (CDCl_3_, 126 MHz) of compound PyE31.

**Figure S110.** HRMS Spectrum of compound PyE31.

**Figure S111.** ^1^H NMR Spectrum (CDCl_3_, 500 MHz) of compound PyE32.

**Figure S112.** ^13^C NMR Spectrum (CDCl_3_, 126 MHz) of compound PyE32.

**Figure S113.** HRMS Spectrum of compound PyE32.

**Figure S114.** ^1^H NMR Spectrum (CDCl_3_, 400 MHz) of compound PyE33.

**Figure S115.** ^13^C NMR Spectrum (CDCl_3_, 126 MHz) of compound PyE33.

**Figure S116.** HRMS Spectrum of compound PyE33.
